# Synthesis of aryl-substituted thieno[3,2-*b*]thiophene derivatives and their use for N,S-heterotetracene construction

**DOI:** 10.3762/bjoc.15.261

**Published:** 2019-11-12

**Authors:** Nadezhda S Demina, Nikita A Kazin, Nikolay A Rasputin, Roman A Irgashev, Gennady L Rusinov

**Affiliations:** 1 Postovsky Institute of Organic Synthesis, Ural Division, Russian Academy of Sciences, S. Kovalevskoy St., 22, Ekaterinburg, 620990, Russia; 2 Ural Federal University named after the first President of Russia B. N. Yeltsin, Mira St., 19, Ekaterinburg, 620002, Russia

**Keywords:** Fiesselmann thiophene synthesis, Fischer indole synthesis, N,S-heteroacene, thieno[3,2-*b*]thiophene, thieno[2',3':4,5]thieno[3,2-*b*]indole

## Abstract

Fiesselmann thiophene synthesis was applied for the convenient construction of thieno[3,2-*b*]thiophene derivatives. Thus, new 5- or 6-aryl-3-hydroxythieno[3,2-*b*]thiophene-2-carboxylates were obtained by condensation of 5- or 4-aryl-3-chlorothiophene-2-carboxylates, respectively, with methyl thioglycolate in the presence of potassium *tert*-butoxide. The saponification of the resulting esters, with decarboxylation of the intermediating acids, gave the corresponding thieno[3,2-*b*]thiophen-3(2*H*)-ones. The latter ketones were used to synthesize new N,S-heterotetracenes, namely 9*H*-thieno[2',3':4,5]thieno[3,2-*b*]indoles by their treatment with arylhydrazines in accordance with the Fischer indolization reaction.

## Introduction

**Figure 1:**
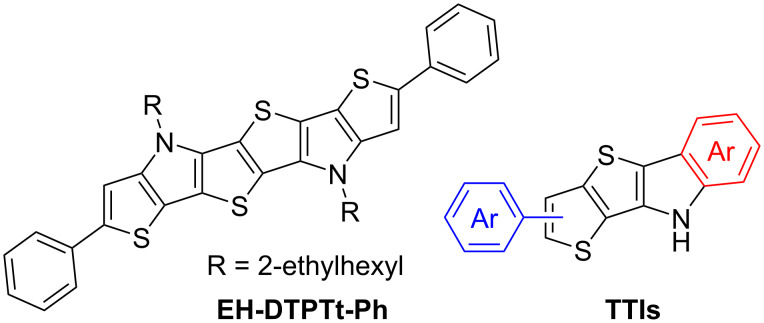
An example of an earlier developed S,N-heterohexacene [13] and general structure of compounds synthesized in this work.

The thieno[3,2-*b*]thiophene (TT) unit is highly demanded in modern organic synthesis since TT-based compounds have a variety of applications, e.g., in the field of organic electronics. Indeed, TT-based polymers and small molecules are used as light-harvesting dyes for dye-sensitized solar cells [1], electron-donating materials for bulk heterojunction solar cells [2–4], and p-type semiconductors for organic field-effect transistors [5–7]. Within the same context of organic semiconductor development, the bicyclic TT subunit has been used to architect various ring-fused S-heteroacenes, which have been studied extensively [8–10] due to their better characteristics compared to heteroatom-free acenes. For instance, S-heteroacenes have a better oxidation stability due to lower-lying HOMO levels, as well as more efficient charge transfer because of their tendency to π-stack, with non-bonded sulfur–sulfur interactions in the solid state, which results in large intermolecular orbital coupling of HOMOs and, as a consequence, enhanced carrier transport properties [10]. However, there is a problem of poor solubility of a number of S-heteroacenes, which can limit the effectiveness of semiconducting films in the case of solution-processable electronics [11]. Introducing the NH-containing moieties, e.g., pyrrole rings, followed by attaching solubilizing substituents is one of the ways to solve this problem. Thus, different types of N,S-heteroacenes, including ones with TT-scaffold (Figure 1), which have promising electronic features, have been developed and are further investigated [12–14].

At present, the challenge is to develop effective ways of constructing the TT-based compounds, such as S- and N,S-heteroacenes, in order to effectively tune their physical characteristics and also reduce production costs since the routine synthesis of heteroacene structures involves cross-coupling reactions catalyzed by expensive transition metals [15]. Thus, in this paper, we would like to present an efficient metal-free synthesis of the aryl-substituted TT building blocks, which can be utilized for the construction of various fused systems, including N,S-heteroacenes, e.g., substituted 9*H*-thieno[2',3':4,5]thieno[3,2-*b*]indoles (TTIs) (Figure 1).

## Results and Discussion

Continuing our previous work in which we used the Fiesselmann thiophene synthesis [16], viz., the interaction of ethyl thioglycolate with ethyl 3-chlorobenzo[*b*]thiophene-2-carboxylates, to form a benzo[*b*]thieno[2,3-*d*]thiophene scaffold [17–18], we have decided to apply a similar approach to the synthesis of aryl-substituted TT derivatives. Thus, 3-chloro- or 3-bromothiophene-2-carboxylates bearing aryl moieties at the C-5 or C-4 position were appropriate starting substrates to construct TT scaffolds according to our strategy. Compounds **2a–k** can be prepared either through direct palladium-catalyzed arylation of methyl 3-chlorothiophene-2-carboxylate [19] or through replacement of the amino group in the corresponding 3-aminothiophene-2-carboxylates with a halogen atom by the Sandmeyer reaction [20–21]. The former transformation is preferable for large-scale syntheses, but we failed to repeat the reported procedures. For example, our attempt to diazotizate methyl 3-amino-5-phenylthiophene-2-carboxylate (**1a**) with sodium nitrite in aqueous HCl or HBr solution failed due to the poor solubility of its hydrohalide salts, while when 3-amino ester **1a** was treated with *tert*-butyl nitrite and CuBr_2_ in acetonitrile solution, we observed only significant decomposition of starting compound.

**Scheme 1:**
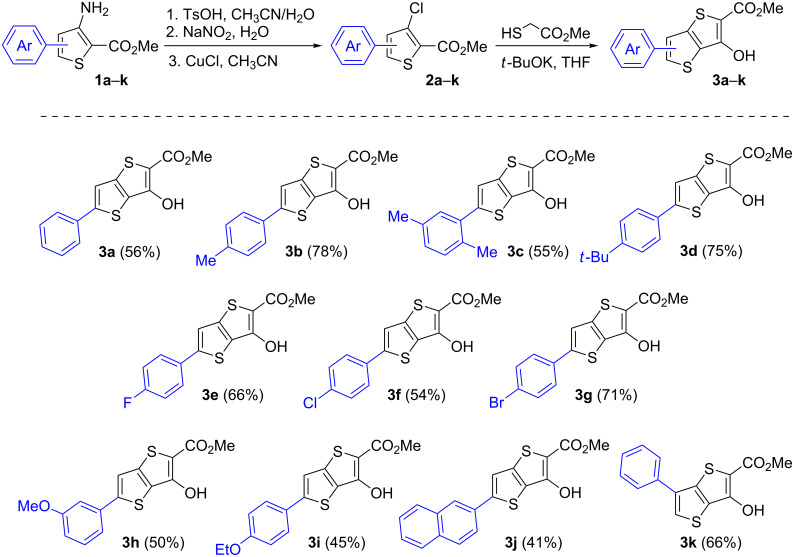
Synthesis of aryl-substituted TT derivatives **3a**–**k**, product scope, and yields.

In this respect, we elaborated our own procedure for the preparation of aryl-substituted methyl 3-chlorothiophene-2-carboxylates **2**, which consists of diazotization of the corresponding 3-aminothiophene-2-carboxylates **1** in acetonitrile solution with an aqueous sodium nitrite (1.1 equiv) solution in the presence of *p*-toluenesulfonic acid (4.0 equiv) followed by the addition of the formed solution of diazonium salts to a warm suspension of CuCl (5.0 equiv) in acetonitrile. Thus, compounds **2a**–**k** were obtained in 43–83% yield (Scheme 1, see Supporting Information File 1 for more experimental details). It should be noted that the initial 3-aminoesters **1** are known compounds and can be obtained by the Fiesselmann thiophene synthesis from aryl-substituted acrylonitriles bearing a good leaving group at the C-3 position [22–24].

Methyl 3-chlorothiophene-2-carboxylates **2a**–**k** were further involved in the Fiesselmann reaction with methyl thioglycolate in the presence of potassium *tert-*butoxide in THF, which afforded aryl-substituted methyl 3-hydroxythieno[3,2-*b*]thiophene-2-carboxylates **3a**–**k** in 41–78% yields (Scheme 1). Compounds **3** were obtained in analytically pure form after single recrystallization from a toluene/ethanol mixture (1:1, v/v), or pure toluene for compounds **3f**,**g**,**i**.

**Scheme 2:**
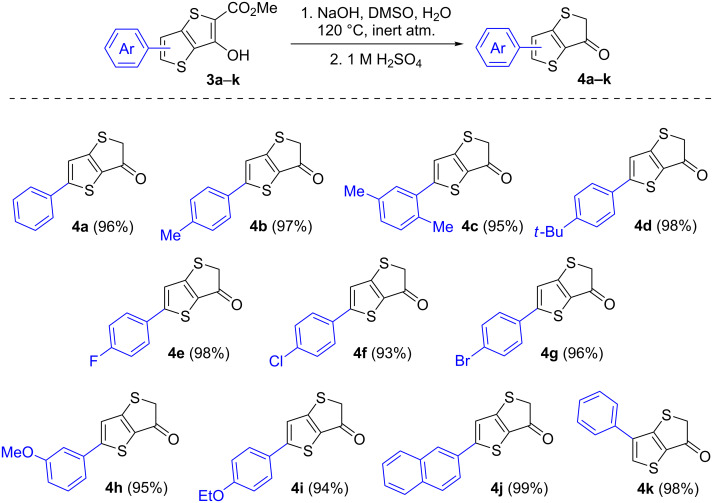
Synthesis of thieno[3,2-*b*]thiophen-3(2*H*)-one **4a**–**k**, product scope, and yields.

Next, we performed saponification of esters **3a**–**k** by their treatment with an excess of sodium hydroxyde in an aqueous DMSO solution at 120 °C for 3 hours, where the intermediating acids underwent decarboxylation to give thieno[3,2-*b*]thiophen-3(2*H*)-ones **4a**–**k** after neutralization of the reaction mixture with a mineral acid. It should be noted that the obtained ketones **4** were more sensitive to the presence of acids than their benzo-annelated counterparts [17–18]: when an excess of a mineral acid was used, we observed a decrease in yields of products **4** because of their partial degradation. Therefore, to neutralize the reaction mixtures, we used one equivalent of sulfuric acid relative to the alkali salt used for saponification. The resulting precipitates were then isolated by filtration, and thoroughly washed with water. As a result, we were able to isolate products in analytically pure form with yields of 93–99% (Scheme 2).

**Scheme 3:**
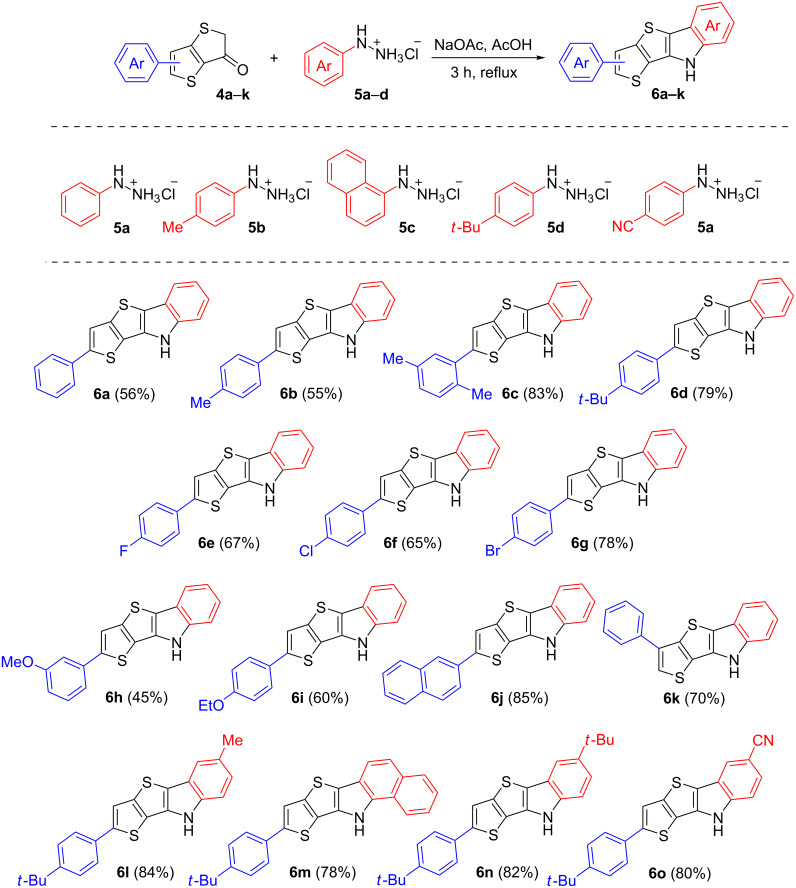
Synthesis of TTI derivatives **6a**–**o**, substrate and product scopes, and yields.

Both compounds **3** and **4** are new functional TT-cored building blocks, which can be useful for the development of various fused molecules, e.g., we used sources **4** to construct derivatives of new TTI ring systems according to the Fisсher indolization protocol [25]. To this end, compounds **4a**–**k** were treated with phenylhydrazine **5a** in acetic acid, thus affording compounds **6a**–**k** in 45–83% yields (Scheme 3). Notably, all the indolization reactions proceeded smoothly, the formed products precipitated from hot solutions during the process, and analytically pure forms of substances were isolated by simple filtration of cooled reaction mixtures, which were diluted with methanol. In order to test the reactivity of compounds **4** and expand the range of the TTI derivatives, ketone **4d** was similarly treated with arylhydrazines **5b**–**d** containing electron–acceptor substituents or electron–donor ones. There was no obvious change in the yields of compounds **6l**–**o** (78–84%, Scheme 3), which may indicate that the starting ketones had a rather high reactivity.

**Scheme 4:**
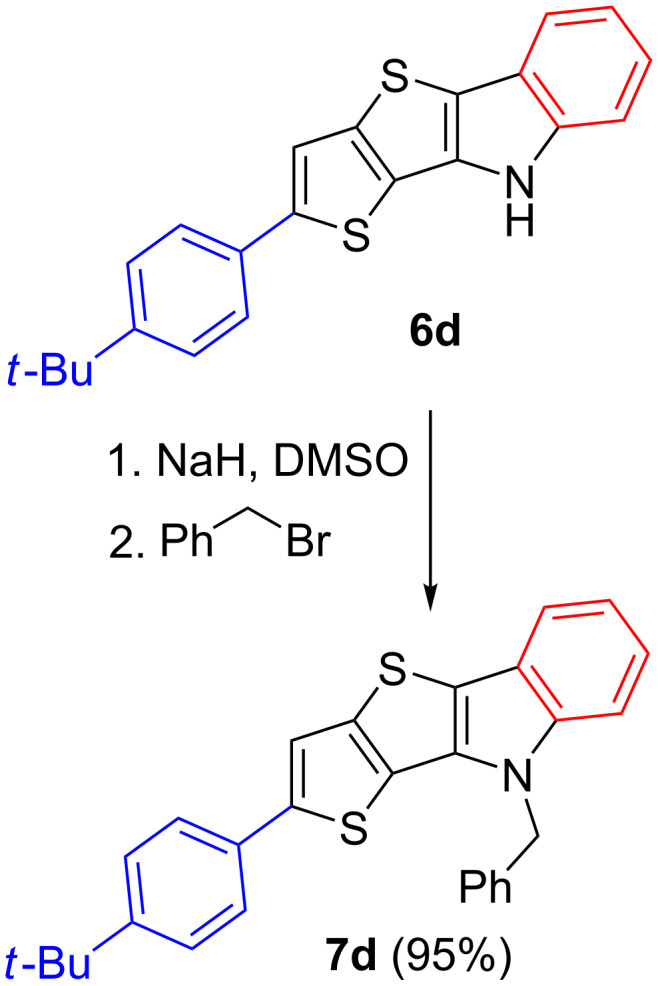
Alkylation of TTI **6d**.

**Figure 2:**
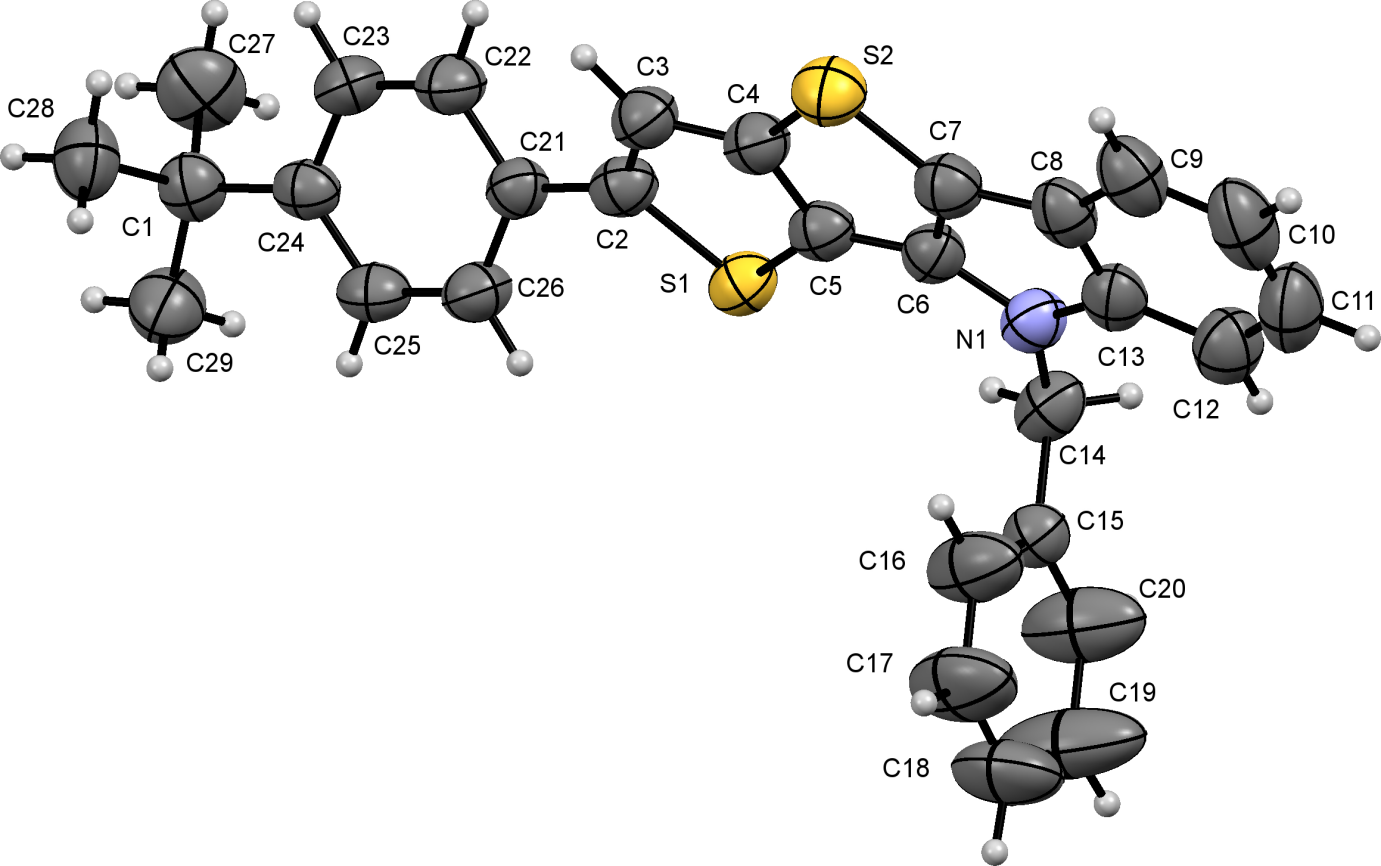
ORTEP diagram for the X-ray structure of compound **7d**. Thermal ellipsoids of 50% probability are shown.

To confirm the structure of TTI derivatives **6**, we attempted to grow crystals of some of these compounds, however, all of them formed powdery precipitates. Therefore, N-functionalization of compound **6d** was carried out with benzyl bromide in the presence of NaH (Scheme 4). The resulting benzyl derivative **7d** was crystallized from ethyl acetate to give crystals, which were suitable for XRD analysis (Figure 2).

## Conclusion

In summary, in this study, we showed the convenience of the Fiesselmann thiophene synthesis for the construction of new aryl-substituted thieno[3,2-*b*]thiophene derivatives, namely the reaction of 5- or 4-aryl-3-chlorothiophene-2-carboxylates with methyl thioglycolate in the presence of base to afford 5- or 6-aryl-3-hydroxythieno[3,2-*b*]thiophene-2-carboxylates. The latter 3-hydroxyesters as well as the thieno[3,2-*b*]thiophen-3(2*H*)-ones prepared this way can be considered as useful synthons for the construction of various heteroacenes, which was demonstrated by the synthesis of aryl-substituted 9*H*-thieno[2',3':4,5]thieno[3,2-*b*]indoles. These π-conjugated ring-fused molecules are of interest as electron-rich subunits for further organic semiconductors development.

## Supporting Information

**X-ray crystallographic data.** Deposition number CCDC 1944913 for compound **7d** contain the crystallographic data for these structures. These data can be obtained free of charge from the Cambridge Crystallographic Data Centre via https://www.ccdc.cam.ac.uk/data_request/cif.

File 1 Experimental section and copies of ^1^H, ^13^C and ^19^F NMR spectra of new compounds.

File 2 Crystal data of **7d**.
