# Supplementary material for: Synthesis of aryl-substituted thieno[3,2-b]thiophene derivatives and their use for N,S-heterotetracene construction
Source: Beilstein J Org Chem. 2019 Nov 12;15:2678–83. doi: 10.3762/bjoc.15.261 (PMC6880844; doi:10.3762/bjoc.15.261)
Supplement: File 1 — Experimental section and copies of 1H, 13C and 19F NMR spectra of new compounds. [file Beilstein_J_Org_Chem-15-2678-s001.pdf]

BEILSTEIN JOURNAL OF ORGANIC CHEMISTRY

Supporting Information

for

Synthesis of aryl-substituted thieno[3,2-*b*]thiophene derivatives and their use for N,S-heterotetracene construction

Nadezhda S. Demina, Nikita A. Kazin, Nikolay A. Rasputin, Roman A. Irgashev and Gennady L. Rusinov

Beilstein J. Org. Chem. **2019,** *15,* 2678–2683. doi:10.3762/bjoc.15.261

Experimental section and copies of ^{1}H, ^{13}C and ^{19}F NMR spectra of new compounds

License and Terms: This is a supporting information file under the terms of the Creative Commons Attribution License (http://creativecommons.org/licenses/by/4.0). Please note that the reuse, redistribution and reproduction in particular requires that the authors and source are credited.
The license is subject to the *Beilstein Journal of Organic Chemistry* terms and conditions: (https://www.beilstein-journals.org/bjoc)

Supporting Information

Table of contents

Experimental section S2

1. General procedure for the synthesis of methyl 3-chloro-5(4)-arylthiophene-2-carboxylates (**2**) S2

2. General procedure for the synthesis of methyl 3-hydroxy-5(6)-arylthieno[3,2-*b*]thiophene-2-carboxylates (**3**) S5

3. General procedure for the synthesis of 5(6)-arylthieno[3,2-*b*]thiophen-3(2*H*)-ones (**4**) S7

4. General procedure for the synthesis of 2(3)-aryl-9*H*-thieno[2',3':4,5]thieno[3,2-*b*]indoles (**6**) S10

Copies of ^{1}H, ^{13}C and ^{19}F NMR spectra of new compounds S15

Crystallographic data and results of refinement for the structure **7d** in the XRD experiment S109

References S110

Experimental section

Instruments and measurements

Analytical studies were carried out using equipment of the Center for Joint Use “Spectroscopy and Analysis of Organic Compounds” at the Postovsky Institute of Organic Synthesis of the Russian Academy of Sciences (Ural Division). Melting points were determined on combined heating stages and are uncorrected. Elemental analysis was carried on an automated CHN analyzer. Mass spectrometry was performed using a high resolution Q-TOF LC–MS/MS spectrometer. NMR measurements were performed on NMR spectrometers at 400 MHz, 500 MHz and 600 Hz for ^{1}H, and 126 MHz for ^{13}C spectra in DMSO-d_6 or $CDCl_3$ with tetramethylsilane as an internal standard. ^{19}F NMR was recorded at 471 MHz in DMSO-d_6 or $CDCl_3$ with hexafluorobenzene as an internal standard. X-ray diffraction analysis was performed on an automated X-ray diffractometer on standard procedure.

Materials

Silica gel (230−450 mesh) was used for chromatography. All solvents used were dried and distilled per standard procedures. All reagents were purchased from commercial sources and used without further purification, except for 3-amino-5-arylthiophene-2-carboxylates **1a**–**j**, which were synthesized by using earlier reported procedures [1–3].

1. General procedure for the synthesis of methyl 3-chloro-5(4)-arylthiophene-2-carboxylates (2)

The appropriate 3-amino-2-thenoate **1** (14 mmol) and TsOH·H_2O (10.64 g, 56 mmol) were added to a mixture of acetonitrile (100 ml) and water (25 ml). The mixture was heated until a homogeneous solution was obtained, and then cooled in an ice bath with vigorous stirring to obtain a finely dispersed precipitate of the aminium tosylate salt. The solution of $NaNO_2$ (1.10 g, 16 mmol) in water (4 ml) was added in one portion to the obtained suspension. The resulting dark-red solution was added dropwise to the boiled suspension of CuCl (6.93 g, 70 mmol) in acetonitrile (40 ml) under the inert atmosphere. The mixture was boiled for 5 minutes, while nitrogen was observed, after that it was diluted with water (300 ml) and extracted with methylene chloride (3 × 40 ml). The organic layers were combined, dried with K_2CO_3, passed through a short layer of SiO_2, and then concentrated under reduced pressure. The resulting residue of **2** was recrystallized from

ethyl acetate : isopropanol (1:1, v/v), except for **2c**, which was obtained in analytically pure form after the column chromatography.

1.1. Methyl 3-chloro-5-phenylthiophene-2-carboxylate (2a)

Beige powder (1.80 g, 51%), m.p. (78-79 °C). ^{1}H NMR (400 MHz, DMSO-*d*$_6$) δ 7.87 – 7.69 (m, 3H), 7.56 – 7.34 (m, 3H), 3.84 (s, 3H). ^{13}C NMR (126 MHz, Chloroform-*d*) δ 161.0, 148.5, 132.2, 131.9, 129.4, 129.2, 125.9, 125.7, 123.9, 52.2. Anal. Calcd for $C_{12}H_9ClO_2S$: C, 57.03; H, 3.59; Found: C, 57.15; H, 3.67.

1.2. Methyl 3-chloro-5-(*p*-tolyl)thiophene-2-carboxylate (2b) [4]

Beige powder (2.84 g, 76%), m.p. (103-104 °C). ^{1}H NMR (500 MHz, Chloroform-*d*) δ 7.51 – 7.46 (m, 2H), 7.27 – 7.18 (m, 2H), 7.17 (s, 1H), 3.91 (s, 3H), 2.39 (s, 3H). NMR data agrees with previously reported data [4].

1.3. Methyl 5-(2,5-dimethylphenyl)-3-chlorothiophene-2-carboxylate (2c)

Light orange viscous oil (3.27 g, 83%). ^{1}H NMR (400 MHz, DMSO-*d*$_6$) δ 7.38 (s, 1H), 7.29 – 7.27 (m, 1H), 7.24 (d, *J* = 7.8 Hz, 1H), 7.20 – 7.15 (m, 1H), 3.85 (s, 3H), 2.36 (s, 3H), 2.32 – 2.27 (m, 3H). ^{13}C NMR (126 MHz, Chloroform-*d*) δ 161.1, 148.3, 135.8, 132.9, 131.9, 131.0, 130.9, 130.5, 130.0, 129.0, 124.5, 52.1, 20.8, 20.4. Anal. Calcd for $C_{14}H_{13}ClO_2S$: C, 59.89; H, 4.67; Found: C, 59.80; H, 4.77.

1.4. Methyl 5-(4-(*tert*-butyl)phenyl)-3-chlorothiophene-2-carboxylate (2d) [4]

Cream crystals (2.51 g, 58%), m.p. (80-81 °C). ^{1}H NMR (400 MHz, DMSO-*d*$_6$) δ 7.75 – 7.62 (m, 3H), 7.53 – 7.44 (m, 2H), 3.84 (s, 3H), 1.30 (s, 9H). NMR data agrees with previously reported data [4].

1.5. Methyl 5-(4-fluorophenyl)-3-chlorothiophene-2-carboxylate (2e) [4]

Beige powder (2.42 g, 64%), m.p. (153-154 °C). ^{1}H NMR (500 MHz, DMSO-*d*$_6$) δ 7.90 – 7.80 (m, 2H), 7.72 (s, 1H), 7.39 – 7.28 (m, 2H), 3.85 (s, 3H). NMR data agrees with previously reported data [4].

1.6. Methyl 5-(4-chlorophenyl)-3-chlorothiophene-2-carboxylate (2f) [4]

Cream powder (2.73 g, 68%), m.p. (156-157 °C). ^{1}H NMR (400 MHz, DMSO-*d*$_6$) δ 7.85 – 7.79 (m, 2H), 7.78 (s, 1H), 7.59 – 7.51 (m, 2H), 3.85 (s, 3H). NMR data agrees with previously reported data [4].

1.7. Methyl 5-(4-bromophenyl)-3-chlorothiophene-2-carboxylate (2g)

Beige powder (2.46 g, 53%), m.p. (141-142 °C). ^{1}H NMR (500 MHz, DMSO-d_6) δ 7.78 (s, 1H), 7.75 – 7.71 (m, 2H), 7.70 – 7.65 (m, 2H), 3.85 (s, 3H). ^{13}C NMR (126 MHz, DMSO-d_6) δ 160.0, 146.5, 132.2, 131.0, 130.7, 127.7, 126.9, 123.6, 123.1, 52.4. Anal. Calcd for $C_{12}H_8BrClO_2S$: C, 43.46; H, 2.43; Found: C, 43.40; H, 2.29.

1.8. Methyl 3-chloro-5-(3-methoxyphenyl)thiophene-2-carboxylate (2h)

Beige powder (1.70 g, 43%), m.p. (63-64 °C). ^{1}H NMR (400 MHz, DMSO-d_6) δ 7.77 (s, 1H), 7.45 – 7.35 (m, 1H), 7.35 – 7.29 (m, 2H), 7.06 – 6.98 (m, 1H), 3.85 (s, 3H), 3.83 (s, 3H). ^{13}C NMR (126 MHz, DMSO-d_6) δ 160.0, 159.8, 147.8, 132.7, 130.9, 130.5, 126.6, 123.2, 118.1, 115.7, 110.9, 55.3, 52.4. Anal. Calcd for $C_{13}H_{11}ClO_3S$: C, 55.23; H, 3.92; Found: C, 55.11; H, 3.79.

1.9. Methyl 5-(4-ethoxyphenyl)-3-chlorothiophene-2-carboxylate (2i)

Dark yellow crystals (2.32g, 56%), m.p. (101-102 °C). ^{1}H NMR (500 MHz, DMSO-d_6) δ 7.74 – 7.65 (m, 2H), 7.61 (s, 1H), 7.05 – 6.97 (m, 2H), 4.13 – 4.06 (m, 2H), 3.83 (s, 3H), 1.34 (t, J = 6.9 Hz, 3H). ^{13}C NMR (126 MHz, DMSO-d_6) δ 160.1, 159.8, 148.3, 131.0, 127.3, 124.9, 123.9, 121.8, 115.1, 63.3, 52.2, 14.5. Anal. Calcd for $C_{14}H_{13}ClO_3S$: C, 56.66; H, 4.42; Found: C, 56.79; H, 4.40.

1.10. Methyl 3-chloro-5-(naphthalen-2-yl)thiophene-2-carboxylate (2j) [4]

Beige crystals (1.99 g, 47%), m.p. (141-142 °C).. ^{1}H NMR (500 MHz, DMSO-d_6) δ 8.40 (d, J = 1.8 Hz, 1H), 8.04 – 7.99 (m, 2H), 7.98 – 7.94 (m, 1H), 7.91 (dd, J = 8.6, 1.9 Hz, 1H), 7.88 (s, 1H), 7.58 (tt, J = 5.6, 4.6 Hz, 2H), 3.87 (s, 3H). NMR data agrees with previously reported data [4].

1.11. Methyl 3-chloro-4-phenylthiophene-2-carboxylate (2k)

Cream crystals (2.87 g, 81%), m.p. (82-83 °C). ^{1}H NMR (500 MHz, DMSO-d_6) δ 8.09 (s, 1H), 7.54 – 7.47 (m, 5H), 3.87 (s, 3H). ^{13}C NMR (126 MHz, DMSO-d_6) δ 160.3, 141.5, 133.3, 130.2, 128.9, 128.8, 128.5, 128.2, 125.9, 52.4. Anal. Calcd for $C_{12}H_9ClO_2S$: C, 57.03; H, 3.59; Found: C, 57.25; H, 3.69.

2. General procedure for the synthesis of methyl 3-hydroxy-5(6)-arylthieno[3,2-*b*]thiophene-2-carboxylates 3

Potassium *tert*-butoxide (2.24 g, 20 mmol) was dissolved in dry THF (50 ml) under the inert atmosphere, and methyl thioglycolate (1.79 ml, 20 mmol) was added. After allowing the mixture to stir for 15 minutes, the appropriate compound **2** (10 mmol) was added, and the mixture was vigorously stirred for 4 hours at room temperature. Then the second portion of potassium *tert*-butoxide (2.24 g, 20 mmol) was added, and the resulting mixture was heated at 60 °C for another 4 hours. After that, the reaction mixture was cooled, poured into waster, diluted with 0.1 M solution of hydrochloric acid (50 ml), and the formed precipitate was filtered off, washed with water and recrystallized from toluene/ethanol mixture (1:1, v/v) for **3a–e,h,j,k** or from toluene for **3f,g,i**.

2.1. Methyl 3-hydroxy-5-phenylthieno[3,2-*b*]thiophene-2-carboxylate (3a)

Beige crystals (1.62 g, 56%), m.p. (168-169 °C). ^{1}H NMR (400 MHz, DMSO-d_6) δ 10.97 (s, 1H), 7.87 (s, 1H), 7.77 – 7.69 (m, 2H), 7.53 – 7.44 (m, 2H), 7.46 – 7.36 (m, 1H), 3.80 (s, 3H). ^{13}C NMR (126 MHz, DMSO-d_6) δ 163.2, 153.6, 149.9, 140.7, 133.2, 129.3, 128.9, 128.3, 125.7, 117.3, 105.5, 51.6. Anal. Calcd for $C_{14}H_{10}O_3S_2$: C, 57.91; H, 3.47; Found: C, 57.85; H, 3.52.

2.2. Methyl 3-hydroxy-5-(*p*-tolyl)thieno[3,2-*b*]thiophene-2-carboxylate (3b)

Light orange powder (2.37 g, 78%), m.p. (192-193 °C). ^{1}H NMR (400 MHz, DMSO-d_6) δ 10.93 (s, 1H), 7.82 (s, 1H), 7.67 – 7.59 (m, 2H), 7.29 (d, J = 8.0 Hz, 2H), 3.80 (s, 3H), 2.34 (s, 3H). ^{13}C NMR (126 MHz, DMSO-d_6) δ 163.3, 153.7, 150.2, 140.8, 138.6, 130.4, 129.9, 127.9, 125.6, 116.7, 105.1, 51.6, 20.8. Anal. Calcd for $C_{15}H_{12}O_3S_2$: C, 59.19; H, 3.97; Found: C, 59.29; H, 4.02.

2.3. Methyl 5-(2,5-dimethylphenyl)-3-hydroxythieno[3,2-*b*]thiophene-2-carboxylate (3c)

Beige crystals (1.75 g, 55%), m.p. (111-112 °C). ^{1}H NMR (500 MHz, DMSO-d_6) δ 10.94 (s, 1H), 7.52 (s, 1H), 7.30 – 7.22 (m, 2H), 7.16 (dd, J = 7.9, 1.8 Hz, 1H), 3.81 (s, 3H), 2.37 (s, 3H), 2.31 (s, 3H). ^{13}C NMR (126 MHz, DMSO-d_6) δ 163.4, 153.6, 149.1, 140.0, 135.4, 132.7, 132.5, 131.0, 130.4, 129.5, 128.9, 120.5, 105.0, 51.6, 20.4, 20.23. Anal. Calcd for $C_{16}H_{14}O_3S_2$: C, 60.36; H, 4.43; Found: C, 60.22; H, 4.30.

2.4. Methyl 5-(4-(*tert*-butyl)phenyl)-3-hydroxythieno[3,2-*b*]thiophene-2-carboxylate (3d)

Beige powder (2.60 g, 75%), m.p. (173-174 °C). ^{1}H NMR (400 MHz, Chloroform-*d*) δ 10.12 (s, 1H), 7.62 – 7.54 (m, 2H), 7.49 – 7.41 (m, 2H), 7.37 (s, 1H), 3.93 (s, 3H), 1.35 (s, 9H). ^{13}C NMR (126 MHz, Chloroform-*d*) δ 167.4, 157.6, 152.3, 152.1, 142.1, 131.0, 127.1, 126.1, 125.9, 115.6, 103.1, 51.9, 34.8, 31.2. Anal. Calcd for $C_{18}H_{18}O_3S_2$: C, 62.40; H, 5.24; Found: C, 62.29; H, 5.32.

2.5. Methyl 5-(4-fluorophenyl)-3-hydroxythieno[3,2-*b*]thiophene-2-carboxylate (3e)

Light orange powder (2.03 g, 66%), m.p. (200-201 °C). ^{1}H NMR (500 MHz, DMSO-d_6) δ 10.99 (s, 1H), 7.85 (s, 1H), 7.83 – 7.74 (m, 2H), 7.39 – 7.29 (m, 2H), 3.81 (s, 3H). ^{13}C NMR (126 MHz, Chloroform-*d*) δ 164.1, 162.1, 150.6, 142.0, 130.1, 128.0, 128.0, 127.4, 116.3, 116.1, 96.1, 52.0. ^{19}F NMR (471 MHz, Chloroform-*d*) δ 49.62 (tt, *J* = 8.4, 5.1 Hz). Anal. Calcd for $C_{14}H_9FO_3S_2$: C, 54.54; H, 2.94; Found: C, 54.45; H, 3.00.

2.6. Methyl 5-(4-chlorophenyl)-3-hydroxythieno[3,2-*b*]thiophene-2-carboxylate (3f)

Light brown powder (1.75 g, 54%), m.p. (221-222 °C). ^{1}H NMR (500 MHz, DMSO-d_6) δ 11.03 (s, 1H), 7.91 (s, 1H), 7.81 – 7.72 (m, 2H), 7.59 – 7.51 (m, 2H), 3.81 (s, 3H). ^{13}C NMR (126 MHz, DMSO-d_6) δ 163., 153.5, 148.3, 140.6, 133.4, 132.1, 129.3, 128.6, 127.4, 118.0, 105.8, 51.6. Anal. Calcd for $C_{14}H_9ClO_3S_2$: C, 51.77; H, 2.79; Found: C, 51.71; H, 2.68.

2.7. Methyl 5-(4-bromophenyl)-3-hydroxythieno[3,2-*b*]thiophene-2-carboxylate (3g)

Beige powder (2.62 g, 71%), m.p. (227-228 °C). ^{1}H NMR (500 MHz, DMSO-d_6) δ 11.02 (s, 1H), 7.92 (s, 1H), 7.68 (d, *J* = 0.9 Hz, 4H), 3.81 (s, 3H). ^{13}C NMR (126 MHz, DMSO-d_6) δ 163.3, 153.4, 148.4, 140.7, 132.5, 132.3, 128.6, 127.7, 122.1, 118.1, 105.9, 51.7. Anal. Calcd for $C_{14}H_9BrO_3S_2$: C, 45.54; H, 2.46; Found: C, 45.63; H, 2.51.

2.8. Methyl 3-hydroxy-5-(3-methoxyphenyl)thieno[3,2-*b*]thiophene-2-carboxylate (3h)

Beige crystals (1.60 g, 50%), m.p. (154-155 °C). ^{1}H NMR (400 MHz, DMSO-d_6) δ 10.98 (s, 1H), 7.91 (s, 1H), 7.44 – 7.35 (m, 1H), 7.33 – 7.24 (m, 2H), 6.99 (ddd, *J* = 8.3, 2.4, 1.1

Hz, 1H), 3.83 (s, 6H), 3.81 (s, 3H). ^{13}C NMR (126 MHz, DMSO-*d*$_6$) δ 163.2, 159.8, 153.6, 149.7, 140.5, 134.5, 130.5, 128.4, 118.1, 117.6, 114.6, 111.1, 105.5, 55.5, 51.6. Anal. Calcd for $C_{15}H_{12}O_4S_2$: C, 56.24; H, 3.78; Found: C, 56.26; H, 3.81.

2.9. Methyl 5-(4-ethoxyphenyl)-3-hydroxythieno[3,2-*b*]thiophene-2-carboxylate (3i)

Pink crystals (1.50 g, 45%), m.p. (188-189 °C). ^{1}H NMR (400 MHz, DMSO-*d*$_6$) δ 10.89 (s, 1H), 7.73 (s, 1H), 7.65 (d, *J* = 8.8 Hz, 2H), 7.02 (d, *J* = 8.7 Hz, 2H), 4.08 (q, *J* = 6.9 Hz, 2H), 3.80 (s, 3H), 1.34 (t, *J* = 6.9 Hz, 3H). ^{13}C NMR (126 MHz, DMSO-*d*$_6$) δ 163.4, 159.2, 153.6, 150.3, 141.0, 127.3, 127.2, 125.6, 115.9, 115.2, 104.8, 63.3, 51.6, 14.6. Anal. Calcd for $C_{16}H_{14}O_4S_2$: C, 57.47; H, 4.22; Found: C, 57.29; H, 4.18.

2.10. Methyl 3-hydroxy-5-(naphthalen-2-yl)thieno[3,2-*b*]thiophene-2-carboxylate (3j)

Beige crystals (1.39 g, 41%), m.p. (205-206 °C). ^{1}H NMR (500 MHz, DMSO-*d*$_6$) δ 11.00 (s, 1H), 8.28 (d, *J* = 1.8 Hz, 1H), 8.02 (t, *J* = 4.2 Hz, 3H), 7.95 (dd, *J* = 7.4, 1.9 Hz, 1H), 7.90 (dd, *J* = 8.7, 1.9 Hz, 1H), 7.57 (tt, *J* = 6.9, 5.2 Hz, 2H), 3.82 (s, 3H). ^{13}C NMR (126 MHz, DMSO-*d*$_6$) δ 163.2, 153.7, 149.9, 140.7, 133.1, 132.8, 130.7, 128.9, 128.5, 128.2, 127.7, 127.0, 126.8, 124.4, 123.7, 117.9, 105.6, 51.6. Anal. Calcd for $C_{18}H_{12}O_3S_2$: C, 63.51; H, 3.55; Found: C, 63.44; H, 3.47.

2.11. Methyl 3-hydroxy-6-phenylthieno[3,2-*b*]thiophene-2-carboxylate (3k)

Cream crystals (1.91 g, 66%), m.p. (173-174 °C). ^{1}H NMR (400 MHz, DMSO-*d*$_6$) δ 11.09 (s, 1H), 8.30 (s, 1H), 7.80 – 7.70 (m, 2H), 7.54 (dd, *J* = 8.4, 7.0 Hz, 2H), 7.47 – 7.37 (m, 1H), 3.82 (s, 3H). ^{13}C NMR (126 MHz, DMSO-*d*$_6$) δ 163.1, 153.7, 137.3, 133.9, 133.1, 130.7, 129.3, 128.7, 128.1, 126.0, 105.5, 51.7. Anal. Calcd for $C_{14}H_{10}O_3S_2$: C, 57.91; H, 3.47; Found: C, 57.88; H, 3.54.

3. General procedure for the synthesis of 5(6)-arylthieno[3,2-*b*]thiophen-3(2*H*)-ones 4

Compound **3** (5 mmol) was placed in a pre-degassed solution of NaOH (4.00 g, 100 mmol) in DMSO/water (1:1, v./v.) (50 ml), and the resulting mixture was heated under the inert atmosphere at 120 °C for 3 hours. Then the solution was cooled, poured into water, diluted with 1 M solution of sulfuric acid (100 ml) and boiled for 30 minutes. The formed

precipitate was filtered off, washed with water and dried, thus giving the desired product **4**.

3.1. 5-Phenylthieno[3,2-*b*]thiophen-3(2*H*)-one (4a)

Light brown powder (1.11 g, 96%), m.p. (142-143°C). ^{1}H NMR (400 MHz, Chloroform-*d*) δ 7.68 – 7.61 (m, 2H), 7.49 – 7.41 (m, 3H), 7.24 (s, 1H), 4.13 (s, 2H). ^{13}C NMR (101 MHz, Chloroform-*d*) δ 190.4, 163.8, 161.6, 133.0, 130.1, 130.0, 129.3, 126.4, 118.3, 44.9. HRMS (+ESI): Calcd. for $C_{12}H_{9}OS_{2}$ m/z 233.0089 $[M+H]^{+}$, found m/z 233.0091 $[M+H]^{+}$.

3.2. 5-(*p*-Tolyl)thieno[3,2-*b*]thiophen-3(2*H*)-one (4b)

Brown powder (1.19 g, 97%), m.p. (171-172 °C). ^{1}H NMR (500 MHz, Chloroform-*d*) δ 7.56 – 7.49 (m, 2H), 7.25 (d, *J* = 7.0 Hz, 2H), 7.19 (s, 1H), 4.11 (s, 2H), 2.40 (s, 3H). ^{13}C NMR (126 MHz, Chloroform-*d*) δ 190.1, 163.6, 161.6, 140.5, 130.2, 129.9, 129.5, 126.3, 117.7, 44.8, 21.4. HRMS (+ESI): Calcd. for $C_{13}H_{11}OS_{2}$ m/z 247.0246 $[M+H]^{+}$, found m/z 247.0247 $[M+H]^{+}$.

3.3. 5-(2,5-Dimethylphenyl)-thieno[3,2-*b*]thiophen-3(2*H*)-one (4c)

Brown crystals (1.24 g, 95%), m.p. (65-66 °C).. ^{1}H NMR (400 MHz, Chloroform-*d*) δ 7.24 – 7.09 (m, 3H), 6.97 (s, 1H), 4.12 (s, 2H), 2.39 (s, 3H), 2.35 (s, 3H). ^{13}C NMR (126 MHz, Chloroform-*d*) δ 190.3, 162.7, 161.0, 135.9, 132.8, 132.6, 131.1, 130.8, 130.5, 130.4, 121.8, 44.8, 20.8, 20.4. HRMS (+ESI): Calcd. for $C_{14}H_{13}OS_{2}$ m/z 261.0402 $[M+H]^{+}$, found m/z 261.0406 $[M+H]^{+}$.

3.4. 5-(4-(*Tert*-butyl)phenyl)-thieno[3,2-*b*]thiophen-3(2*H*)-one (4d)

Dark red powder (1.41 g, 98%), m.p. (145-146 °C). ^{1}H NMR (400 MHz, Chloroform-*d*) δ 10.12 (s, 1H), 7.62 – 7.54 (m, 2H), 7.49 – 7.41 (m, 2H), 7.37 (s, 1H), 3.93 (s, 3H), 1.35 (s, 9H). ^{13}C NMR (126 MHz, Chloroform-*d*) δ 190.1, 163.6, 161.5, 153.7, 130.1, 129.6, 126.2, 126.2, 117.8, 44.8, 34.9, 31.1. HRMS (+ESI): Calcd. for $C_{16}H_{17}OS_{2}$ m/z 289.0715 $[M+H]^{+}$, found m/z 289.0719 $[M+H]^{+}$.

3.5. 5-(4-Fluorophenyl)-thieno[3,2-*b*]thiophen-3(2*H*)-one (4e)

Brown powder (1.23 g, 98%), m.p. (186-187 °C). ^{1}H NMR (500 MHz, Chloroform-*d*) δ 7.65 – 7.60 (m, 2H), 7.18 – 7.12 (m, 20H), 4.12 (s, 2H). ^{13}C NMR (126 MHz, Chloroform-*d*) δ 190.1, 163.6, 162.8, 159.9, 128.4, 128.3, 118.3, 116.5, 116.4, 44.8. ^{19}F NMR (471

MHz, Chloroform-*d*) δ 51.75 (ddd, *J* = 8.3, 5.2, 3.2 Hz). HRMS (+ESI): Calcd. for $C_{12}H_8FOS_2$ m/z 250.9995 $[M+H]^+$, found m/z 250.9992 $[M+H]^+$.

3.6. 5-(4-Chlorophenyl)-thieno[3,2-*b*]thiophen-3(2*H*)-one (4f)

Brown powder (1.24 g, 93%), m.p. (222-223 °C). ^1H NMR (500 MHz, Chloroform-*d*) δ 7.59 – 7.57 (m, 2H), 7.45 – 7.42 (m, 2H), 7.22 (s, 1H), 4.13 (s, 2H).^{13}C NMR spectrum was not recorded due to poor solubility in most deuterated solvents. Anal. Calcd for $C_{12}H_7ClOS_2$: C, 54.03; H, 2.65; Found: C, 54.09; H, 2.55.

3.7. 5-(4-Bromophenyl)-thieno[3,2-*b*]thiophen-3(2*H*)-one (4g)

Brown powder (1.49 g, 96%), m.p. (218-219 °C). ^1H NMR (500 MHz, Chloroform-*d*) δ 7.59 – 7.57 (m, 2H), 7.50 – 7.47 (m, 2H), 7.24 (s, 1H), 4.12 (s, 2H). .^{13}C NMR spectrum was not recorded due to poor solubility in most deuterated solvents. Anal. Calcd for $C_{12}H_7BrOS_2$: C, 46.31; H, 2.27; Found: C, 46.27; H, 2.20.

3.8. 5-(3-Methoxyphenyl)thieno[3,2-*b*]thiophen-3(2*H*)-one (4h)

Light brown powder (1.24 g, 95%), m.p. (127-128 °C). ^1H NMR (400 MHz, Chloroform-*d*) δ 7.37 (td, *J* = 8.1, 6.7 Hz, 1H), 7.25 – 7.19 (m, 2H), 7.14 (t, *J* = 2.2 Hz, 1H), 6.97 (ddd, *J* = 8.3, 2.6, 0.9 Hz, 1H), 4.12 (s, 2H), 3.87 (s, 3H). ^{13}C NMR (126 MHz, Chloroform-*d*) δ 190.2, 163.4, 161.1, 160.1, 134.2, 130.3, 130.0, 118.9, 118.5, 115.5, 112.0, 55.4, 44.8.HRMS (+ESI): Calcd. for $C_{13}H_{11}O_2S_2$ m/z 263.0195 $[M+H]^+$, found m/z 263.0198 $[M+H]^+$.

3.9. 5-(4-Ethoxyphenyl)-thieno[3,2-*b*]thiophen-3(2*H*)-one (4i)

Light brown powder (1.30 g, 94%), m.p. (195-196 °C). ^1H NMR (500 MHz, Chloroform-*d*) δ 7.60 – 7.53 (m, 2H), 7.12 (s, 1H), 6.98 – 6.91 (m, 2H), 4.13 – 4.04 (m, 4H), 1.45 (t, *J* = 7.0 Hz, 3H).^{13}C NMR (126 MHz, Chloroform-*d*) δ 189.9, 163.8, 161.6, 160.6, 128.9, 127.8, 125.4, 117.0, 115.1, 63.7, 44.8, 14.7. HRMS (+ESI): Calcd. for $C_{14}H_{13}O_2S_2$ m/z 277.0351 $[M+H]^+$, found m/z 277.0354 $[M+H]^+$.

3.10. 5-(Naphthalen-2-yl)thieno[3,2-*b*]thiophen-3(2*H*)-one (4j)

Beige powder (1.40 g, 99%), m.p. (179-180 °C). ^1H NMR (400 MHz, Chloroform-*d*) δ 8.15 – 8.10 (m, 1H), 7.94 – 7.82 (m, 3H), 7.71 (dd, *J* = 8.6, 1.9 Hz, 1H), 7.59 – 7.50 (m, 2H), 7.37 (s, 1H), 4.14 (s, 2H). ^{13}C NMR (126 MHz, Chloroform-*d*) δ 190.1, 163.6, 161.3, 133.9, 133.2, 130.2, 130.0, 129.1, 128.5, 127.8, 127.3, 127.1, 125.8, 123.7, 118.5, 44.9. HRMS (+ESI): Calcd. for $C_{16}H_{11}OS_2$ m/z 283.0246 $[M+H]^+$, found m/z 283.0250 $[M+H]^+$.

3.11. 6-Phenylthieno[3,2-*b*]thiophen-3(2*H*)-one (4k)

Dark brown powder (1.49 g, 98%), m.p. (100-101 °C). ^{1}H NMR (400 MHz, Chloroform-*d*) δ 7.96 (s, 1H), 7.63 – 7.55 (m, 2H), 7.52 – 7.42 (m, 2H), 7.44 – 7.30 (m, 1H), 4.16 (s, 2H). ^{13}C NMR (126 MHz, Chloroform-*d*) δ 190.7, 161.4, 137.5, 136.5, 133.1, 132.8, 129.2, 128.3, 126.6, 44.9. HRMS (+ESI): Calcd. for $C_{12}H_9OS_2$ m/z 233.0089 $[M+H]^+$, found m/z 233.0094 $[M+H]^+$.

4. General procedure for the synthesis of 2(3)-aryl-9*H*-thieno[2',3':4,5]thieno[3,2-*b*]indoles 6

Compound **4** (1.0 mmol), the appropriate arylhydrazine hydrochloride **5** (1.5 mmol) and sodium acetate (123 mg, 1.5 mmol) were added to glacial acetic acid (7 ml), and the resulting mixture was refluxed for 4 hours. Then the reaction mixture was cooled, diluted with methanol (5 ml), and the formed precipitate of product **6** was filtered off, washed with methanol and dried.

4.1. 2-Phenyl-9*H*-thieno[2',3':4,5]thieno[3,2-*b*]indole (6a)

Brown powder (171 mg, 56%), m.p. (296-297°C). ^{1}H NMR (500 MHz, DMSO-d_6) δ 11.90 (s, 1H), 8.01 (s, 1H), 7.78 – 7.72 (m, 3H), 7.57 – 7.51 (m, 1H), 7.47 (t, *J* = 7.7 Hz, 2H), 7.39 – 7.32 (m, 1H), 7.24 (ddd, *J* = 8.2, 7.1, 1.2 Hz, 1H), 7.14 (ddd, *J* = 8.1, 7.1, 1.0 Hz, 1H). ^{13}C NMR (126 MHz, DMSO-d_6) δ 143.4, 141.4, 140.3, 134.7, 134.0, 129.2, 127.8, 125.1, 122.5, 122.2, 122.0, 119.5, 118.11, 118.08, 117.4, 112.5. Anal. Calcd for $C_{18}H_{11}NS_2$: C, 70.79; H, 3.63; N, 4.59; Found: C, 70.81; H, 3.53; N, 4.50.

4.2. 2-(*p*-Tolyl)-9*H*-thieno[2',3':4,5]thieno[3,2-*b*]indole (6b)

Dark brown powder (175 mg, 55%), m.p. (317-318 °C). ^{1}H NMR (400 MHz, DMSO-d_6) δ 11.89 (s, 1H), 7.94 (s, 1H), 7.73 (d, *J* = 8.1 Hz, 1H), 7.64 (d, *J* = 7.8 Hz, 2H), 7.53 (d, *J* = 8.3 Hz, 1H), 7.31 – 7.19 (m, 2H), 7.13 (t, *J* = 7.5 Hz, 1H), 3.31 (s, 3H). ^{13}C NMR (126 MHz, DMSO-d_6) δ 143.7, 141.5, 140.2, 137.4, 134.8, 131.3, 129.8, 125.0, 122.4, 122.2, 121.6, 119.5, 118.1, 117.5, 117.2, 112.5, 20.8. Anal. Calcd for $C_{19}H_{13}NS_2$: C, 71.44; H, 4.10; N, 4.38; Found: C, 71.31; H, 4.12; N, 4.44.

4.3. 2-(2,5-Dimethylphenyl)-9*H*-thieno[2',3':4,5]thieno[3,2-*b*]indole (6c)

Brown powder (276 mg, 83%), m.p. (186-187 °C). ^{1}H NMR (500 MHz, DMSO-d_6) δ 11.88 (s, 1H), 7.75 (dd, *J* = 7.9, 1.0 Hz, 1H), 7.64 (s,1H), 7.57 – 7.51 (m, 1H), 7.34 – 7.31 (m, 1H), 7.27 – 7.21 (m, 2H), 7.17 – 7.09 (m, 2H), 2.44 (s, 3H), 2.33 (s, 3H). ^{13}C NMR (126

MHz, DMSO-d_6) δ 142.5, 140.8, 140.2, 135.3, 134.7, 133.4, 132.3, 131.0, 130.3, 128.8, 122.6, 122.3, 122.2, 121.1, 119.4, 118.1, 116.9, 112.5, 20.5, 20.4. Anal. Calcd for $C_{20}H_{15}NS_2$: C, 72.04; H, 4.53; N, 4.20; Found: C, 72.24; H, 4.47; N, 4.33.

4.4. 2-(4-(*Tert*-butyl)phenyl)-9*H*-thieno[2',3':4,5]thieno[3,2-*b*]indole (6d)

Brown powder (286 mg, 79%), m.p. (322-323 °C). ^1H NMR (400 MHz, DMSO-d_6) δ 11.87 (s, 1H), 7.94 (s, 1H), 7.74 (dt, *J* = 7.9, 1.0 Hz, 1H), 7.71 – 7.63 (m, 2H), 7.54 (dt, *J* = 8.2, 0.9 Hz, 1H), 7.51 – 7.44 (m, 2H), 7.28 – 7.19 (m, 1H), 7.13 (t, *J* = 7.5 Hz, 1H), 1.32 (s, 9H). ^{13}C NMR (126 MHz, DMSO-d_6) δ 150.5, 143.6, 141.4, 140.2, 134.7, 131.3, 125.9, 124.9, 122.4, 122.2, 121.7, 119.4, 118.0, 117.6, 117.2, 112.5, 34.3, 30.9. Anal. Calcd for $C_{22}H_{19}NS_2$: C, 73.09; H, 5.30; N, 3.87; Found: C, 73.05; H, 5.32; N, 3.78.

4.5. 2-(4-Fluorophenyl)-9*H*-thieno[2',3':4,5]thieno[3,2-*b*]indole (6e)

Brown powder (216 mg, 67%), m.p. (319-320 °C). ^1H NMR (400 MHz, DMSO-d_6) δ 11.90 (s, 1H), 7.98 (s, 1H), 7.83 – 7.76 (m, 2H), 7.74 (d, *J* = 7.9 Hz, 1H), 7.57 – 7.50 (m, 1H), 7.37 – 7.28 (m, 2H), 7.24 (ddd, *J* = 8.3, 7.1, 1.2 Hz, 1H), 7.13 (td, *J* = 7.5, 7.1, 1.1 Hz, 1H). ^{13}C NMR (126 MHz, DMSO-d_6) δ 162.7, 160.7, 142.2, 141.4, 140.3, 134.6, 130.7, 127.1, 122.5, 122.0, 119.5, 118.2, 118.1, 116.2, 116.0, 112.4. ^{19}F NMR (471 MHz, DMSO-d_6) δ 48.55 (ddd, *J* = 8.8, 5.7, 3.4 Hz). Anal. Calcd for $C_{18}H_{10}FNS_2$: C, 66.85; H, 3.12; N, 4.33; Found: C, 66.92; H, 3.22; N, 4.34.

4.6. 2-(4-Chlorophenyl)-9*H*-thieno[2',3':4,5]thieno[3,2-*b*]indole (6f)

Beige powder (221 mg, 65%), m.p. (337-338 °C). ^1H NMR (400 MHz, DMSO-d_6) δ 11.92 (s, 1H), 8.05 (s, 1H), 7.81 – 7.71 (m, 3H), 7.58 – 7.48 (m, 3H), 7.25 (ddd, *J* = 8.3, 7.1, 1.2 Hz, 1H), 7.14 (ddd, *J* = 8.1, 7.1, 1.0 Hz, 1H). ^{13}C NMR (126 MHz, DMSO-d_6) δ 141.9, 141.5, 140.3, 134.6, 133.0, 132.1, 129.2, 126.7, 122.6, 122.4, 122.1, 119.5, 118.8, 118.2, 117.8, 112.6. Anal. Calcd for $C_{18}H_{10}ClNS_2$: C, 63.61; H, 2.97; N, 4.12; Found: C, 63.55; H, 2.87; N, 4.18.

4.7. 2-(4-Bromophenyl)-9*H*-thieno[2',3':4,5]thieno[3,2-*b*]indole (6g)

Beige powder (300 mg, 78%), m.p. (340-341 °C). ^1H NMR (400 MHz, DMSO-d_6) δ 11.91 (s, 1H), 8.06 (s, 1H), 7.75 (d, *J* = 7.9 Hz, 1H), 7.72 – 7.64 (m, 4H), 7.54 (dd, *J* = 8.2, 1.0 Hz, 1H), 7.25 (ddd, *J* = 8.2, 7.1, 1.2 Hz, 1H), 7.14 (ddd, *J* = 8.1, 7.1, 1.1 Hz, 1H). ^{13}C NMR (126 MHz, DMSO-d_6) δ 141.9, 141.5, 140.3, 134.6, 133.3, 132.1, 127.0, 122.6, 122.4,

122.1, 120.7, 119.5, 118.8, 118.2, 117.8, 112.6. Anal. Calcd for $C_{18}H_{10}BrNS_2$: C, 56.26; H, 2.62; N, 3.64; Found: C, 56.21; H, 2.66; N, 3.60.

4.8. 2-(3-Methoxyphenyl)-9*H*-thieno[2',3':4,5]thieno[3,2-*b*]indole (6h)

Cream powder (150 mg, 45%), m.p. (230-231 °C). ^1H NMR (400 MHz, DMSO-*d*$_6$) δ 11.89 (s, 1H), 8.04 (s, 1H), 7.74 (d, *J* = 7.9 Hz, 1H), 7.58 – 7.50 (m, 1H), 7.39 (dd, *J* = 8.7, 7.2 Hz, 1H), 7.31 (dd, *J* = 7.4, 1.5 Hz, 2H), 7.29 – 7.20 (m, 1H), 7.14 (td, *J* = 7.5, 1.0 Hz, 1H), 6.97 – 6.89 (m, 1H), 3.85 (s, 3H). ^{13}C NMR (126 MHz, DMSO-*d*$_6$) δ 159.8, 143.2, 141.3, 140.3, 135.4, 134.7, 130.3, 122.5, 122.2, 122.1, 119.5, 118.4, 118.1, 117.55, 117.48, 113.5, 112.5, 110.5, 55.2. Anal. Calcd for $C_{19}H_{13}NOS_2$: C, 68.03; H, 3.91; N, 4.18; Found: C, 68.11; H, 3.95; N, 4.12.

4.9. 2-(4-Ethoxyphenyl)-9*H*-thieno[2',3':4,5]thieno[3,2-*b*]indole (6i)

Brown powder (209 mg, 60%), m.p. (337-338 °C). ^1H NMR (400 MHz, DMSO-*d*$_6$) δ 11.86 (s, 1H), 7.84 (s, 1H), 7.72 (dd, *J* = 7.8, 1.1 Hz, 1H), 7.70 – 7.61 (m, 2H), 7.53 (dt, *J* = 8.2, 0.9 Hz, 1H), 7.27 – 7.18 (m, 1H), 7.17 – 7.07 (m, 1H), 7.06 – 6.97 (m, 2H), 4.08 (q, *J* = 7.0 Hz, 2H), 1.35 (t, *J* = 7.0 Hz, 3H). ^{13}C NMR (126 MHz, DMSO-*d*$_6$) δ 158.3, 143.7, 141.5, 140.2, 134.8, 126.6, 126.5, 122.3, 122.2, 121.1, 119.4, 117.9, 116.8, 116.7, 115.1, 112.4, 63.2, 14.5. Anal. Calcd for $C_{20}H_{15}NOS_2$: C, 68.74; H, 4.33; N, 4.01; Found: C, 68.79; H, 4.39; N, 4.02.

4.10. 2-(Naphthalen-2-yl)-9*H*-thieno[2',3':4,5]thieno[3,2-*b*]indole (6j)

Beige powder (302 mg, 85%), m.p. (342-343 °C). ^1H NMR (400 MHz, DMSO-*d*$_6$) δ 11.95 (s, 1H), 8.29 – 8.23 (m, 1H), 8.16 (s, 1H), 8.01 (d, *J* = 8.5 Hz, 2H), 7.94 (dt, *J* = 8.1, 1.9 Hz, 1H), 7.80 – 7.73 (m, 1H), 7.61 – 7.48 (m, 3H), 7.26 (ddd, *J* = 8.3, 7.1, 1.2 Hz, 1H), 7.15 (ddd, *J* = 8.1, 7.1, 1.1 Hz, 1H). ^{13}C NMR (126 MHz, DMSO-*d*$_6$) δ 143.5, 141.5, 140.3, 134.7, 133.2, 132.3, 131.6, 128.7, 127.9, 127.6, 126.8, 126.2, 123.6, 123.1, 122.5, 122.3, 122.2, 119.5, 118.7, 118.1, 117.7, 112.5. Anal. Calcd for $C_{22}H_{13}NS_2$: C, 74.34; H, 3.69; N, 3.94; Found: C, 74.31; H, 3.75; N, 3.99.

4.11. 3-Phenyl-9*H*-thieno[2',3':4,5]thieno[3,2-*b*]indole (6k)

Dark grey powder (214 mg, 70%), m.p. (185-186°C). ^1H NMR (500 MHz, DMSO-*d*$_6$) δ 11.93 (s, 1H), 8.00 (s, 1H), 7.89 – 7.83 (m, 2H), 7.77 (dd, *J* = 7.9, 1.1 Hz, 1H), 7.60 – 7.53 (m, 3H), 7.47 – 7.40 (m, 1H), 7.29 – 7.22 (m, 1H), 7.19 – 7.12 (m, 1H). ^{13}C NMR (126

MHz, DMSO-*d*6) δ 140.1, 138.5, 134.85, 134.77, 133.9, 129.2, 127.9, 126.2, 123.9, 122.6, 122.5, 122.1, 119.5, 118.2, 117.0, 112.6. Anal. Calcd for $C_{18}H_{11}NS_2$: C, 70.79; H, 3.63; N, 4.59; Found: C, 70.81; H, 3.67; N, 4.55.

4.12. 2-(4-(*Tert*-butyl)phenyl)-6-methyl-9*H*-thieno[2',3':4,5]thieno[3,2-*b*]indole (6l)

Red brown powder (315 mg, 84%), m.p. (333-334 °C). ^{1}H NMR (400 MHz, DMSO-*d*6) δ 11.72 (s, 1H), 7.92 (s, 1H), 7.70 – 7.62 (m, 2H), 7.54 – 7.45 (m, 3H), 7.42 (d, *J* = 8.3 Hz, 1H), 7.10 – 7.02 (m, 1H), 2.43 (s, 3H), 1.32 (s, 9H). ^{13}C NMR (151 MHz, Chloroform-*d*) δ 151.0, 144.4, 142.0, 138.8, 134.6, 132.0, 129.8, 125.9, 125.5, 124.3, 123.5, 122.3, 119.3, 118.3, 116.8, 111.7, 34.6, 31.2, 21.4. Anal. Calcd for $C_{23}H_{21}NS_2$: C, 70.79; H, 3.63; N, 4.59; Found: C, 70.81; H, 3.66; N, 4.65.

4.13. 9-(4-(*Tert*-butyl)phenyl)-11*H*-benzo[*g*]thieno[2',3':4,5]thieno[3,2-*b*]indole (6m)

Grey powder (321 mg, 78%), m.p. (290-291 °C). ^{1}H NMR (400 MHz, DMSO-*d*6) δ 12.86 (s, 1H), 8.40 (d, *J* = 8.1 Hz, 1H), 8.04 – 7.98 (m, 4H), 7.96 (s, 1H), 7.88 (d, *J* = 8.6 Hz, 2H), 7.72 – 7.57 (m, 4H), 7.54 – 7.44 (m, 3H), 1.32 (s, 9H). ^{13}C NMR (126 MHz, DMSO-*d*6) δ 150.4, 143.0, 140.8, 134.7, 133.3, 131.4, 130.0, 128.6, 125.9, 125.8, 124.9, 124.2, 122.3, 121.9, 120.6, 120.2, 119.1, 118.3, 117.8, 117.6, 34.3, 30.9. Anal. Calcd for $C_{26}H_{21}NS_2$: C, 75.87; H, 5.14; N, 3.40; Found: C, 75.81; H, 5.17; N, 3.38.

4.14. 6-(*Tert*-butyl)-2-(4-(*tert*-butyl)phenyl)-9*H*-thieno[2',3':4,5]thieno[3,2-*b*]indole (6n)

Beige powder (342 mg, 82%), m.p. (344-345 °C). ^{1}H NMR (400 MHz, DMSO-*d*6) δ 11.72 (s, 1H), 7.93 (s, 1H), 7.72 – 7.62 (m, 3H), 7.47 (dd, *J* = 12.4, 8.6 Hz, 3H), 7.32 (dd, *J* = 8.6, 1.9 Hz, 1H), 1.37 (s, 9H), 1.32 (s, 9H). ^{13}C NMR (126 MHz, Chloroform-*d*) δ 151.0, 144.2, 143.5, 141.9, 138.5, 134.6, 131.9, 129.7, 126.0, 125.4, 123.1, 122.3, 121.0, 116.8, 114.6, 111.5, 34.74, 34.67, 31.9, 31.3. Anal. Calcd for $C_{26}H_{27}NS_2$: C, 74.78; H, 6.52; N, 3.35; Found: C, 74.79; H, 6.45; N, 3.30.

4.15. 2-(4-(*Tert*-butyl)phenyl)-9*H*-thieno[2',3':4,5]thieno[3,2-*b*]indole-6-carbonitrile (6o)

Beige powder (309 mg, 80%), m.p. (343-344 °C). ^{1}H NMR (400 MHz, DMSO-*d*6) δ 12.49 (s, 1H), 8.39 (d, *J* = 1.5 Hz, 1H), 7.98 (s, 1H), 7.74 – 7.64 (m, 3H), 7.58 (dd, *J* = 8.5, 1.6 Hz, 1H), 7.56 – 7.45 (m, 2H), 1.32 (s, 9H). ^{13}C NMR (151 MHz, DMSO-*d*6) δ 172.5, 151.3,

145.5, 143.7, 142.5, 137.0, 131.6, 126.6, 125.6, 124.0, 122.6, 121.7, 120.9, 118.2, 117.9, 114.1, 101.8, 34.9, 31.5. Anal. Calcd for $C_{23}H_{18}N_2S_2$: C, 71.47; H, 4.69; N, 7.25; Found: C, 71.47; H, 4.69; N, 7.25.

5. Procedure for the synthesis of 9-benzyl-2-(4-(*tert*-butyl)phenyl)-9*H*-thieno[2',3':4,5]thieno[3,2-*b*]indole (7d)

Compound **6d** (180 mg, 0.5 mmol) was dissolved in DMSO (10 ml). The solution was cooled to 10 °C (cold water bath), NaH (24 mg, 1.0 mmol) was added, and the resulting suspension was stirred for an hour. After the addition of benzyl bromide (119 µl, 1.0 mmol) the reaction mixture was stirred for another hour, then diluted with water (20 ml), the formed precipitate was filtered off, dried and recrystallized from ethyl acetate to afford the desired product **7d**.

9-Benzyl-2-(4-(*tert*-butyl)phenyl)-9*H*-thieno[2',3':4,5]thieno[3,2-*b*]indole (7d)

Light yellow crystals (214 mg, 95%), m.p. (249-250 °C). ^{1}H NMR (500 MHz, Chloroform-*d*) δ 7.74 (dd, *J* = 7.5, 1.2 Hz, 1H), 7.57 – 7.50 (m, 3H), 7.45 – 7.37 (m, 3H), 7.34 – 7.22 (m, 6H), 7.21 (td, *J* = 7.5, 1.0 Hz, 1H), 5.61 (s, 2H), 1.35 (s, 9H). ^{13}C NMR (126 MHz, DMSO-d_6) δ 150.6, 143.9, 141.7, 140.4, 137.5, 136.1, 131.0, 128.7, 127.6, 126.8, 126.0, 124.9, 122.7, 122.0, 120.9, 119.9, 118.4, 117.5, 116.8, 111.0, 48.1, 34.3, 30.91. Anal. Calcd for $C_{29}H_{25}NS_2$: C, 77.12; H, 5.58; N, 3.10; Found: C, 77.10; H, 5.61; N, 3.09.

Copies of ^{1}H, ^{13}C and ^{19}F NMR spectra of new compounds

Methyl 3-chloro-5-phenylthiophene-2-carboxylate (2a)

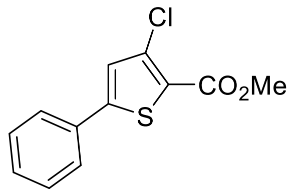

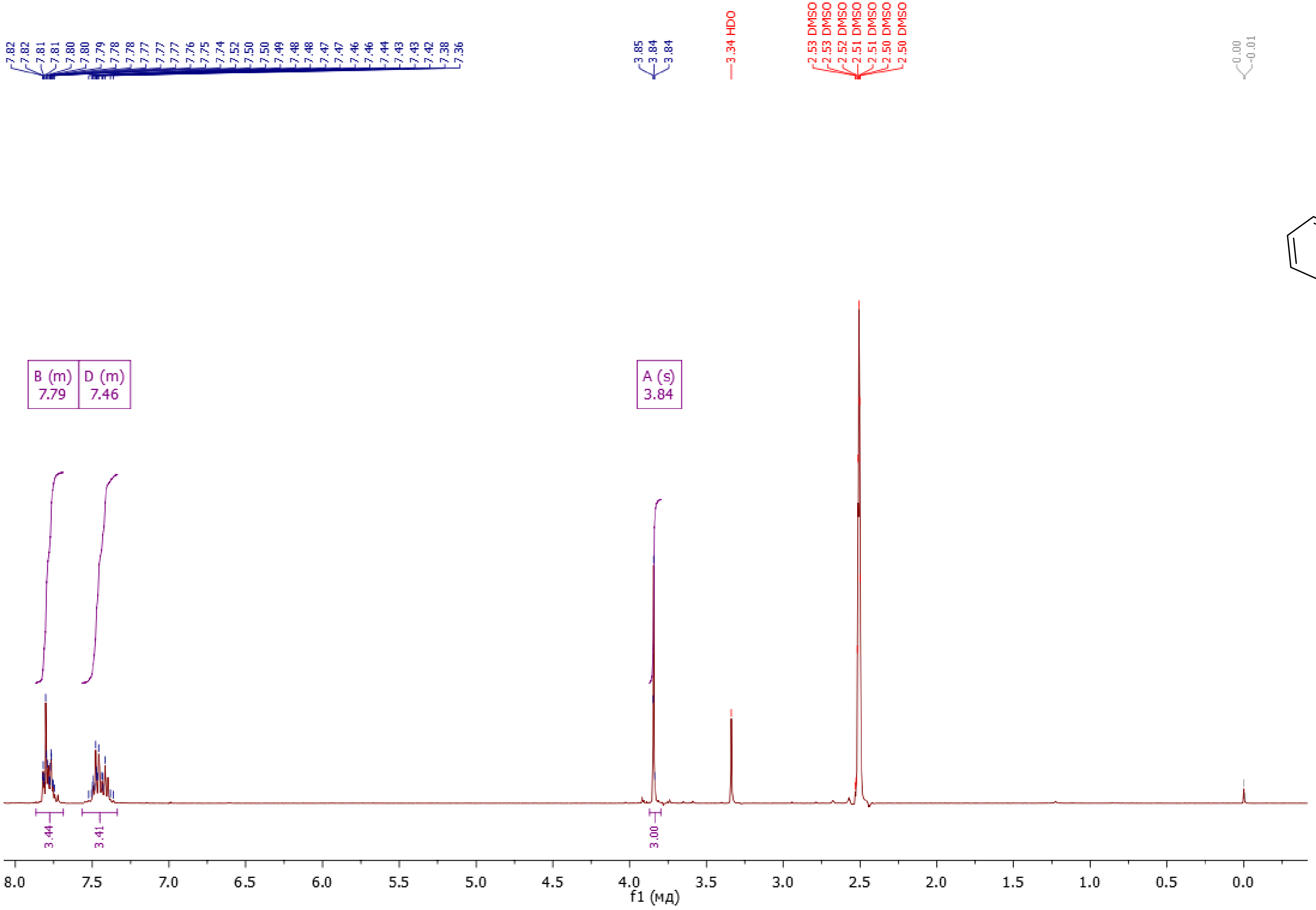

^{1}H NMR (400 MHz, DMSO-d_6) δ 7.87 – 7.69 (m, 3H), 7.56 – 7.34 (m, 3H), 3.84 (s, 3H).

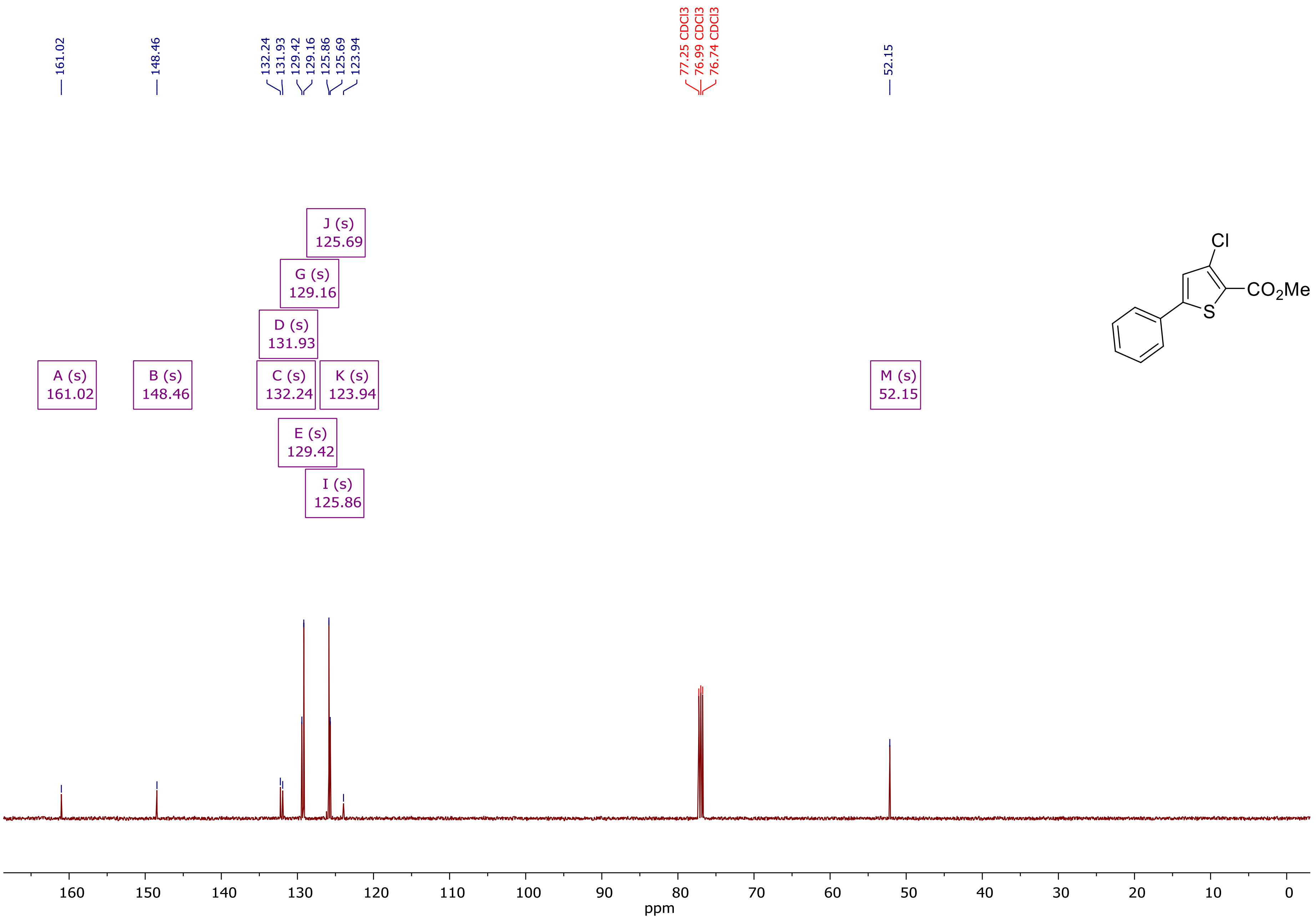

^{13}C NMR (126 MHz, chloroform-*d*) δ 161.0, 148.5, 132.2, 131.9, 129.4, 129.2, 125.9, 125.7, 123.9, 52.2.

Methyl 3-chloro-5-(*p*-tolyl)thiophene-2-carboxylate (2b)

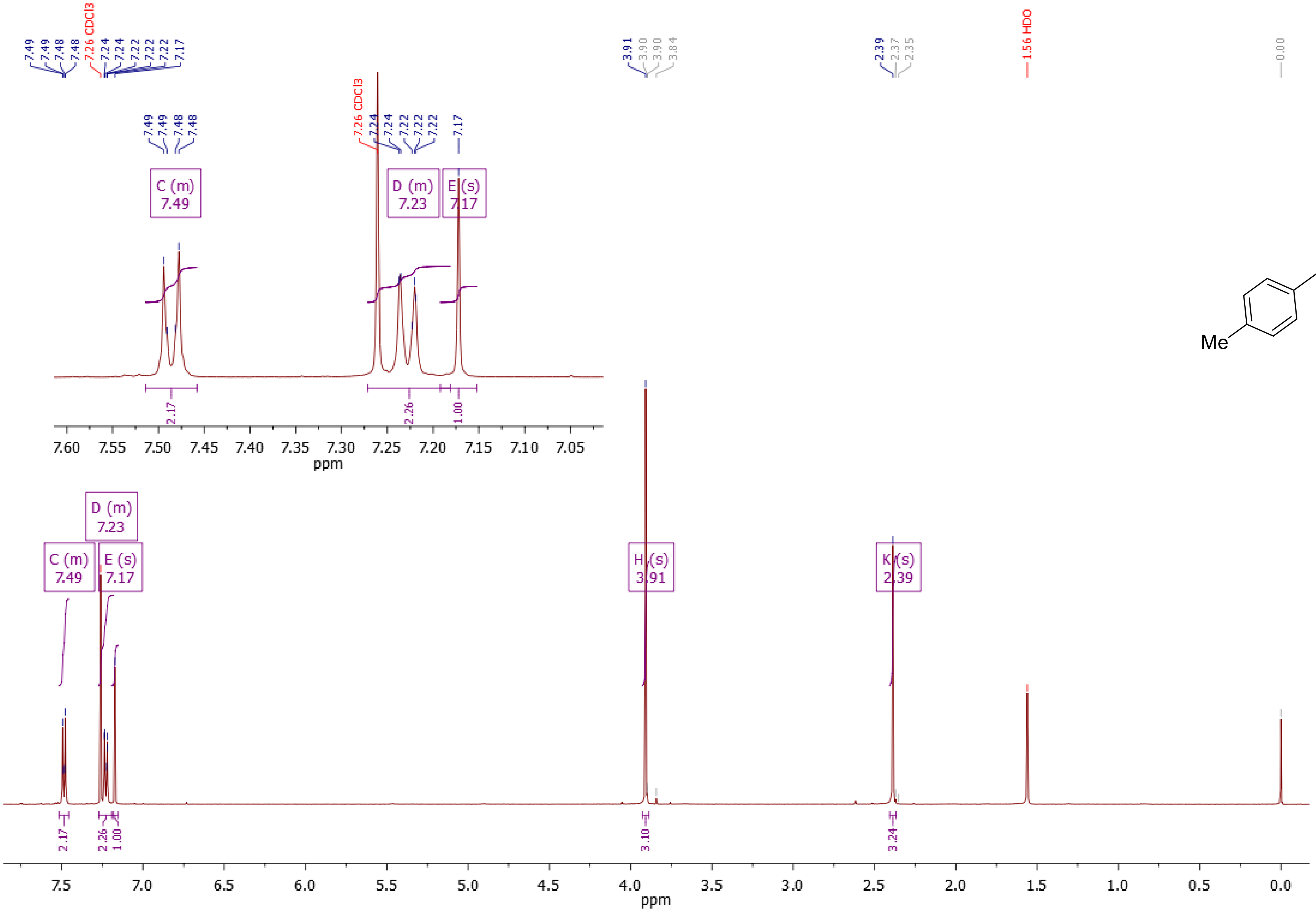

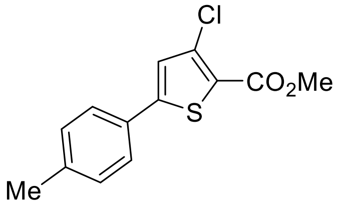

^{1}H NMR (500 MHz, chloroform-*d*) δ 7.51 – 7.46 (m, 2H), 7.27 – 7.18 (m, 2H), 7.17 (s, 1H), 3.91 (s, 3H), 2.39 (s, 3H).

Methyl 5-(2,5-dimethylphenyl)-3-chlorothiophene-2-carboxylate (2c)

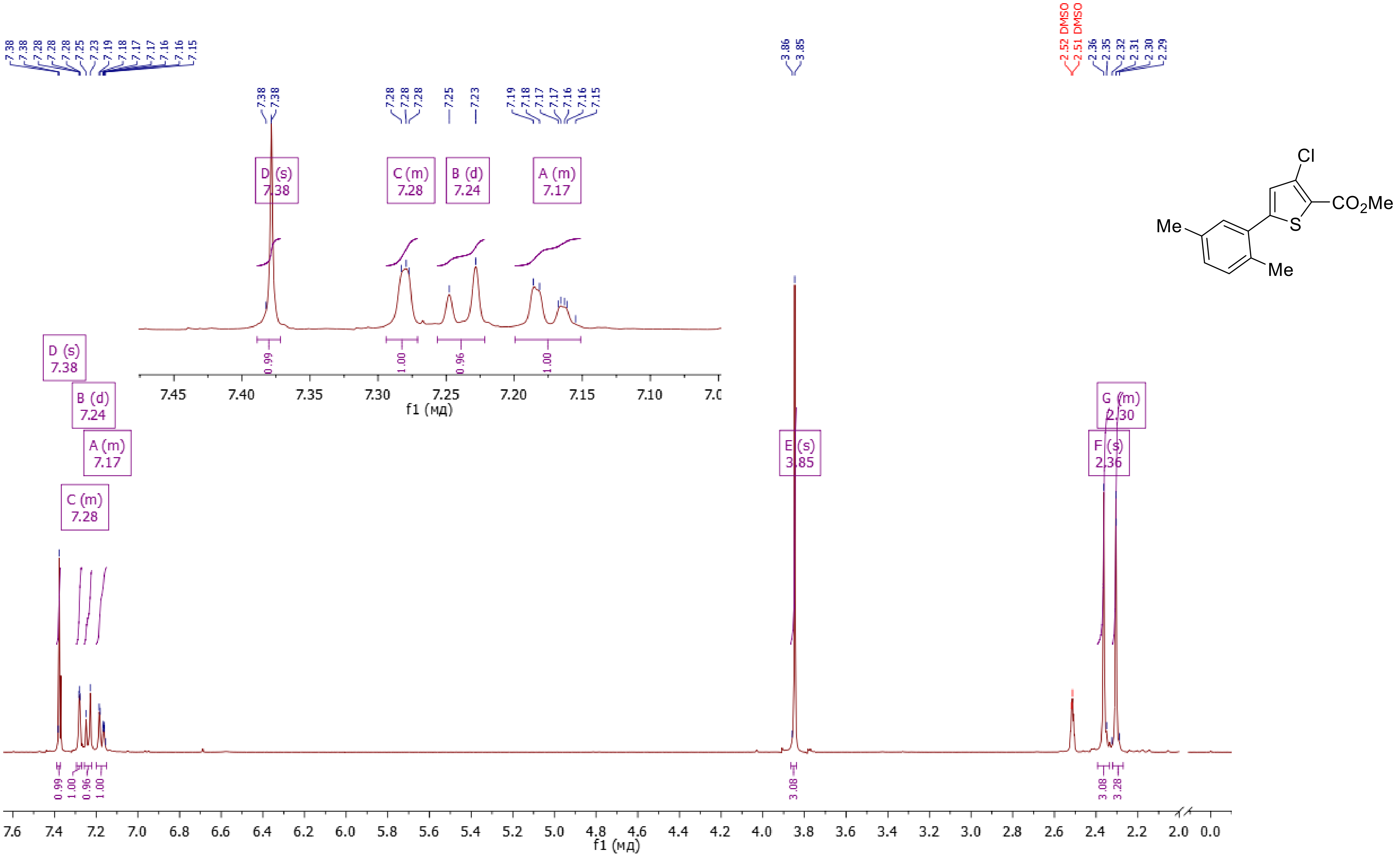

^{1}H NMR (400 MHz, DMSO-d_6) δ 7.38 (s, 1H), 7.29 – 7.27 (m, 1H), 7.24 (d, J = 7.8 Hz, 1H), 7.20 – 7.15 (m, 1H), 3.85 (s, 3H), 2.36 (s, 3H), 2.32 – 2.27 (m, 3H).

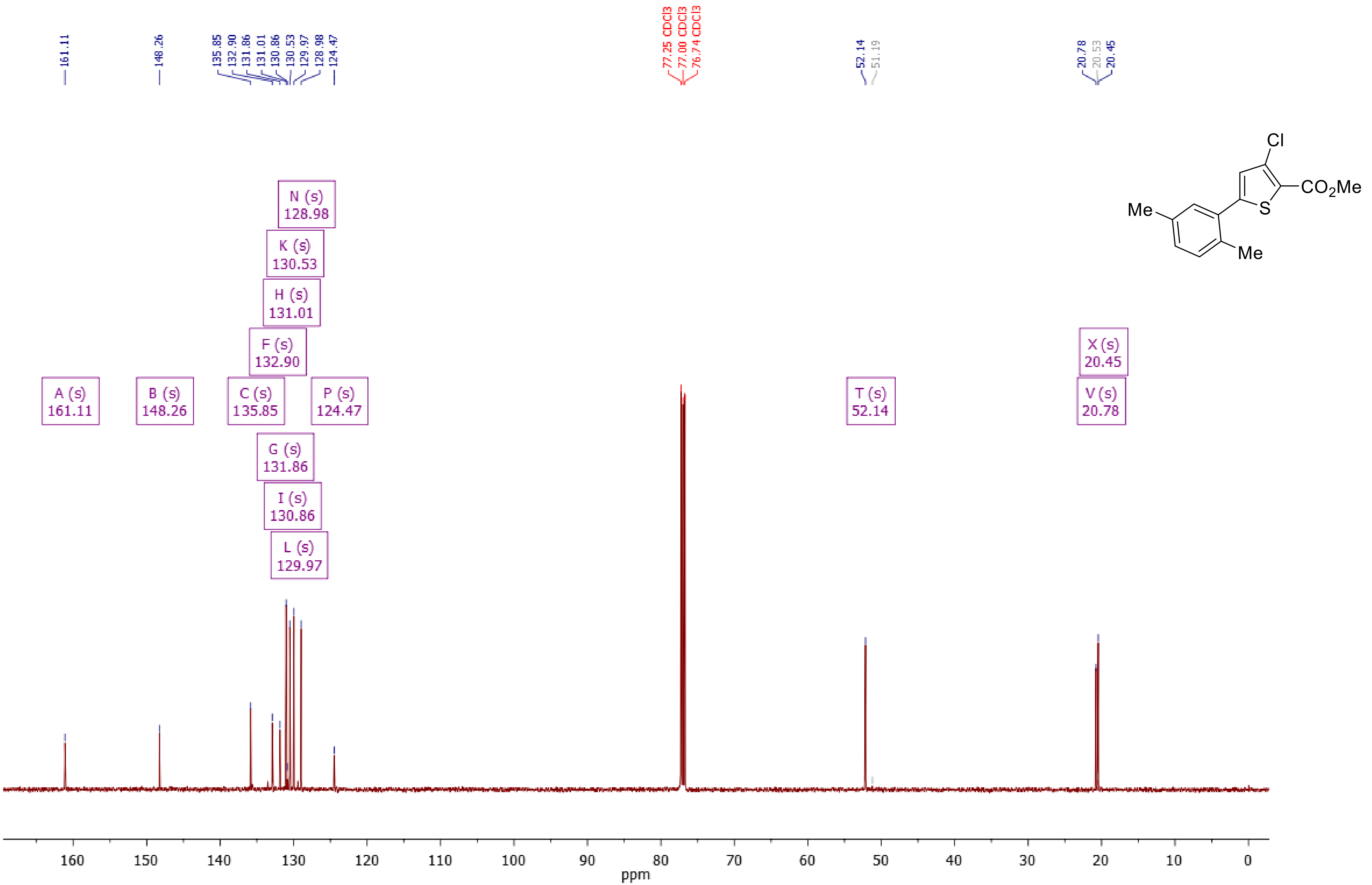

^{13}C NMR (126 MHz, chloroform-*d*) δ 161.1, 148.3, 135.8, 132.9, 131.9, 131.0, 130.9, 130.5, 130.0, 129.0, 124.5, 52.1, 20.8, 20.4.

Methyl 5-(4-(*tert*-butyl)phenyl)-3-chlorothiophene-2-carboxylate (2d)

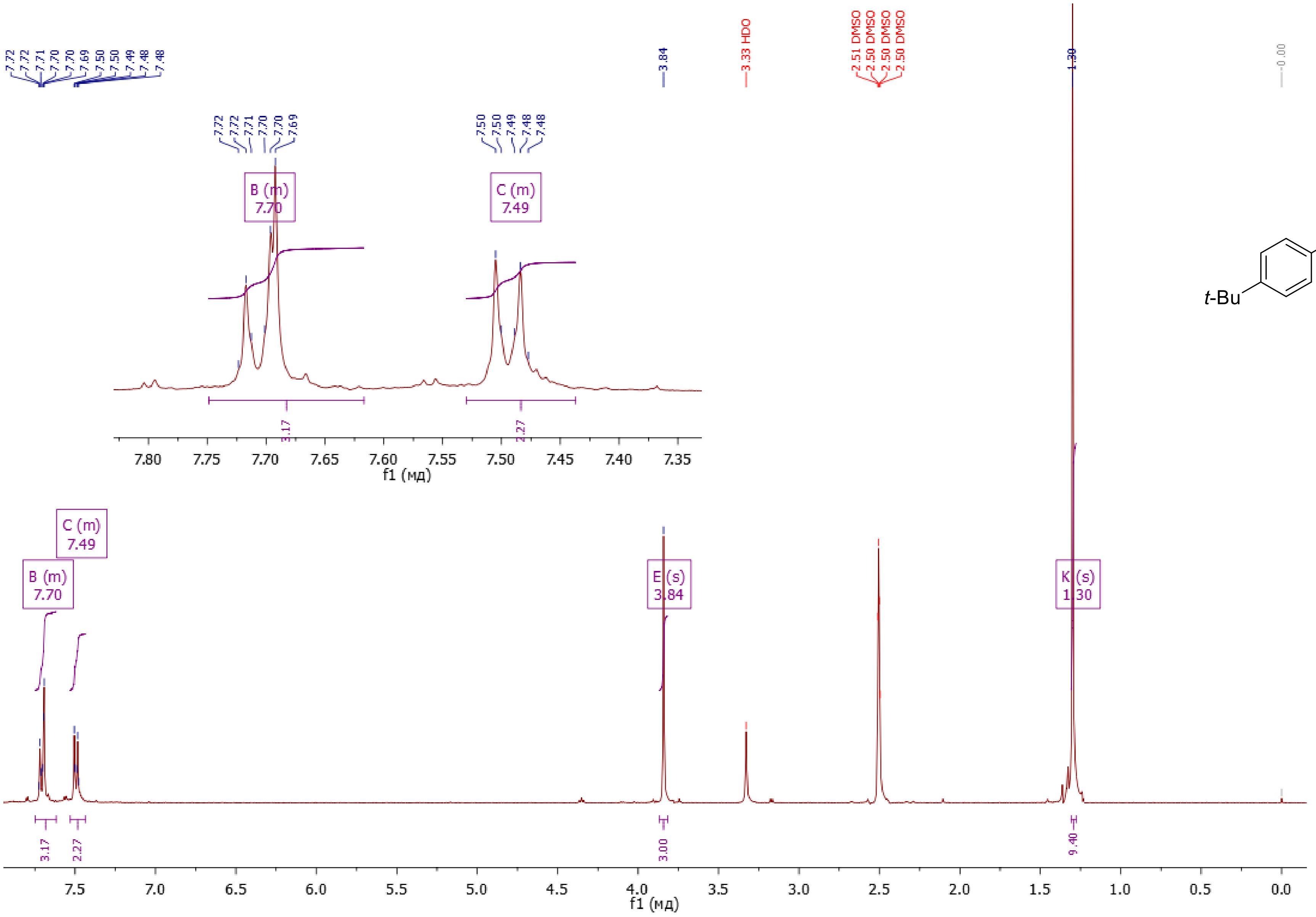

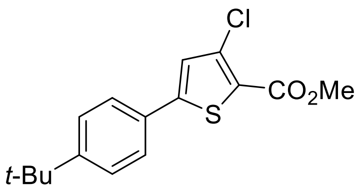

^{1}H NMR (400 MHz, DMSO-d_6) δ 7.75 – 7.62 (m, 3H), 7.53 – 7.44 (m, 2H), 3.84 (s, 3H), 1.30 (s, 9H).

Methyl 5-(4-fluorophenyl)-3-chlorothiophene-2-carboxylate (2e)

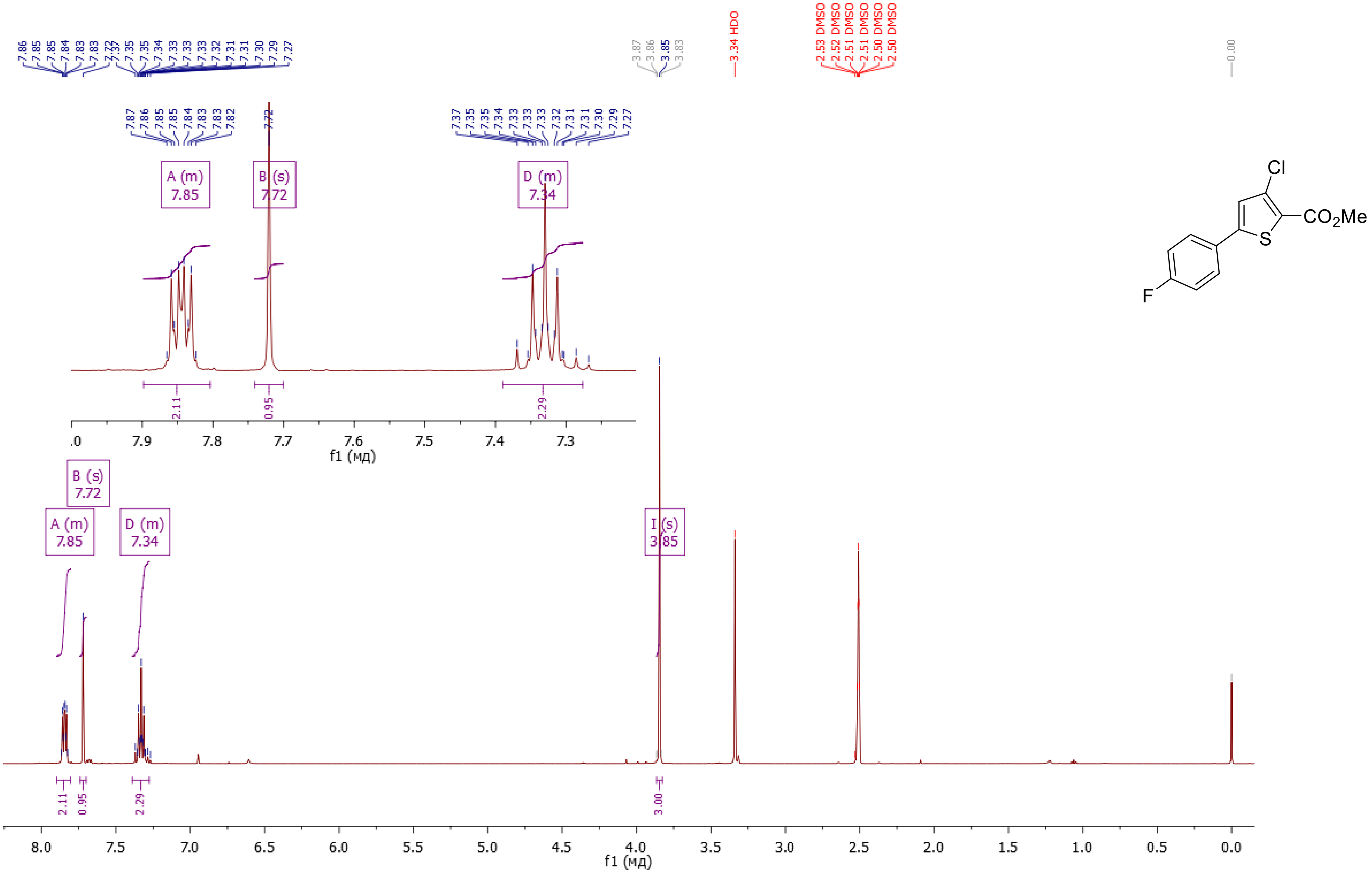

^{1}H NMR (500 MHz, DMSO-*d*6) δ 7.90 – 7.80 (m, 2H), 7.72 (s, 1H), 7.39 – 7.28 (m, 2H), 3.85 (s, 3H).

Methyl 5-(4-chlorophenyl)-3-chlorothiophene-2-carboxylate (2f)

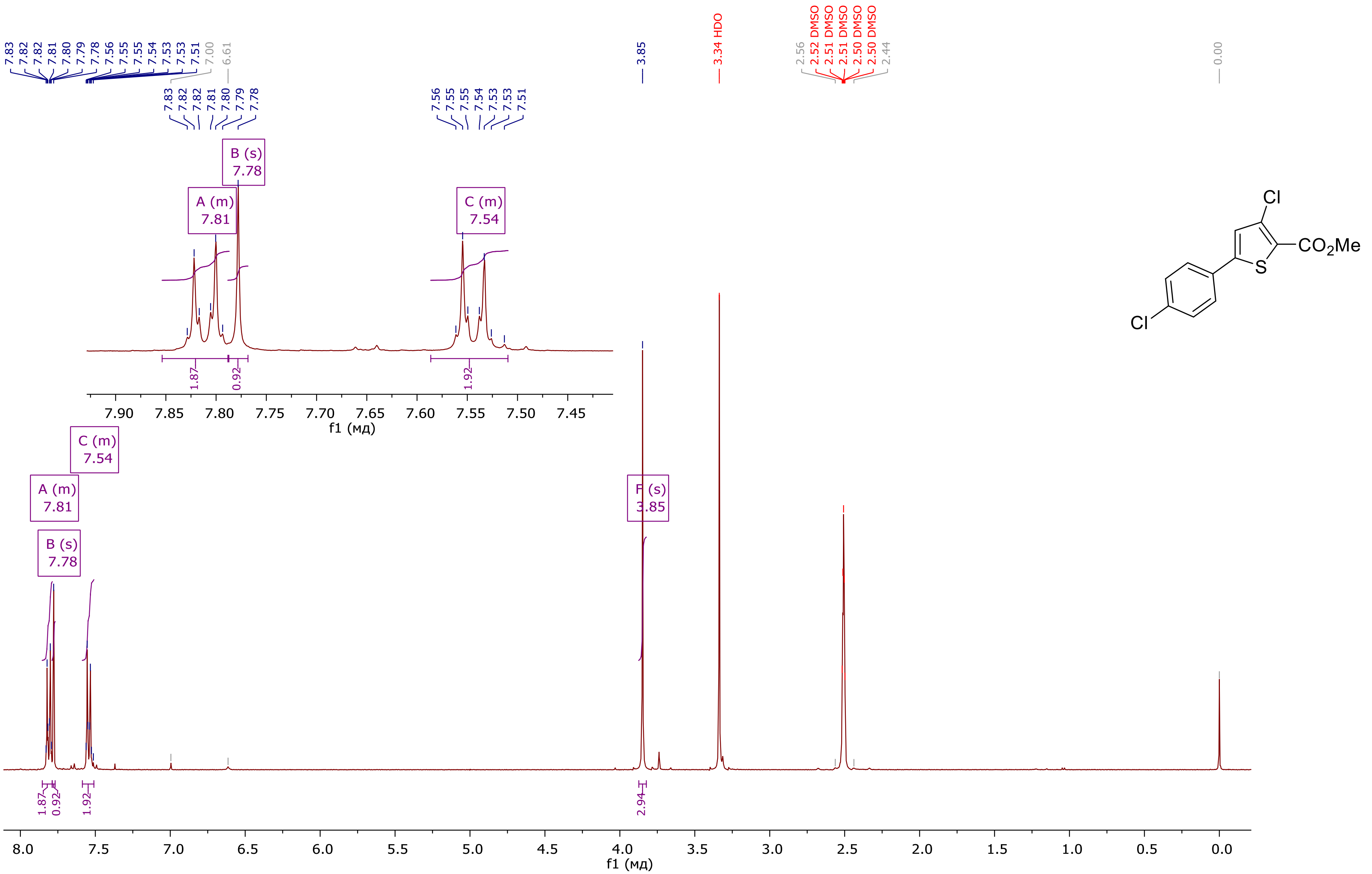

^{1}H NMR (400 MHz, DMSO-d_6) δ 7.85 – 7.79 (m, 2H), 7.78 (s, 1H), 7.59 – 7.51 (m, 2H), 3.85 (s, 3H).

Methyl 5-(4-bromophenyl)-3-chlorothiophene-2-carboxylate (2g)

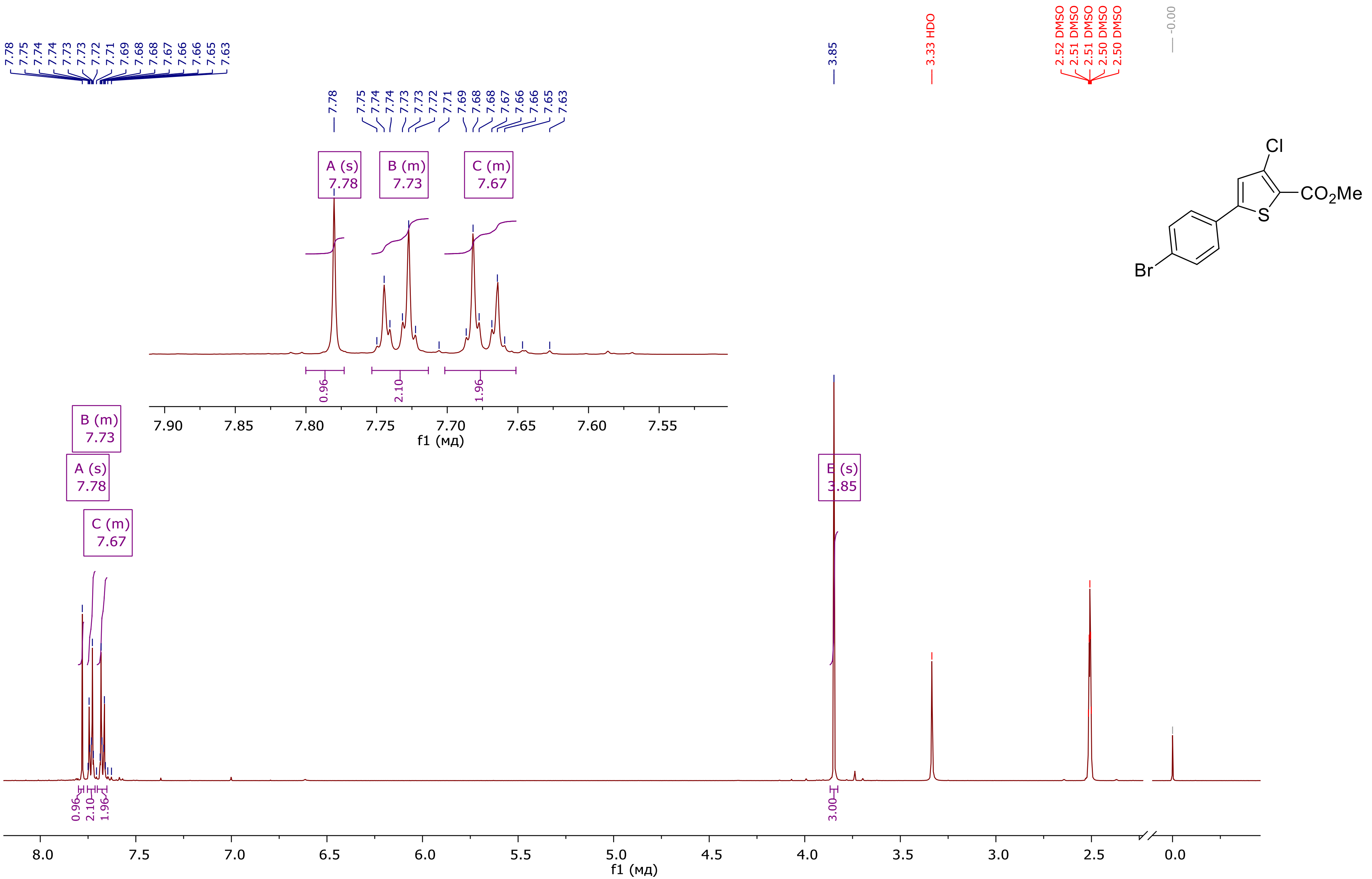

^{1}H NMR (500 MHz, DMSO-d_6) δ 7.78 (s, 1H), 7.75 – 7.71 (m, 2H), 7.70 – 7.65 (m, 2H), 3.85 (s, 3H).

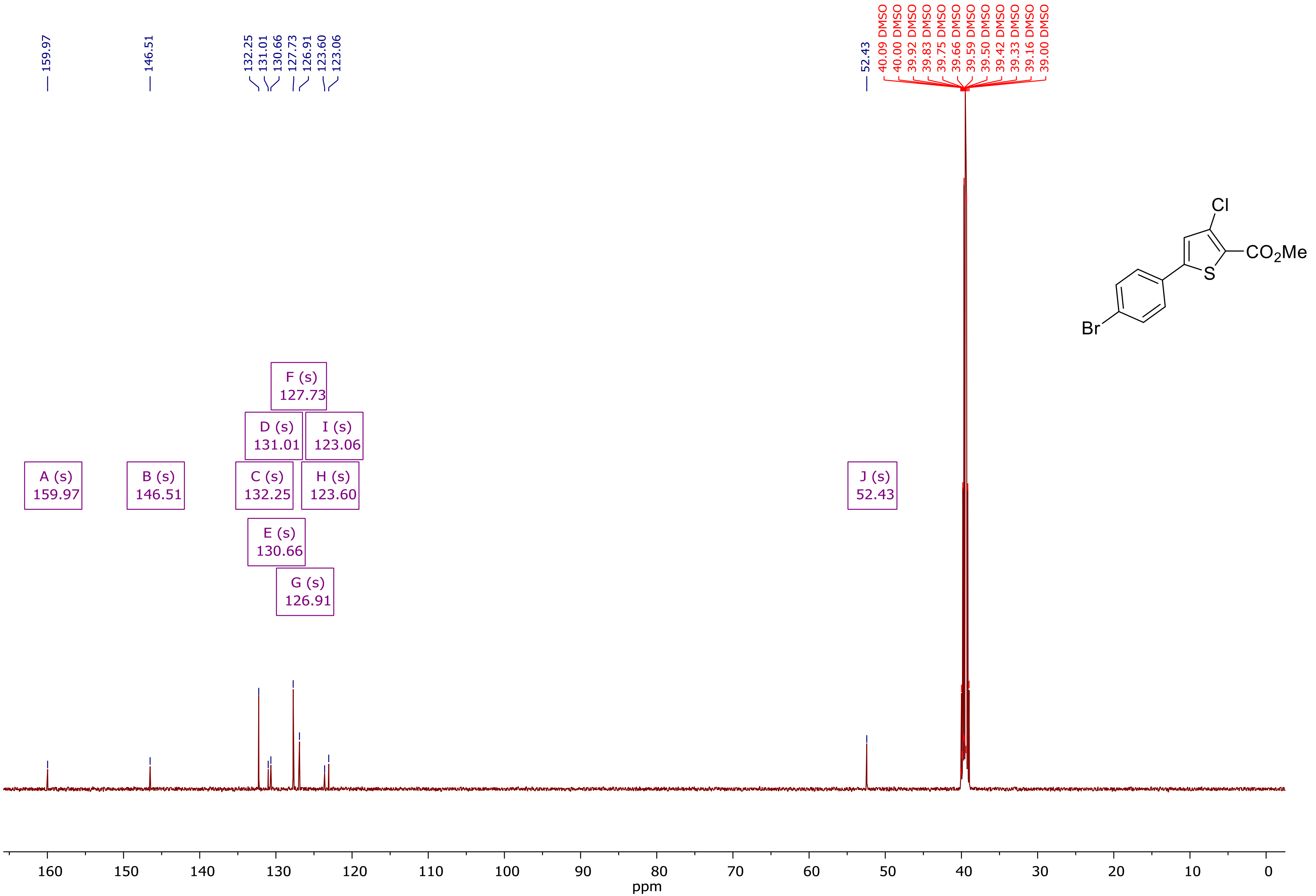

^{13}C NMR (126 MHz, DMSO-d_6) δ 160.0, 146.5, 132.2, 131.0, 130.7, 127.7, 126.9, 123.6, 123.1, 52.4.

Methyl 3-chloro-5-(3-methoxyphenyl)thiophene-2-carboxylate (2h)

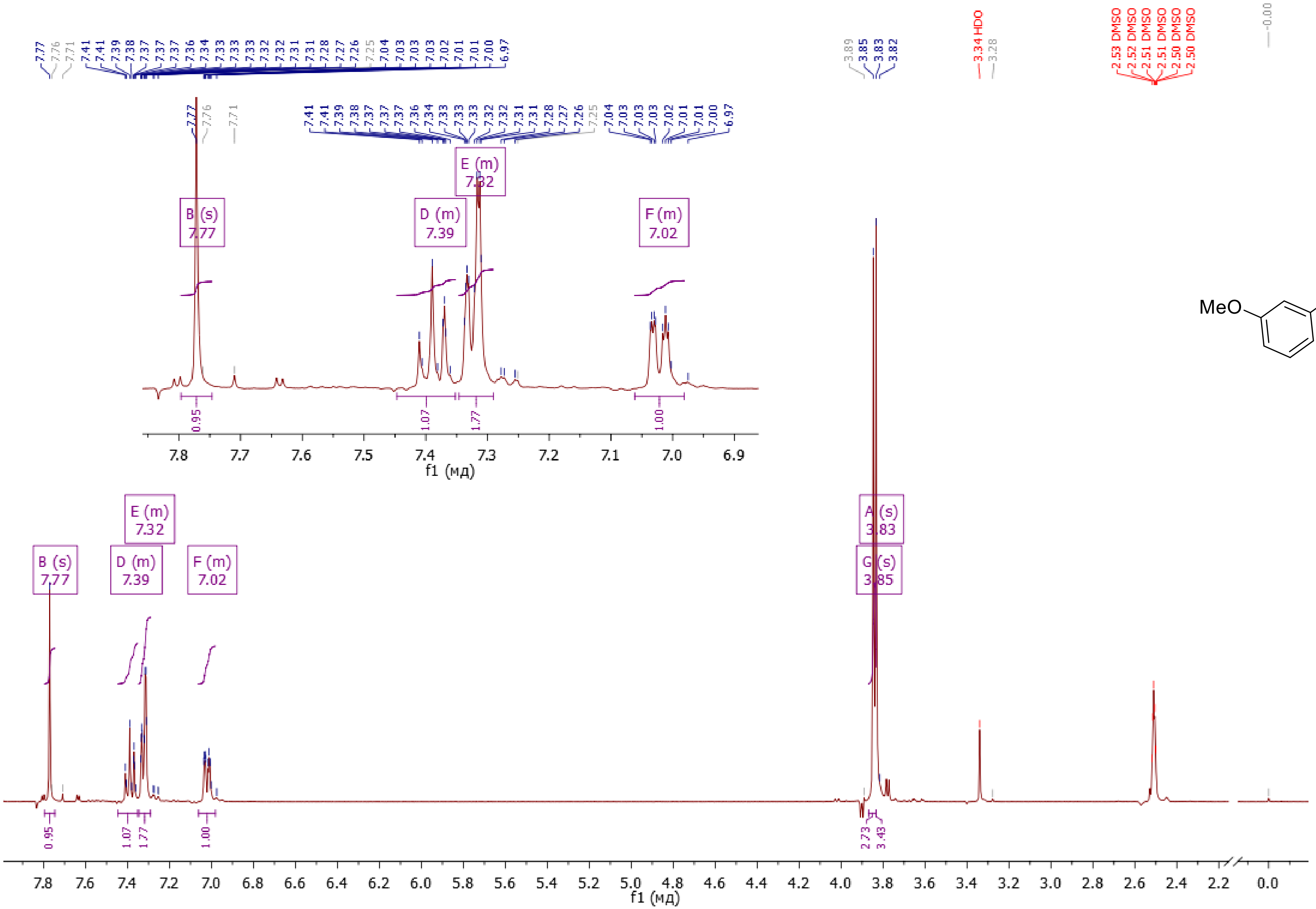

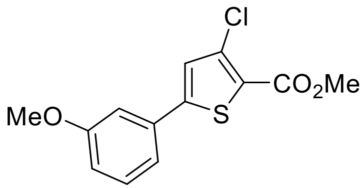

^{1}H NMR (400 MHz, DMSO-d_6) δ 7.77 (s, 1H), 7.45 – 7.35 (m, 1H), 7.35 – 7.29 (m, 2H), 7.06 – 6.98 (m, 1H), 3.85 (s, 3H), 3.83 (s, 3H).

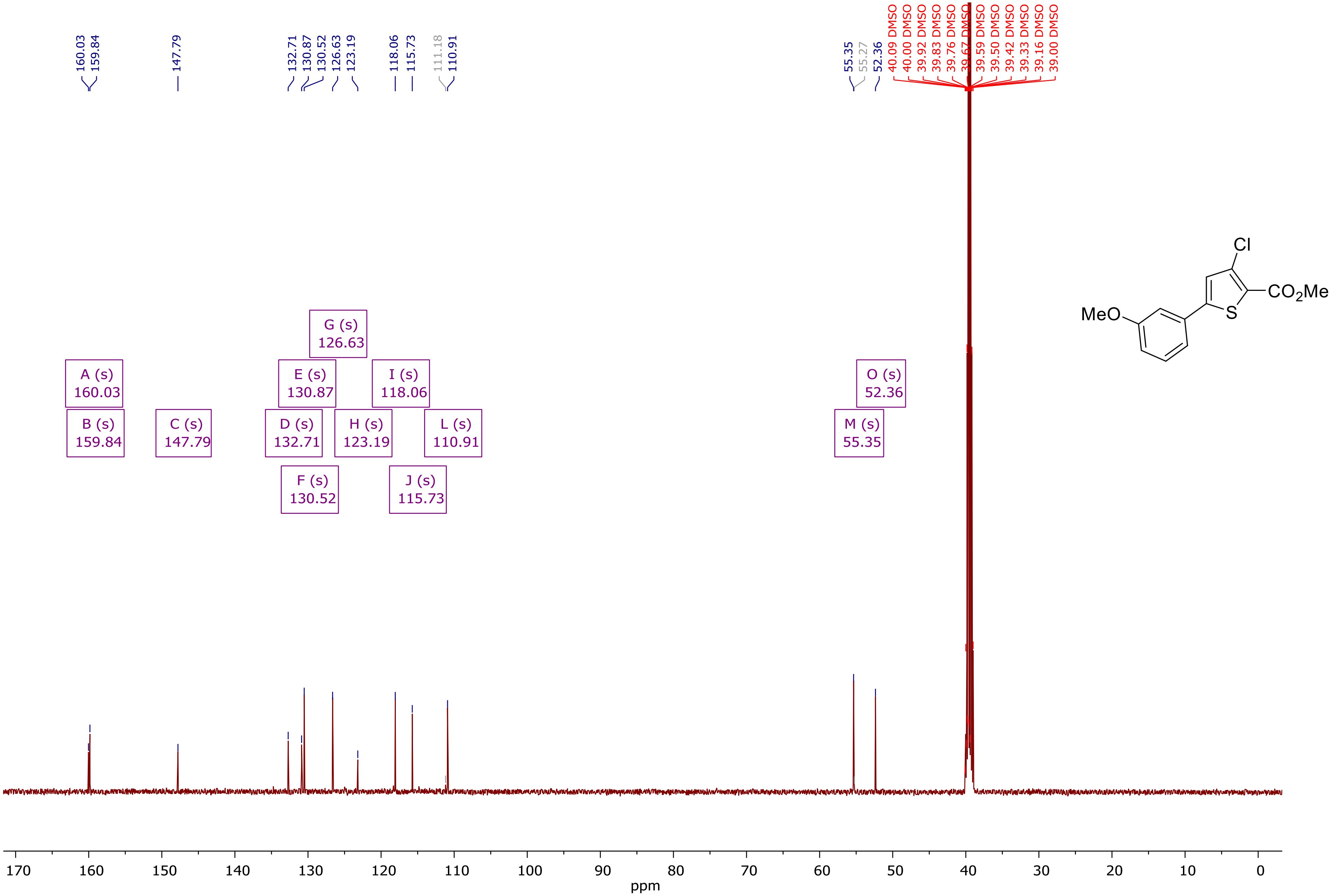

^{13}C NMR (126 MHz, DMSO-d_6) δ 160.0, 159.8, 147.8, 132.7, 130.9, 130.5, 126.6, 123.2, 118.1, 115.7, 110.9, 55.3, 52.4.

Methyl 5-(4-ethoxyphenyl)-3-chlorothiophene-2-carboxylate (2i)

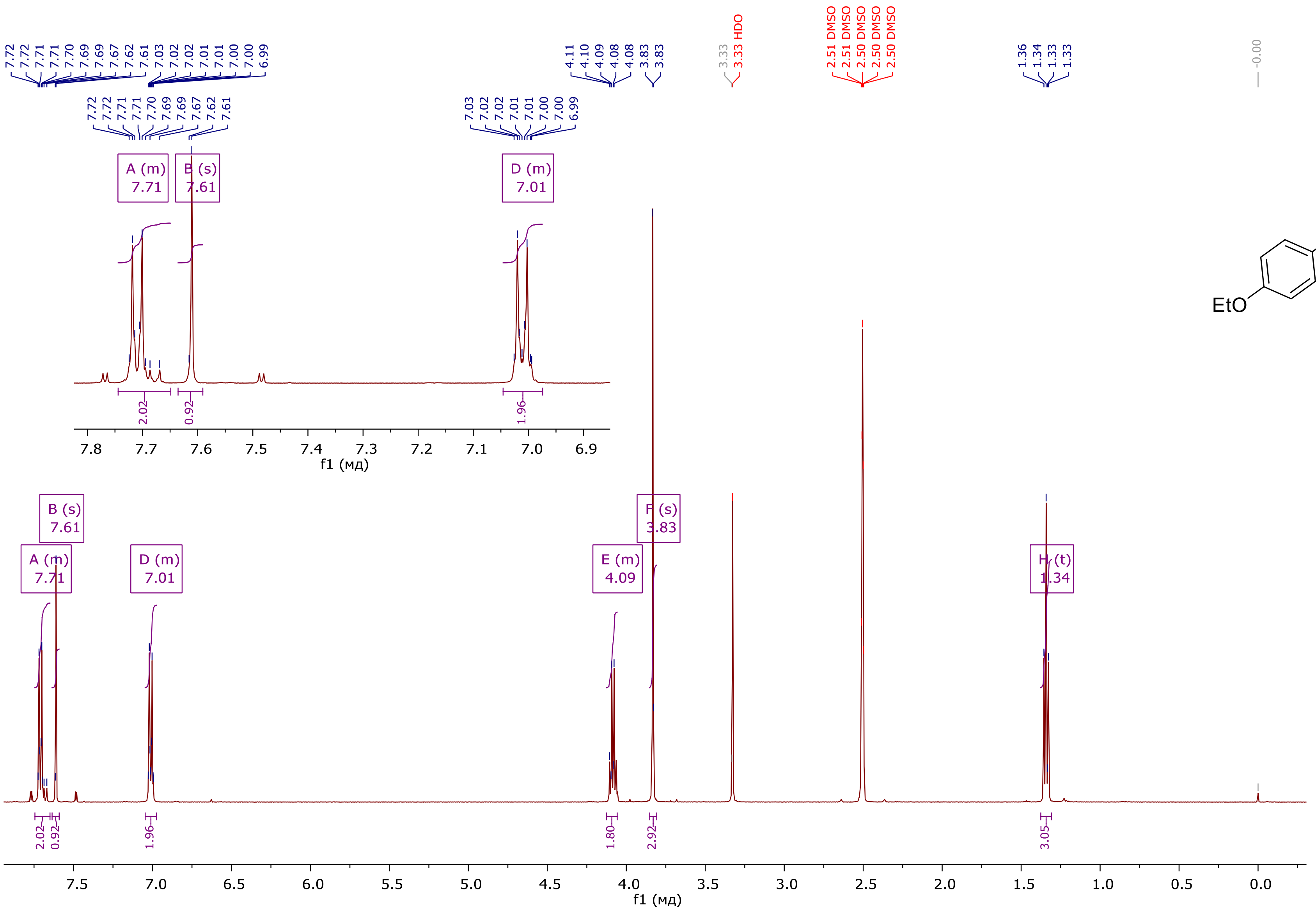

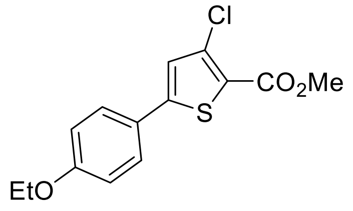

^1H NMR (500 MHz, DMSO-d_6) δ 7.74 – 7.65 (m, 2H), 7.61 (s, 1H), 7.05 – 6.97 (m, 2H), 4.13 – 4.06 (m, 2H), 3.83 (s, 3H), 1.34 (t, *J* = 6.9 Hz, 3H).

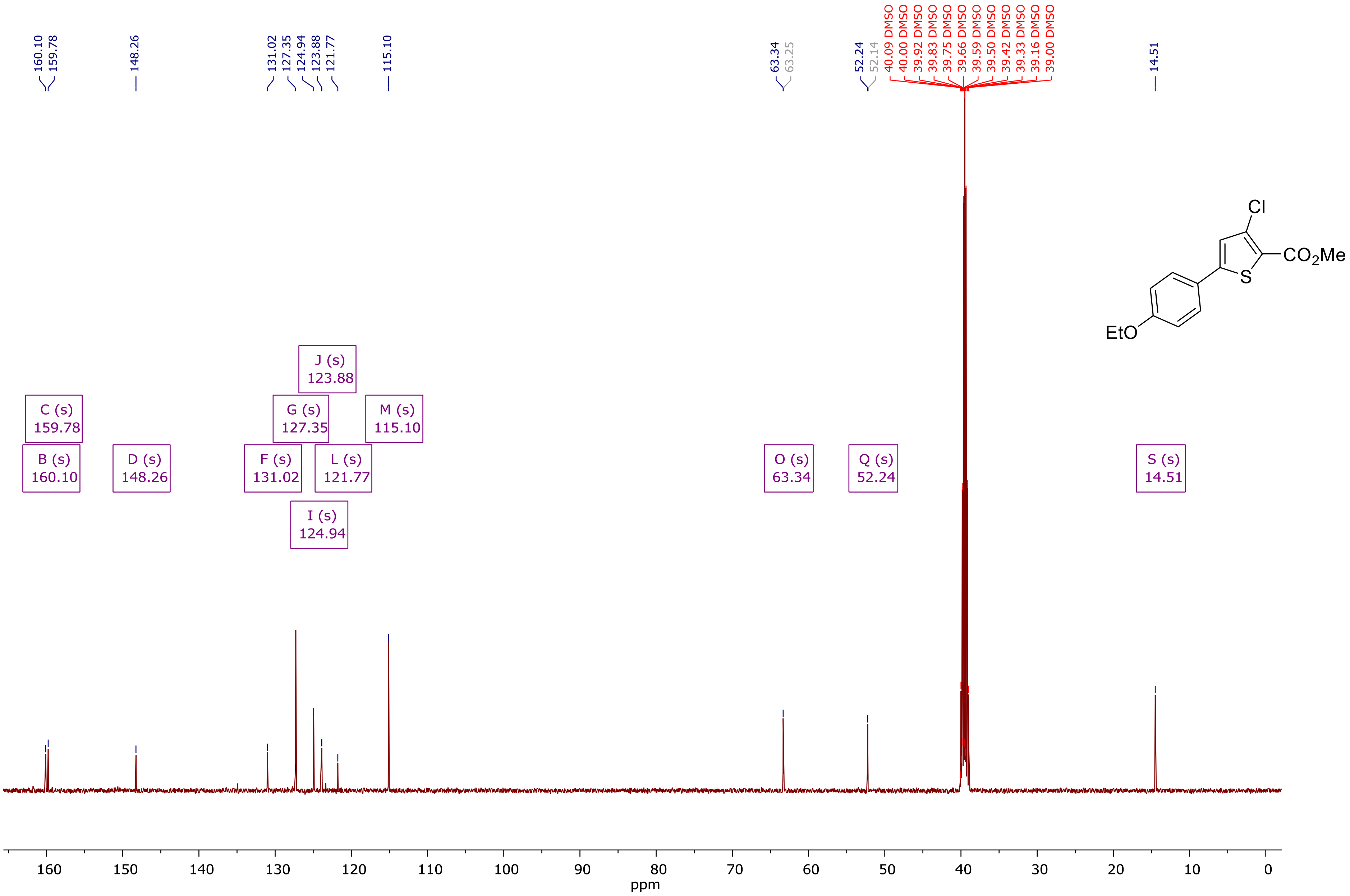

^{13}C NMR (126 MHz, DMSO-d_6) δ 160.1, 159.8, 148.3, 131.0, 127.3, 124.9, 123.9, 121.8, 115.1, 63.3, 52.2, 14.5.

Methyl 3-chloro-5-(naphthalen-2-yl)thiophene-2-carboxylate (2j)

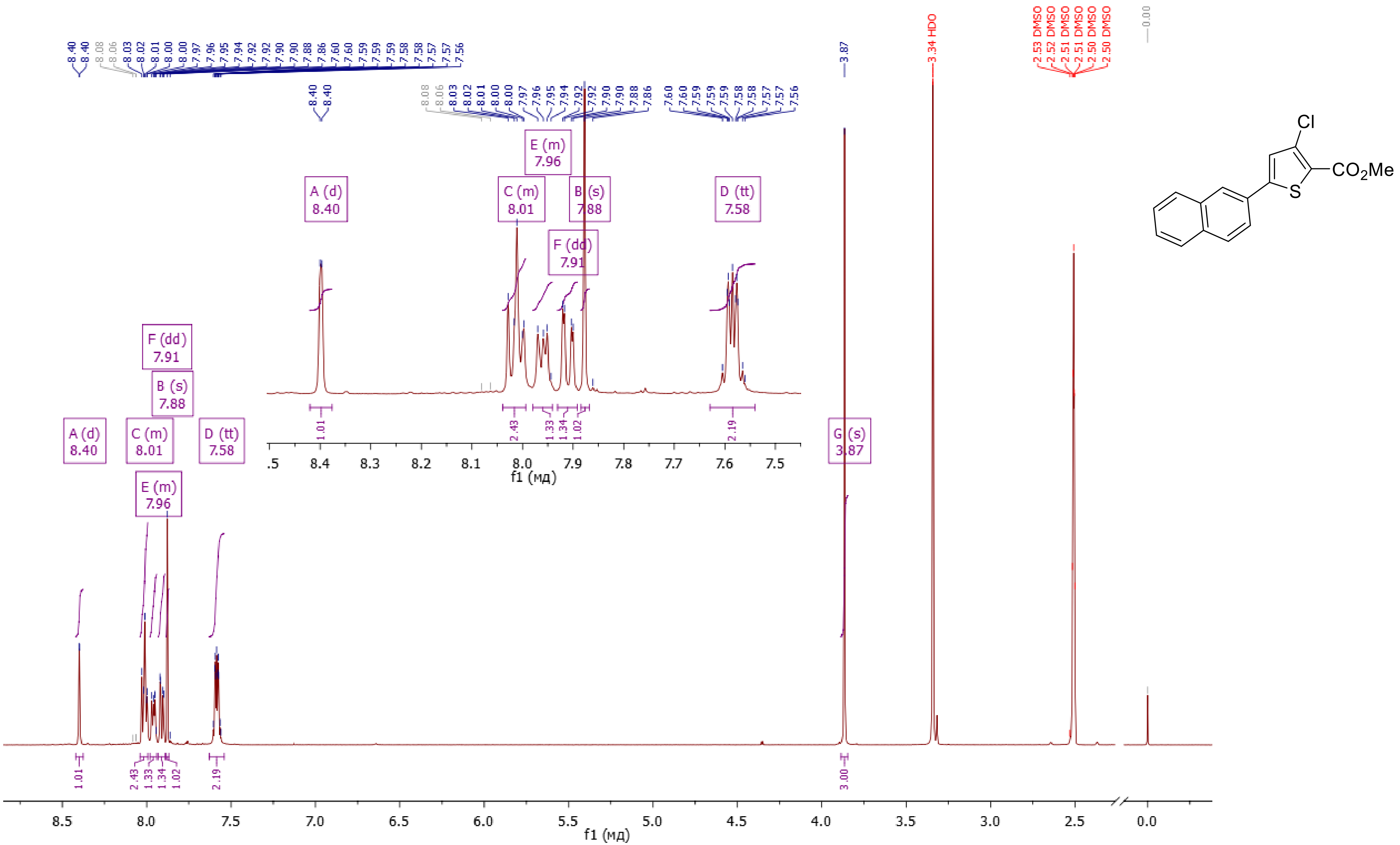

^{1}H NMR (500 MHz, DMSO-d_6) δ 8.40 (d, *J* = 1.8 Hz, 1H), 8.04 – 7.99 (m, 2H), 7.98 – 7.94 (m, 1H), 7.91 (dd, *J* = 8.6, 1.9 Hz, 1H), 7.88 (s, 1H), 7.58 (tt, *J* = 5.6, 4.6 Hz, 2H), 3.87 (s, 3H).

Methyl 3-chloro-6-phenylthiophene-2-carboxylate (2k)

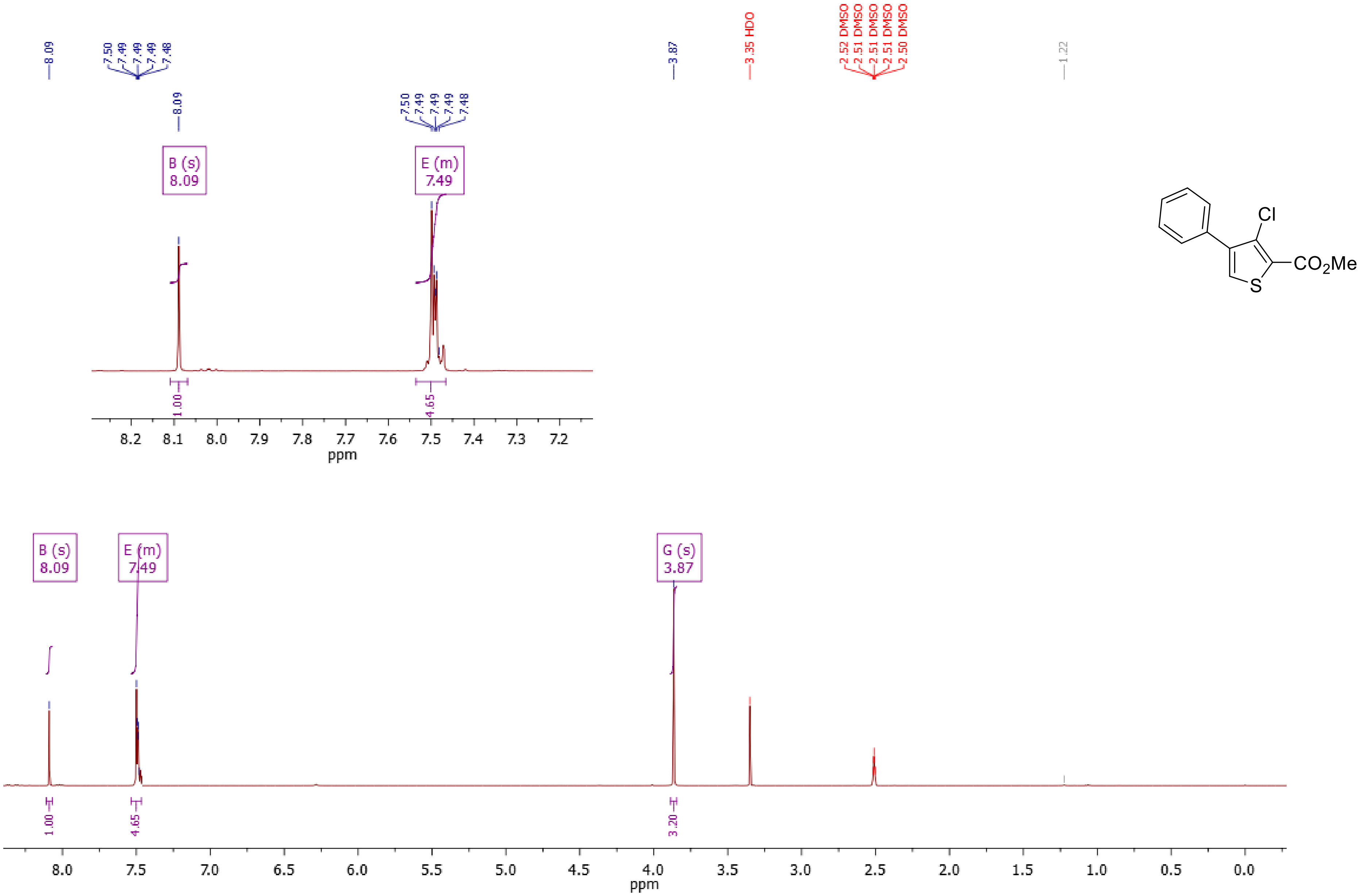

^{1}H NMR (500 MHz, DMSO-d_6) δ 8.09 (s, 1H), 7.54 – 7.47 (m, 5H), 3.87 (s, 3H).

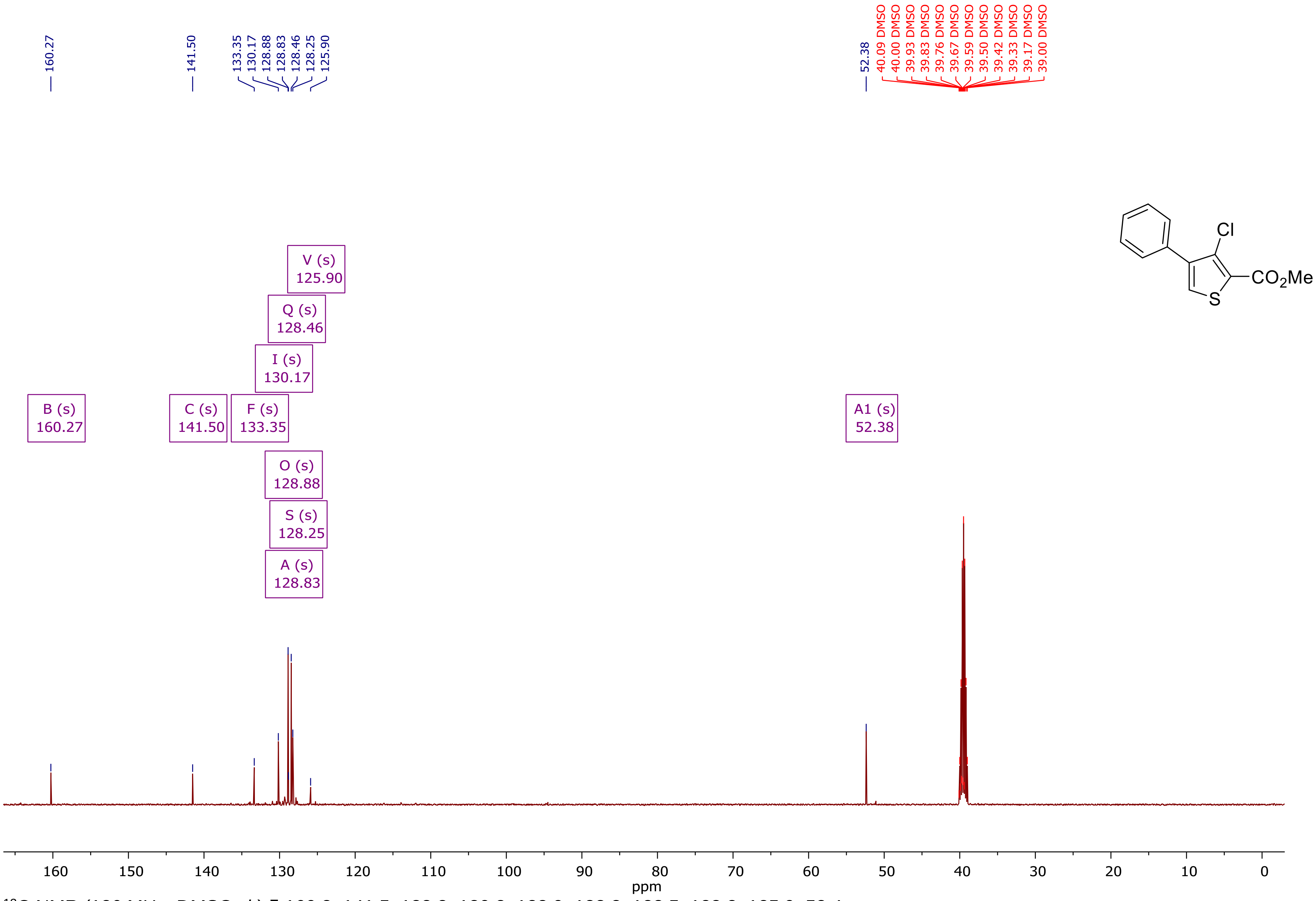

^{13}C NMR (126 MHz, DMSO-d_6) δ 160.3, 141.5, 133.3, 130.2, 128.9, 128.8, 128.5, 128.2, 125.9, 52.4.

Methyl 3-hydroxy-5-phenylthieno[3,2-*b*]thiophene-2-carboxylate (3a)

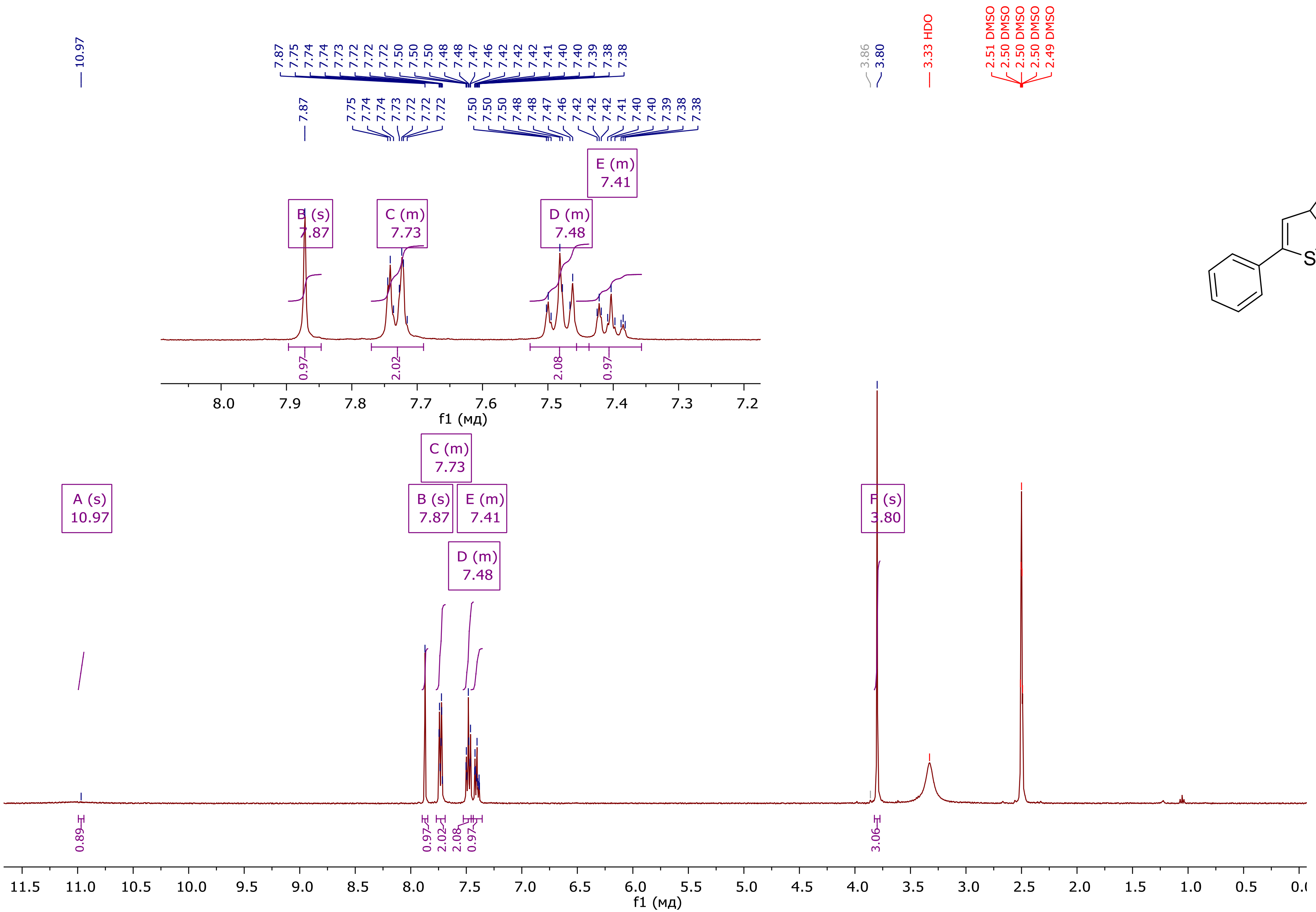

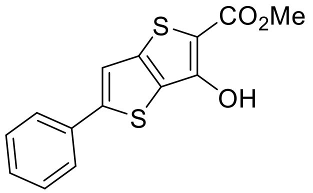

^{1}H NMR (400 MHz, DMSO-d_6) δ 10.97 (s, 1H), 7.87 (s, 1H), 7.77 – 7.69 (m, 2H), 7.53 – 7.44 (m, 2H), 7.46 – 7.36 (m, 1H), 3.80 (s, 3H).

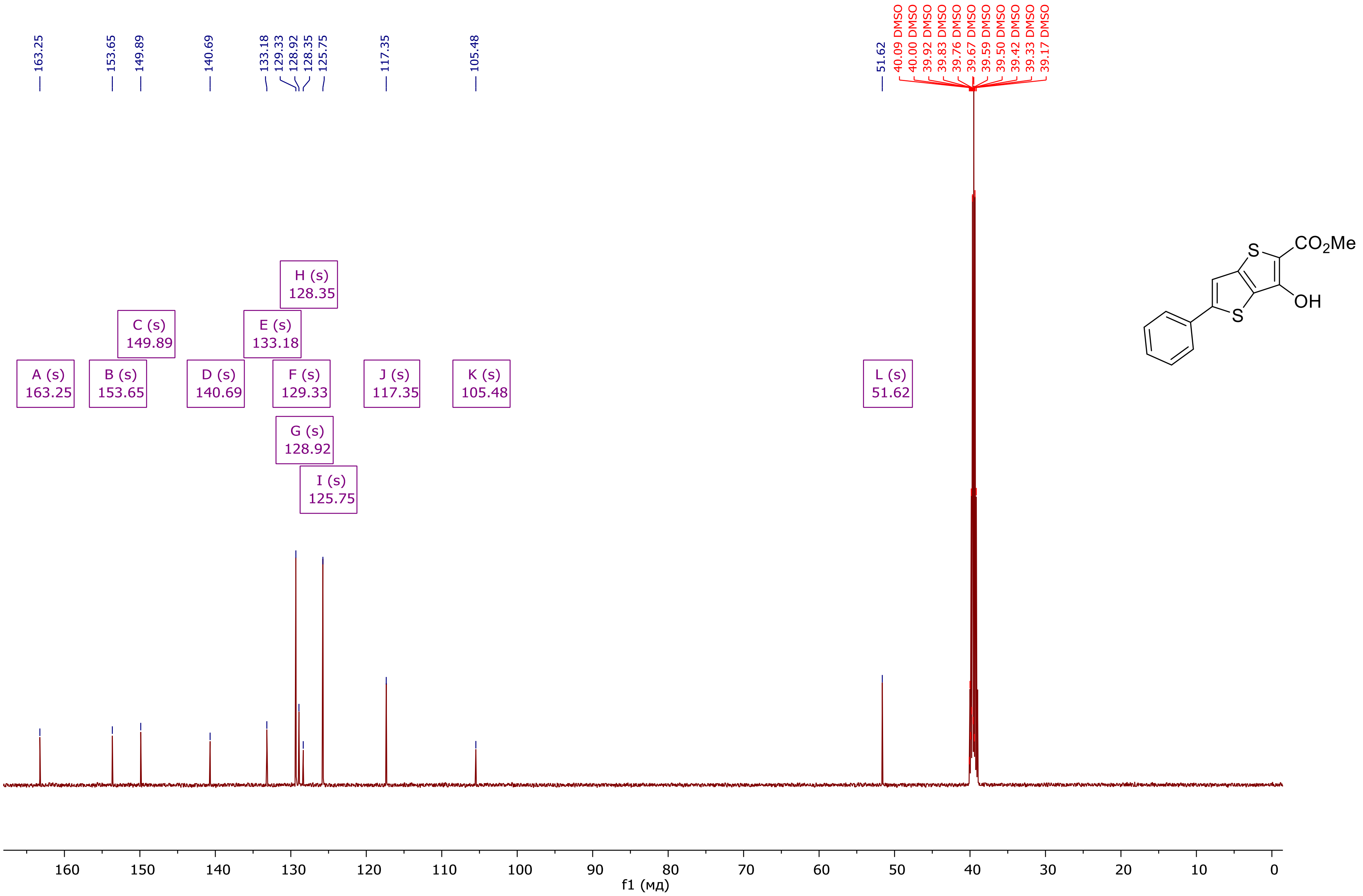

^{13}C NMR (126 MHz, DMSO-*d*$_6$) δ 163.2, 153.6, 149.9, 140.7, 133.2, 129.3, 128.9, 128.3, 125.7, 117.3, 105.5, 51.6.

Methyl 3-hydroxy-5-(*p*-tolyl)thieno[3,2-*b*]thiophene-2-carboxylate (3b)

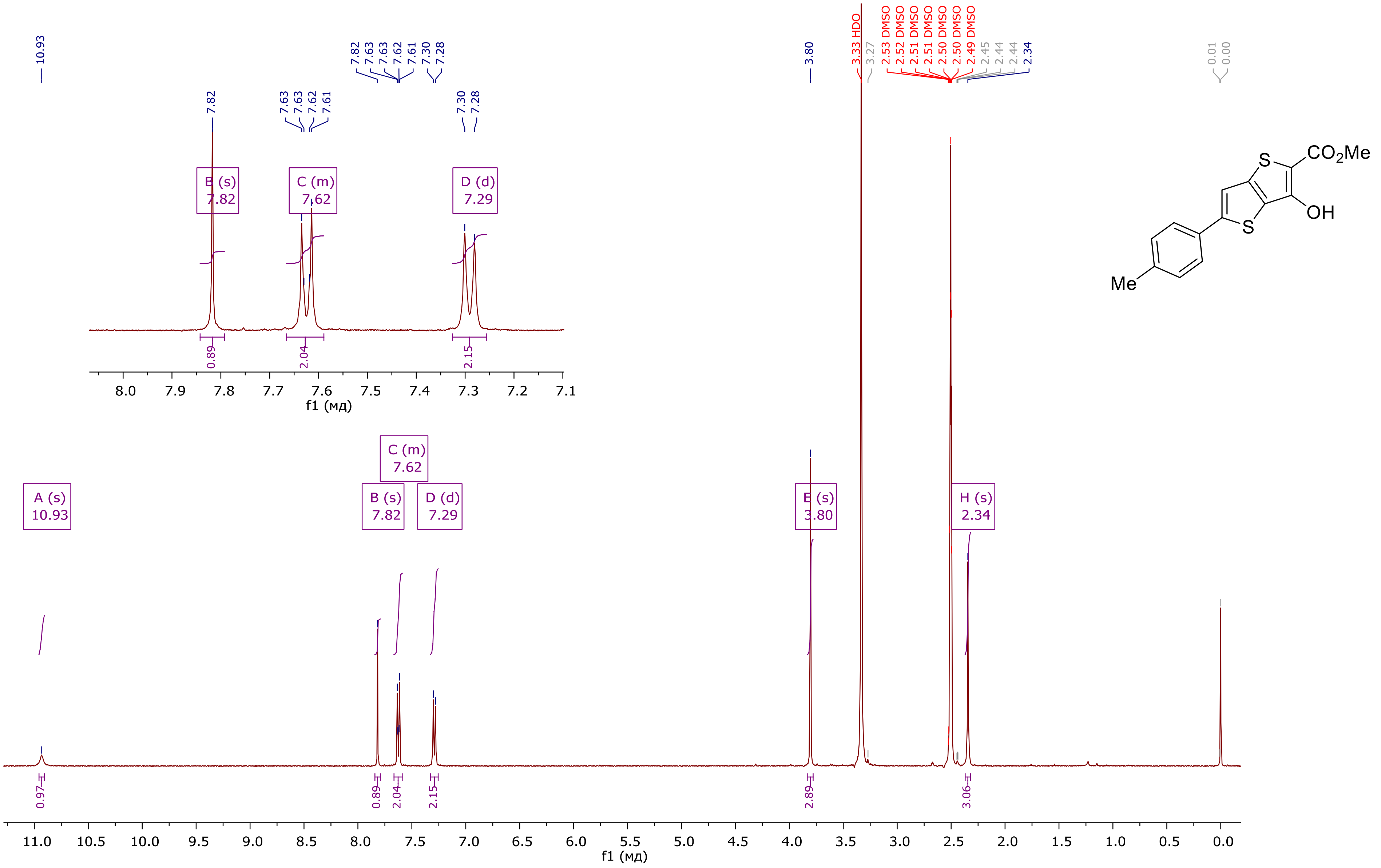

^{1}H NMR (400 MHz, DMSO-d_6) δ 10.93 (s, 1H), 7.82 (s, 1H), 7.67 – 7.59 (m, 2H), 7.29 (d, *J* = 8.0 Hz, 2H), 3.80 (s, 3H), 2.34 (s, 3H).

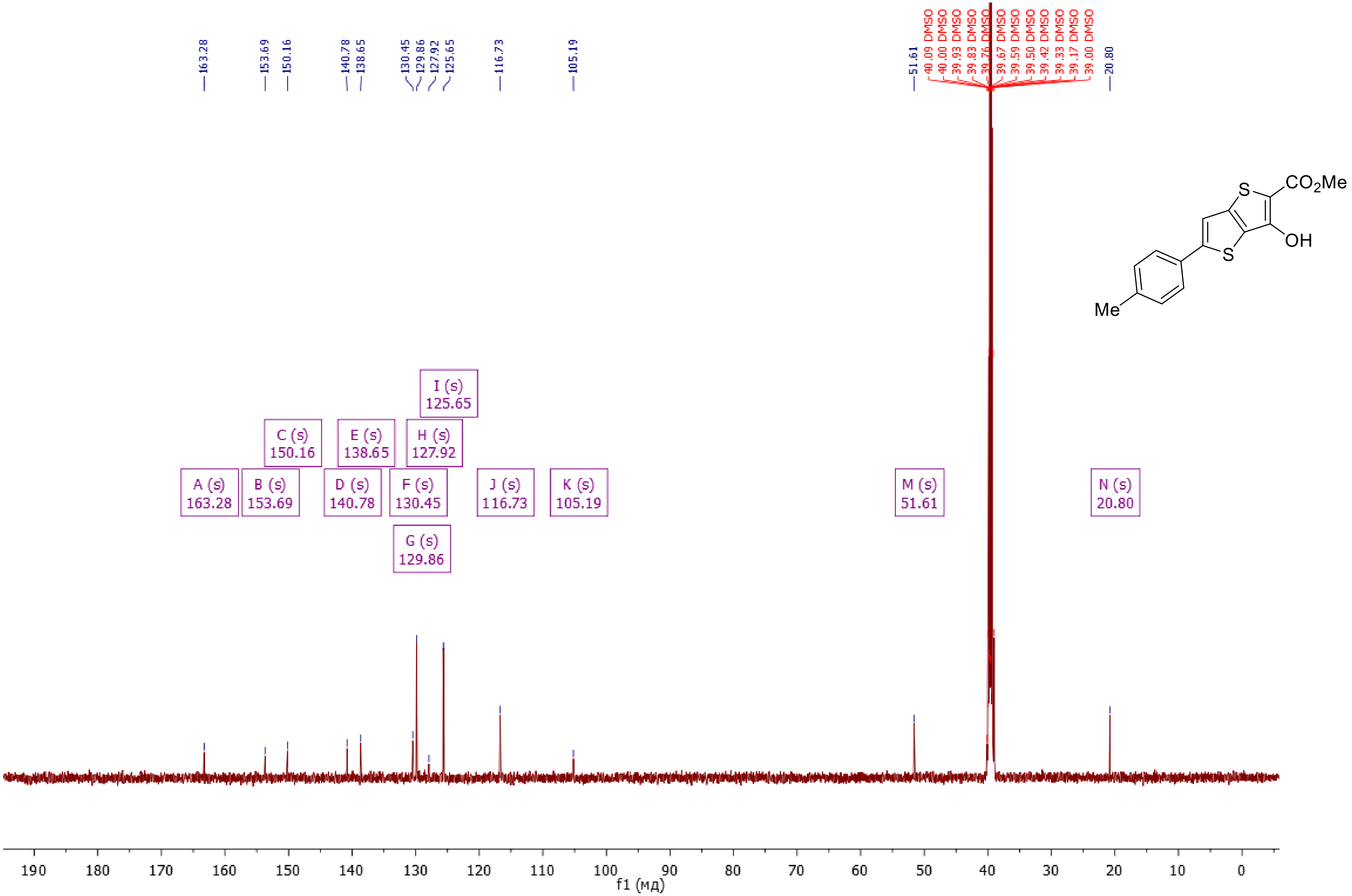

^{13}C NMR (126 MHz, DMSO-d_6) δ 163.3, 153.7, 150.2, 140.8, 138.6, 130.4, 129.9, 127.9, 125.6, 116.7, 105.1, 51.6, 20.8.

Methyl 5-(2,5-dimethylphenyl)-3-hydroxythieno[3,2-*b*]thiophene-2-carboxylate (3c)

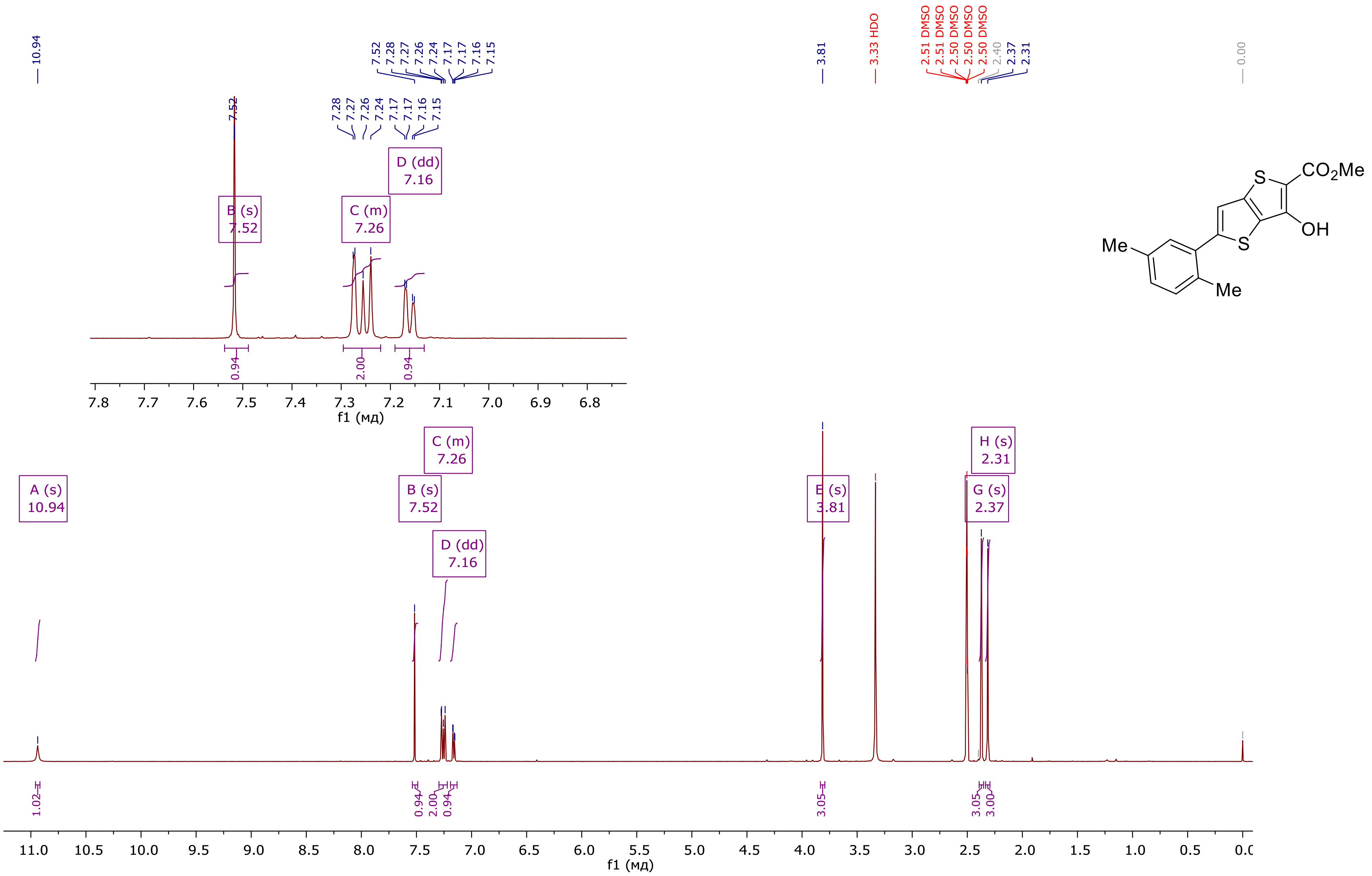

^{1}H NMR (500 MHz, DMSO-d_6) δ 10.94 (s, 1H), 7.52 (s, 1H), 7.30 – 7.22 (m, 2H), 7.16 (dd, *J* = 7.9, 1.8 Hz, 1H), 3.81 (s, 3H), 2.37 (s, 3H), 2.31 (s, 3H).

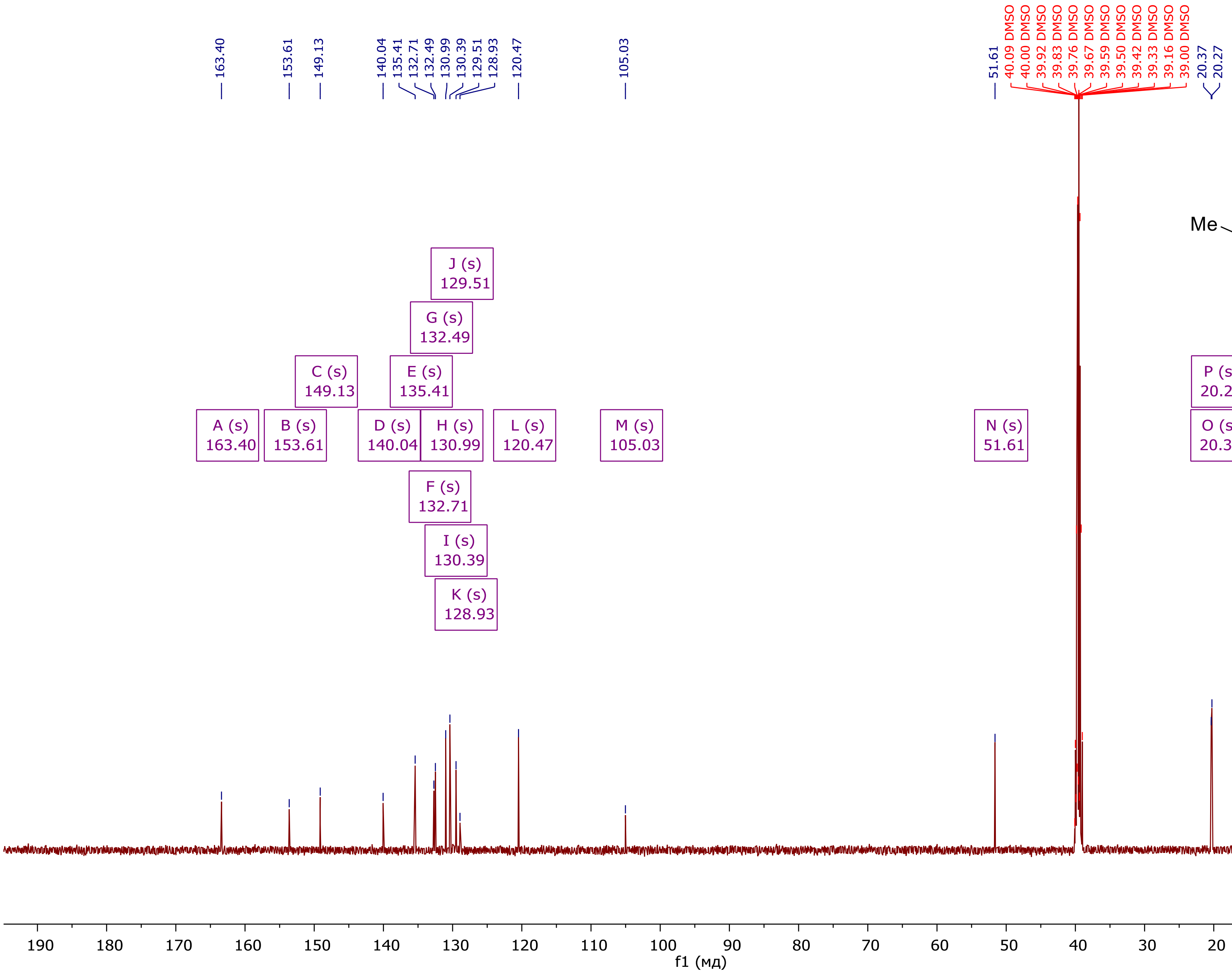

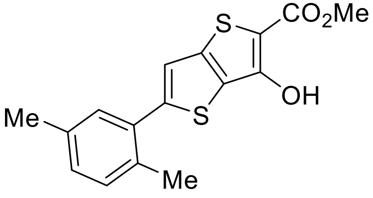

^{13}C NMR (126 MHz, DMSO-d_6) δ 163.4, 153.6, 149.1, 140.0, 135.4, 132.7, 132.5, 131.0, 130.4, 129.5, 128.9, 120.5, 105.0, 51.6, 20.4, 20.23.

Methyl 5-(4-(*tert*-butyl)phenyl)-3-hydroxythieno[3,2-*b*]thiophene-2-carboxylate (3d)

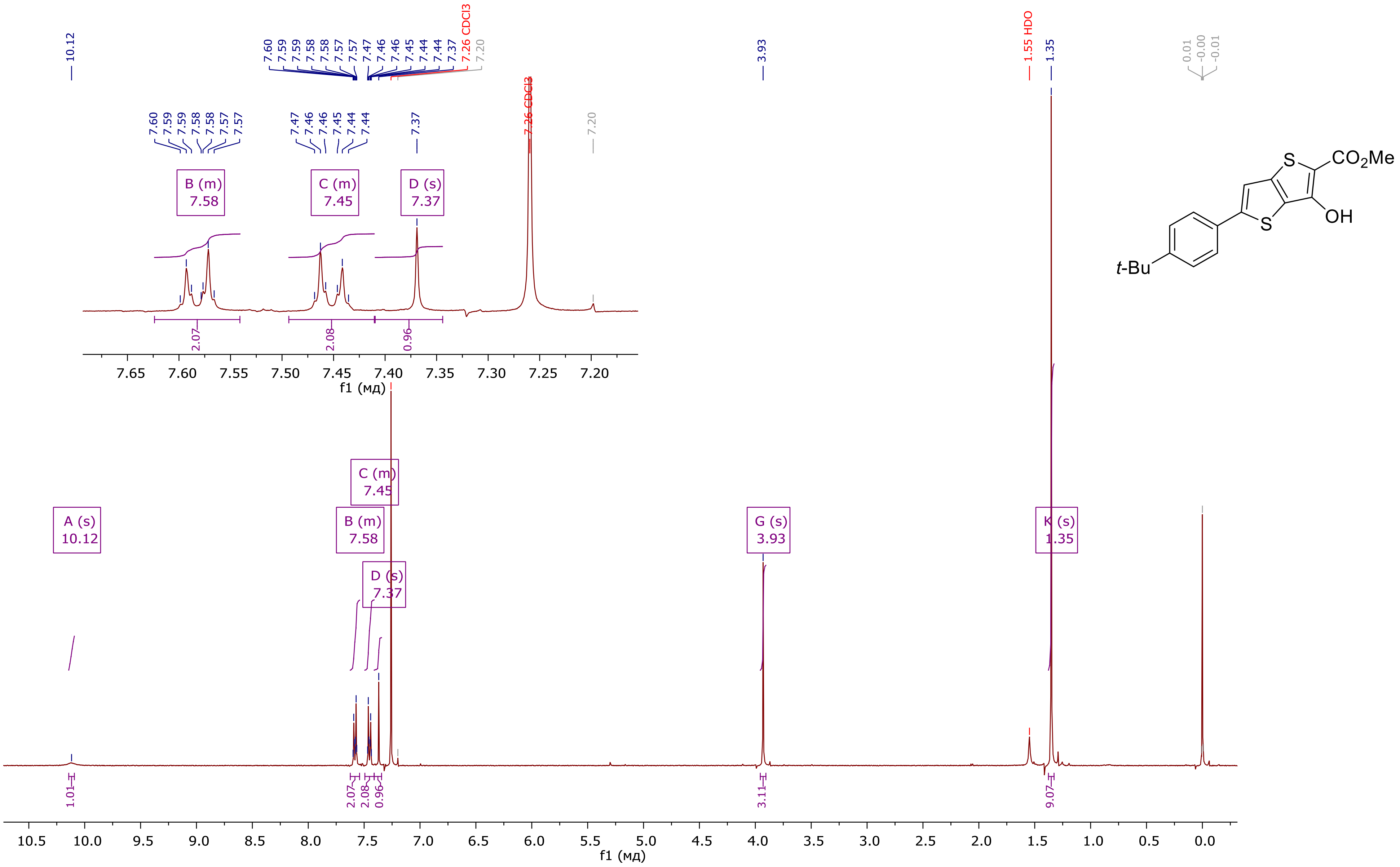

^{1}H NMR (400 MHz, chloroform-*d*) δ 10.12 (s, 1H), 7.62 – 7.54 (m, 2H), 7.49 – 7.41 (m, 2H), 7.37 (s, 1H), 3.93 (s, 3H), 1.35 (s, 9H).

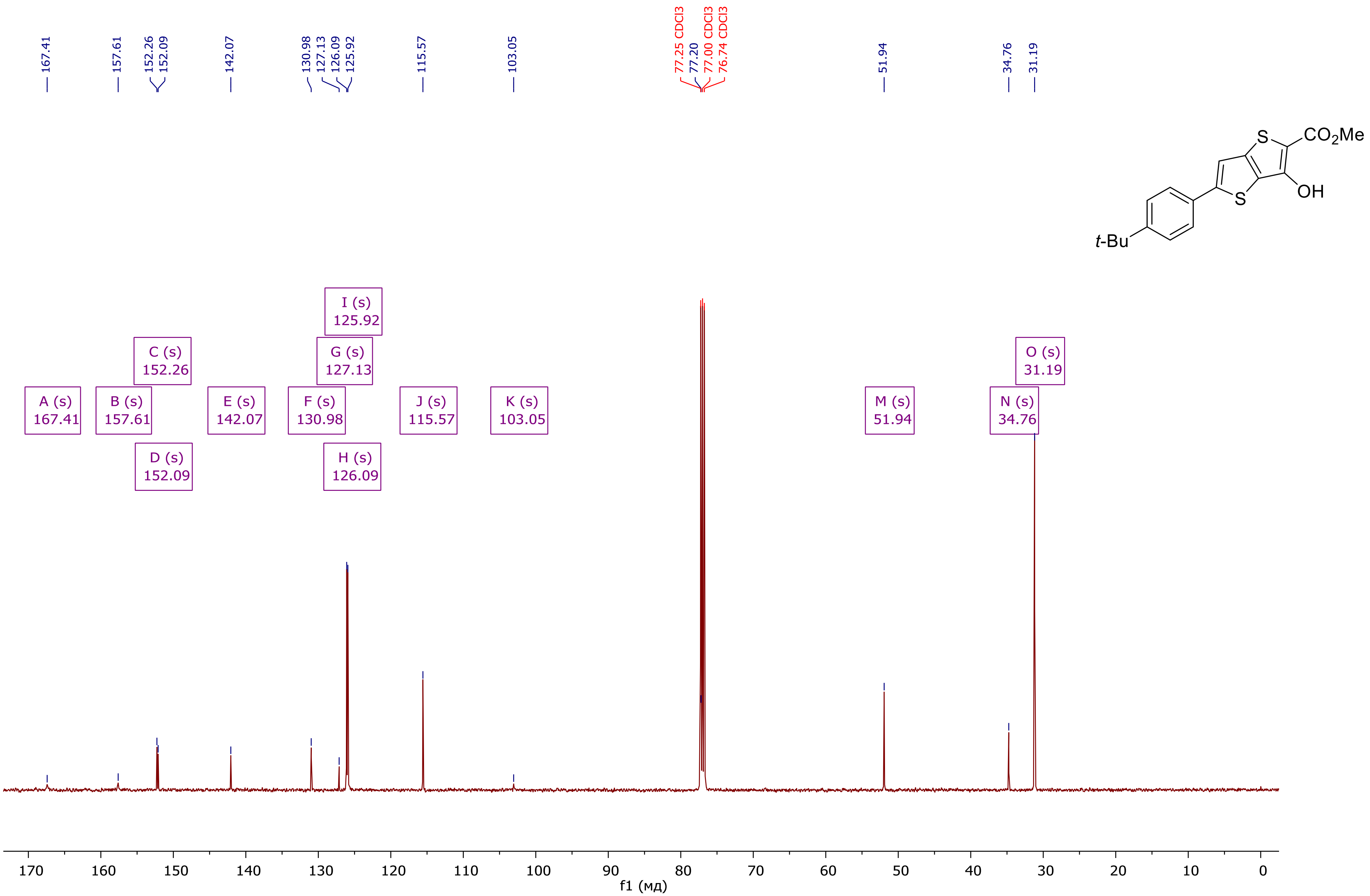

^{13}C NMR (126 MHz, chloroform-*d*) δ 167.4, 157.6, 152.3, 152.1, 142.1, 131.0, 127.1, 126.1, 125.9, 115.6, 103.1, 51.9, 34.8, 31.2.

Methyl 5-(4-fluorophenyl)-3-hydroxythieno[3,2-*b*]thiophene-2-carboxylate (3e)

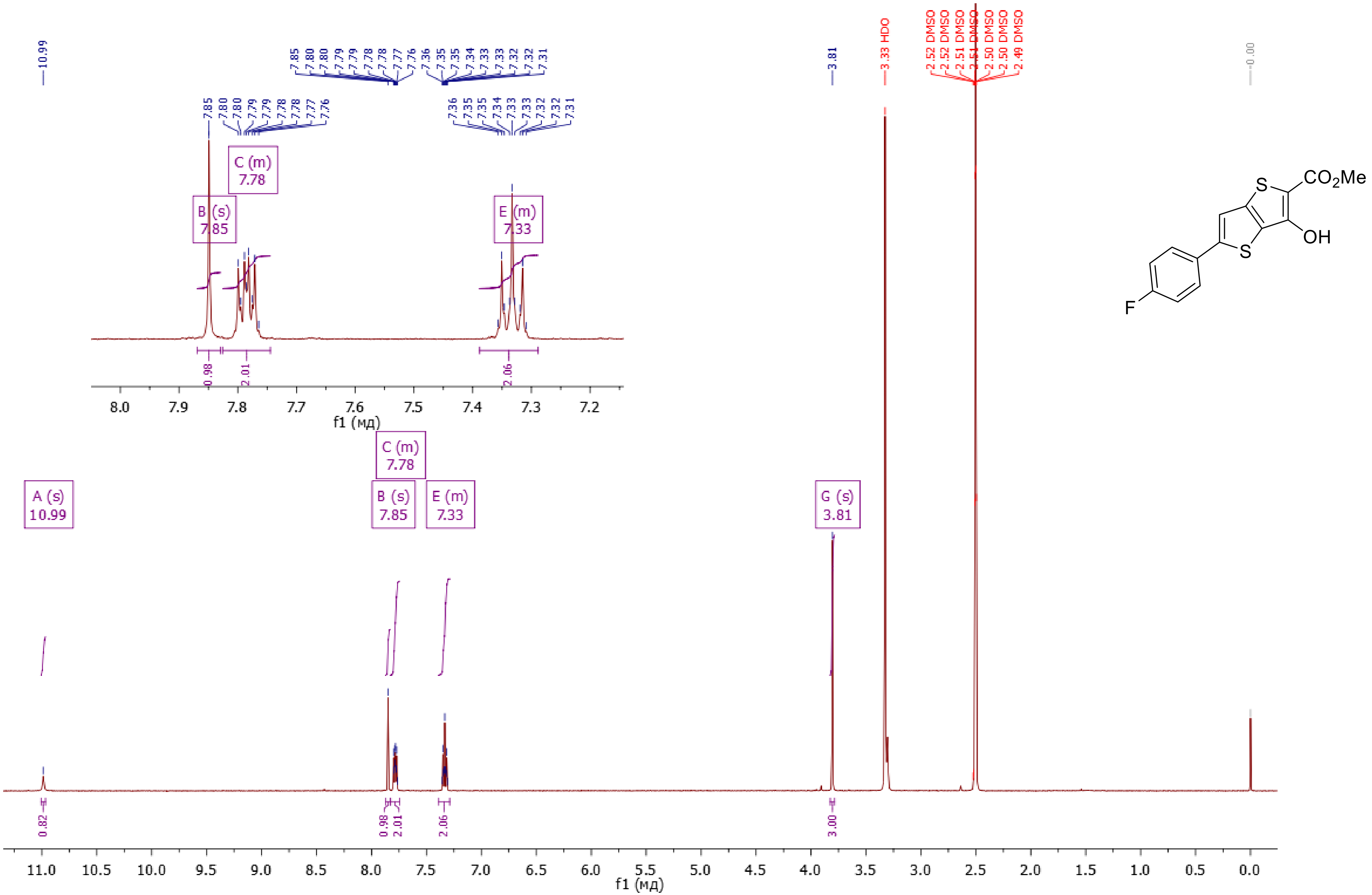

^{1}H NMR (500 MHz, DMSO-d_6) δ 10.99 (s, 1H), 7.85 (s, 1H), 7.83 – 7.74 (m, 2H), 7.39 – 7.29 (m, 2H), 3.81 (s, 3H).

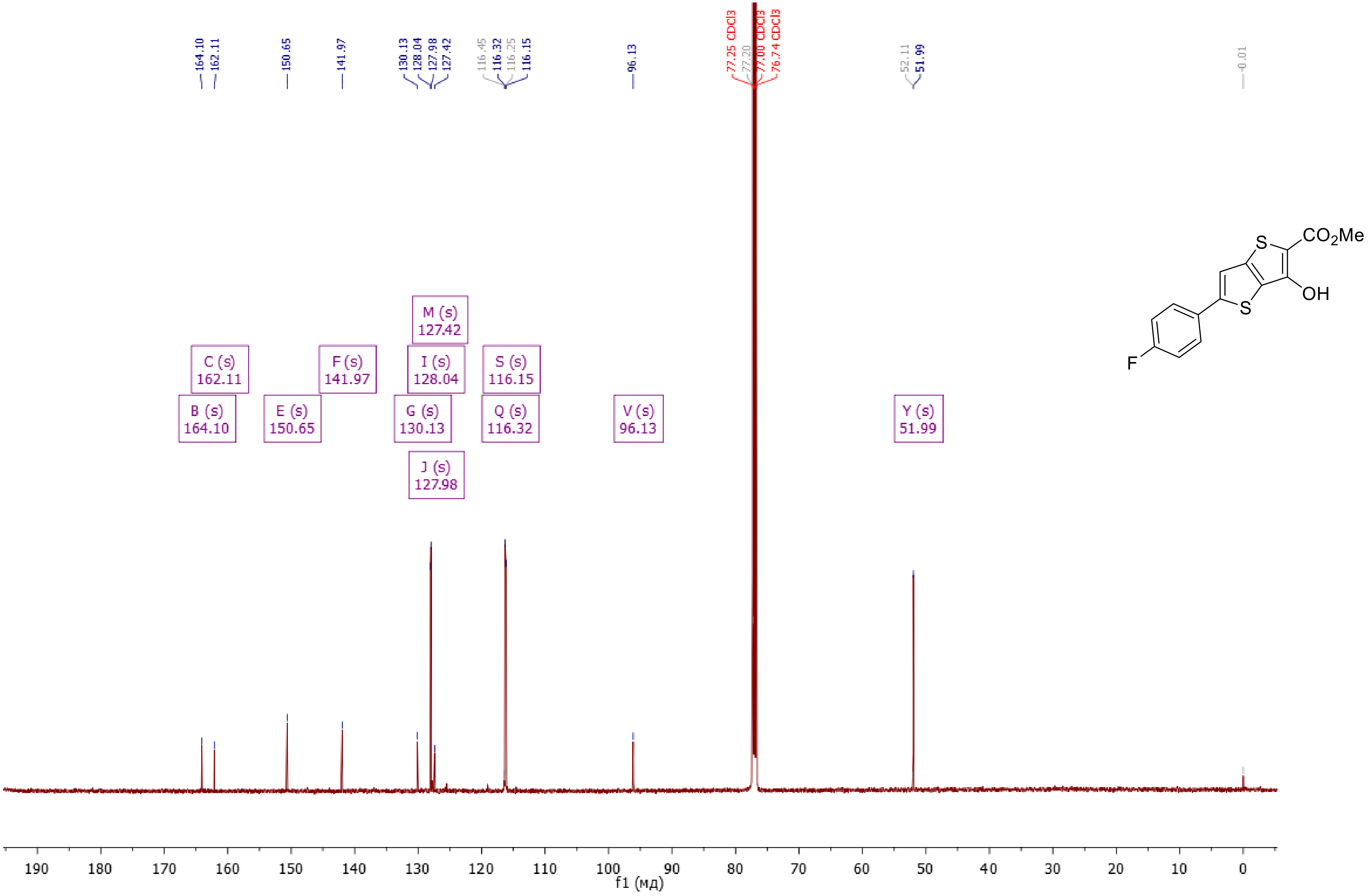

^{13}C NMR (126 MHz, chloroform-*d*) δ 164.1, 162.1, 150.6, 142.0, 130.1, 128.0, 128.0, 127.4, 116.3, 116.1, 96.1, 52.0.

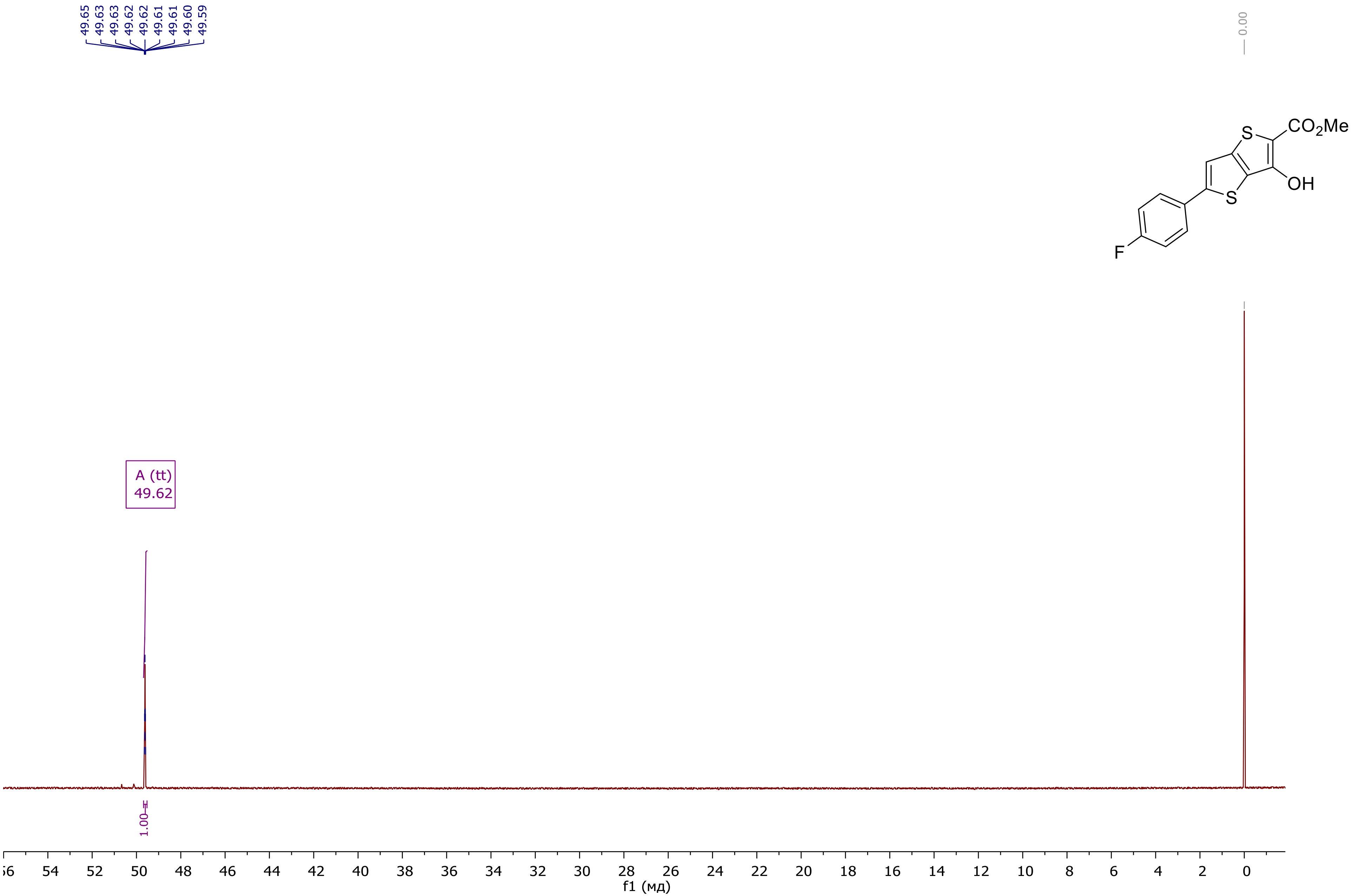

^{19}F NMR (471 MHz, chloroform-*d*) δ 49.62 (tt, *J* = 8.4, 5.1 Hz).

Methyl 5-(4-chlorophenyl)-3-hydroxythieno[3,2-*b*]thiophene-2-carboxylate (3f)

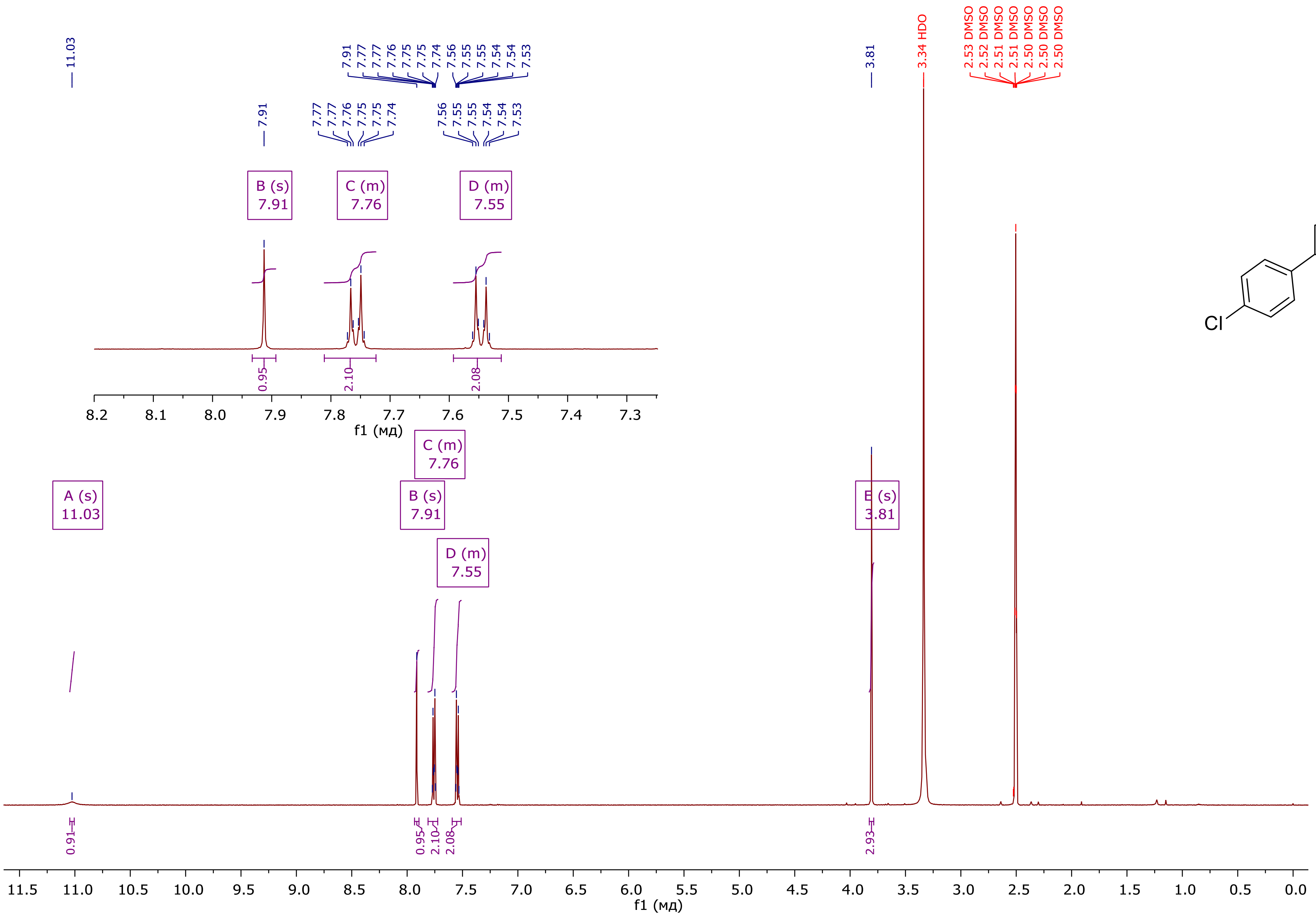

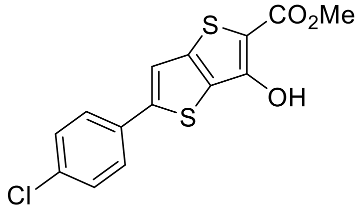

^{1}H NMR (500 MHz, DMSO-d_6) δ 11.03 (s, 1H), 7.91 (s, 1H), 7.81 – 7.72 (m, 2H), 7.59 – 7.51 (m, 2H), 3.81 (s, 3H).

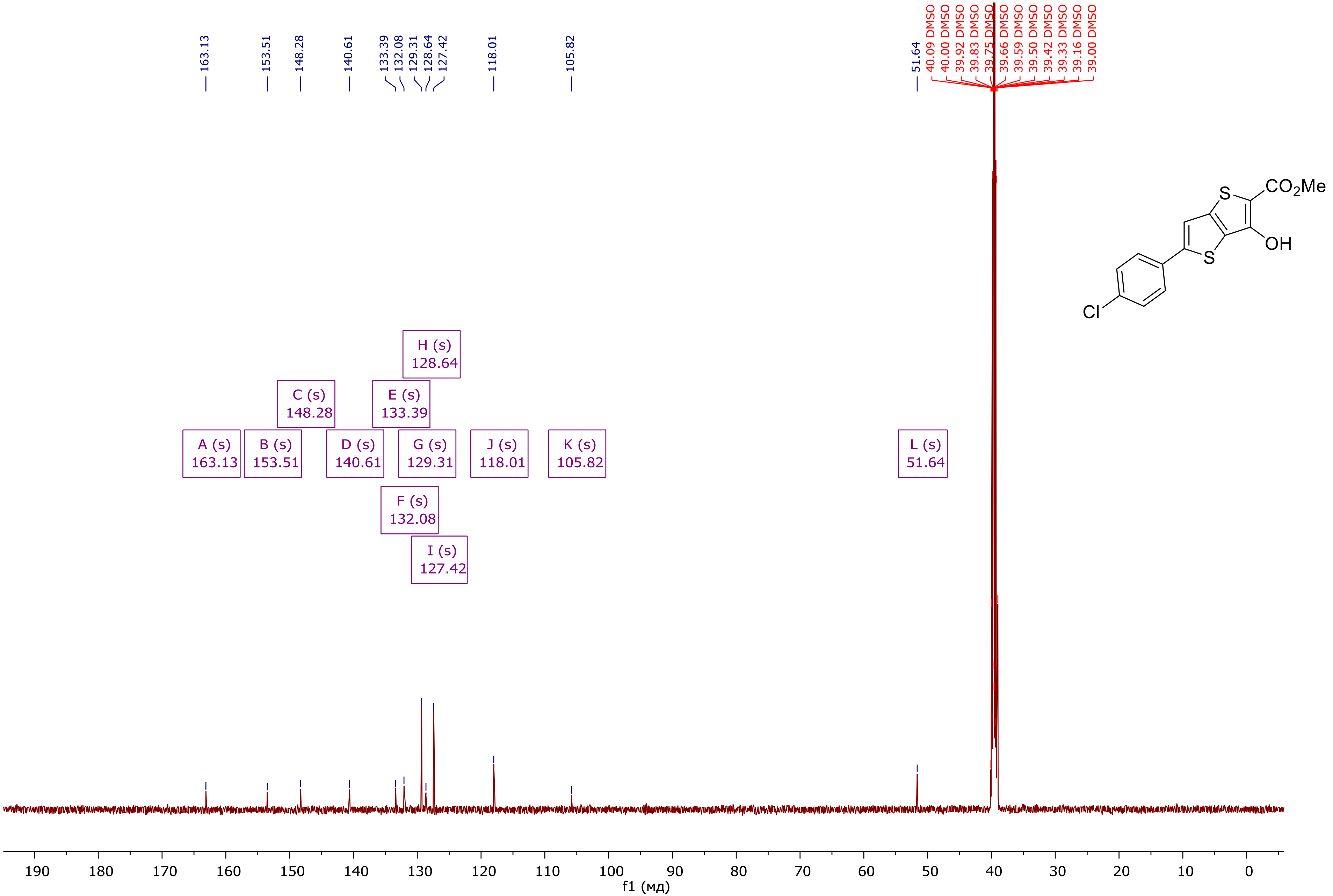

^{13}C NMR (126 MHz, DMSO-d_6) δ 163., 153.5, 148.3, 140.6, 133.4, 132.1, 129.3, 128.6, 127.4, 118.0, 105.8, 51.6.

Methyl 5-(4-bromophenyl)-3-hydroxythieno[3,2-*b*]thiophene-2-carboxylate (3g)

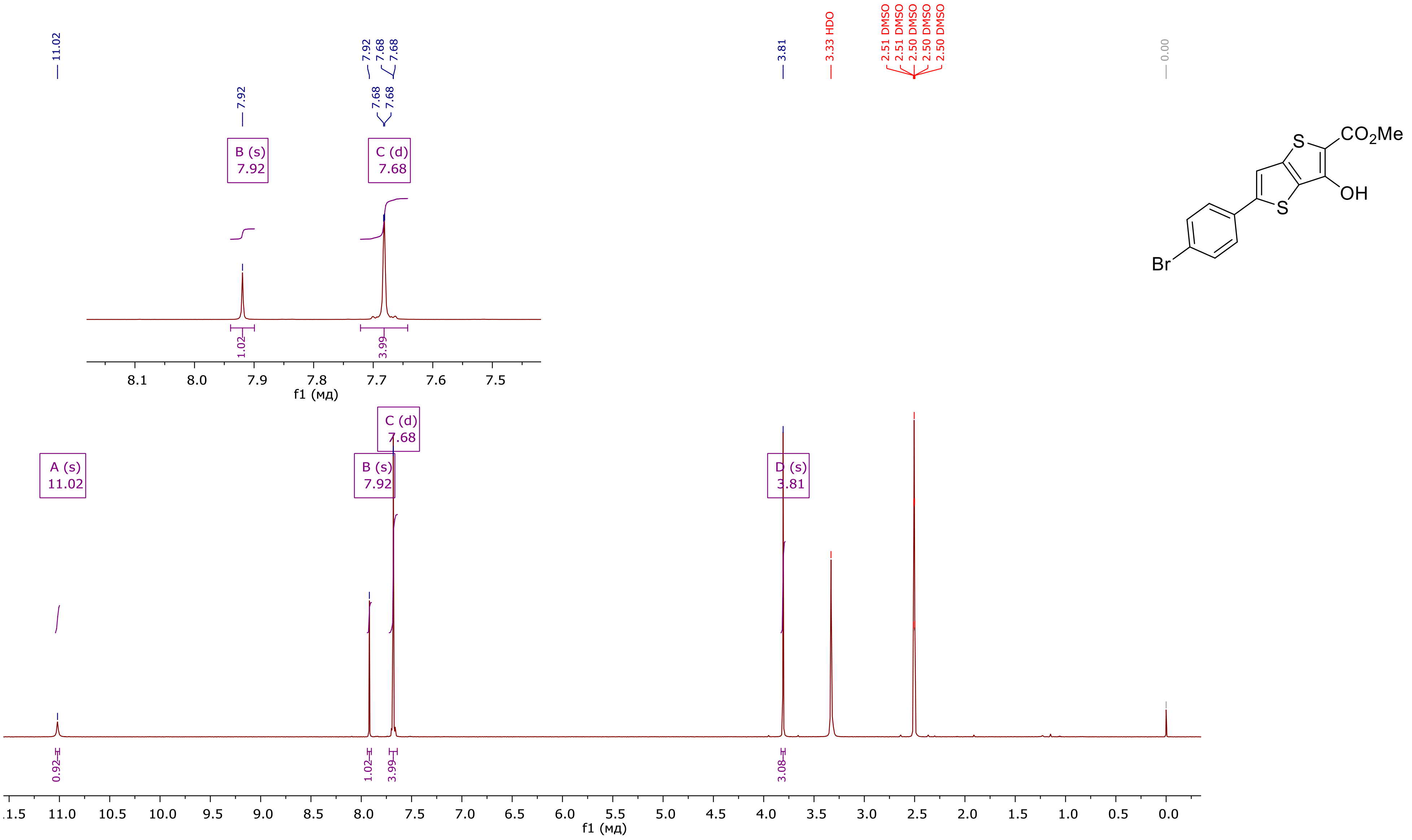

^{1}H NMR (500 MHz, DMSO-d_6) δ 11.02 (s, 1H), 7.92 (s, 1H), 7.68 (d, *J* = 0.9 Hz, 4H), 3.81 (s, 3H).

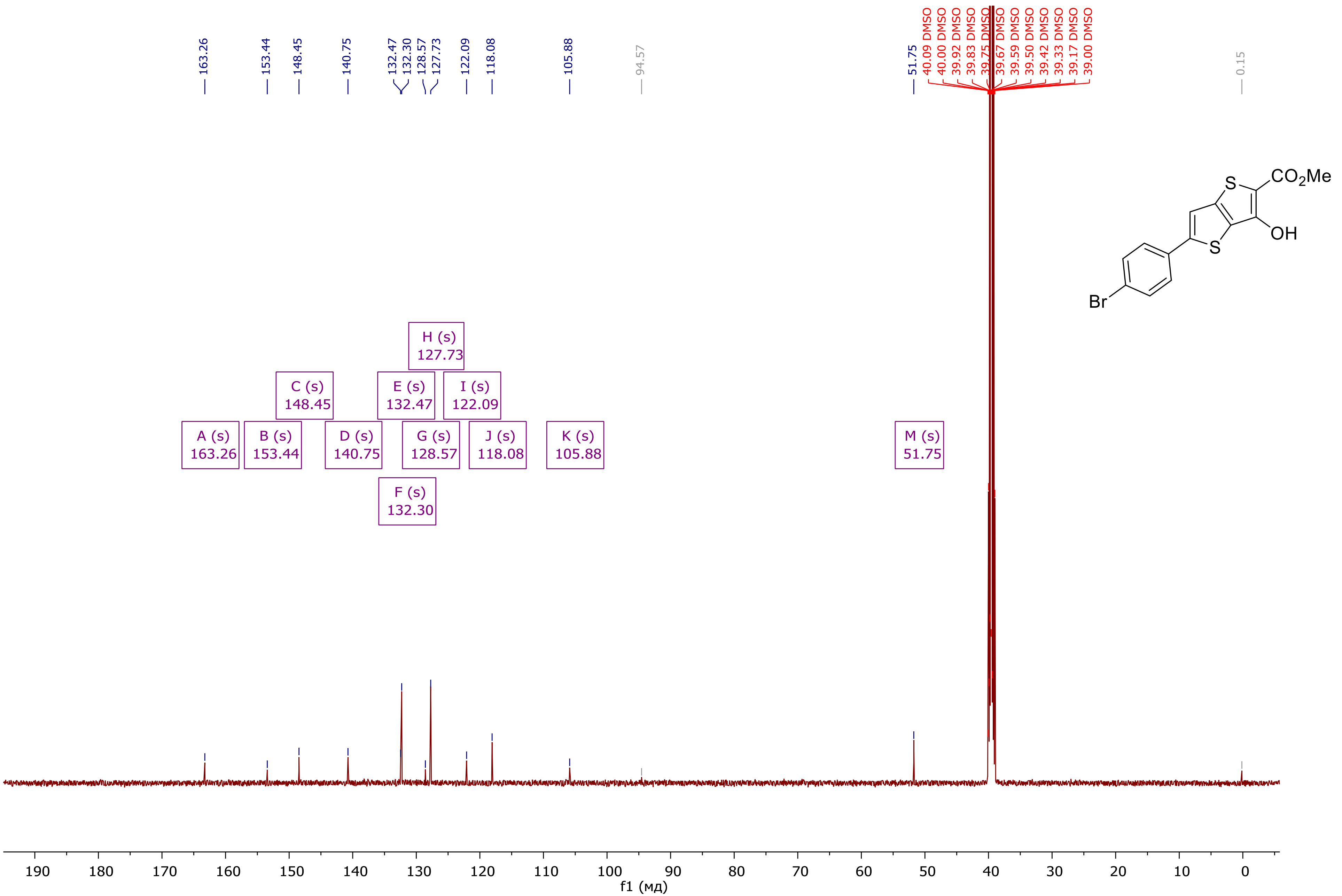

^{13}C NMR (126 MHz, DMSO-d_6) δ 163.3, 153.4, 148.4, 140.7, 132.5, 132.3, 128.6, 127.7, 122.1, 118.1, 105.9, 51.7.

Methyl 3-hydroxy-5-(3-methoxyphenyl)thieno[3,2-*b*]thiophene-2-carboxylate (3h)

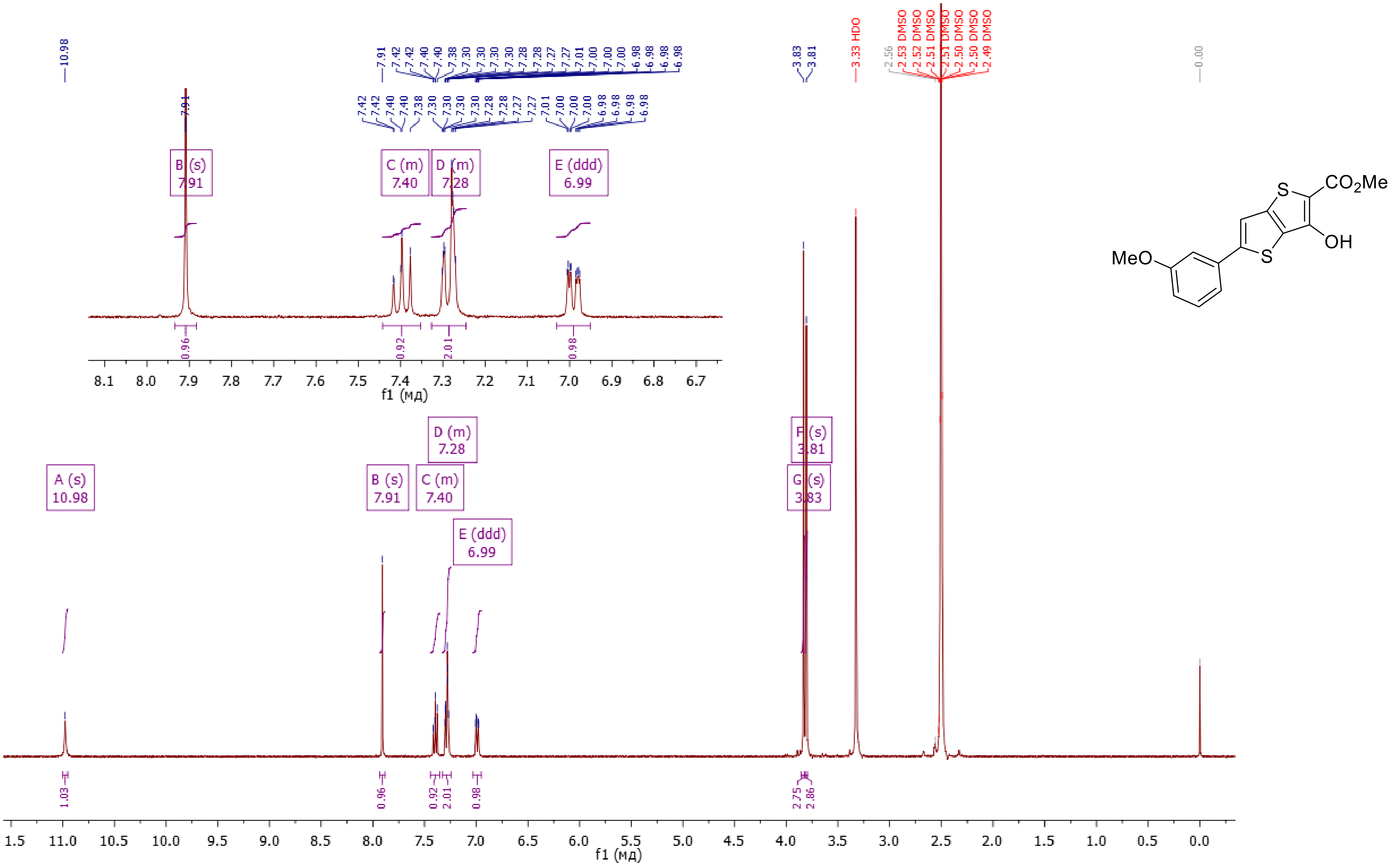

^{1}H NMR (400 MHz, DMSO-d_6) δ 10.98 (s, 1H), 7.91 (s, 1H), 7.44 – 7.35 (m, 1H), 7.33 – 7.24 (m, 2H), 6.99 (ddd, *J* = 8.3, 2.4, 1.1 Hz, 1H), 3.83 (s, 6H), 3.81 (s, 3H).

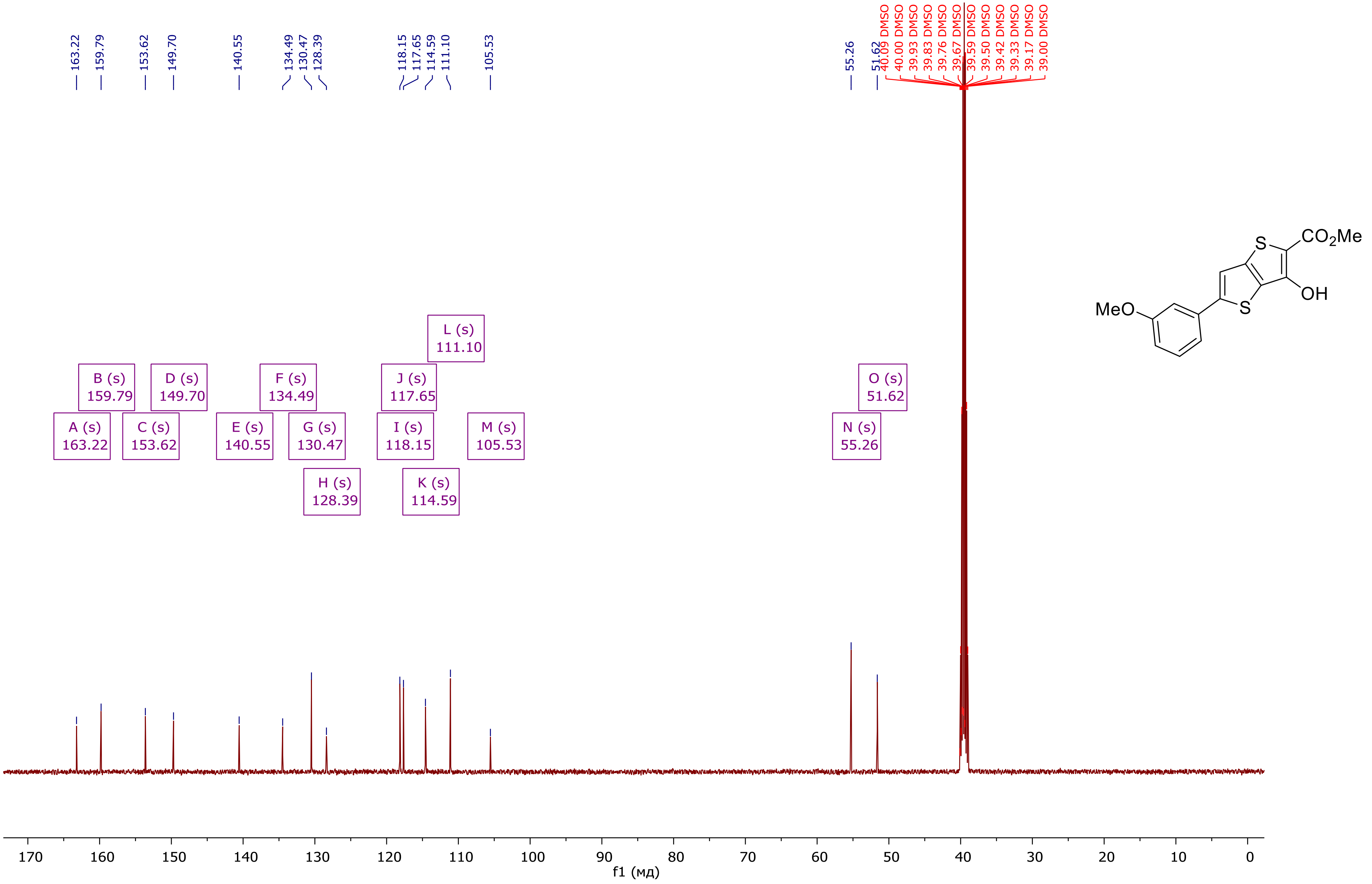

^{13}C NMR (126 MHz, DMSO-d_6) δ 163.2, 159.8, 153.6, 149.7, 140.5, 134.5, 130.5, 128.4, 118.1, 117.6, 114.6, 111.1, 105.5, 55.5, 51.6.

Methyl 5-(4-ethoxyphenyl)-3-hydroxythieno[3,2-*b*]thiophene-2-carboxylate (3i)

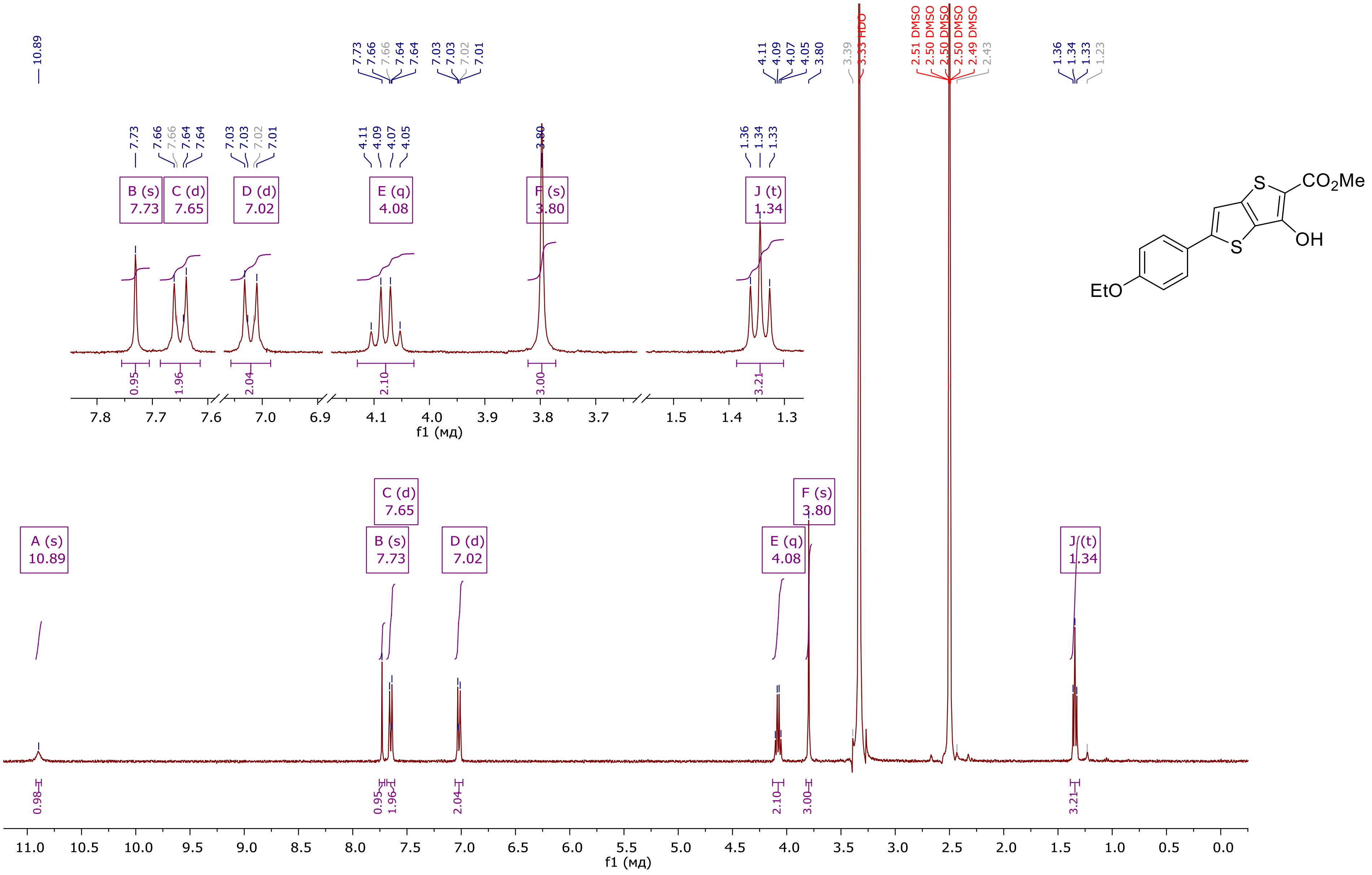

^{1}H NMR (400 MHz, DMSO-d_6) δ 10.89 (s, 1H), 7.73 (s, 1H), 7.65 (d, *J* = 8.8 Hz, 2H), 7.02 (d, *J* = 8.7 Hz, 2H), 4.08 (q, *J* = 6.9 Hz, 2H), 3.80 (s, 3H), 1.34 (t, *J* = 6.9 Hz, 3H).

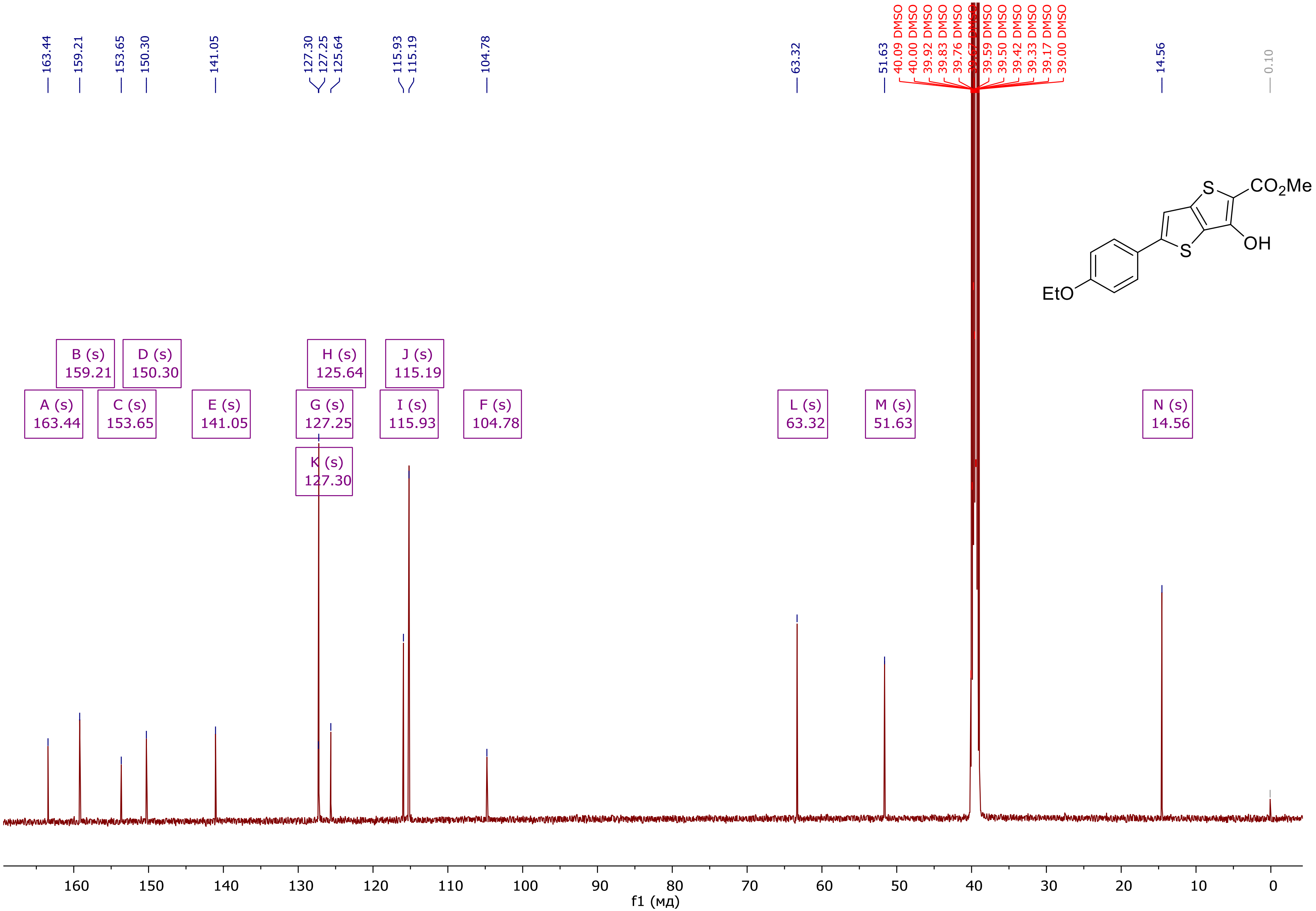

^{13}C NMR (126 MHz, DMSO-d_6) δ 163.4, 159.2, 153.6, 150.3, 141.0, 127.3, 127.2, 125.6, 115.9, 115.2, 104.8, 63.3, 51.6, 14.6.

Methyl 3-hydroxy-5-(naphthalen-2-yl)thieno[3,2-*b*]thiophene-2-carboxylate (3j)

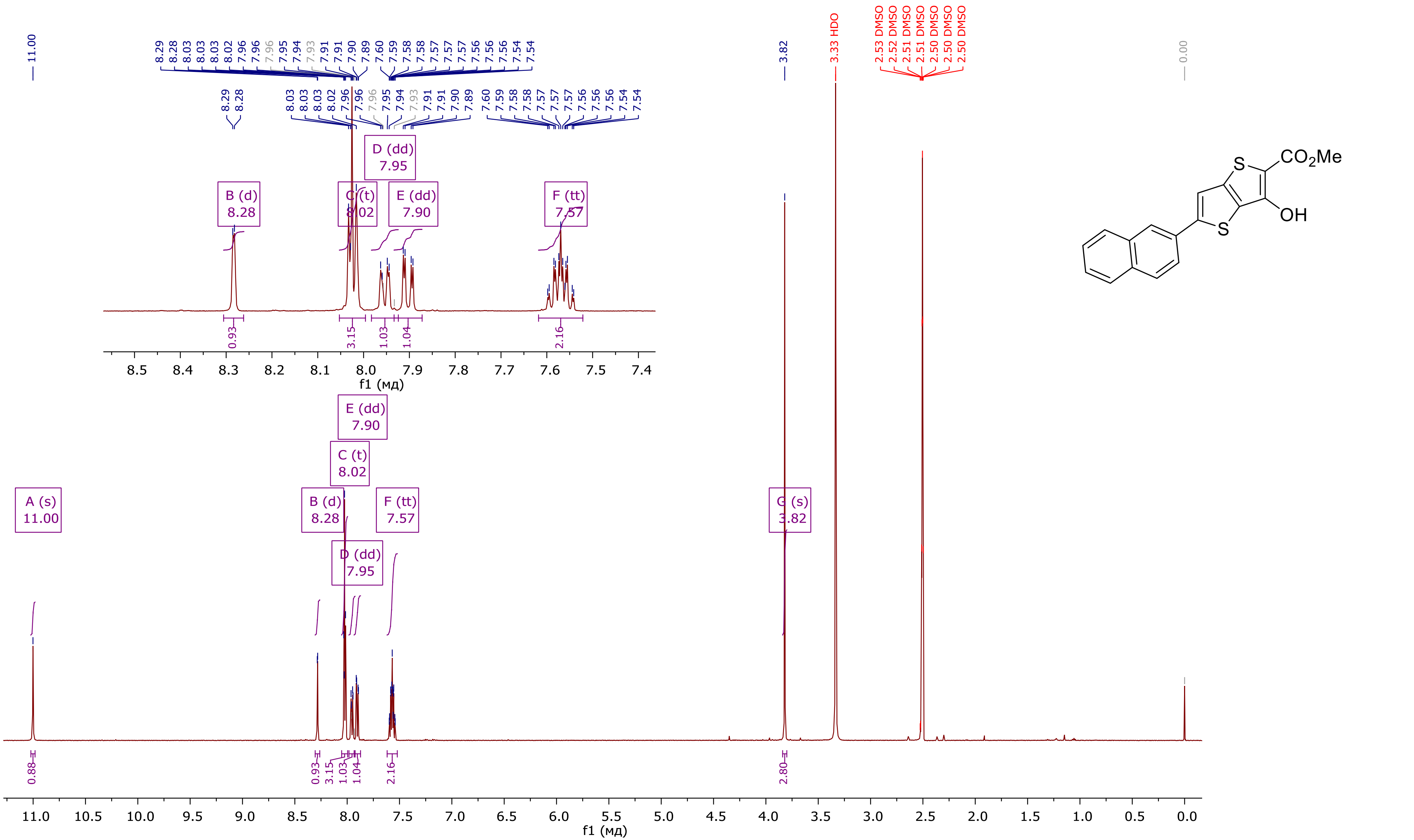

^{1}H NMR (500 MHz, DMSO-d_6) δ 11.00 (s, 1H), 8.28 (d, *J* = 1.8 Hz, 1H), 8.02 (t, *J* = 4.2 Hz, 3H), 7.95 (dd, *J* = 7.4, 1.9 Hz, 1H), 7.90 (dd, *J* = 8.7, 1.9 Hz, 1H), 7.57 (tt, *J* = 6.9, 5.2 Hz, 2H), 3.82 (s, 3H).

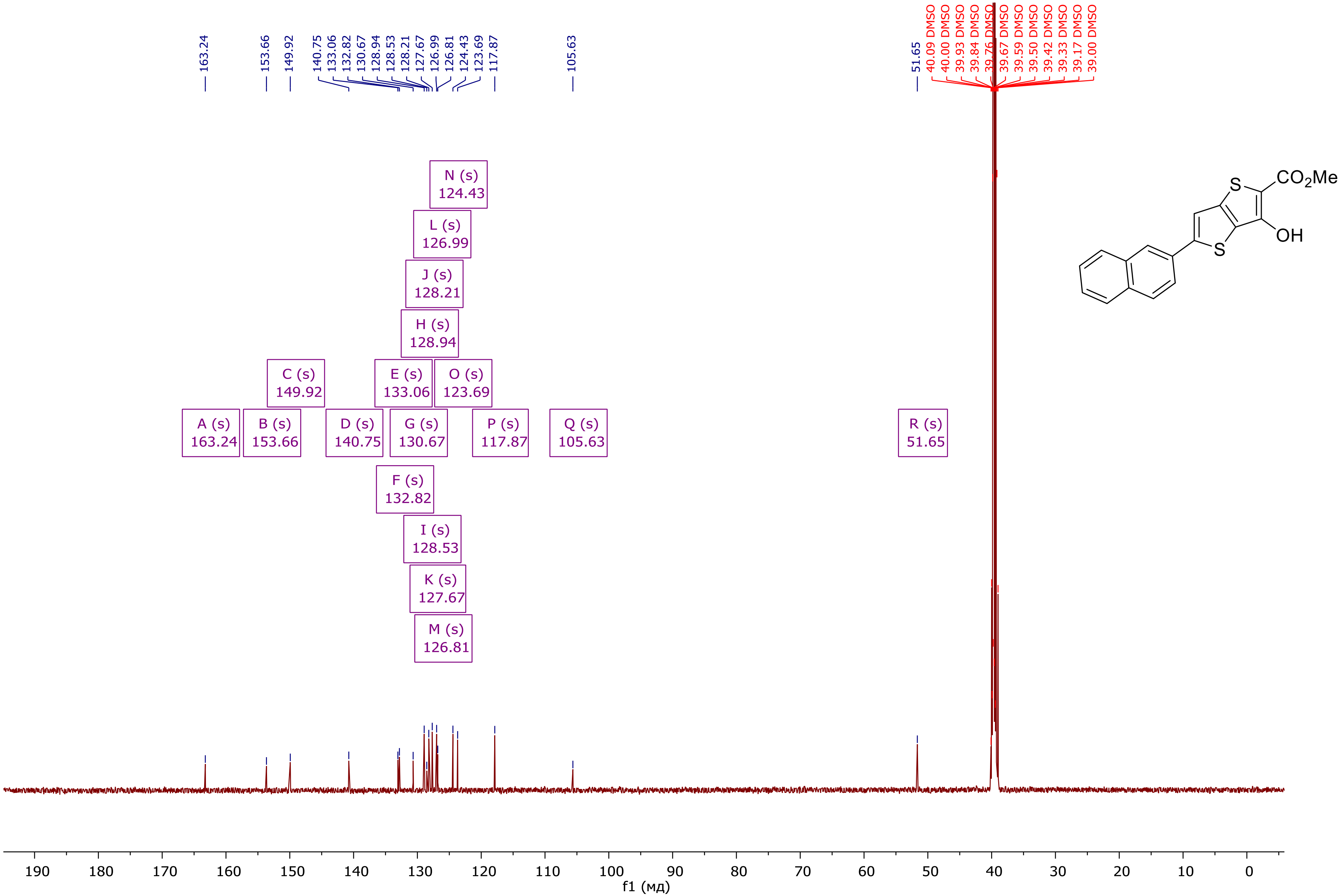

^{13}C NMR (126 MHz, DMSO-d_6) δ 163.2, 153.7, 149.9, 140.7, 133.1, 132.8, 130.7, 128.9, 128.5, 128.2, 127.7, 127.0, 126.8, 124.4, 123.7, 117.9, 105.6, 51.6.

Methyl 3-hydroxy-6-phenylthieno[3,2-*b*]thiophene-2-carboxylate (3k)

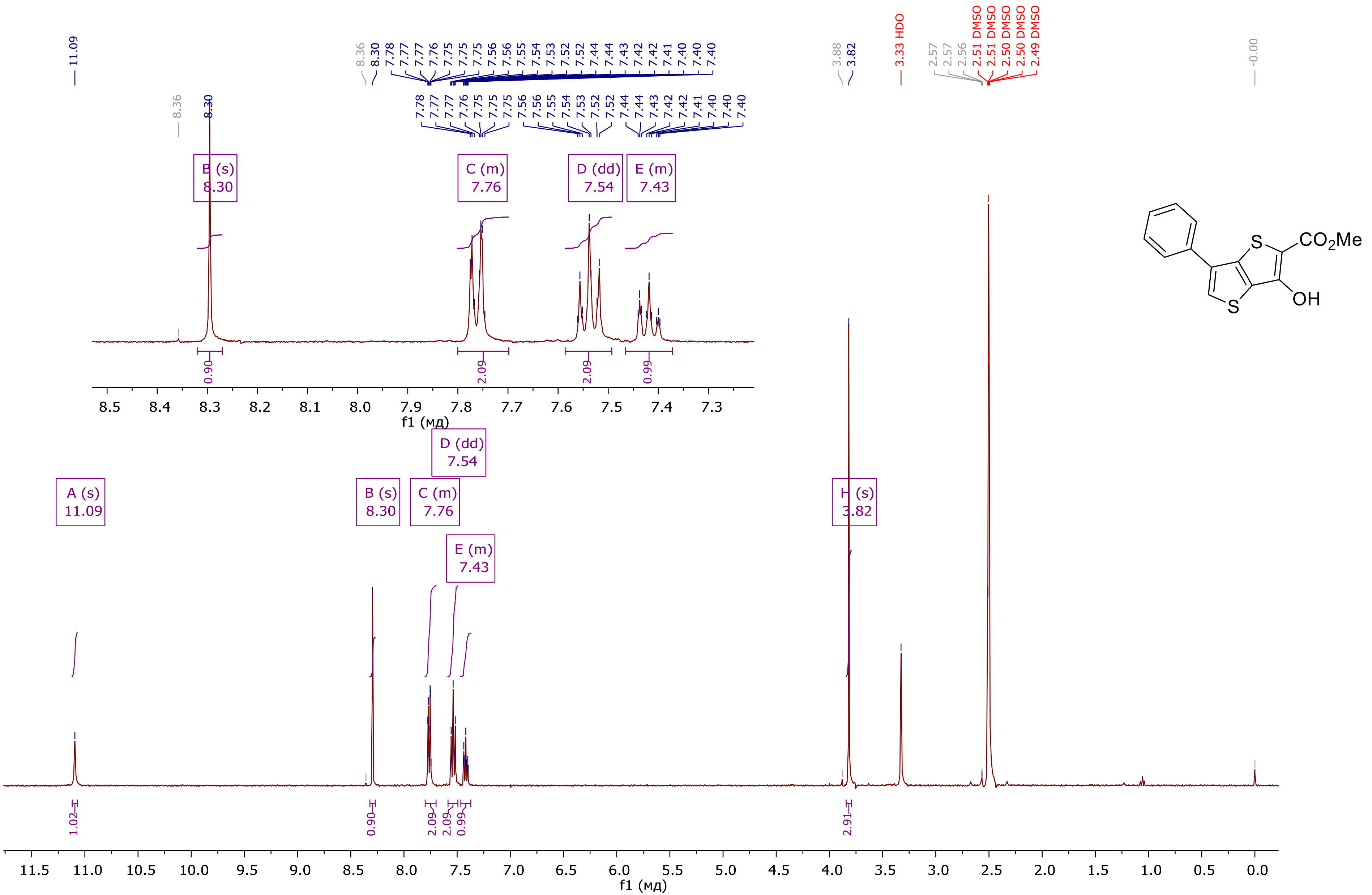

^{1}H NMR (400 MHz, DMSO-d_6) δ 11.09 (s, 1H), 8.30 (s, 1H), 7.80 – 7.70 (m, 2H), 7.54 (dd, *J* = 8.4, 7.0 Hz, 2H), 7.47 – 7.37 (m, 1H), 3.82 (s, 3H).

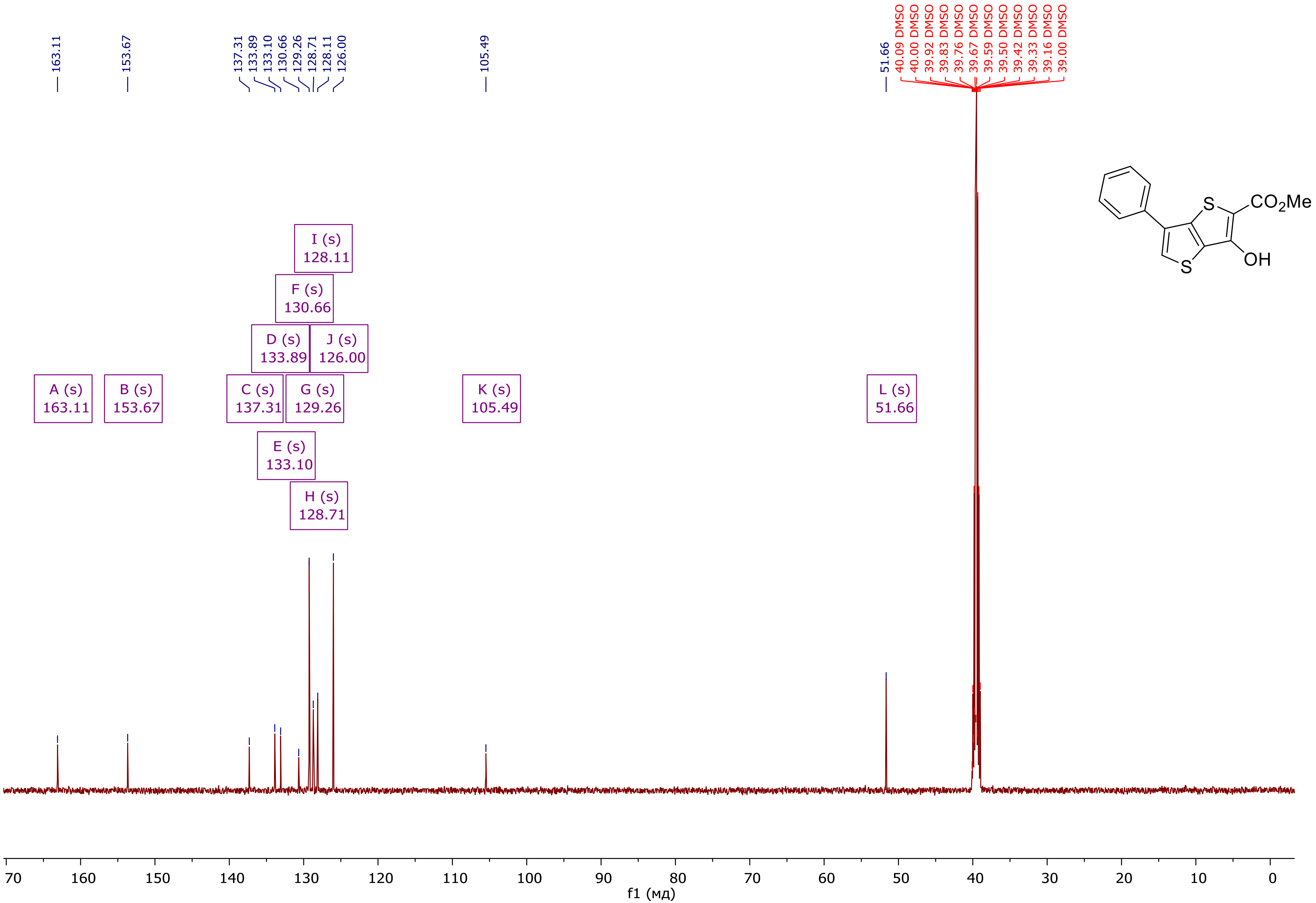

^{13}C NMR (126 MHz, DMSO-d_6) δ 163.1, 153.7, 137.3, 133.9, 133.1, 130.7, 129.3, 128.7, 128.1, 126.0, 105.5, 51.7.

5-Phenylthieno[3,2-*b*]thiophen-3(2*H*)-one (4a)

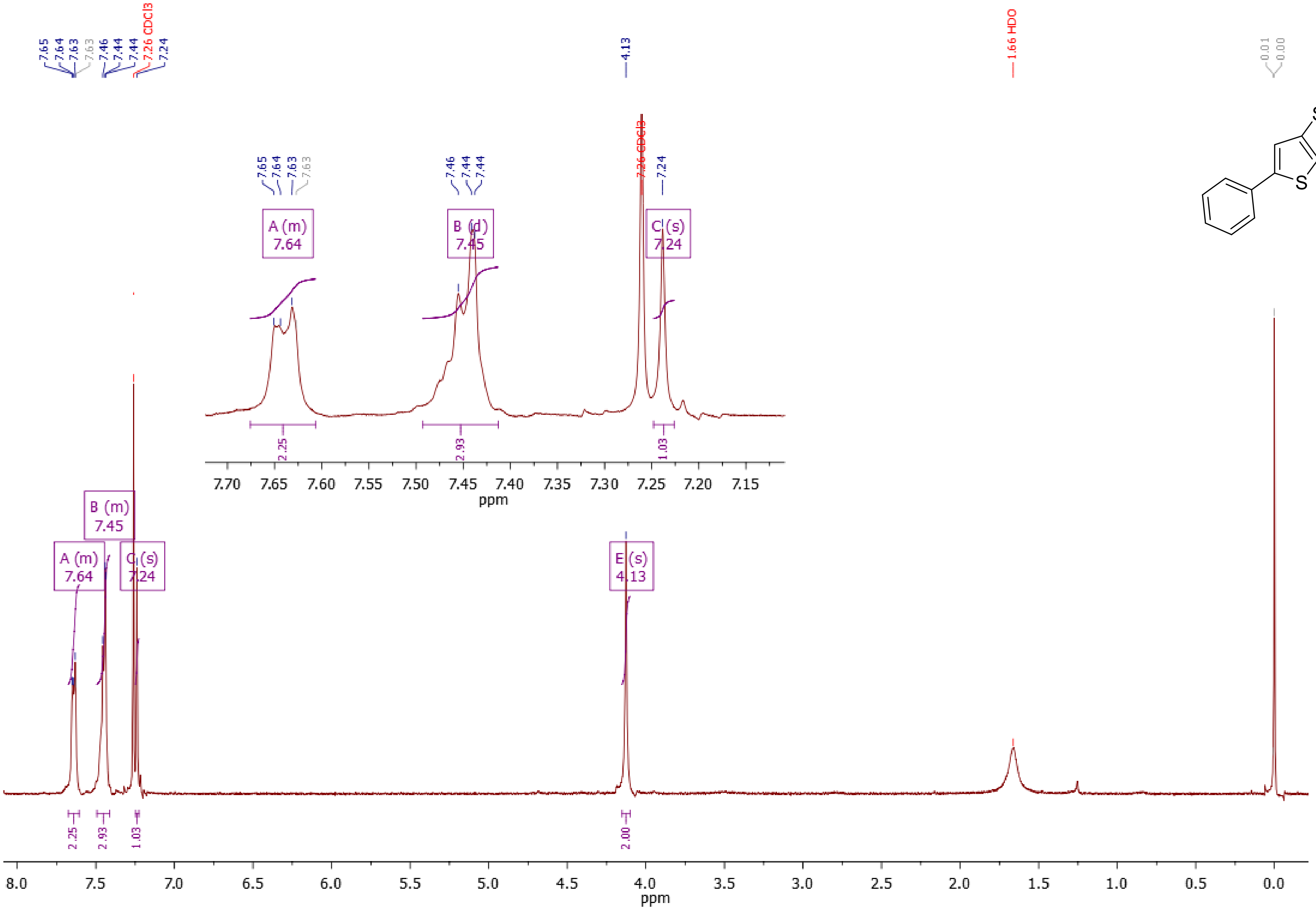

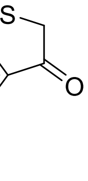

^{1}H NMR (400 MHz, chloroform-*d*) δ 7.68 – 7.61 (m, 2H), 7.49 – 7.41 (m, 3H), 7.24 (s, 1H), 4.13 (s, 2H).

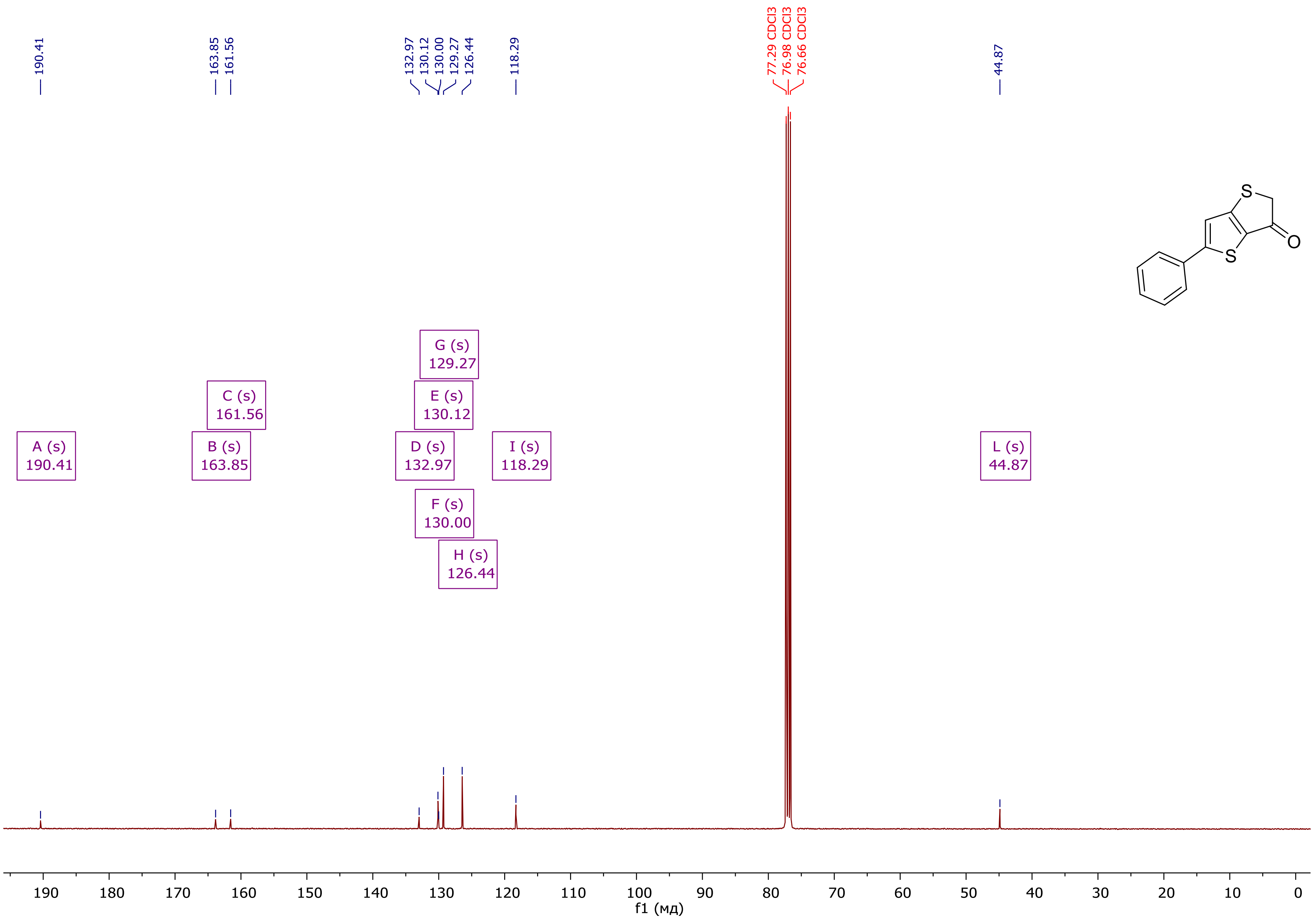

^{13}C NMR (101 MHz, chloroform-*d*) δ 190.4, 163.8, 161.6, 133.0, 130.1, 130.0, 129.3, 126.4, 118.3, 44.9.

5-(*p*-Tolyl)thieno[3,2-*b*]thiophen-3(2*H*)-one (4b)

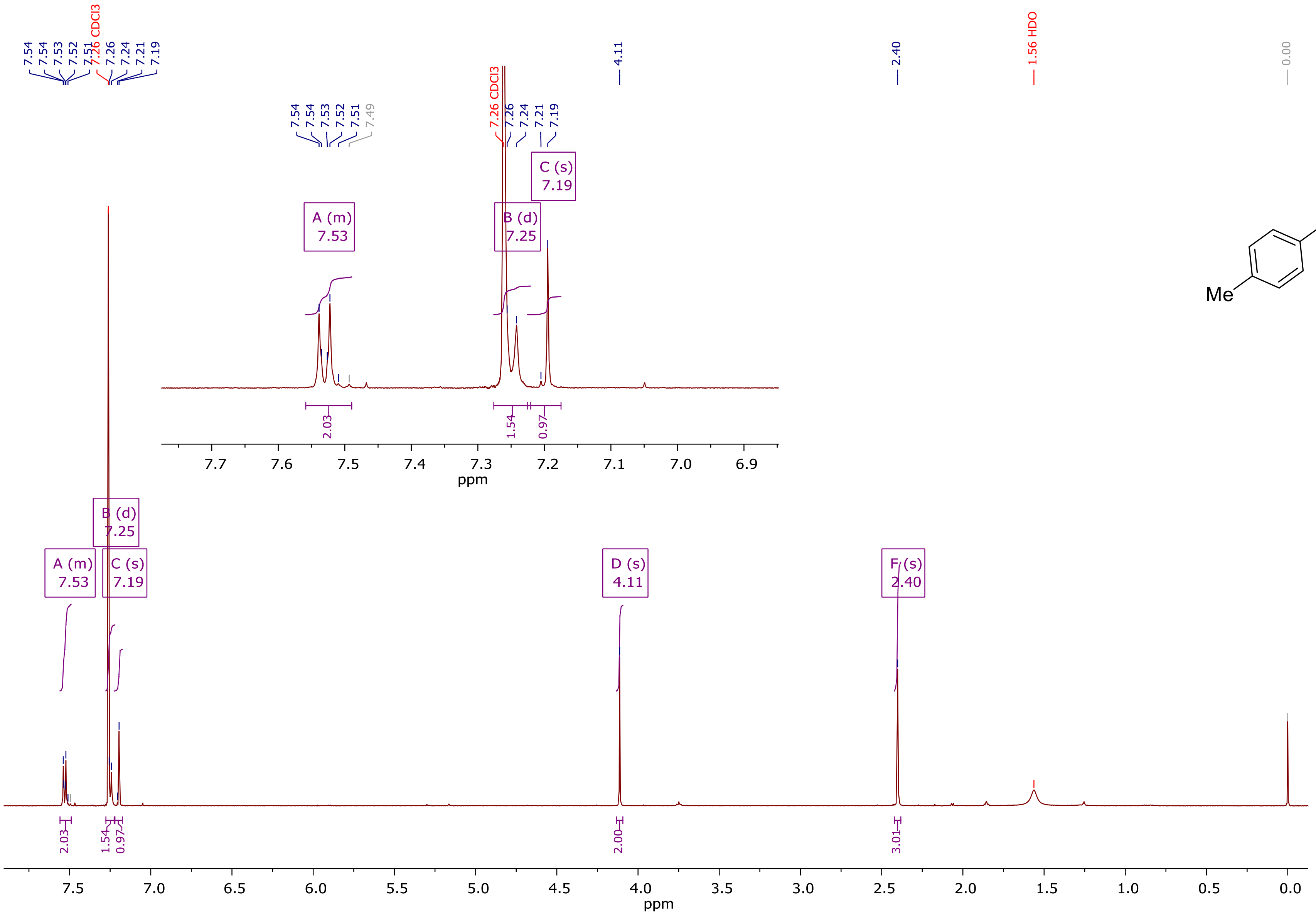

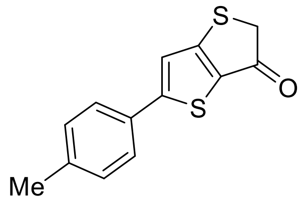

^{1}H NMR (500 MHz, chloroform-*d*) δ 7.56 – 7.49 (m, 2H), 7.25 (d, *J* = 7.0 Hz, 2H), 7.19 (s, 1H), 4.11 (s, 2H), 2.40 (s, 3H).

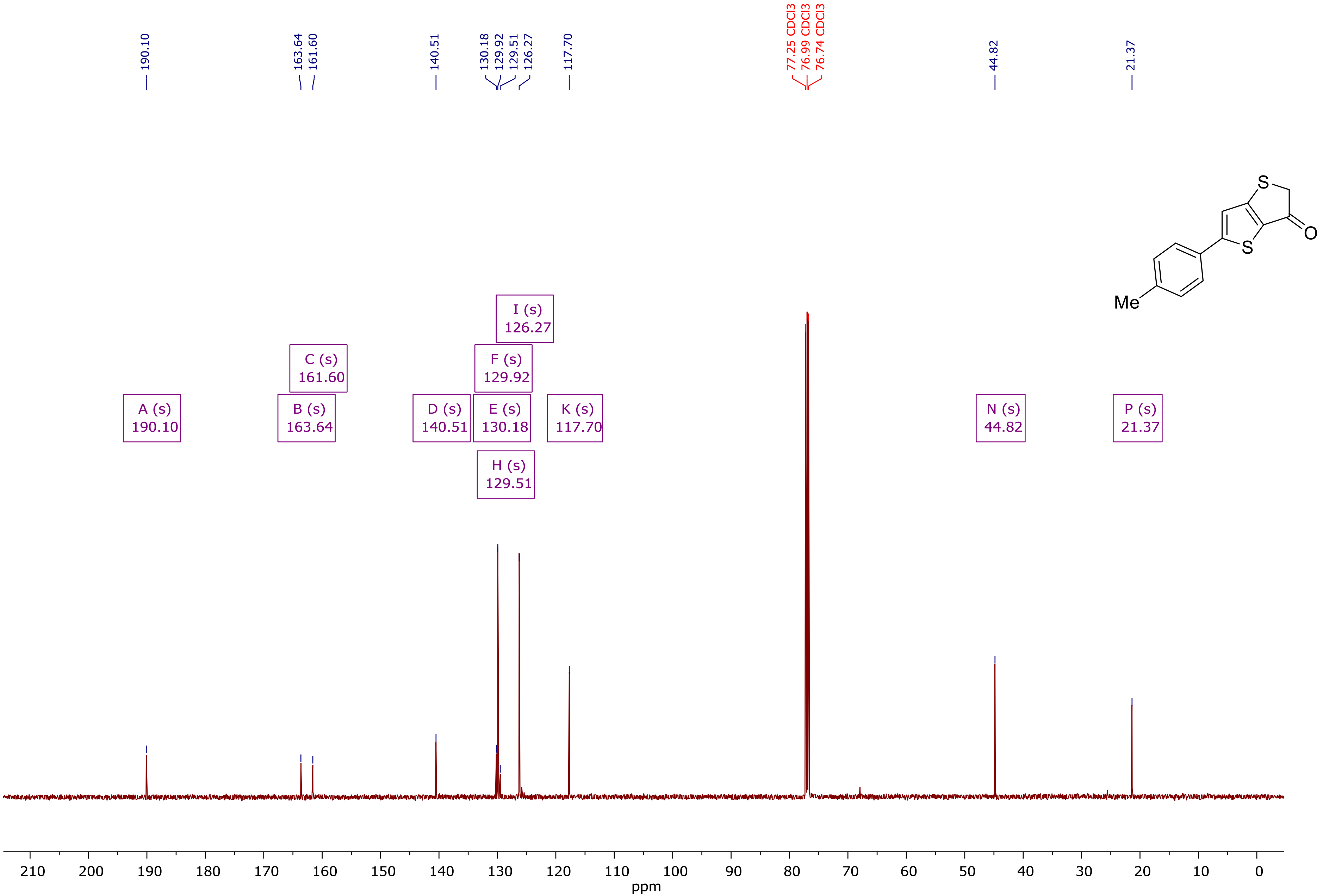

^{13}C NMR (126 MHz, chloroform-*d*) δ 190.1, 163.6, 161.6, 140.5, 130.2, 129.9, 129.5, 126.3, 117.7, 44.8, 21.4.

5-(2,5-Dimethylphenyl)-thieno[3,2-*b*]thiophen-3(2*H*)-one (4c)

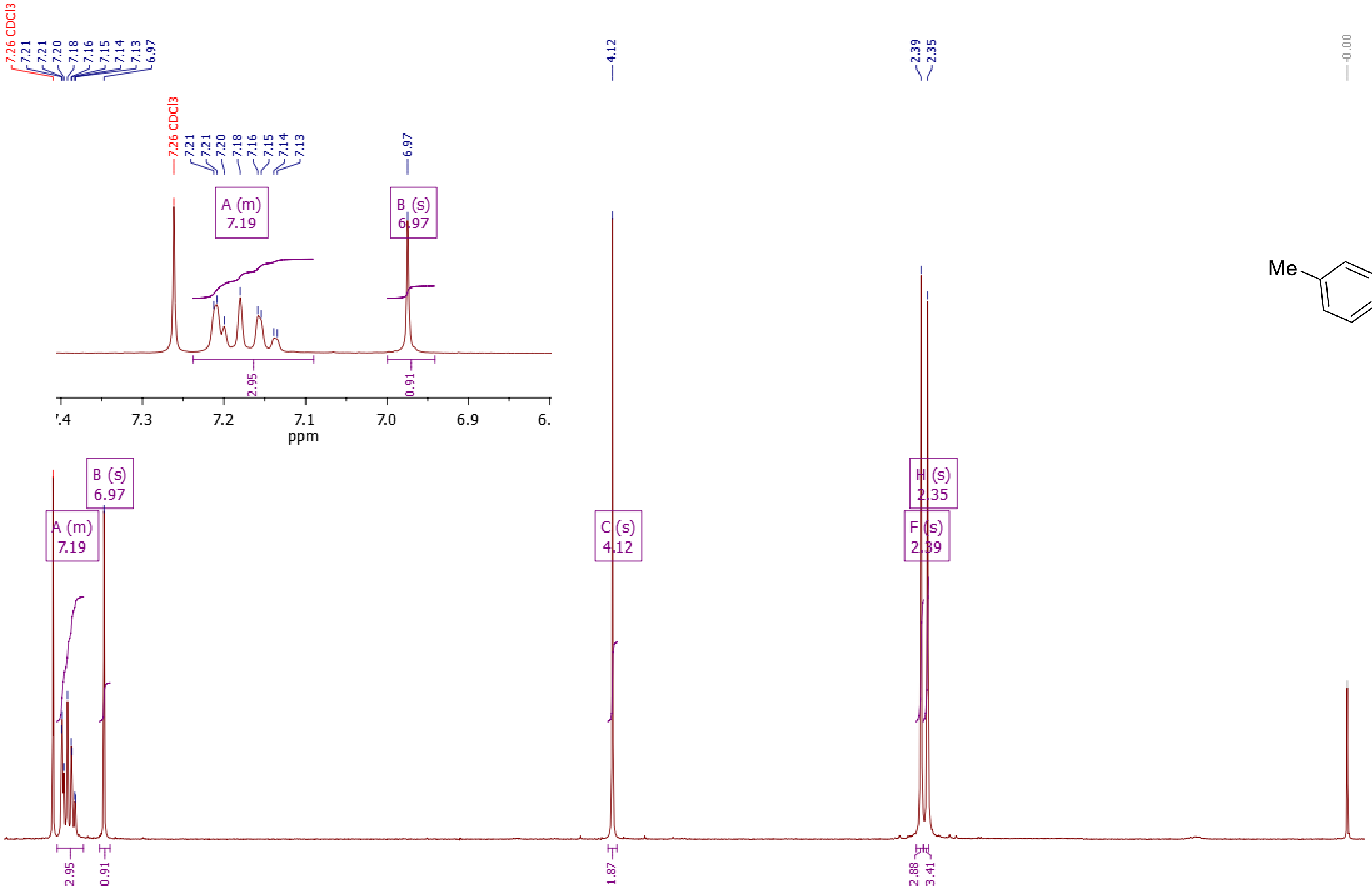

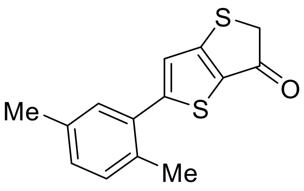

^{1}H NMR (400 MHz, chloroform-*d*) δ 7.24 – 7.09 (m, 3H), 6.97 (s, 1H), 4.12 (s, 2H), 2.39 (s, 3H), 2.35 (s, 3H).

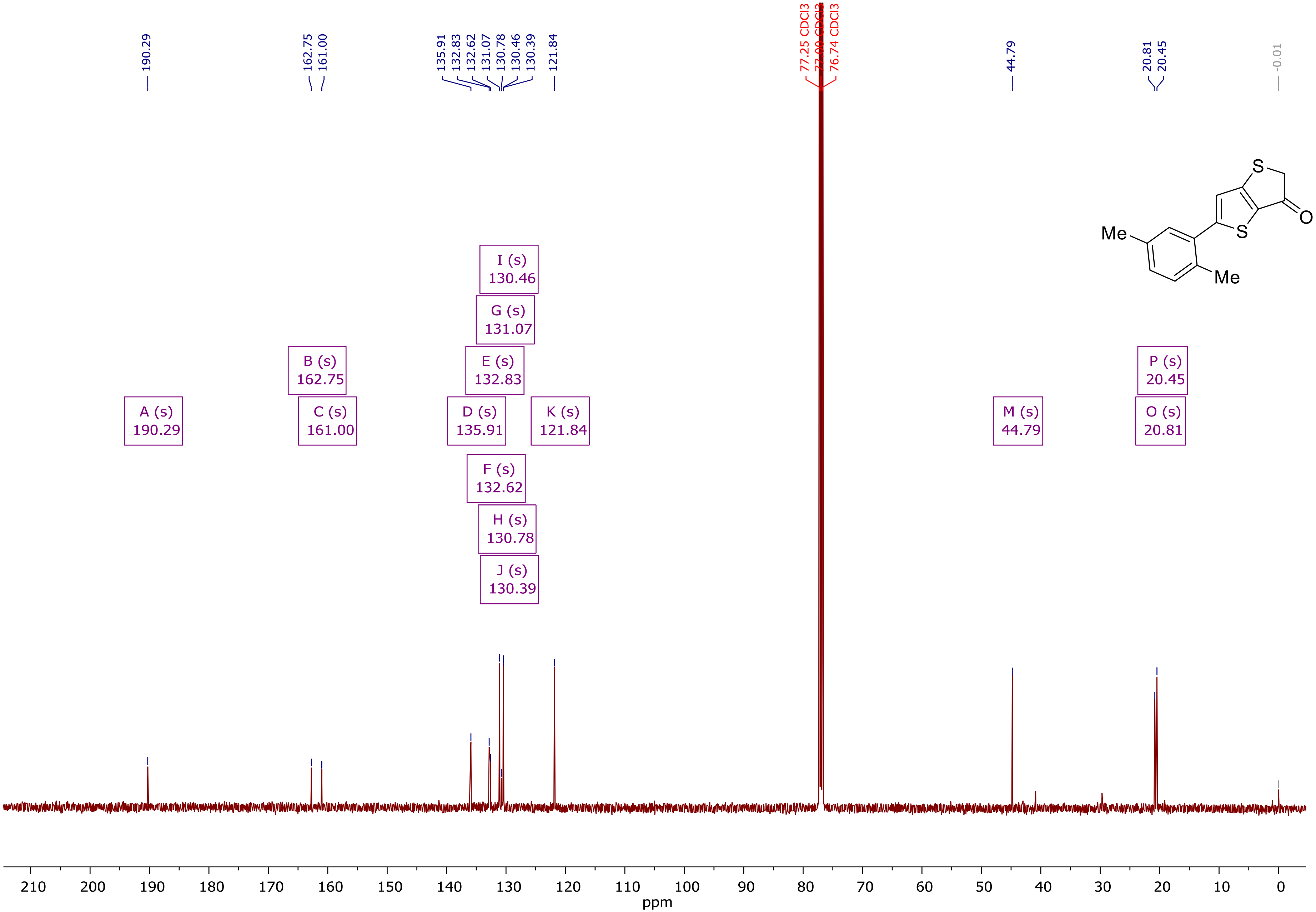

^{13}C NMR (126 MHz, chloroform-*d*) δ 190.3, 162.7, 161.0, 135.9, 132.8, 132.6, 131.1, 130.8, 130.5, 130.4, 121.8, 44.8, 20.8, 20.4.

5-(4-(*Tert*-butyl)phenyl)-thieno[3,2-*b*]thiophen-3(2*H*)-one (4d)

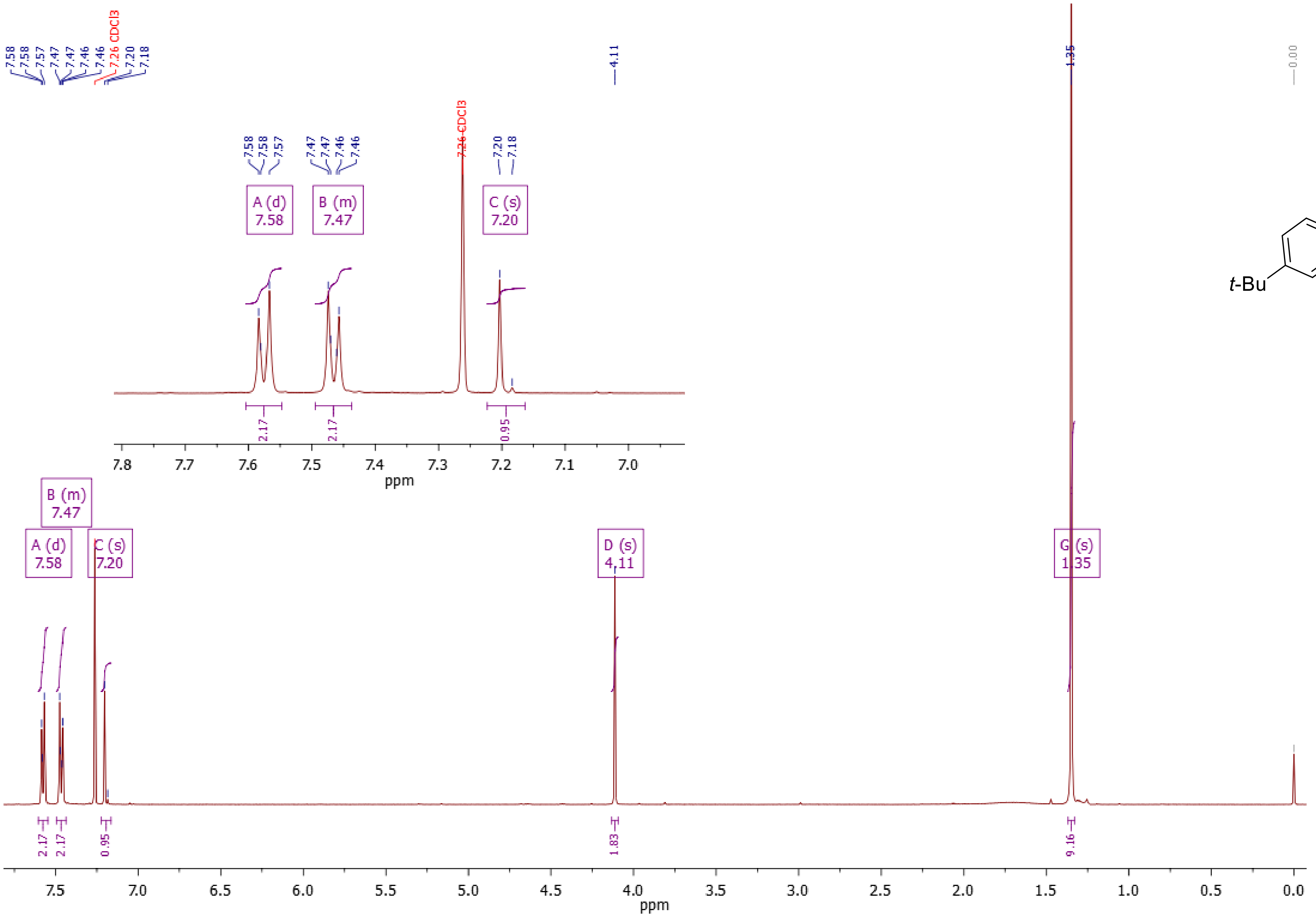

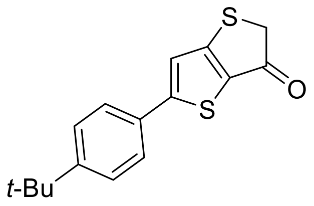

^{1}H NMR (500 MHz, chloroform-*d*) δ 7.58 (d, *J* = 8.4 Hz, 2H), 7.49 – 7.44 (m, 2H), 7.20 (s, 1H), 4.11 (s, 2H), 1.35 (s, 9H).

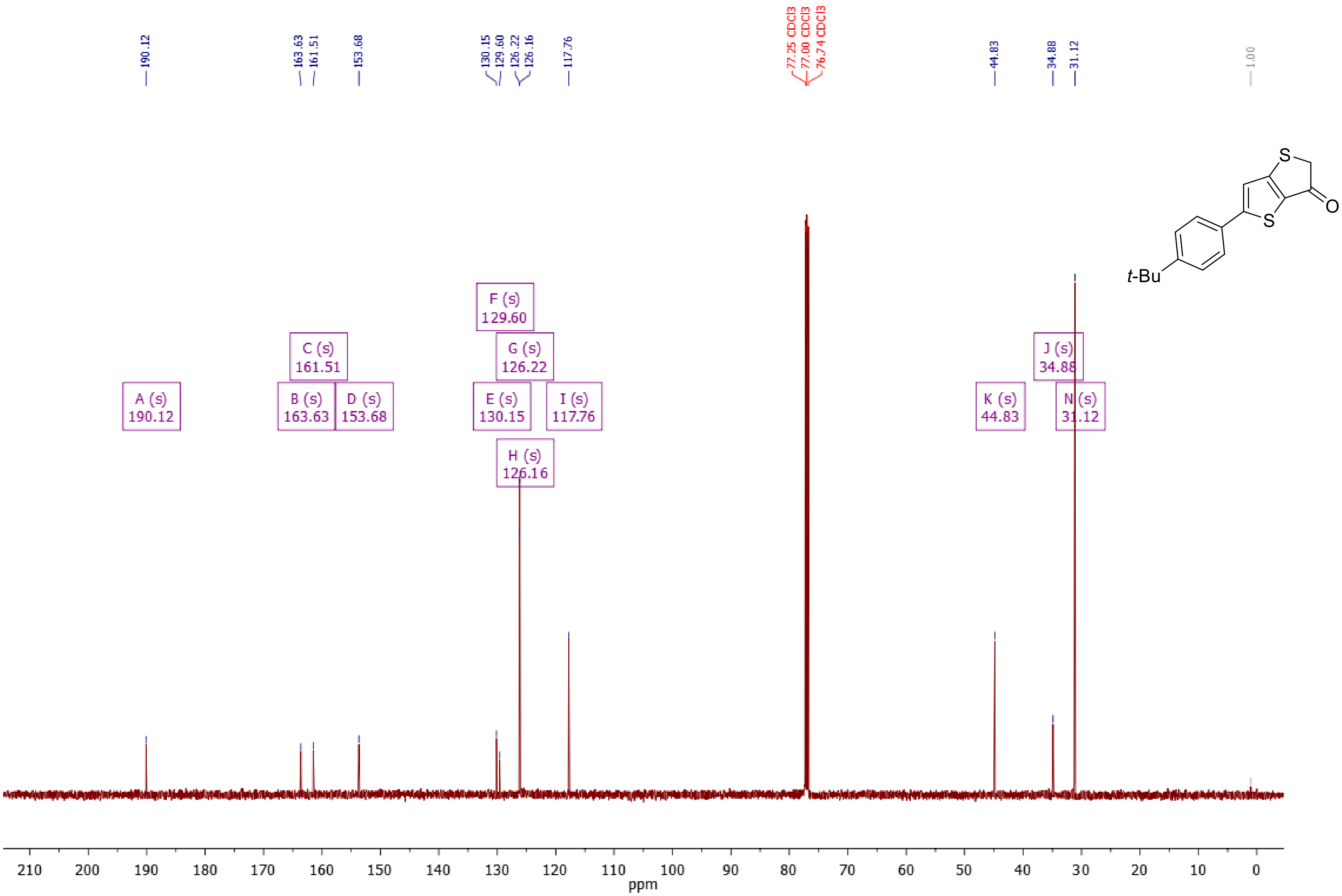

^{13}C NMR (126 MHz, chloroform-*d*) δ 190.1, 163.6, 161.5, 153.7, 130.1, 129.6, 126.2, 126.2, 117.8, 44.8, 34.9, 31.1.

5-(4-Fluorophenyl)-thieno[3,2-*b*]thiophen-3(2*H*)-one (4e)

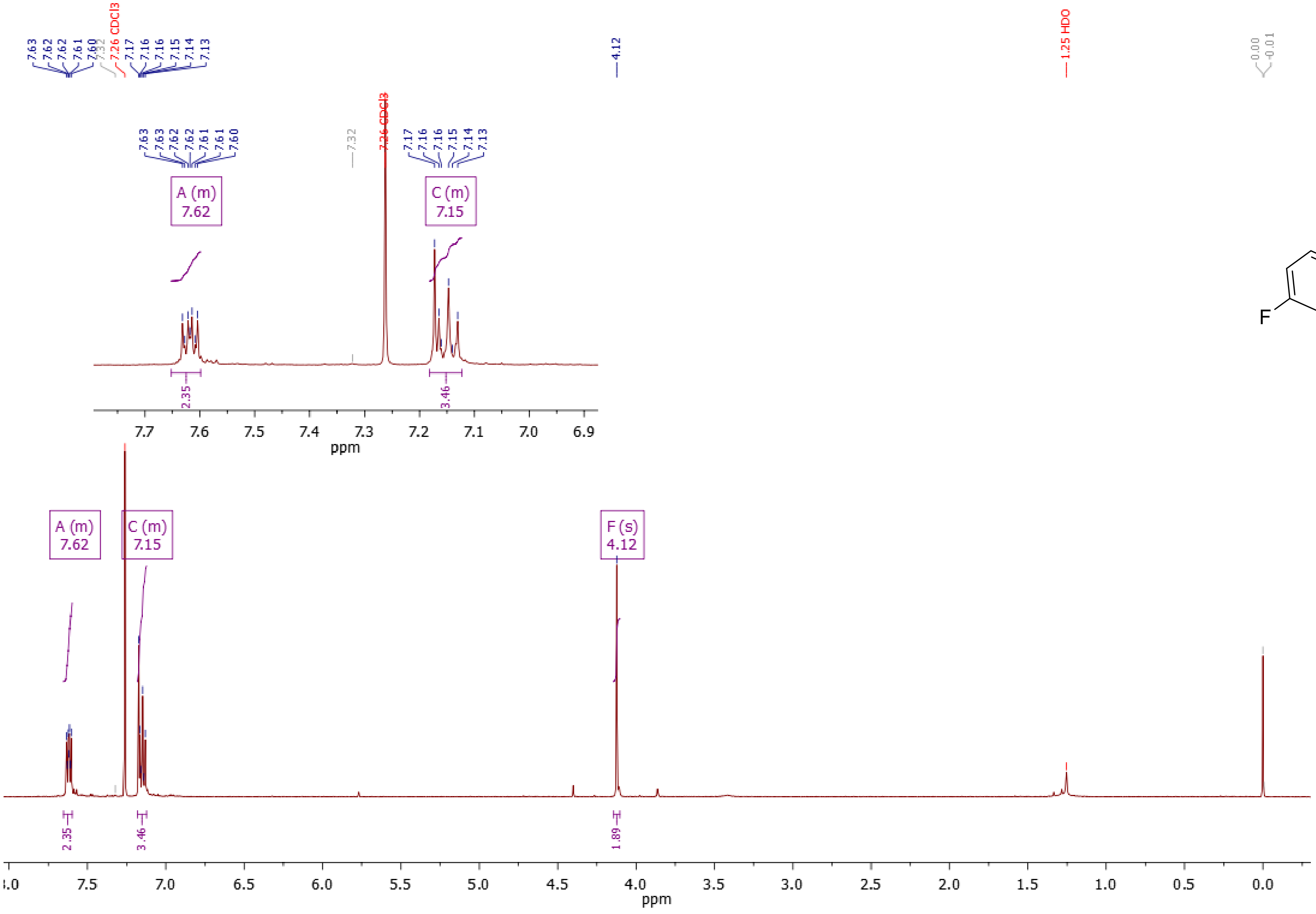

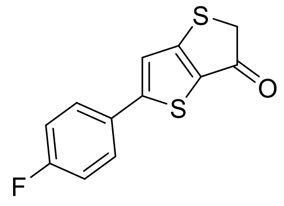

^{1}H NMR (500 MHz, chloroform-*d*) δ 7.65 – 7.60 (m, 2H), 7.18 – 7.12 (m, 20H), 4.12 (s, 2H).

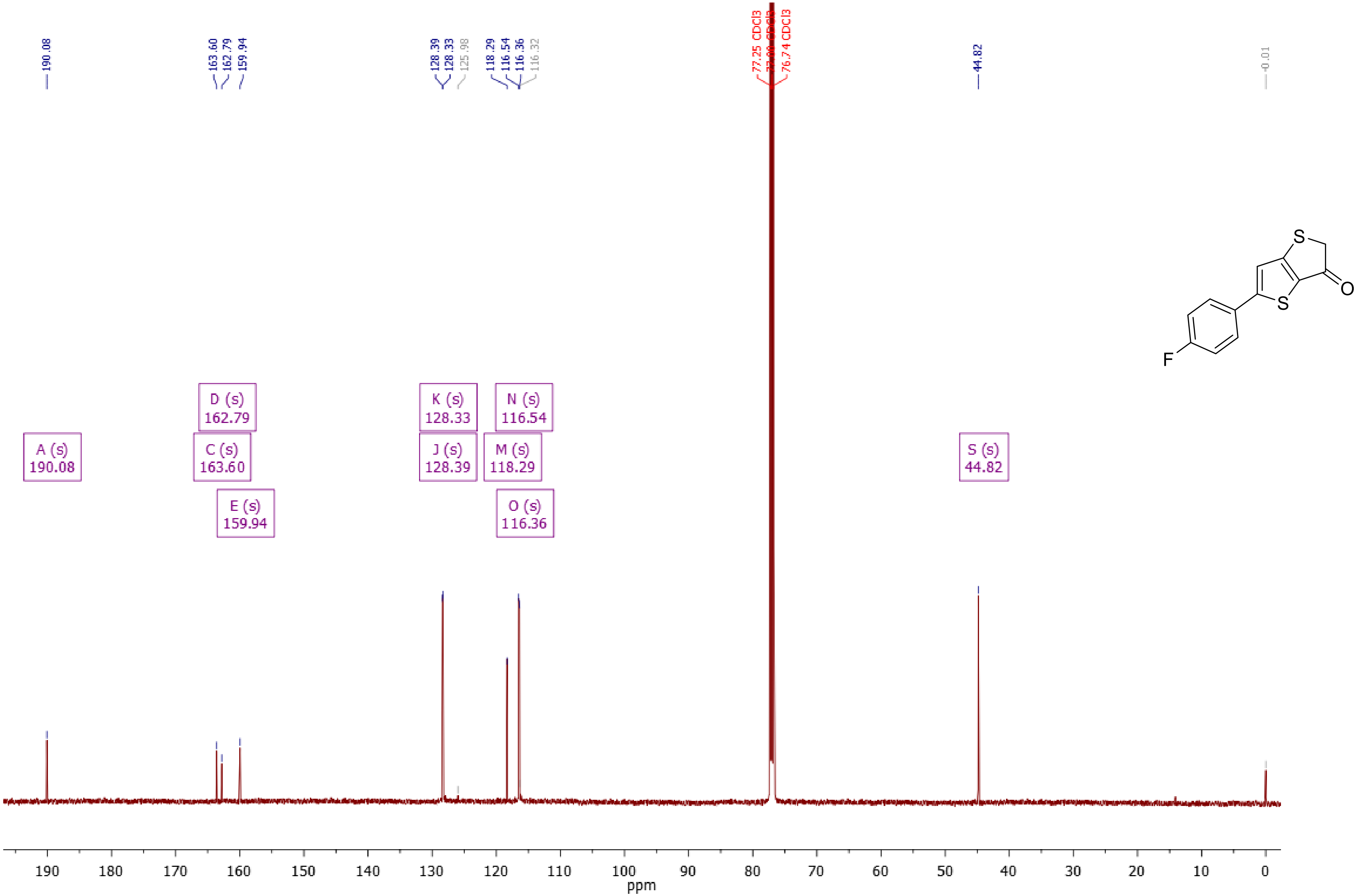

^{13}C NMR (126 MHz, chloroform-*d*) δ 190.1, 163.6, 162.8, 159.9, 128.4, 128.3, 118.3, 116.5, 116.4, 44.8.

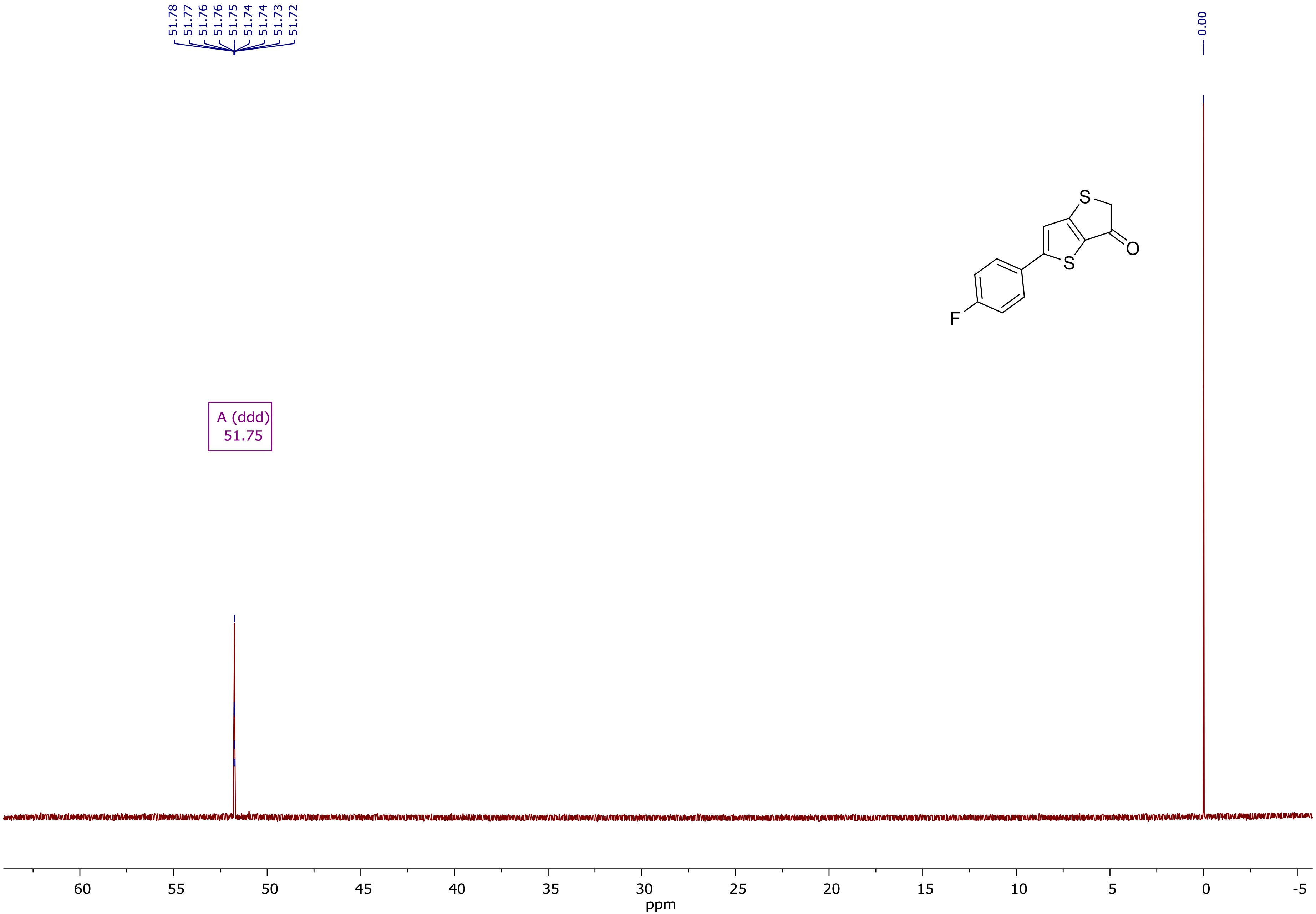

^{19}F NMR (471 MHz, chloroform-*d*) δ 51.75 (ddd, *J* = 8.3, 5.2, 3.2 Hz).

5-(4-Chlorophenyl)-thieno[3,2-*b*]thiophen-3(2*H*)-one (4f)

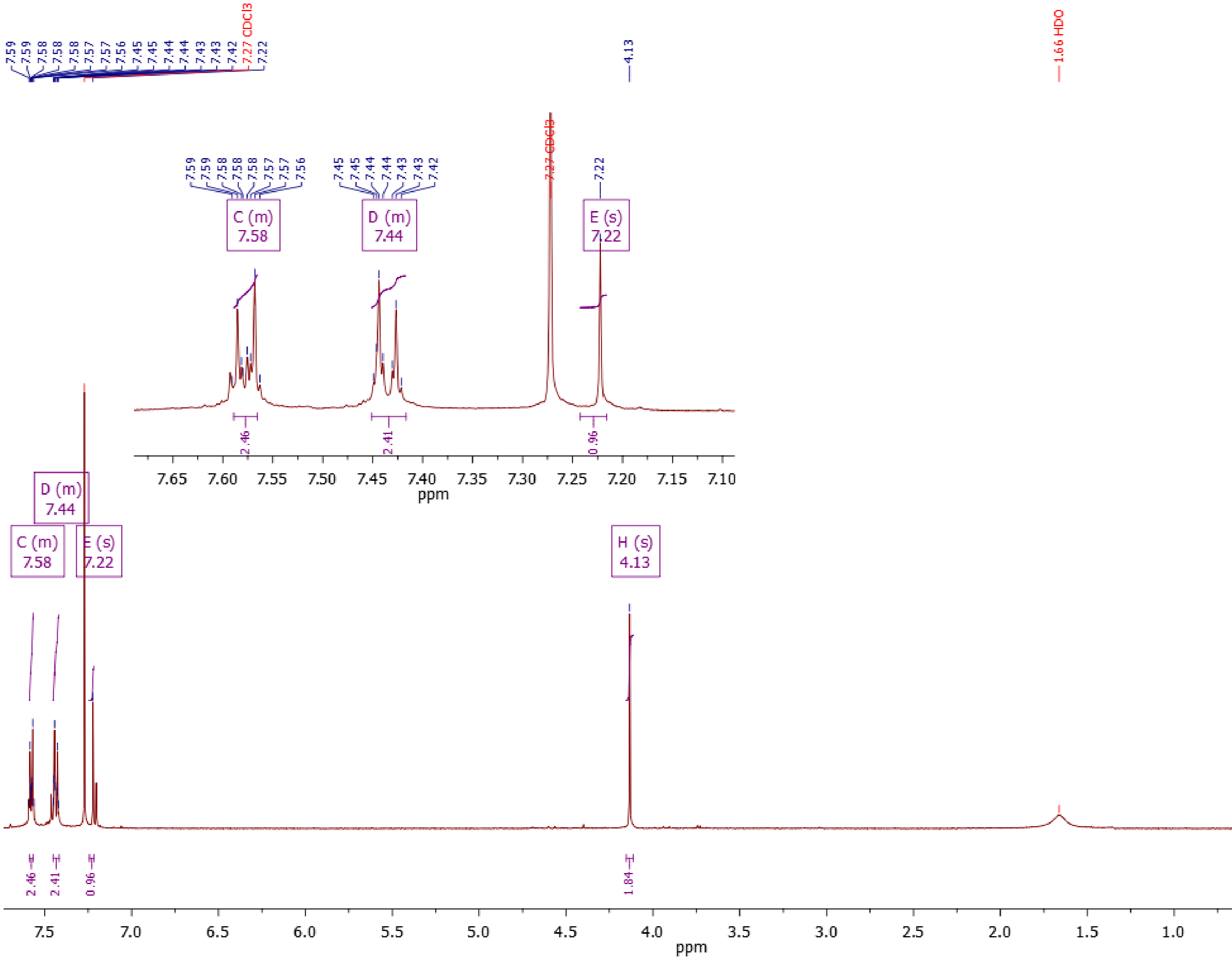

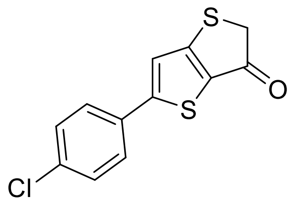

^{1}H NMR (500 MHz, chloroform-*d*) δ 7.59 – 7.57 (m, 2H), 7.45 – 7.42 (m, 2H), 7.22 (s, 1H), 4.13 (s, 2H).

5-(4-Bromophenyl)-thieno[3,2-*b*]thiophen-3(2*H*)-one (4g)

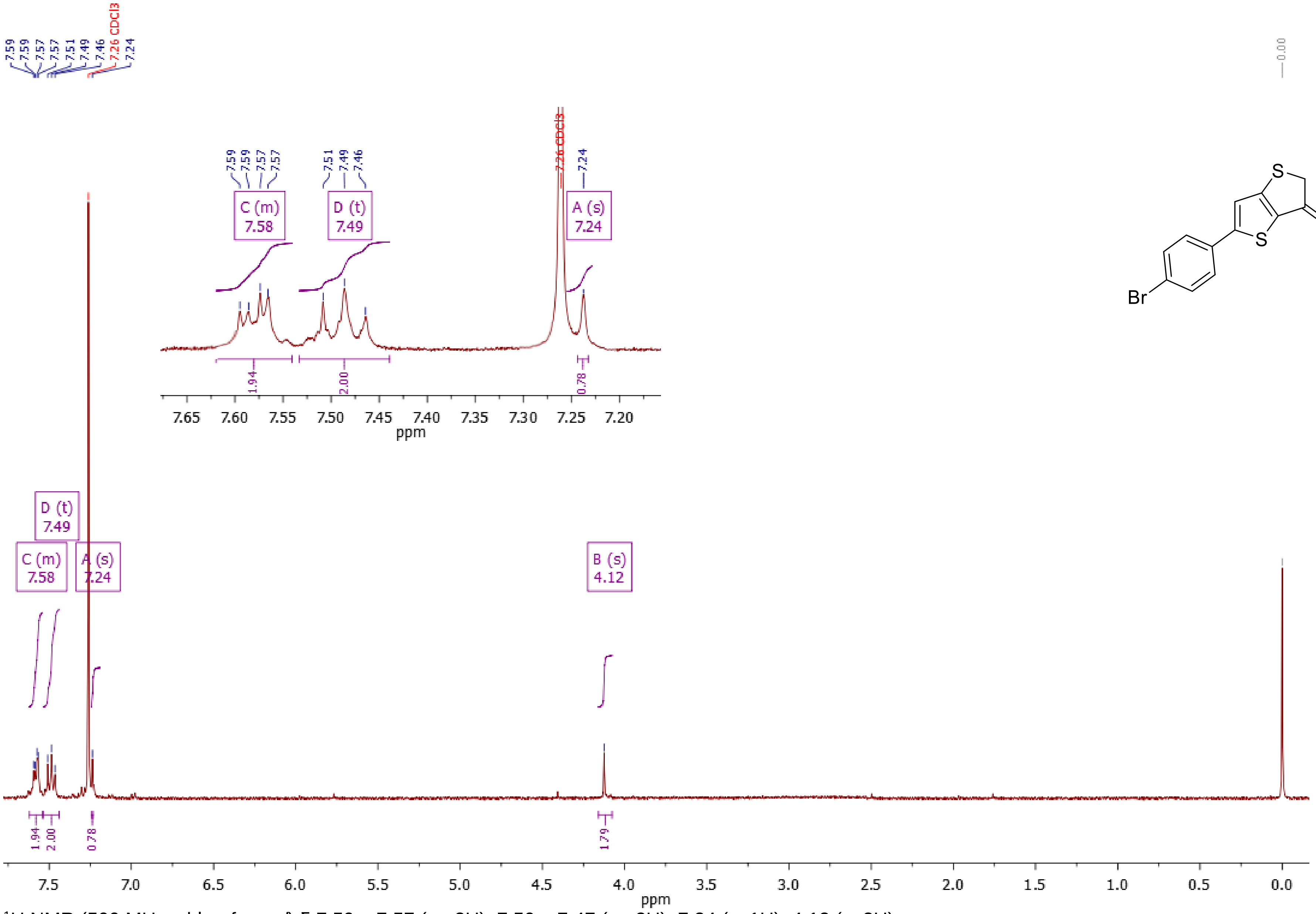

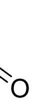

^{1}H NMR (500 MHz, chloroform-*d*) δ 7.59 – 7.57 (m, 2H), 7.50 – 7.47 (m, 2H), 7.24 (s, 1H), 4.12 (s, 2H).

5-(3-Methoxyphenyl)thieno[3,2-*b*]thiophen-3(2*H*)-one (4h)

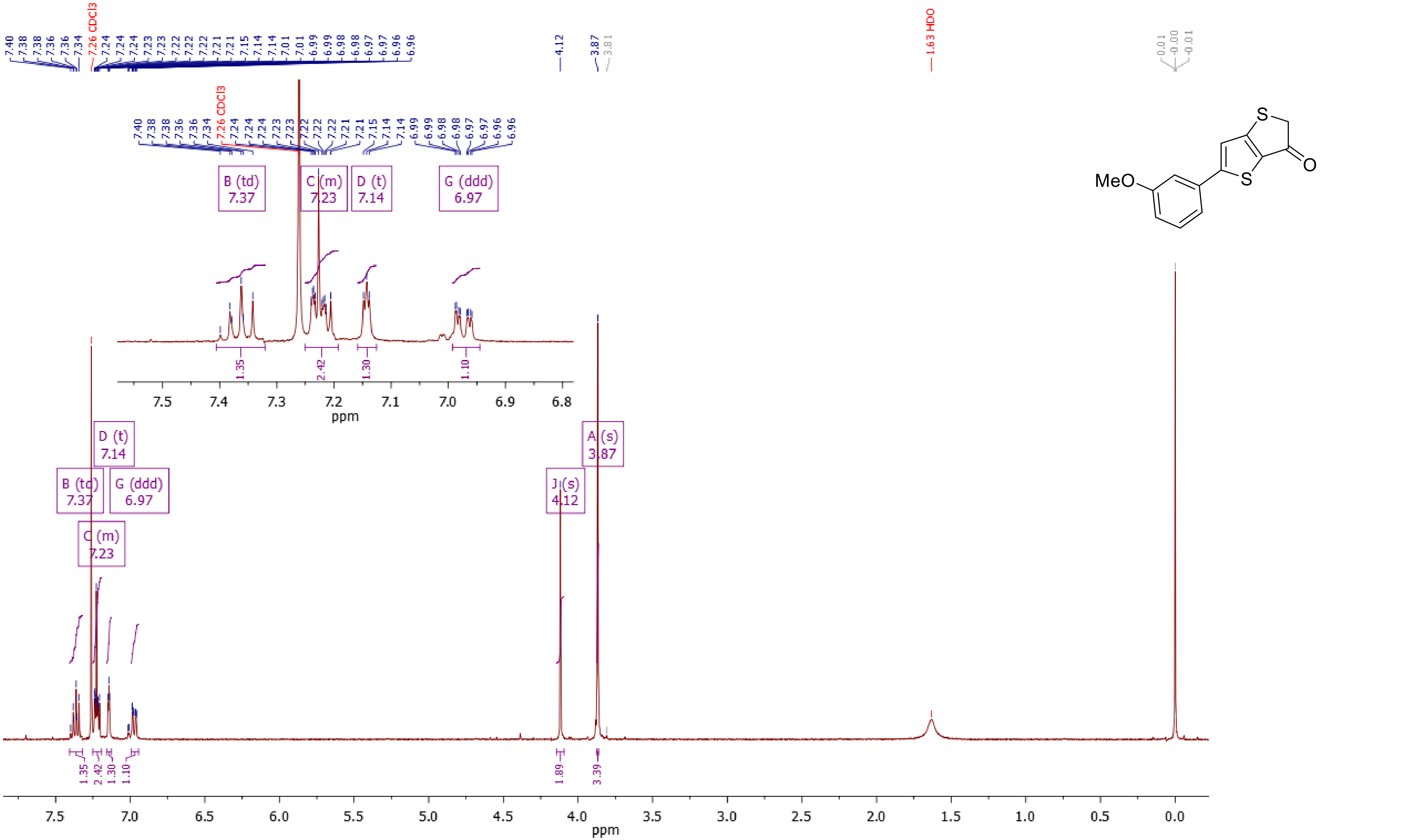

^{1}H NMR (400 MHz, chloroform-*d*) δ 7.37 (td, *J* = 8.1, 6.7 Hz, 1H), 7.25 – 7.19 (m, 2H), 7.14 (t, *J* = 2.2 Hz, 1H), 6.97 (ddd, *J* = 8.3, 2.6, 0.9 Hz, 1H), 4.12 (s, 2H), 3.87 (s, 3H).

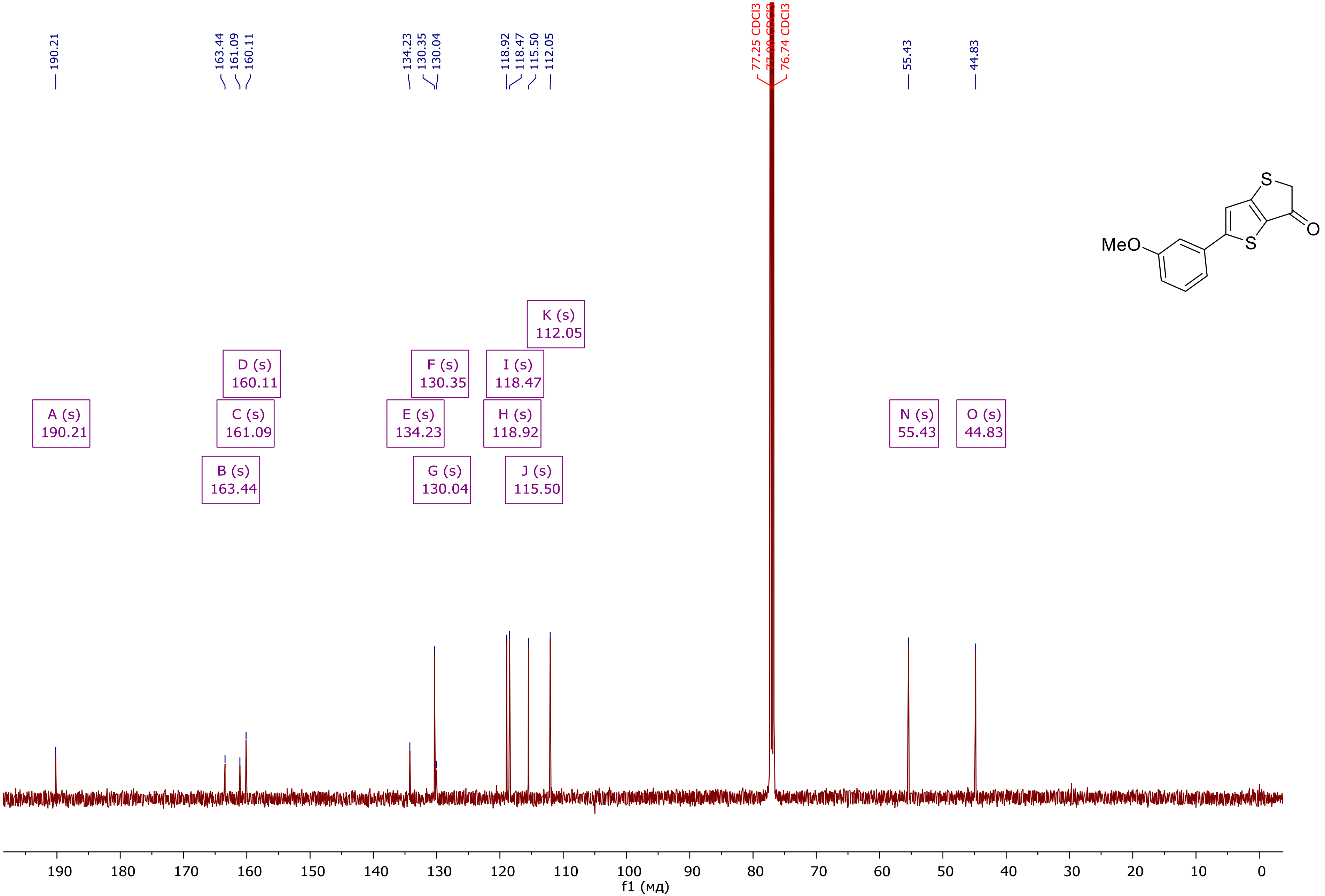

^{13}C NMR (126 MHz, chloroform-*d*) δ 190.2, 163.4, 161.1, 160.1, 134.2, 130.3, 130.0, 118.9, 118.5, 115.5, 112.0, 55.4, 44.8.

5-(4-Ethoxyphenyl)-thieno[3,2-*b*]thiophen-3(2*H*)-one (4i)

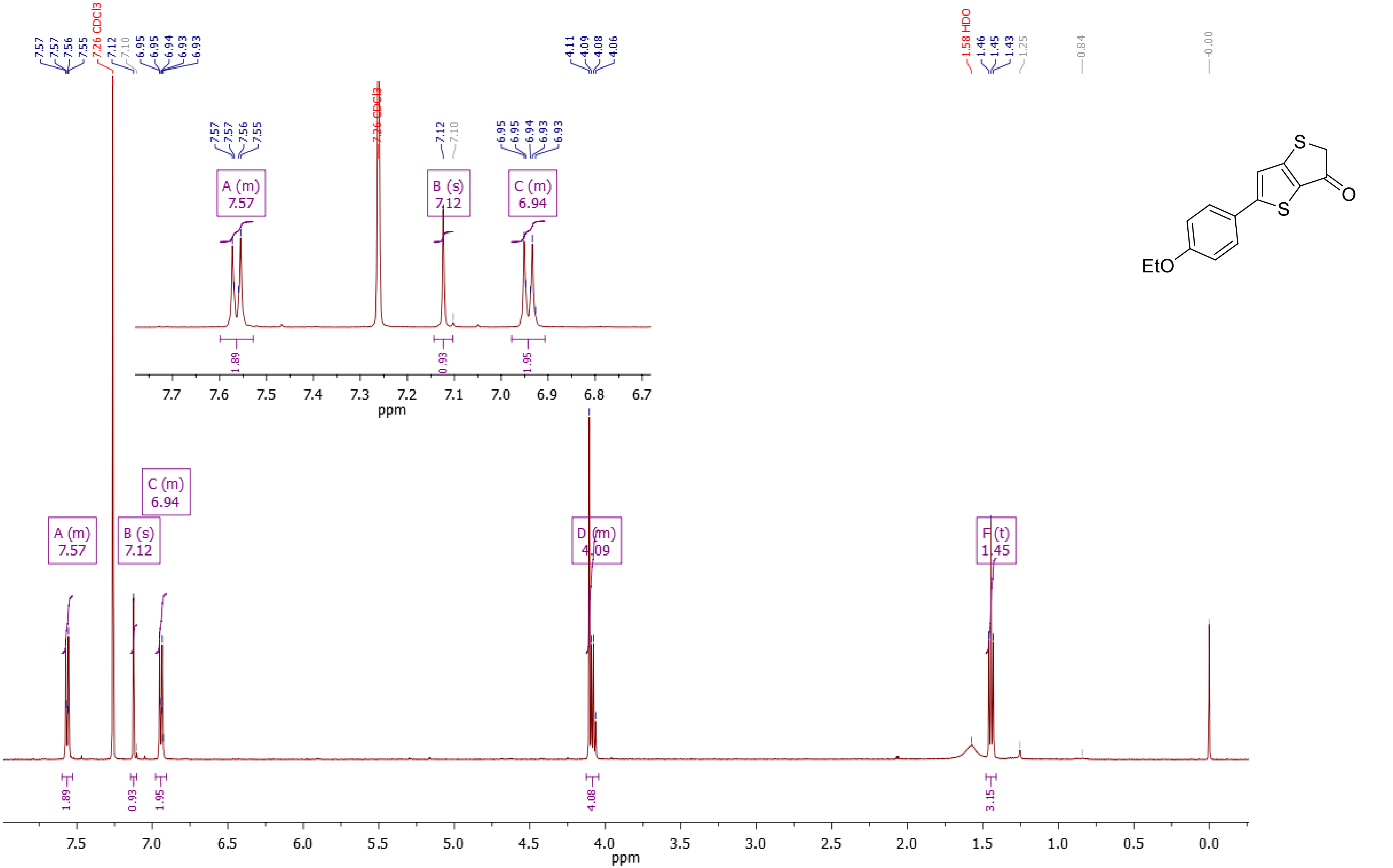

^{1}H NMR (500 MHz, chloroform-*d*) δ 7.60 – 7.53 (m, 2H), 7.12 (s, 1H), 6.98 – 6.91 (m, 2H), 4.13 – 4.04 (m, 4H), 1.45 (t, *J* = 7.0 Hz, 3H).

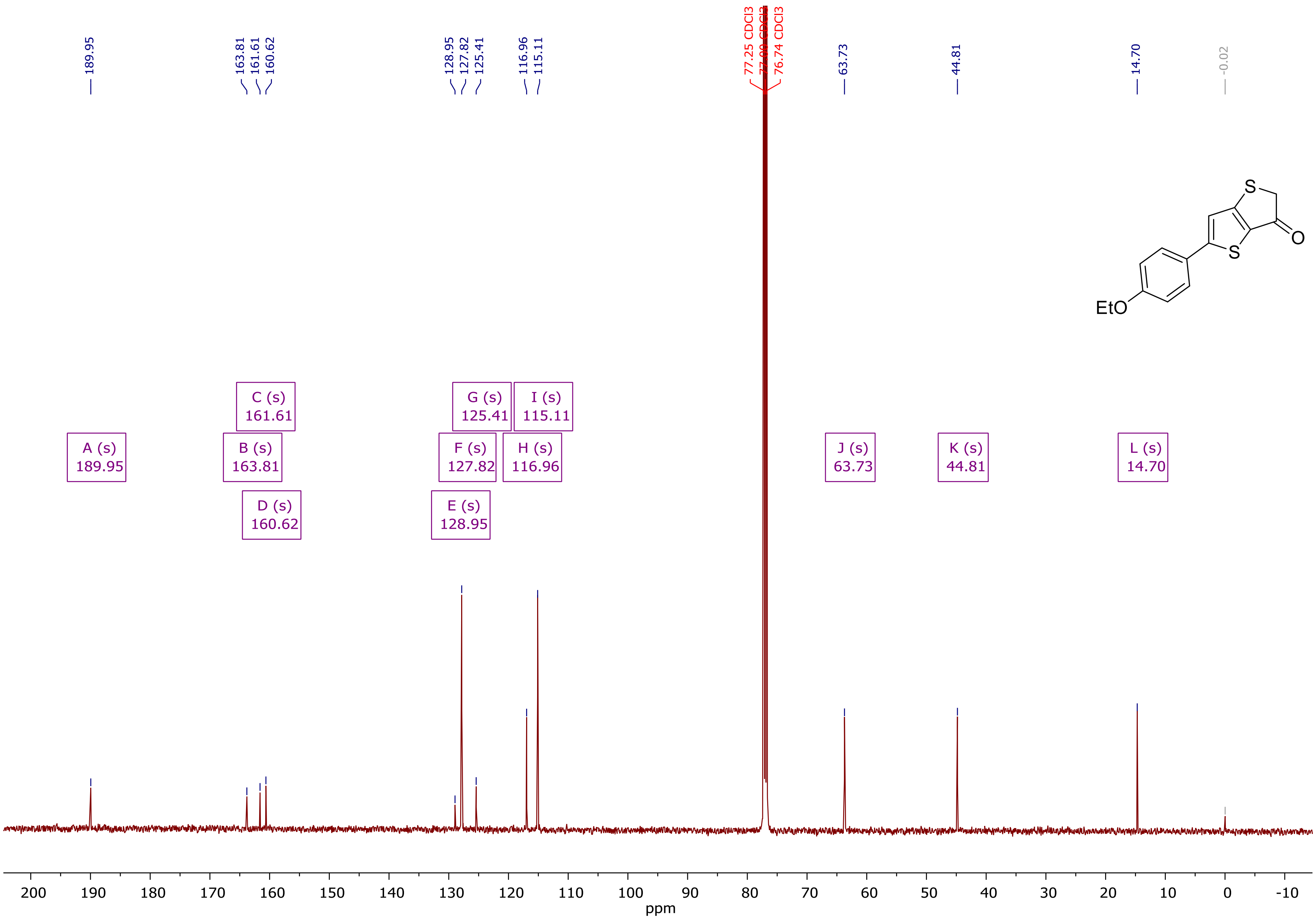

^{13}C NMR (126 MHz, chloroform-*d*) δ 189.9, 163.8, 161.6, 160.6, 128.9, 127.8, 125.4, 117.0, 115.1, 63.7, 44.8, 14.7.

5-(Naphthalen-2-yl)thieno[3,2-*b*]thiophen-3(2*H*)-one (4j)

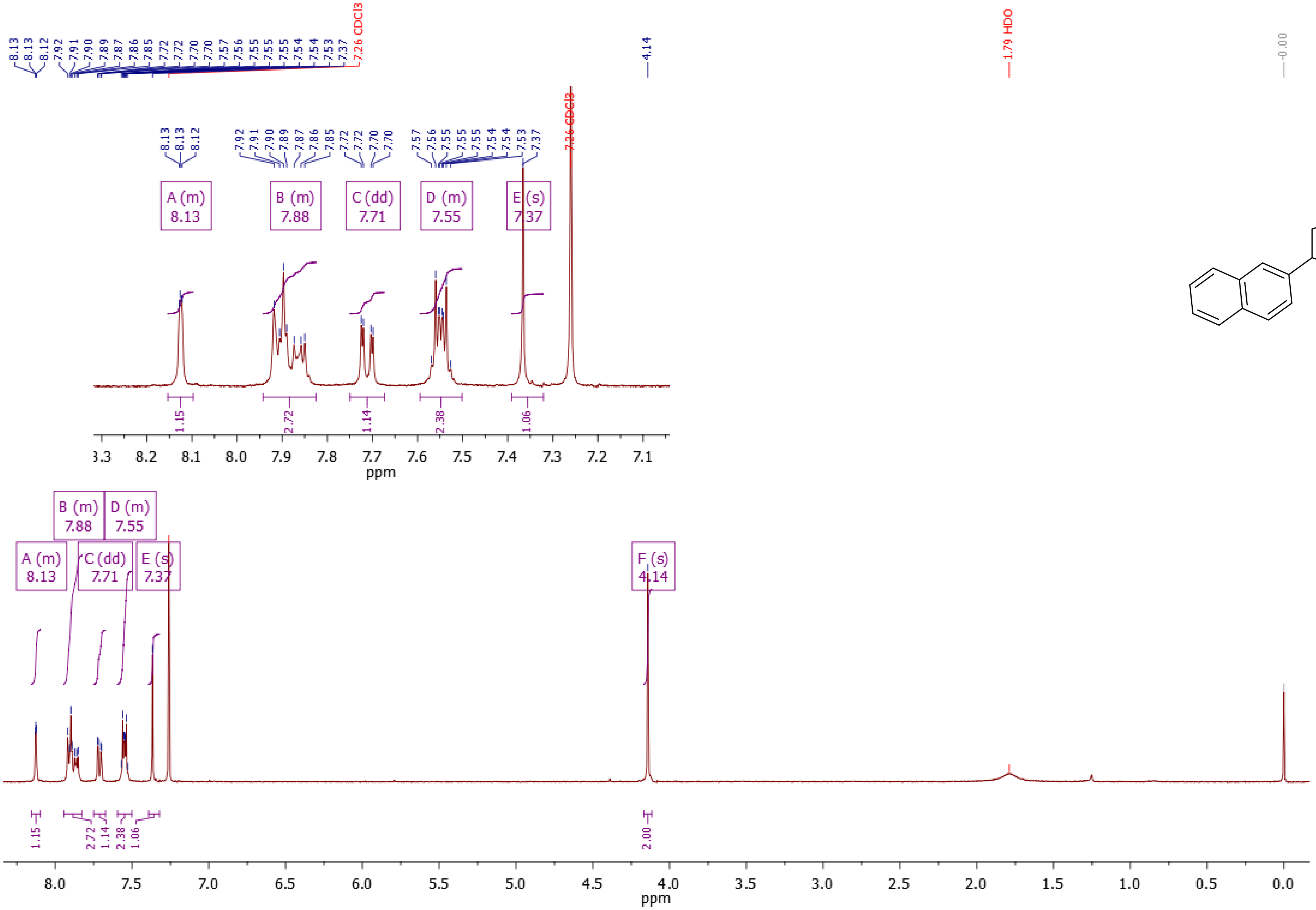

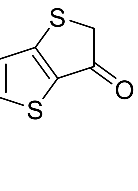

^{1}H NMR (400 MHz, chloroform-*d*) δ 8.15 – 8.10 (m, 1H), 7.94 – 7.82 (m, 3H), 7.71 (dd, *J* = 8.6, 1.9 Hz, 1H), 7.59 – 7.50 (m, 2H), 7.37 (s, 1H), 4.14 (s, 2H).

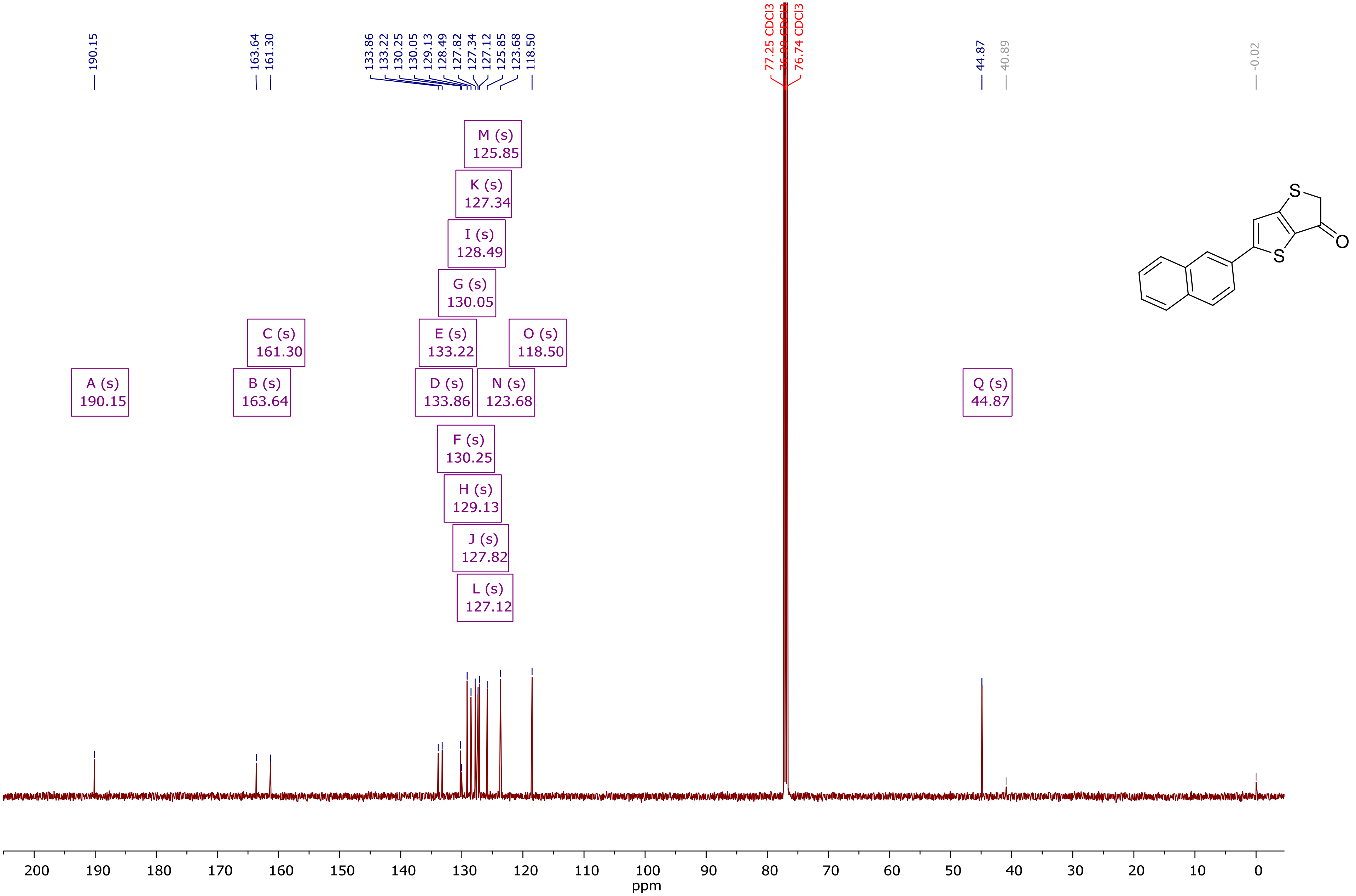

^{13}C NMR (126 MHz, chloroform-*d*) δ 190.1, 163.6, 161.3, 133.9, 133.2, 130.2, 130.0, 129.1, 128.5, 127.8, 127.3, 127.1, 125.8, 123.7, 118.5, 44.9.

6-Phenylthieno[3,2-*b*]thiophen-3(2*H*)-one (4k)

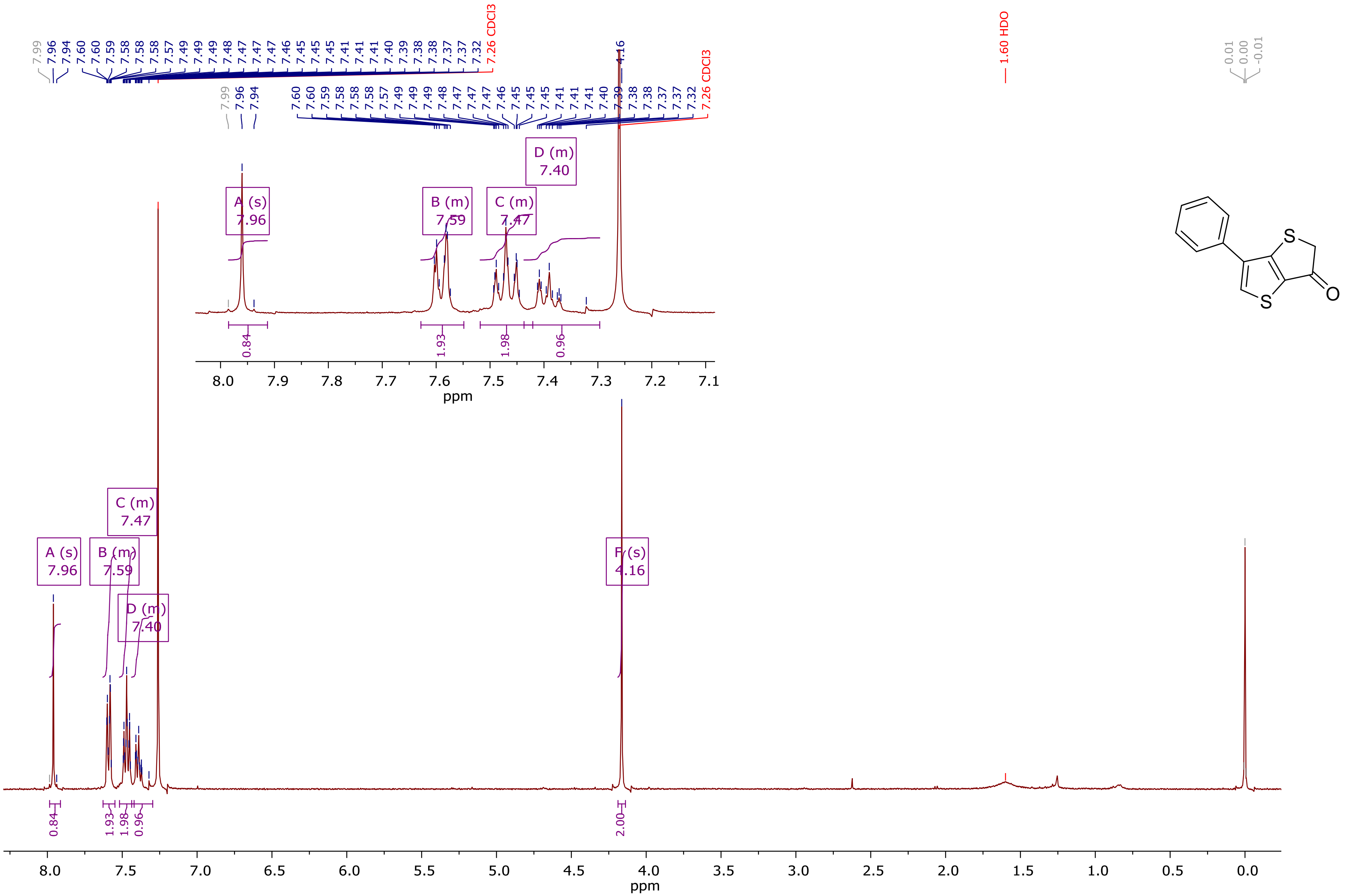

^{1}H NMR (400 MHz, chloroform-*d*) δ 7.96 (s, 1H), 7.63 – 7.55 (m, 2H), 7.52 – 7.42 (m, 2H), 7.44 – 7.30 (m, 1H), 4.16 (s, 2H).

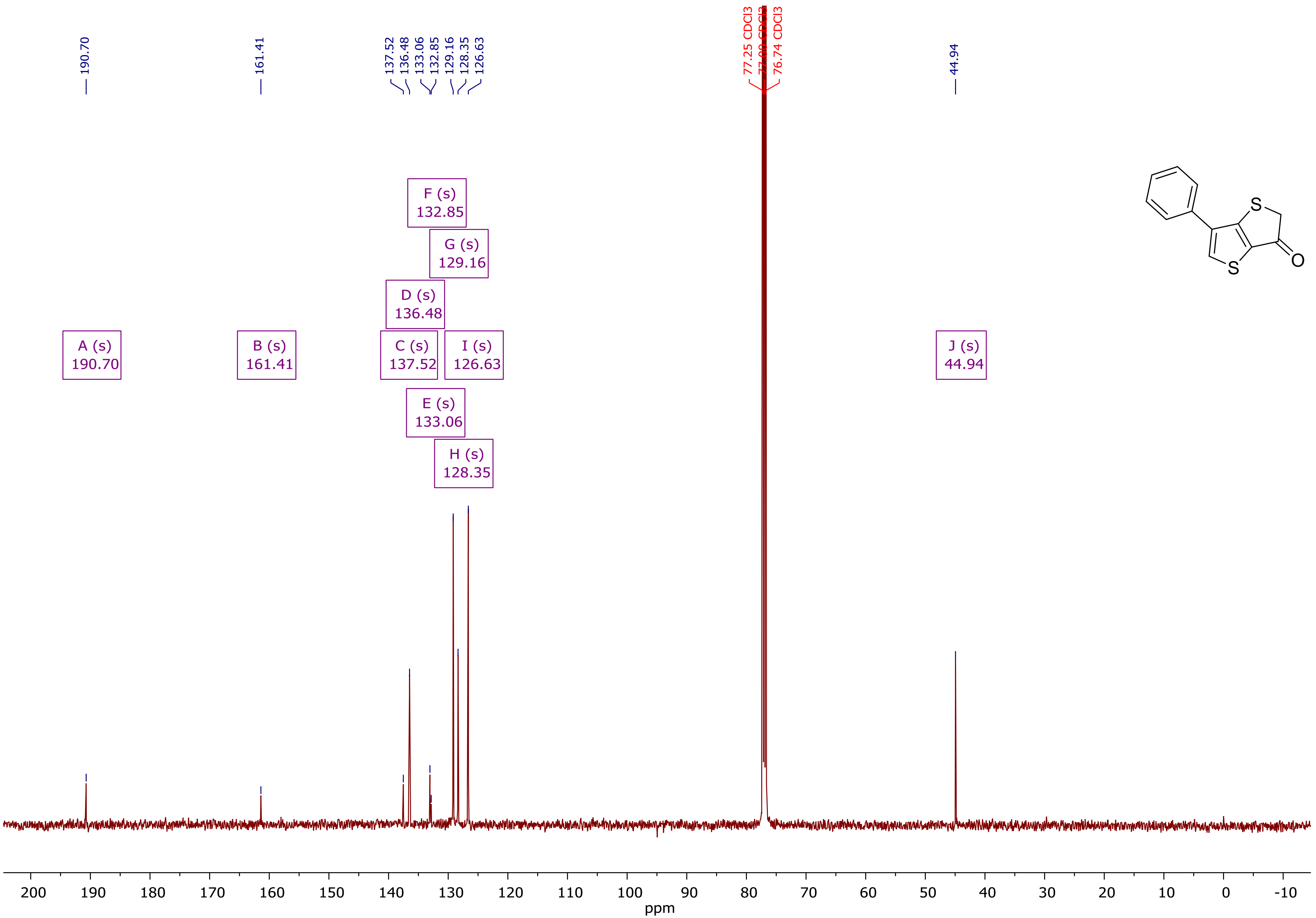

^{13}C NMR (126 MHz, chloroform-*d*) δ 190.7, 161.4, 137.5, 136.5, 133.1, 132.8, 129.2, 128.3, 126.6, 44.9.

2-Phenyl-9*H*-thieno[2',3':4,5]thieno[3,2-*b*]indole (6a)

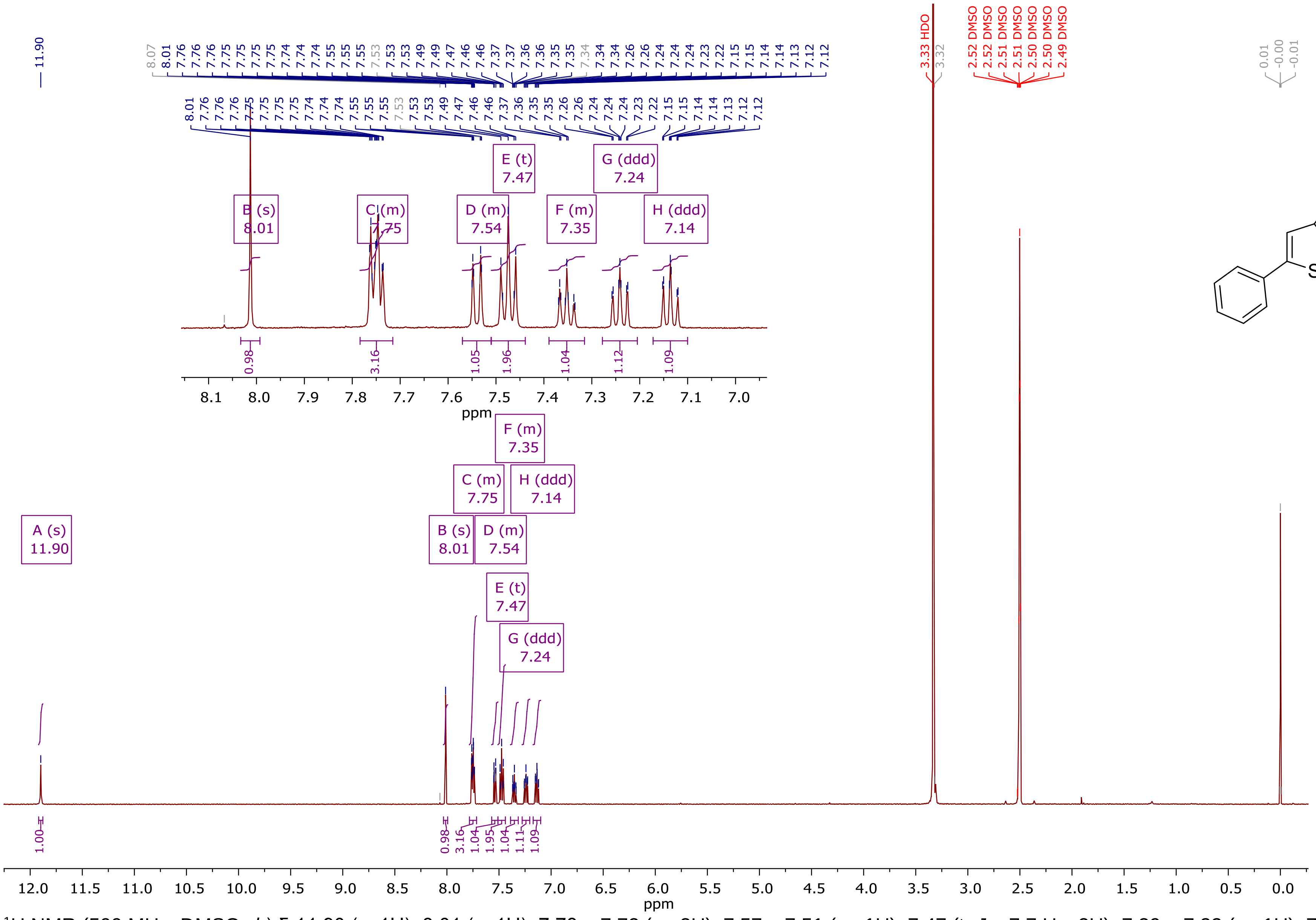

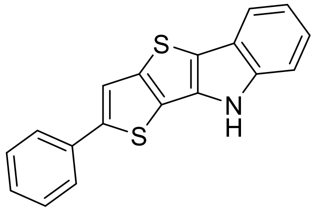

^{1}H NMR (500 MHz, DMSO-d_6) δ 11.90 (s, 1H), 8.01 (s, 1H), 7.78 – 7.72 (m, 3H), 7.57 – 7.51 (m, 1H), 7.47 (t, *J* = 7.7 Hz, 2H), 7.39 – 7.32 (m, 1H), 7.24 (ddd, *J* = 8.2, 7.1, 1.2 Hz, 1H), 7.14 (ddd, *J* = 8.1, 7.1, 1.0 Hz, 1H).

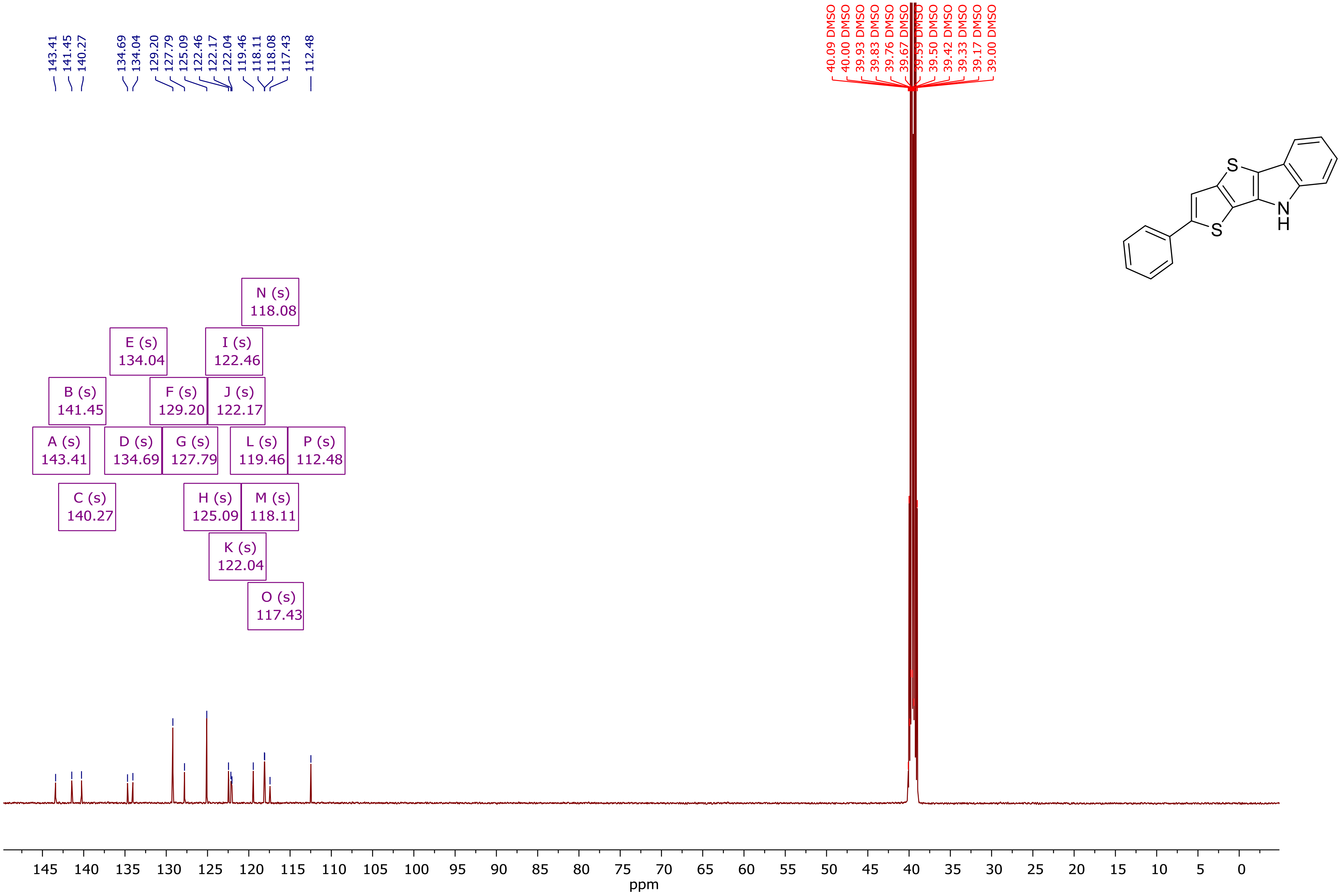

^{13}C NMR (126 MHz, DMSO-d_6) δ 143.4, 141.4, 140.3, 134.7, 134.0, 129.2, 127.8, 125.1, 122.5, 122.2, 122.0, 119.5, 118.11, 118.08, 117.4, 112.5.

2-(*p*-Tolyl)-9*H*-thieno[2',3':4,5]thieno[3,2-*b*]indole (6b)

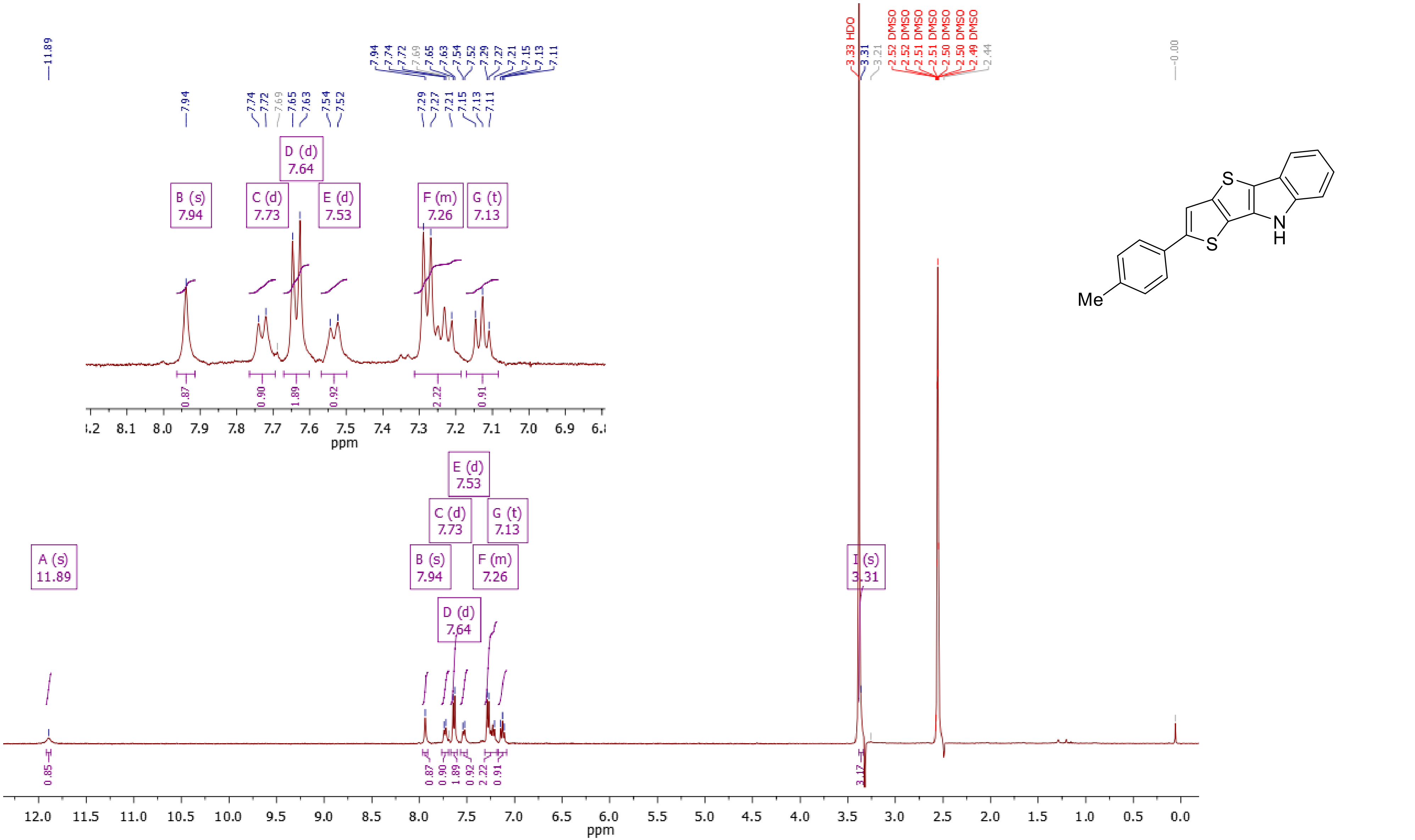

^{1}H NMR (400 MHz, DMSO-d_6) δ 11.89 (s, 1H), 7.94 (s, 1H), 7.73 (d, *J* = 8.1 Hz, 1H), 7.64 (d, *J* = 7.8 Hz, 2H), 7.53 (d, *J* = 8.3 Hz, 1H), 7.31 – 7.19 (m, 2H), 7.13 (t, *J* = 7.5 Hz, 1H), 3.31 (s, 3H).

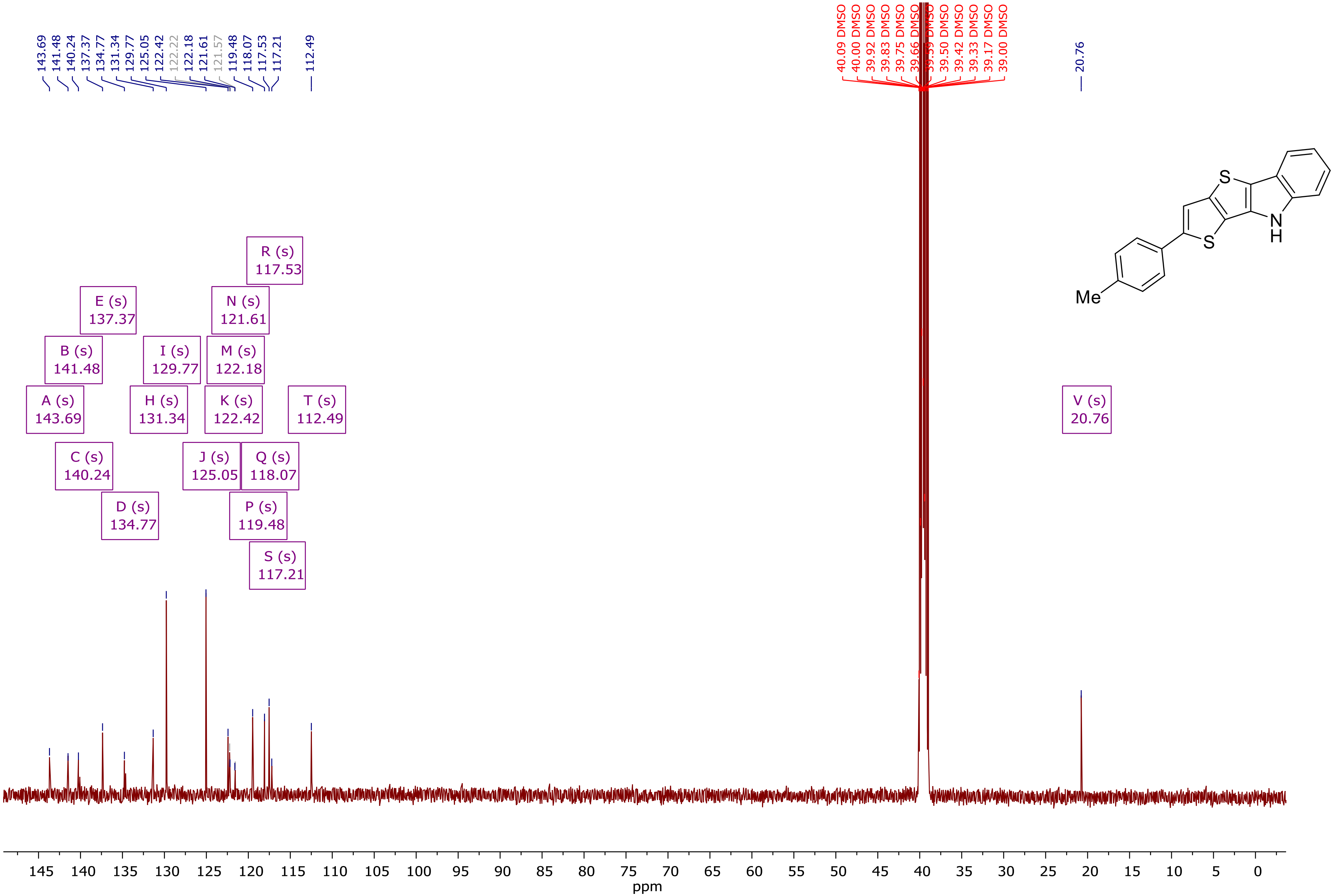

^{13}C NMR (126 MHz, DMSO-d_6) δ 143.7, 141.5, 140.2, 137.4, 134.8, 131.3, 129.8, 125.0, 122.4, 122.2, 121.6, 119.5, 118.1, 117.5, 117.2, 112.5, 20.8.

2-(2,5-Dimethylphenyl)-9*H*-thieno[2',3':4,5]thieno[3,2-*b*]indole (6c)

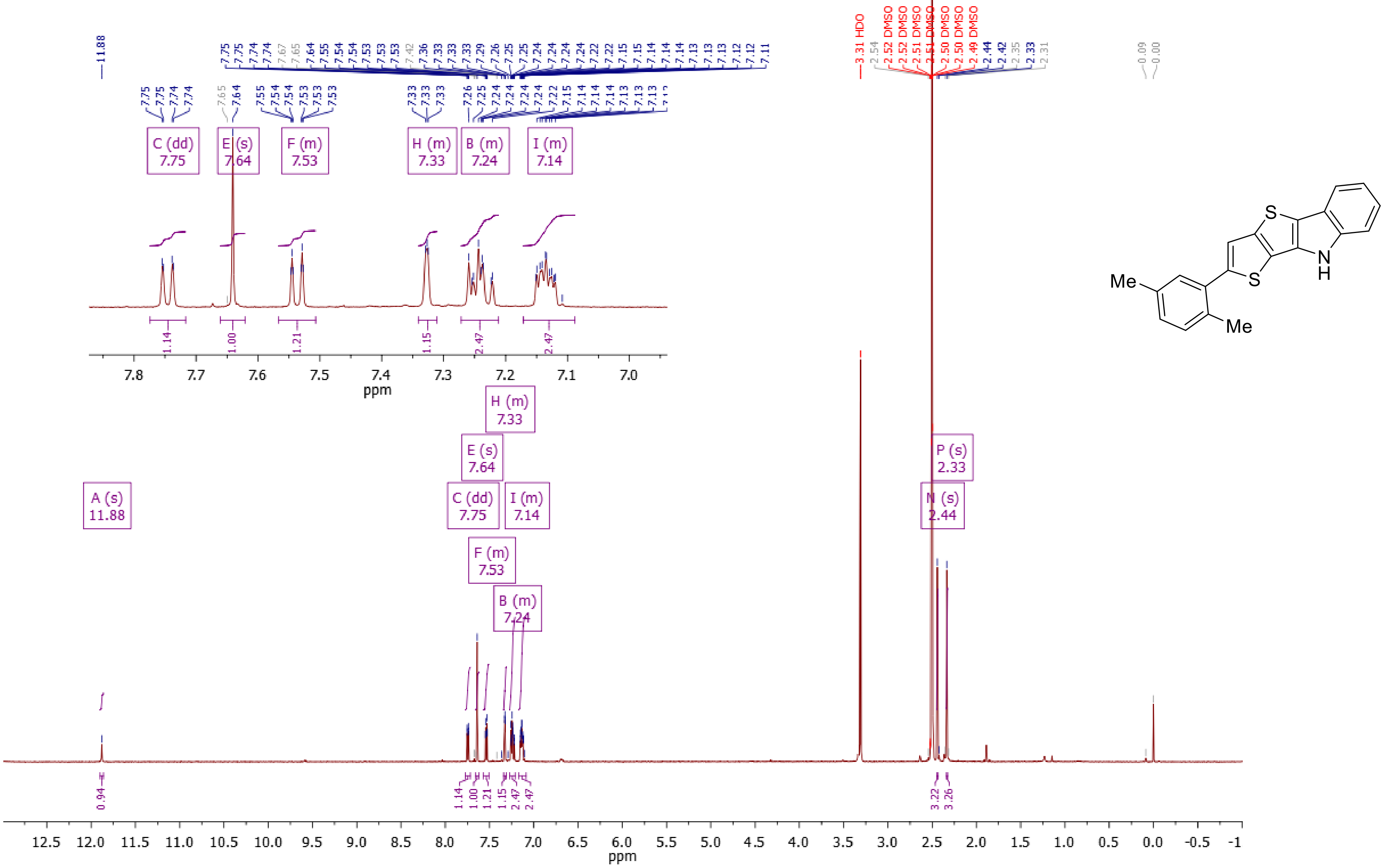

^{1}H NMR (500 MHz, DMSO-d_6) δ 11.88 (s, 1H), 7.75 (dd, J = 7.9, 1.0 Hz, 1H), 7.64 (s,1H), 7.57 – 7.51 (m, 1H), 7.34 – 7.31 (m, 1H), 7.27 – 7.21 (m, 2H), 7.17 – 7.09 (m, 2H), 2.44 (s, 3H), 2.33 (s, 3H).

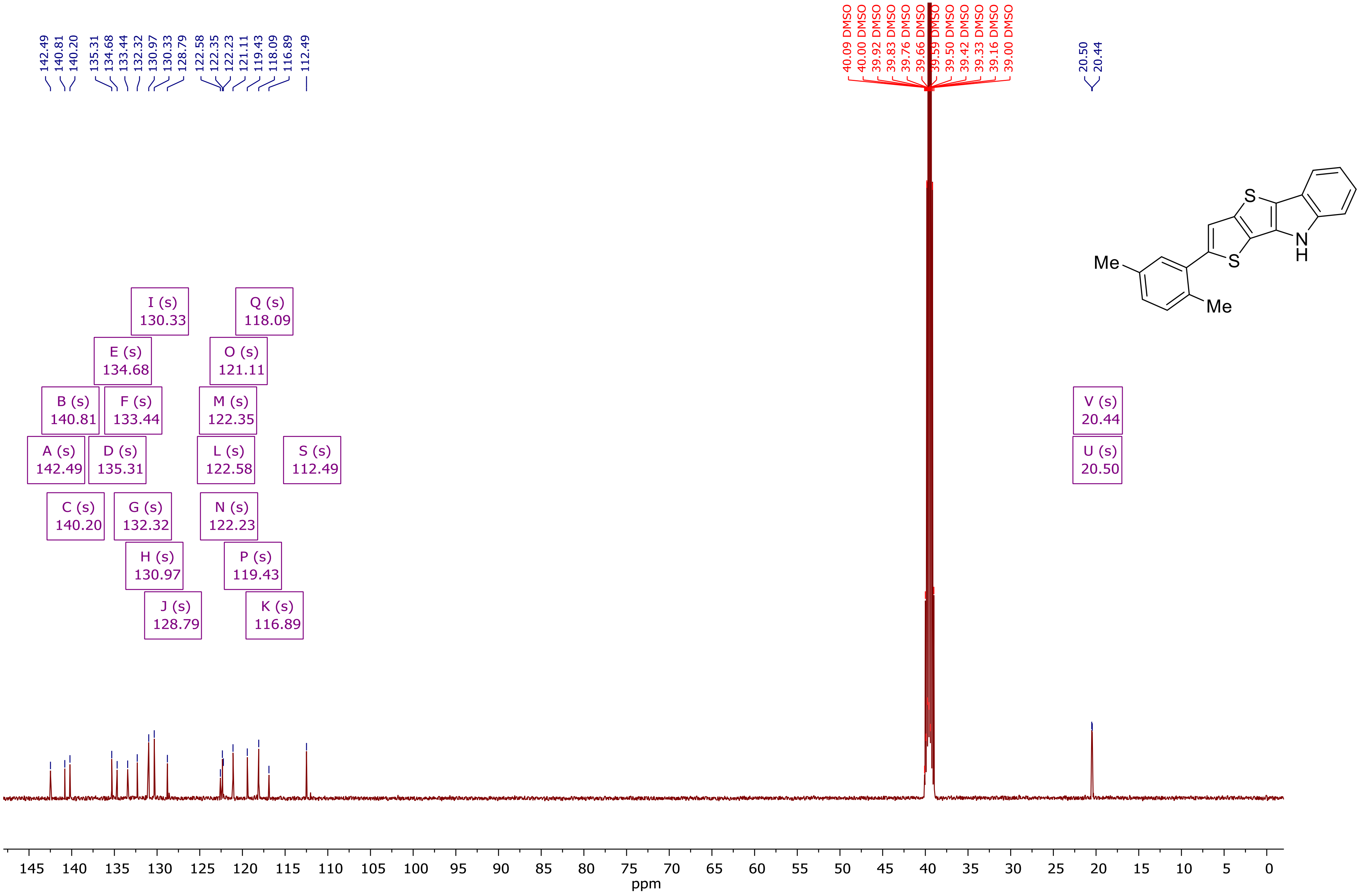

^{13}C NMR (126 MHz, DMSO-d_6) δ 142.5, 140.8, 140.2, 135.3, 134.7, 133.4, 132.3, 131.0, 130.3, 128.8, 122.6, 122.3, 122.2, 121.1, 119.4, 118.1, 116.9, 112.5, 20.5, 20.4.

2-(4-(*Tert*-butyl)phenyl)-9*H*-thieno[2',3':4,5]thieno[3,2-*b*]indole (6d)

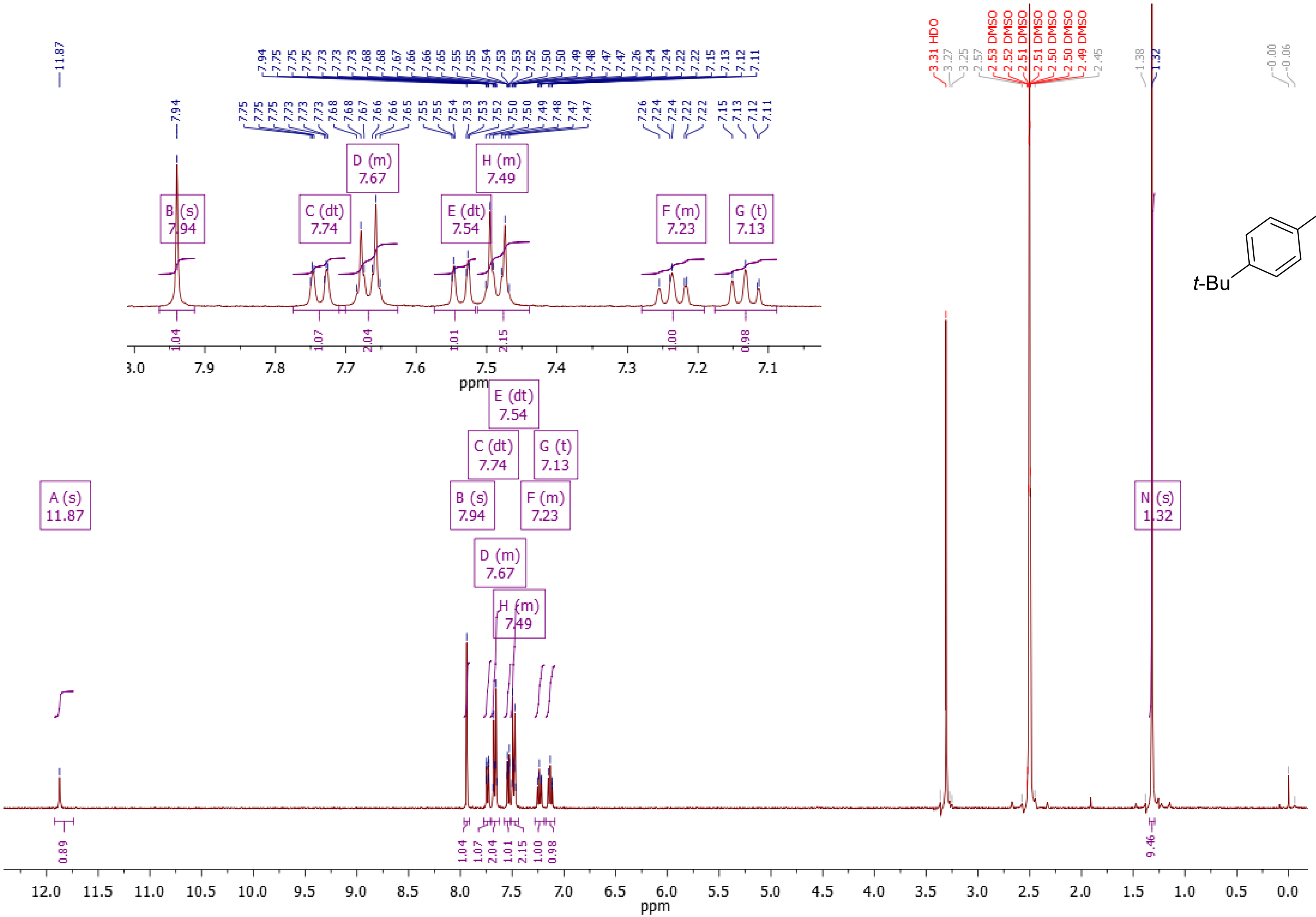

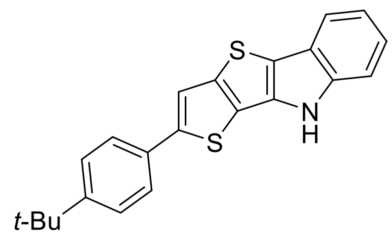

^{1}H NMR (400 MHz, DMSO-d_6) δ 11.87 (s, 1H), 7.94 (s, 1H), 7.74 (dt, *J* = 7.9, 1.0 Hz, 1H), 7.71 – 7.63 (m, 2H), 7.54 (dt, *J* = 8.2, 0.9 Hz, 1H), 7.51 – 7.44 (m, 2H), 7.28 – 7.19 (m, 1H), 7.13 (t, *J* = 7.5 Hz, 1H), 1.32 (s, 9H).

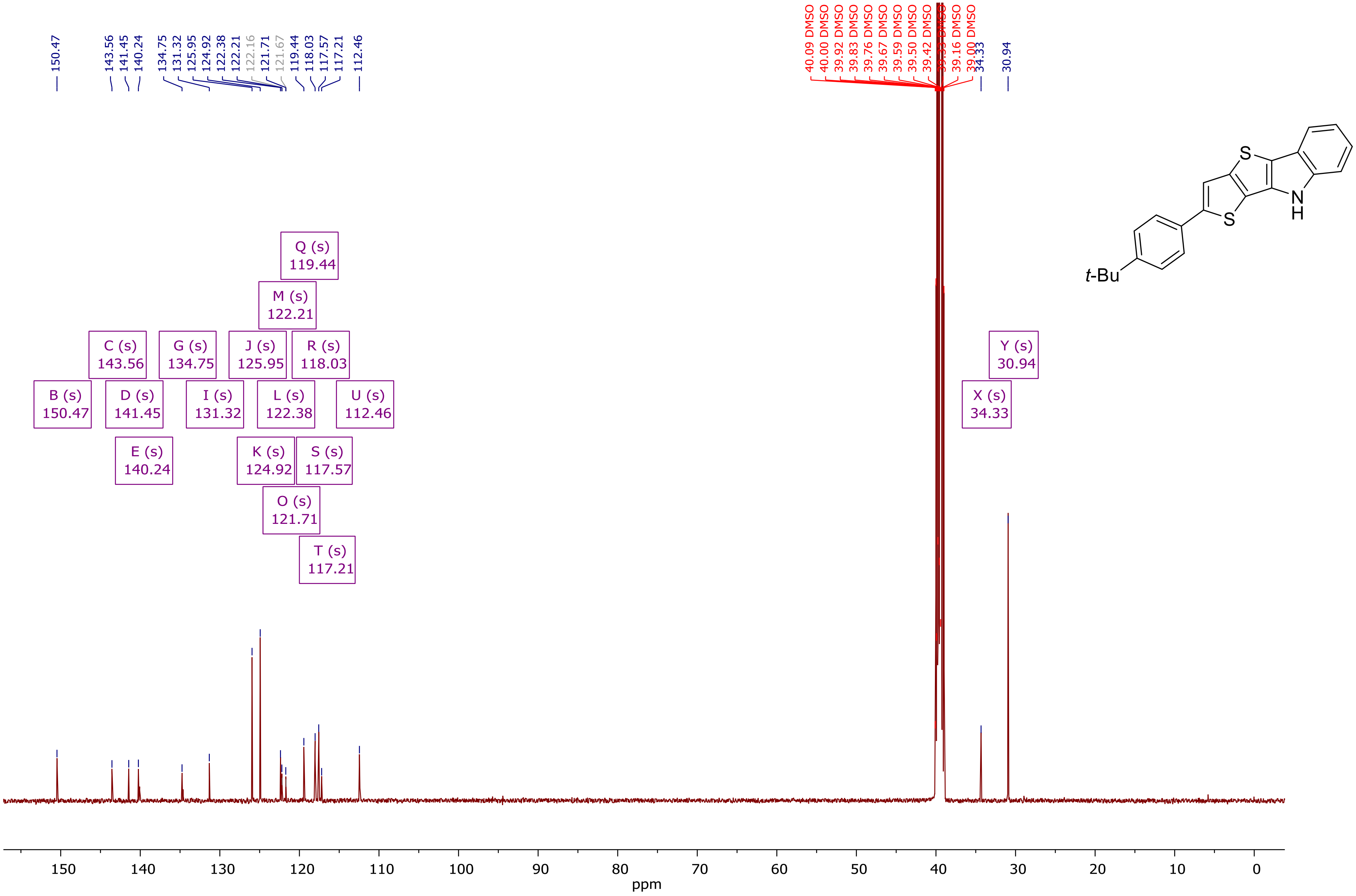

^{13}C NMR (126 MHz, DMSO-d_6) δ 150.5, 143.6, 141.4, 140.2, 134.7, 131.3, 125.9, 124.9, 122.4, 122.2, 121.7, 119.4, 118.0, 117.6, 117.2, 112.5, 34.3, 30.9.

2-(4-Fluorophenyl)-9*H*-thieno[2',3':4,5]thieno[3,2-*b*]indole (6e)

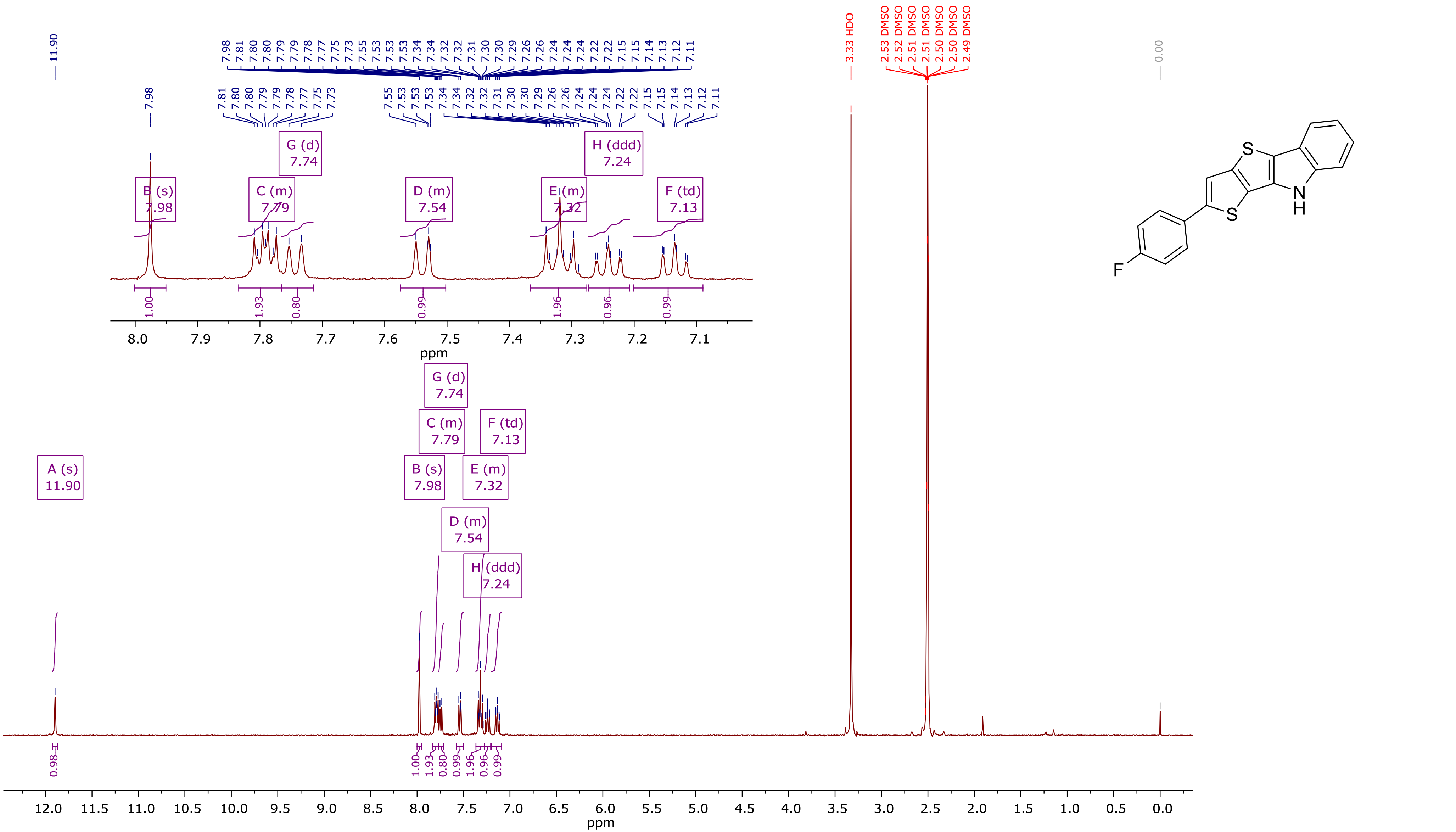

^{1}H NMR (400 MHz, DMSO-d_6) δ 11.90 (s, 1H), 7.98 (s, 1H), 7.83 – 7.76 (m, 2H), 7.74 (d, *J* = 7.9 Hz, 1H), 7.57 – 7.50 (m, 1H), 7.37 – 7.28 (m, 2H), 7.24 (ddd, *J* = 8.3, 7.1, 1.2 Hz, 1H), 7.13 (td, *J* = 7.5, 7.1, 1.1 Hz, 1H).

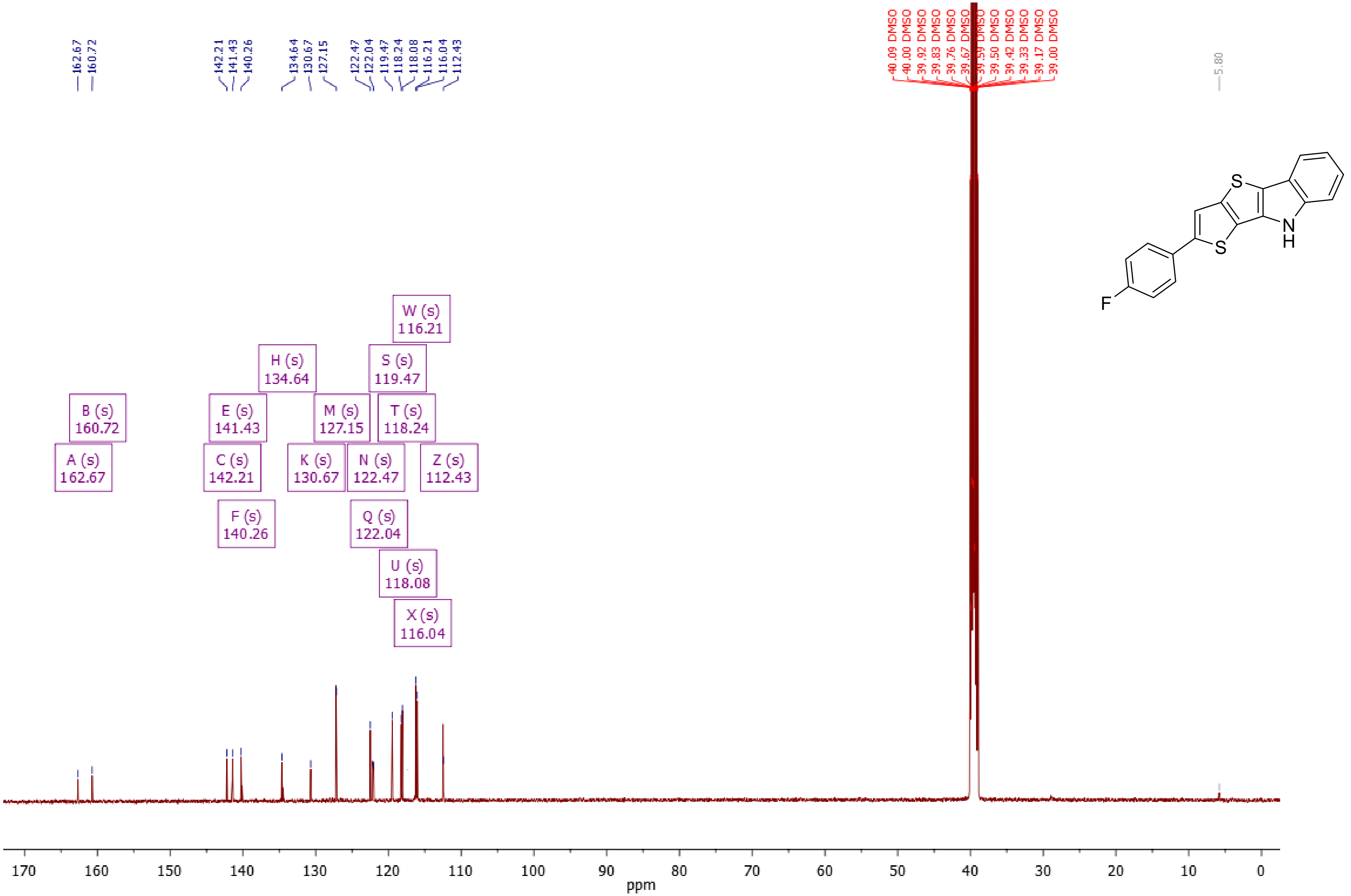

^{13}C NMR (126 MHz, DMSO-d_6) δ 162.7, 160.7, 142.2, 141.4, 140.3, 134.6, 130.7, 127.1, 122.5, 122.0, 119.5, 118.2, 118.1, 116.2, 116.0, 112.4.

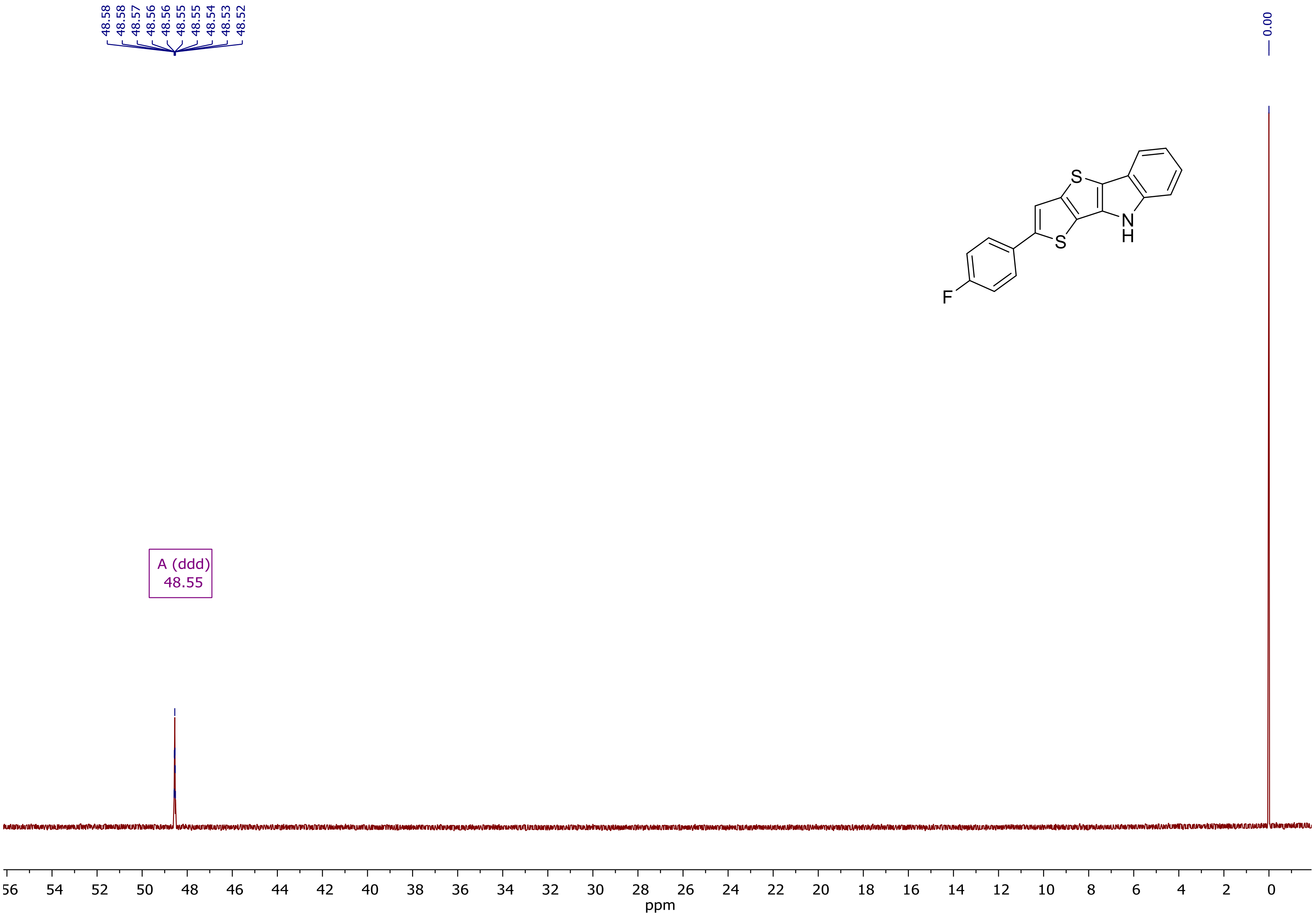

^{19}F NMR (471 MHz, DMSO-d_6) δ 48.55 (ddd, J = 8.8, 5.7, 3.4 Hz).

2-(4-Chlorophenyl)-9*H*-thieno[2',3':4,5]thieno[3,2-*b*]indole (6f)

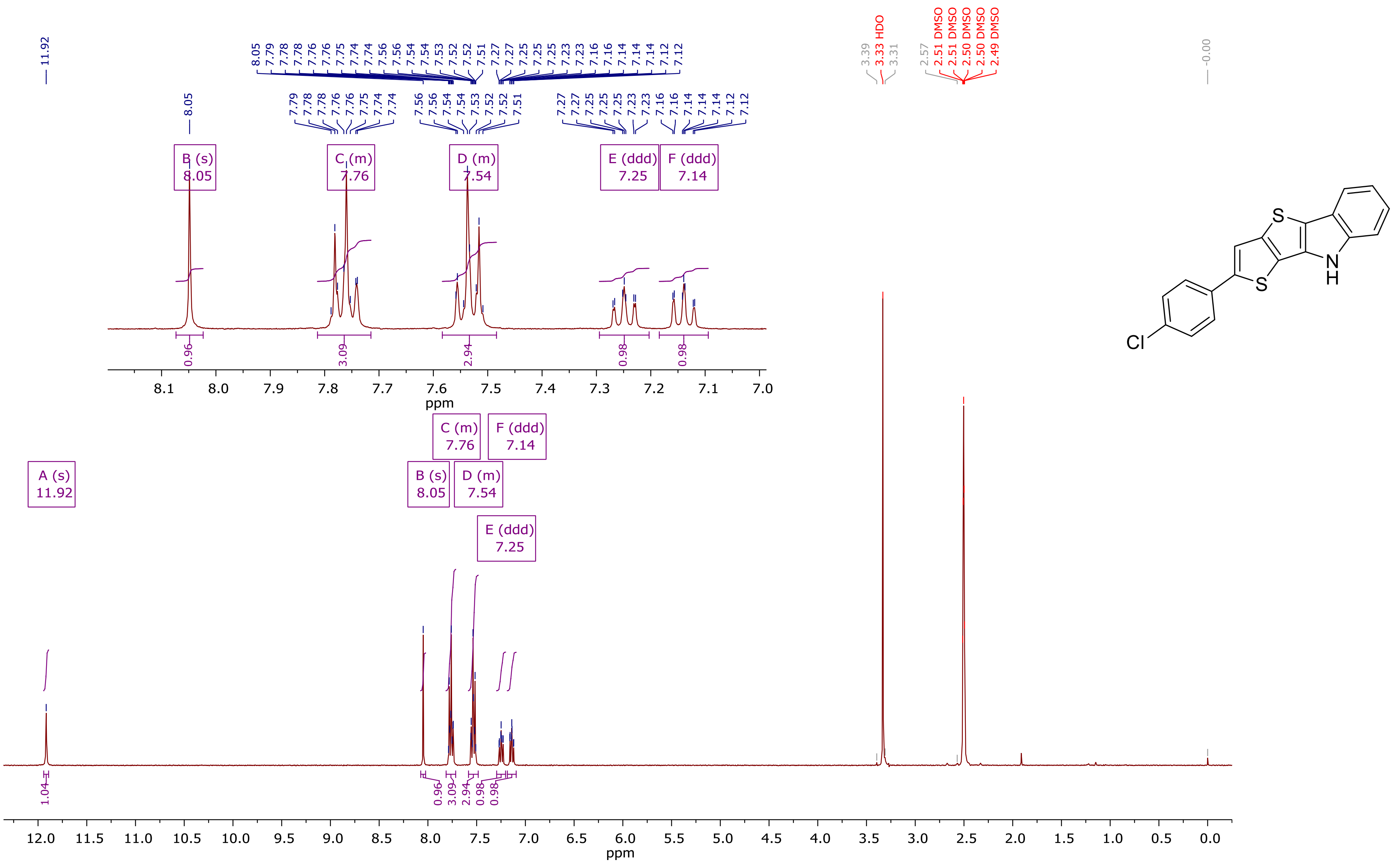

^{1}H NMR (400 MHz, DMSO-d_6) δ 11.92 (s, 1H), 8.05 (s, 1H), 7.81 – 7.71 (m, 3H), 7.58 – 7.48 (m, 3H), 7.25 (ddd, *J* = 8.3, 7.1, 1.2 Hz, 1H), 7.14 (ddd, *J* = 8.1, 7.1, 1.0 Hz, 1H).

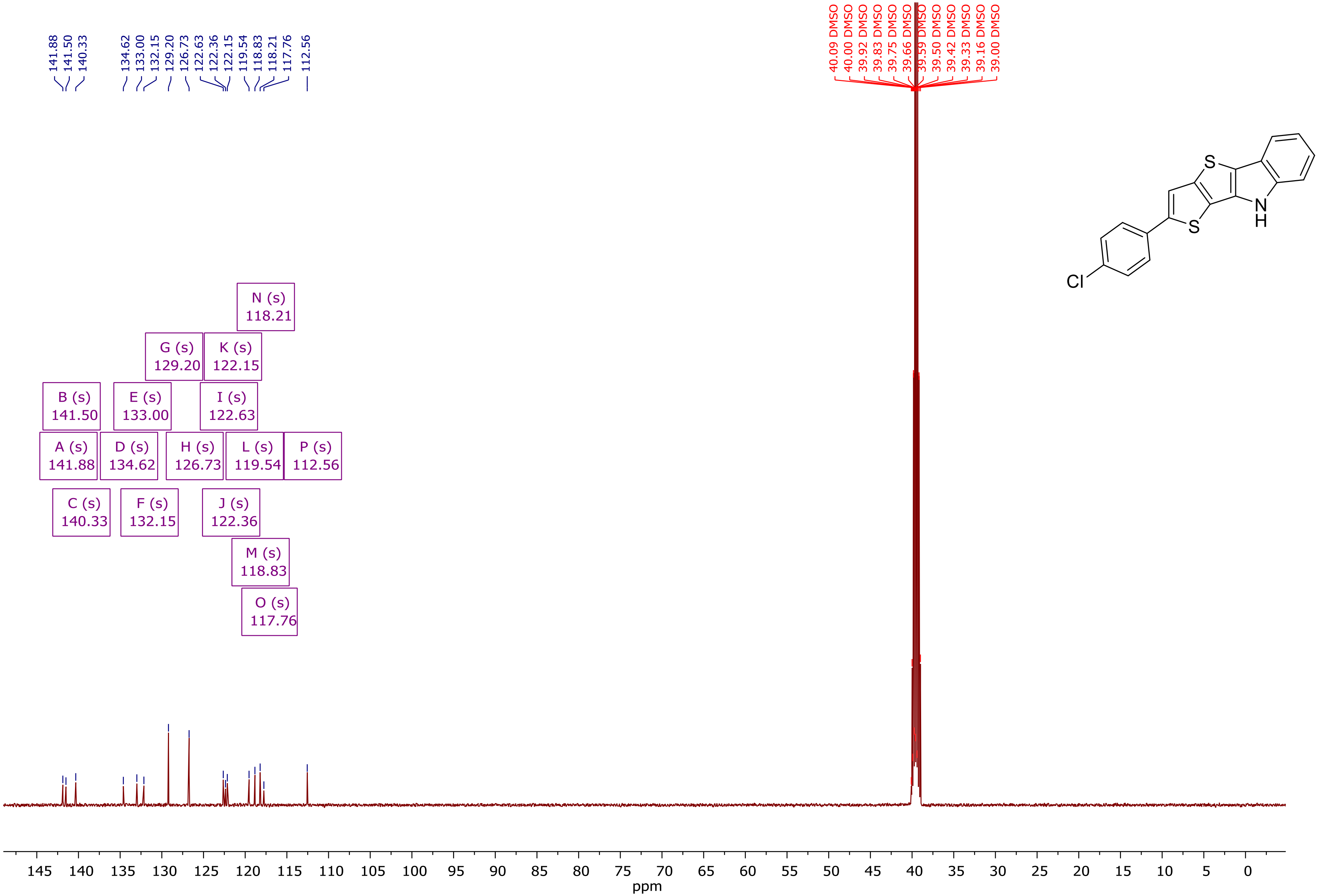

^{13}C NMR (126 MHz, DMSO-d_6) δ 141.9, 141.5, 140.3, 134.6, 133.0, 132.1, 129.2, 126.7, 122.6, 122.4, 122.1, 119.5, 118.8, 118.2, 117.8, 112.6.

2-(4-Bromophenyl)-9*H*-thieno[2',3':4,5]thieno[3,2-*b*]indole (6g)

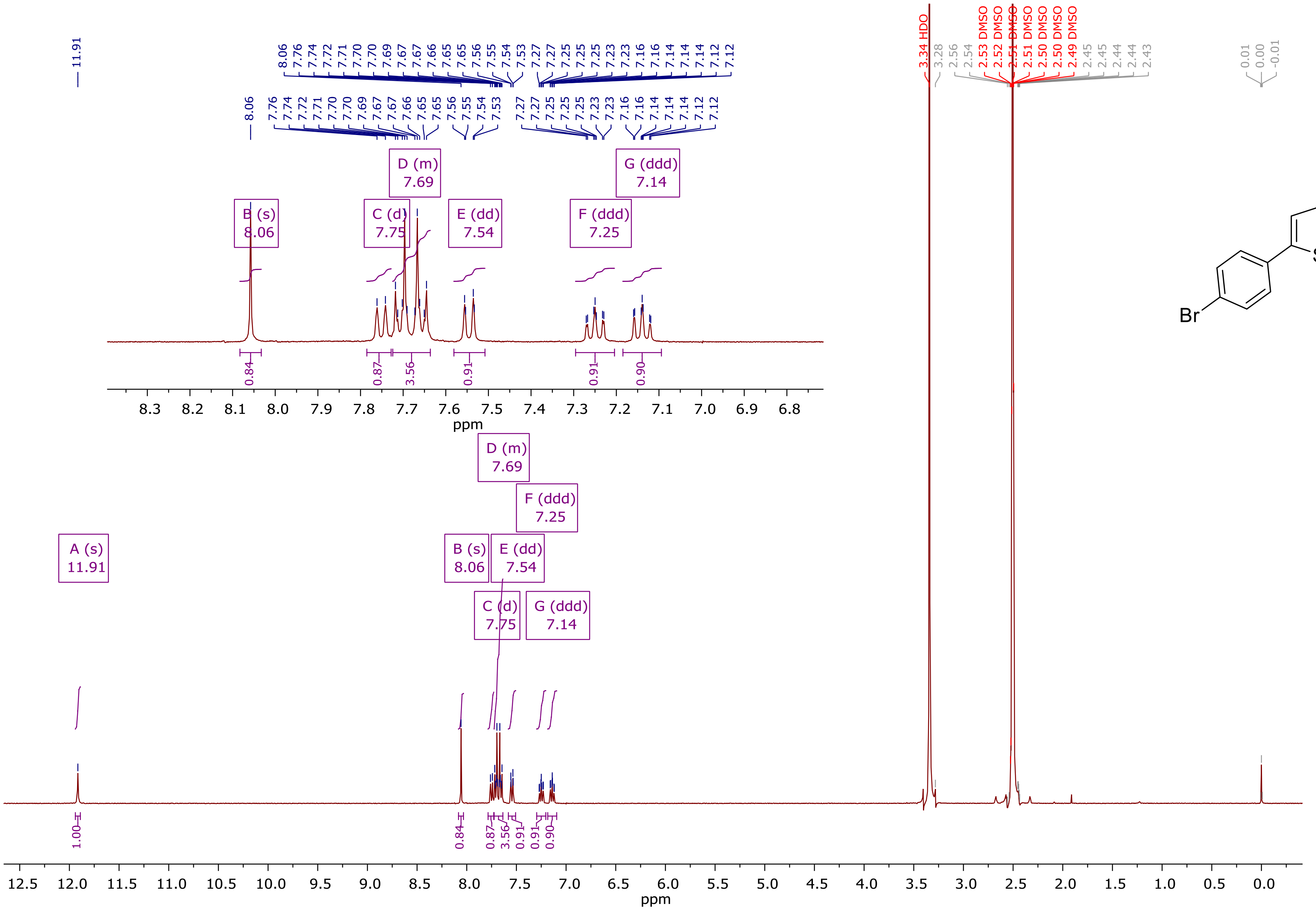

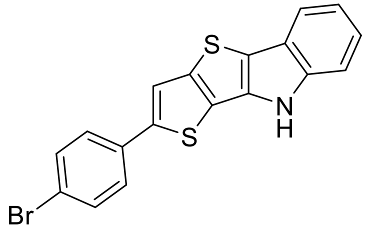

^{1}H NMR (400 MHz, DMSO-d_6) δ 11.91 (s, 1H), 8.06 (s, 1H), 7.75 (d, *J* = 7.9 Hz, 1H), 7.72 – 7.64 (m, 4H), 7.54 (dd, *J* = 8.2, 1.0 Hz, 1H), 7.25 (ddd, *J* = 8.2, 7.1, 1.2 Hz, 1H), 7.14 (ddd, *J* = 8.1, 7.1, 1.1 Hz, 1H).

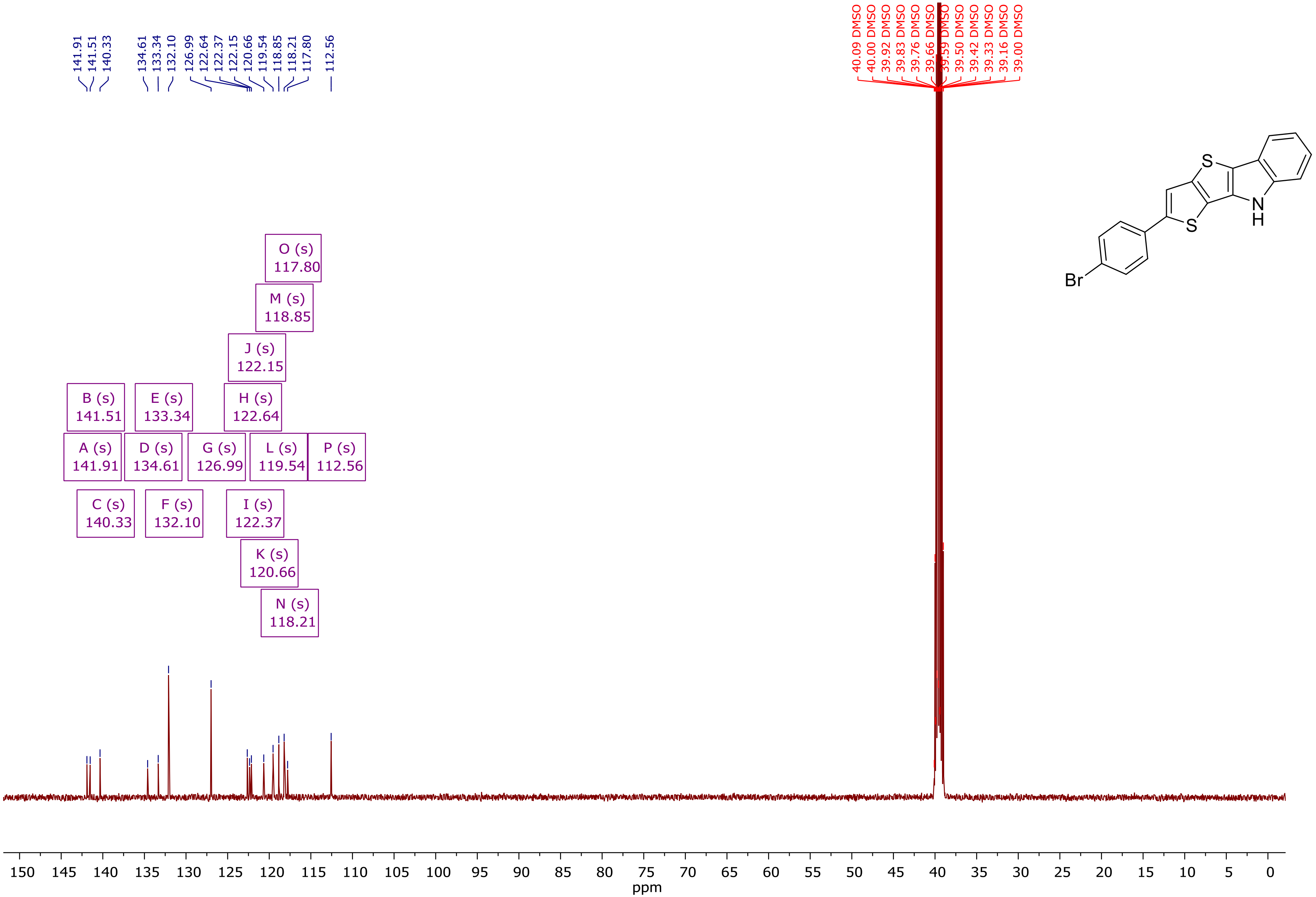

^{13}C NMR (126 MHz, DMSO-d_6) δ 141.9, 141.5, 140.3, 134.6, 133.3, 132.1, 127.0, 122.6, 122.4, 122.1, 120.7, 119.5, 118.8, 118.2, 117.8, 112.6.

2-(3-Methoxyphenyl)-9*H*-thieno[2',3':4,5]thieno[3,2-*b*]indole (6h)

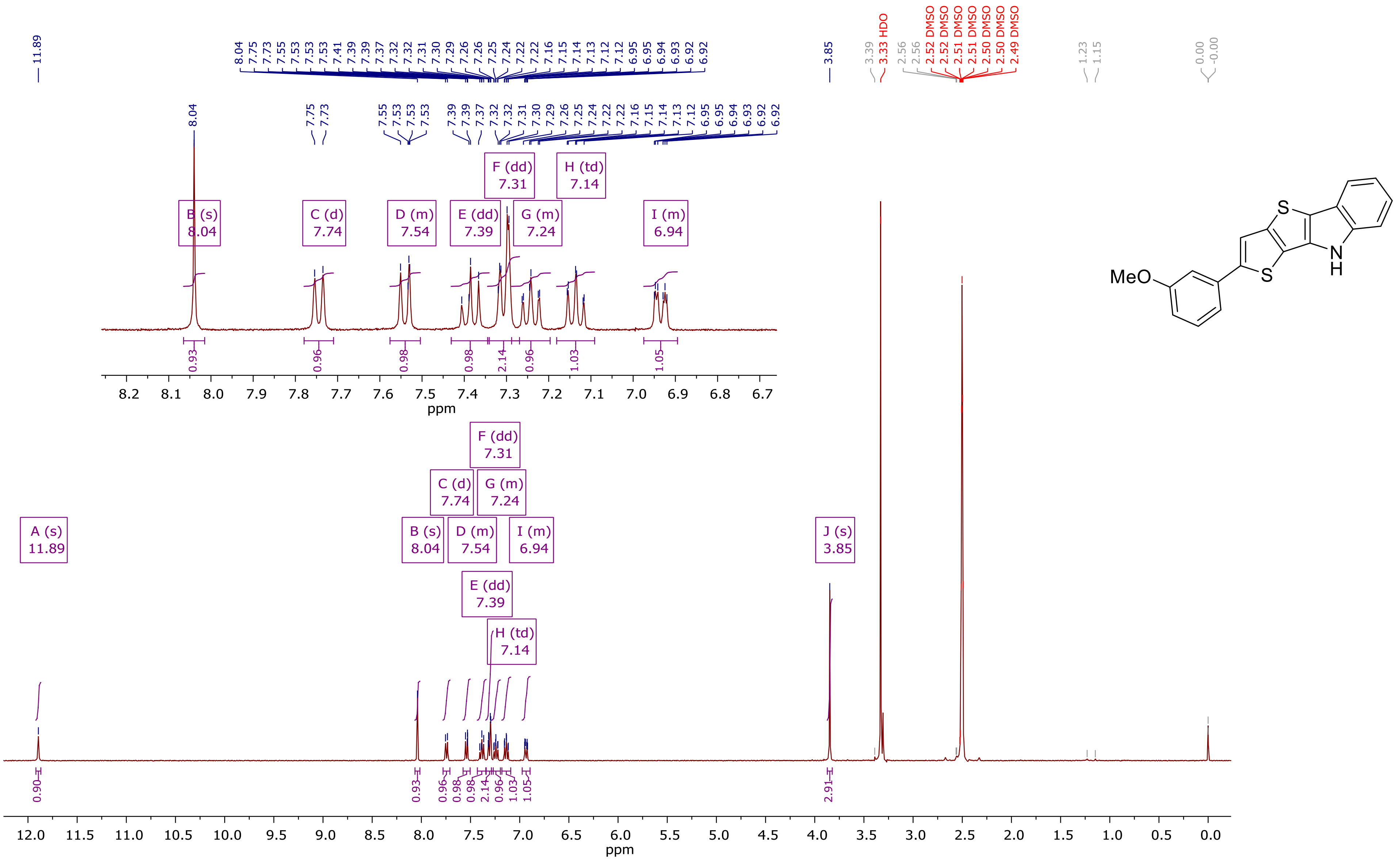

^{1}H NMR (400 MHz, DMSO-d_6) δ 11.89 (s, 1H), 8.04 (s, 1H), 7.74 (d, *J* = 7.9 Hz, 1H), 7.58 – 7.50 (m, 1H), 7.39 (dd, *J* = 8.7, 7.2 Hz, 1H), 7.31 (dd, *J* = 7.4, 1.5 Hz, 2H), 7.29 – 7.20 (m, 1H), 7.14 (td, *J* = 7.5, 1.0 Hz, 1H), 6.97 – 6.89 (m, 1H), 3.85 (s, 3H).

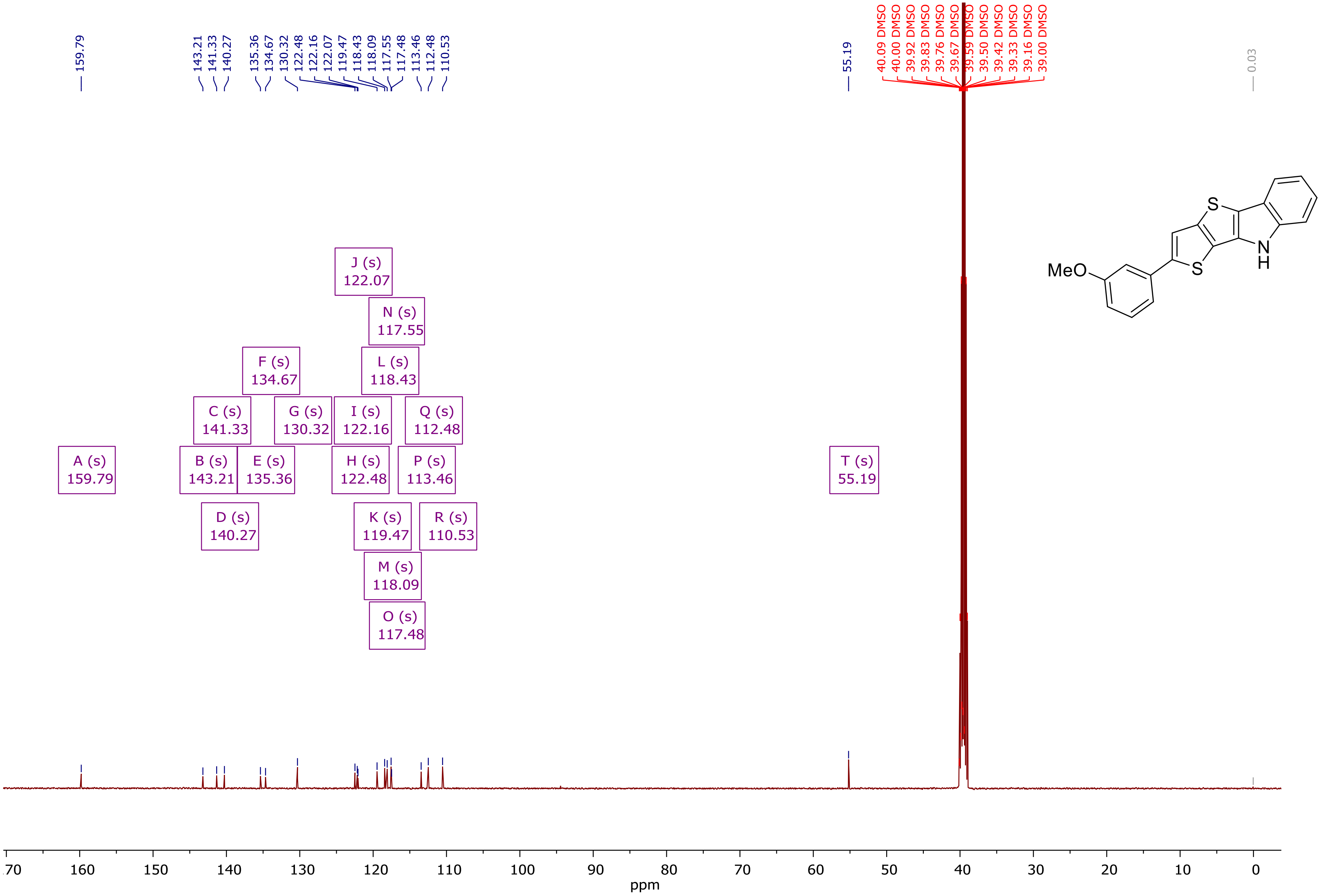

^{13}C NMR (126 MHz, DMSO-d_6) δ 159.8, 143.2, 141.3, 140.3, 135.4, 134.7, 130.3, 122.5, 122.2, 122.1, 119.5, 118.4, 118.1, 117.55, 117.48, 113.5, 112.5, 110.5, 55.2.

2-(4-Ethoxyphenyl)-9*H*-thieno[2',3':4,5]thieno[3,2-*b*]indole (6i)

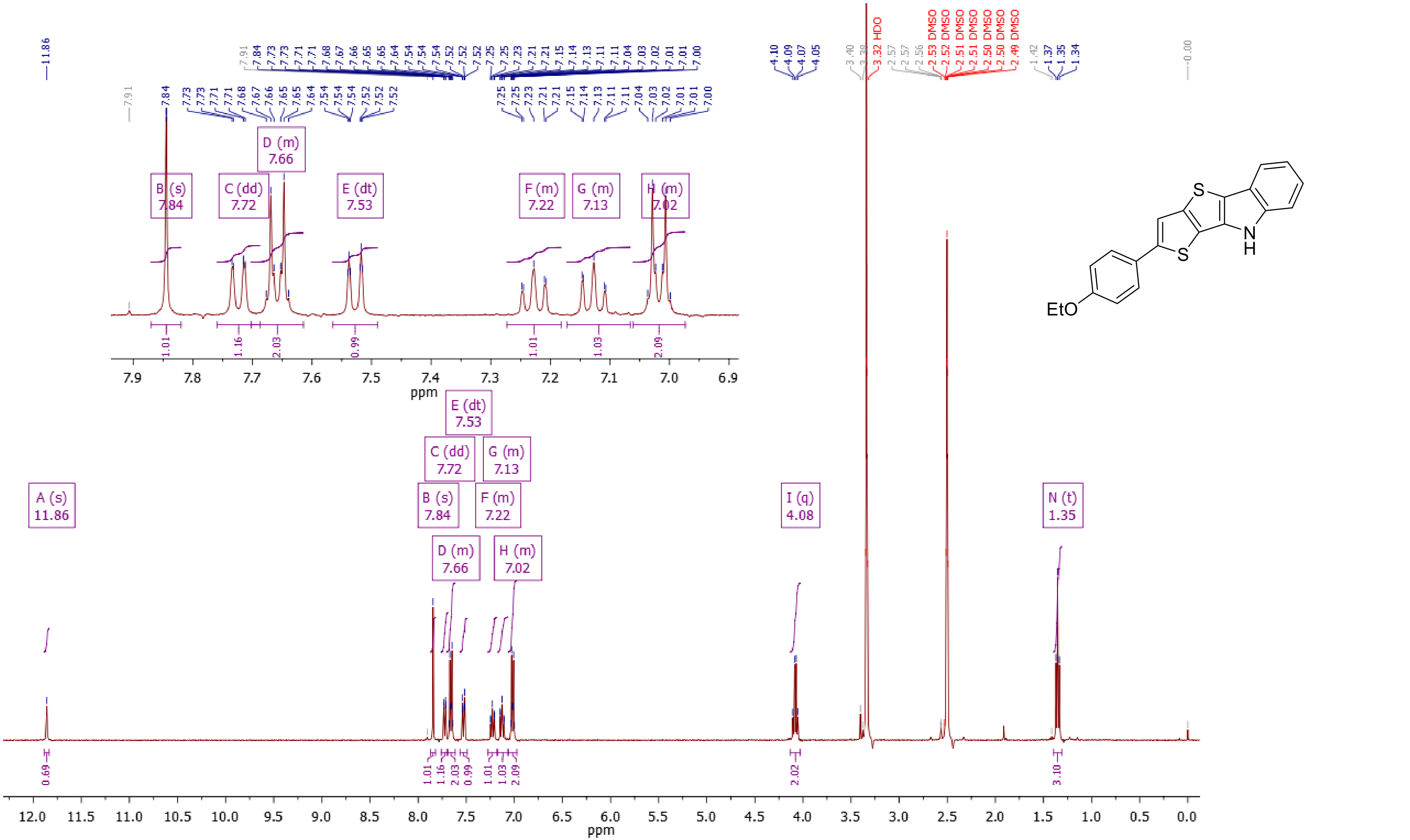

^{1}H NMR (400 MHz, DMSO-d_6) δ 11.86 (s, 1H), 7.84 (s, 1H), 7.72 (dd, *J* = 7.8, 1.1 Hz, 1H), 7.70 – 7.61 (m, 2H), 7.53 (dt, *J* = 8.2, 0.9 Hz, 1H), 7.27 – 7.18 (m, 1H), 7.17 – 7.07 (m, 1H), 7.06 – 6.97 (m, 2H), 4.08 (q, *J* = 7.0 Hz, 2H), 1.35 (t, *J* = 7.0 Hz, 3H).

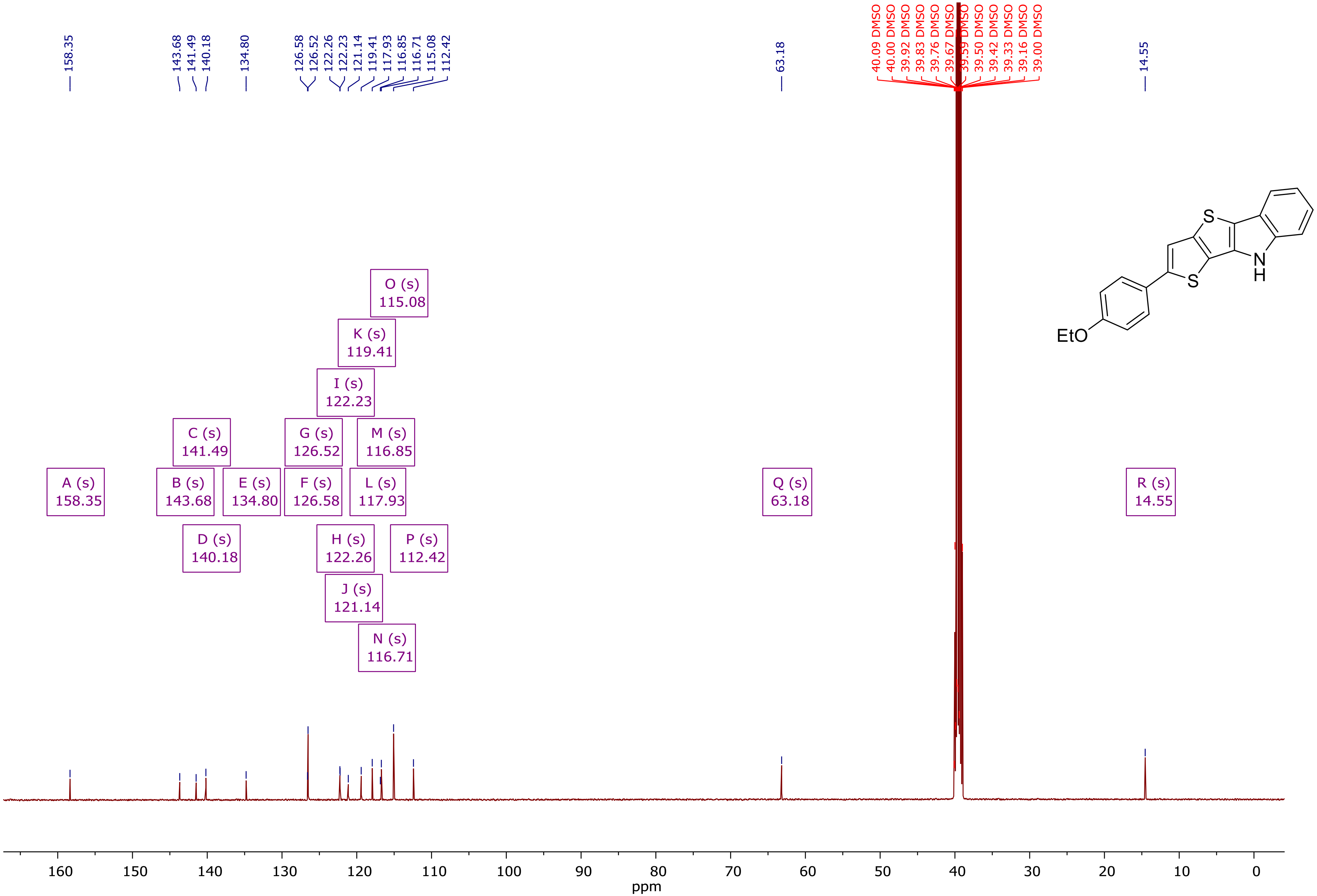

^{13}C NMR (126 MHz, DMSO-d_6) δ 158.3, 143.7, 141.5, 140.2, 134.8, 126.6, 126.5, 122.3, 122.2, 121.1, 119.4, 117.9, 116.8, 116.7, 115.1, 112.4, 63.2, 14.5.

2-(Naphthalen-2-yl)-9*H*-thieno[2',3':4,5]thieno[3,2-*b*]indole (6j)

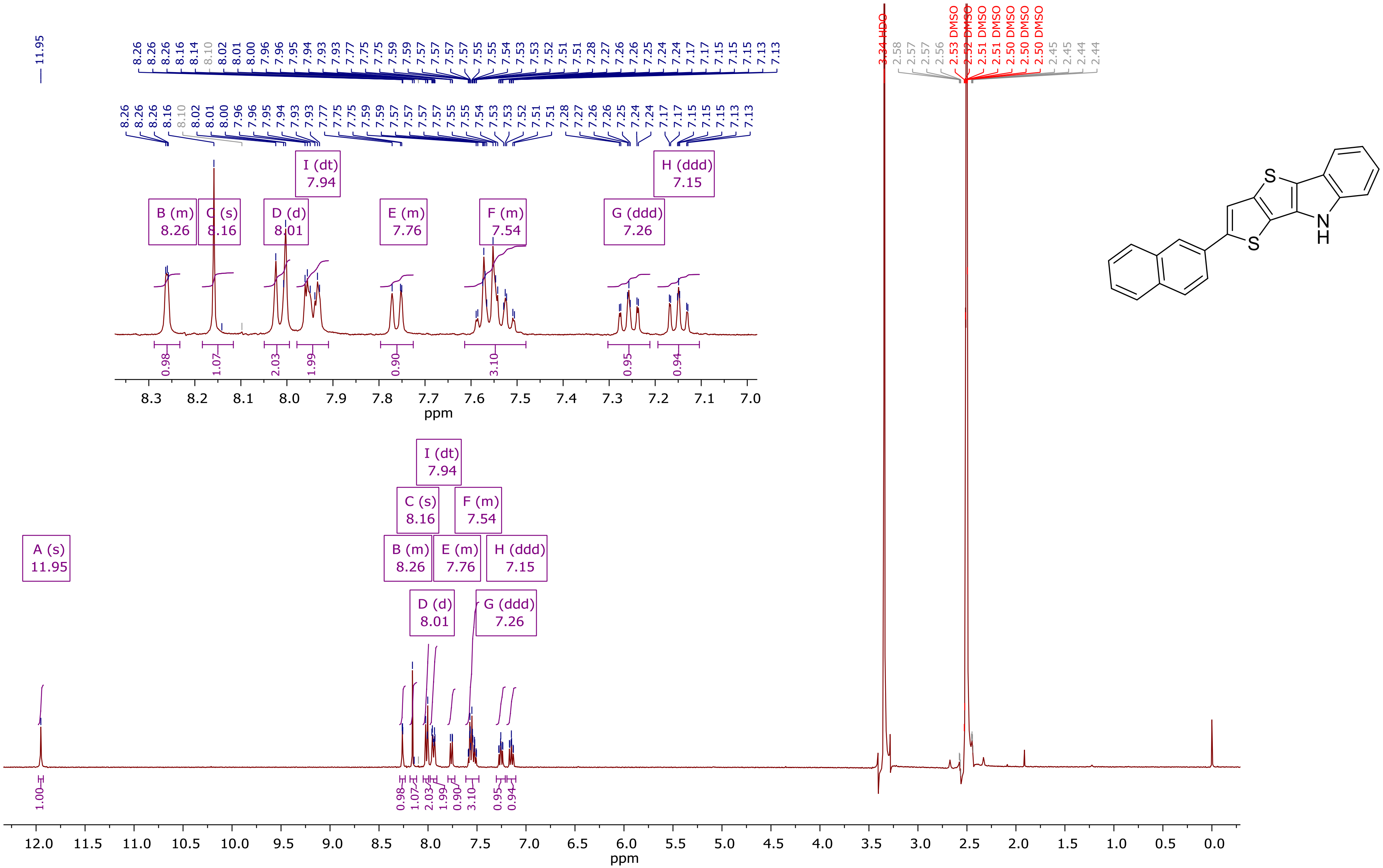

^{1}H NMR (400 MHz, DMSO-d_6) δ 11.95 (s, 1H), 8.29 – 8.23 (m, 1H), 8.16 (s, 1H), 8.01 (d, *J* = 8.5 Hz, 2H), 7.94 (dt, *J* = 8.1, 1.9 Hz, 1H), 7.80 – 7.73 (m, 1H), 7.61 – 7.48 (m, 3H), 7.26 (ddd, *J* = 8.3, 7.1, 1.2 Hz, 1H), 7.15 (ddd, *J* = 8.1, 7.1, 1.1 Hz, 1H).

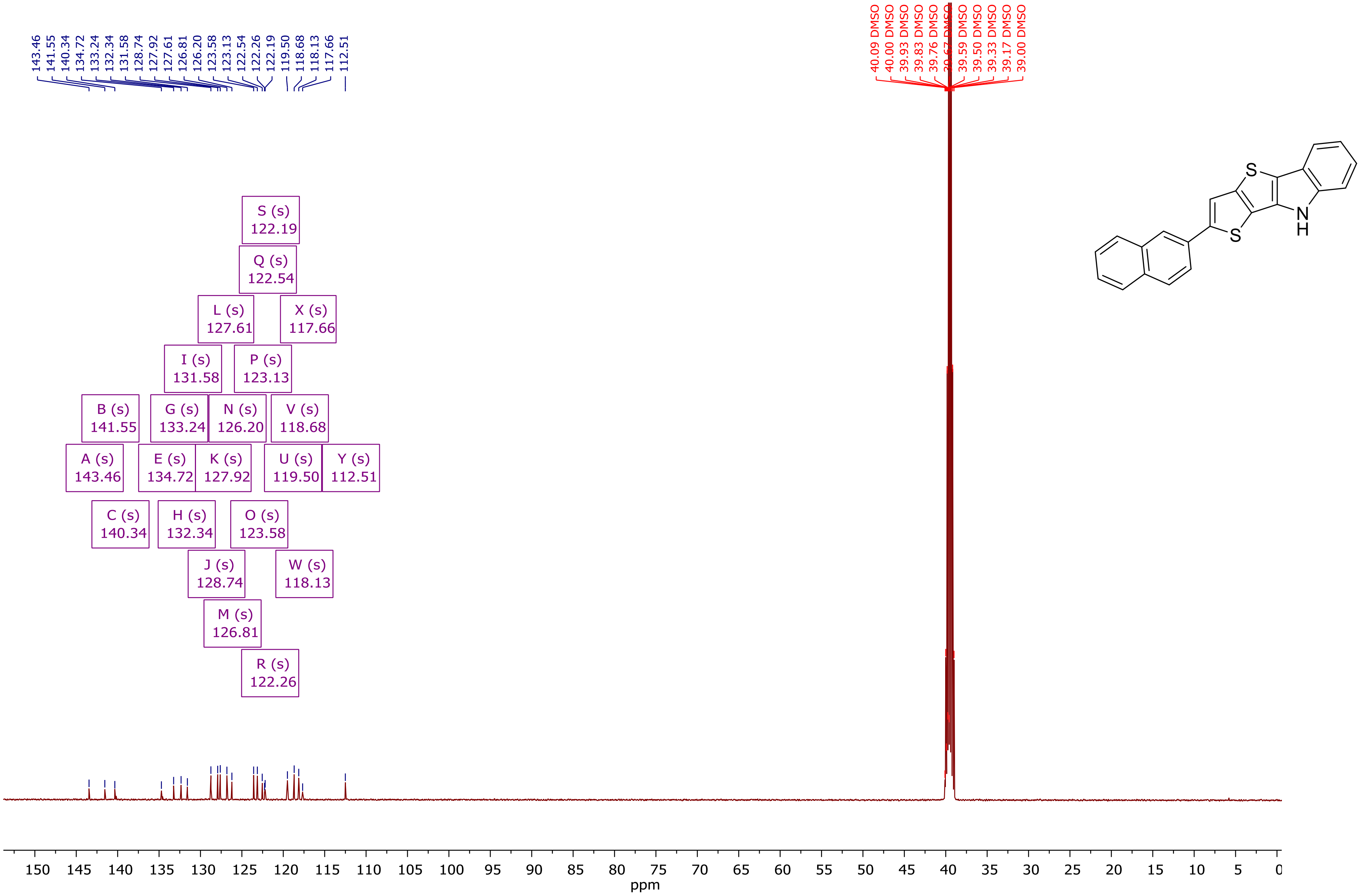

^{13}C NMR (126 MHz, DMSO-d_6) δ 143.5, 141.5, 140.3, 134.7, 133.2, 132.3, 131.6, 128.7, 127.9, 127.6, 126.8, 126.2, 123.6, 123.1, 122.5, 122.3, 122.2, 119.5, 118.7, 118.1, 117.7, 112.5.

3-Phenyl-9*H*-thieno[2',3':4,5]thieno[3,2-*b*]indole (6k)

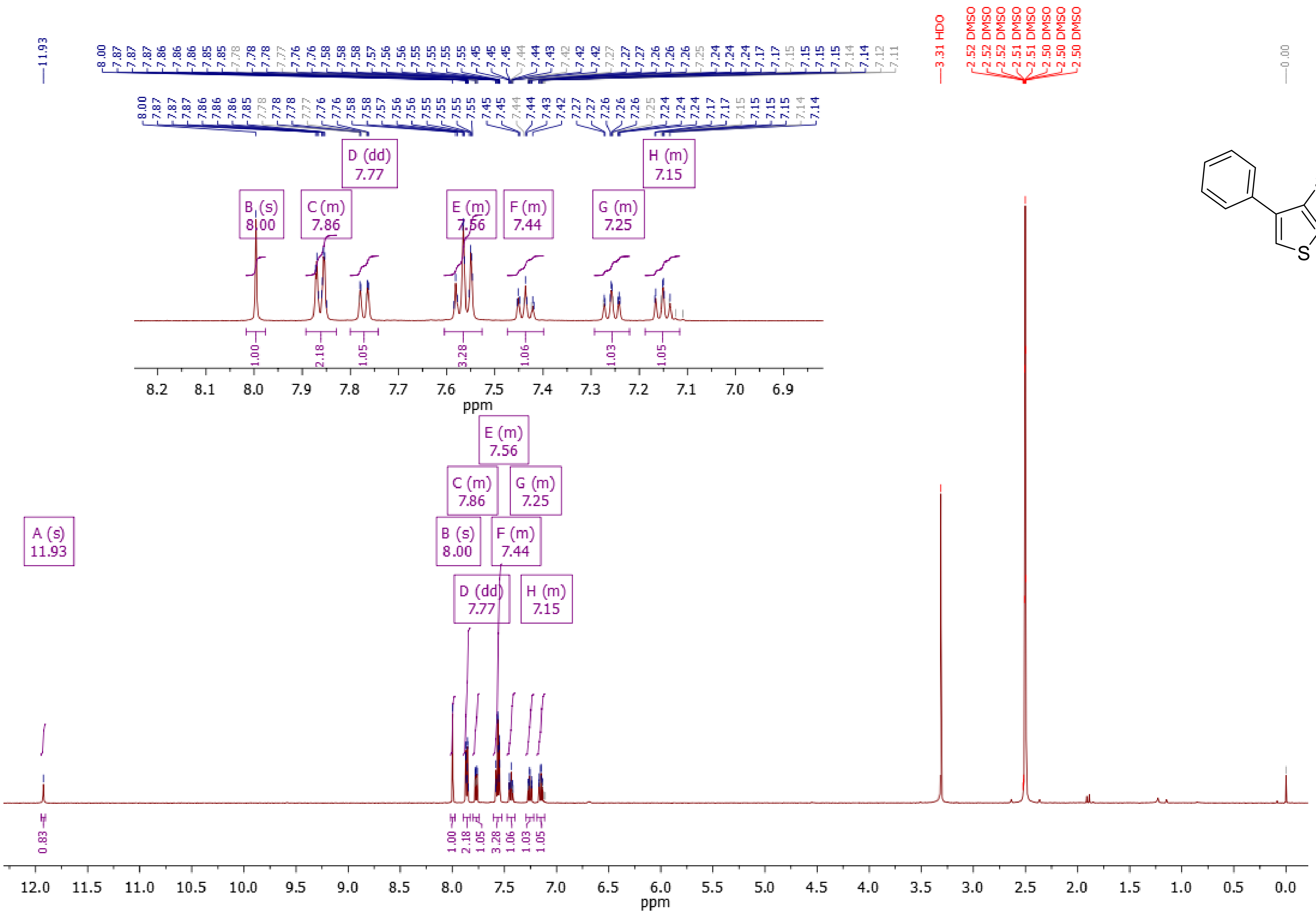

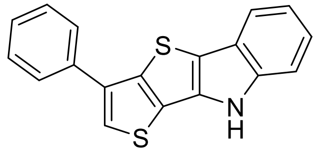

^{1}H NMR (500 MHz, DMSO-d_6) δ 11.93 (s, 1H), 8.00 (s, 1H), 7.89 – 7.83 (m, 2H), 7.77 (dd, *J* = 7.9, 1.1 Hz, 1H), 7.60 – 7.53 (m, 3H), 7.47 – 7.40 (m, 1H), 7.29 – 7.22 (m, 1H), 7.19 – 7.12 (m, 1H).

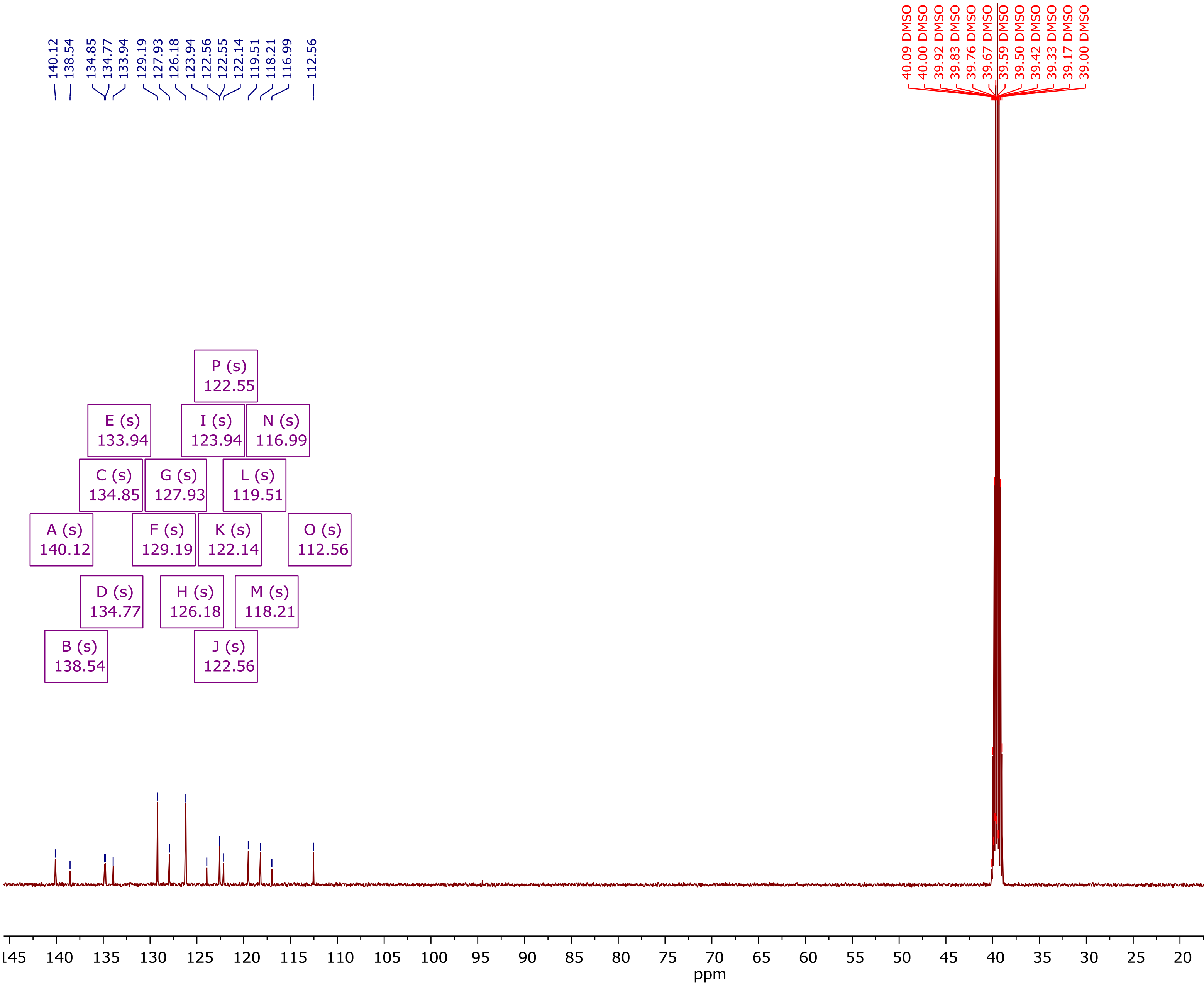

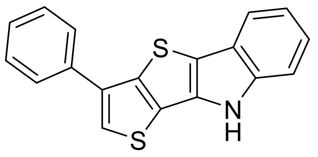

^{13}C NMR (126 MHz, DMSO-*d*$_6$) δ 140.1, 138.5, 134.85, 134.77, 133.9, 129.2, 127.9, 126.2, 123.9, 122.6, 122.5, 122.1, 119.5, 118.2, 117.0, 112.6.

2-(4-(*Tert*-butyl)phenyl)-6-methyl-9*H*-thieno[2',3':4,5]thieno[3,2-*b*]indole (6l)

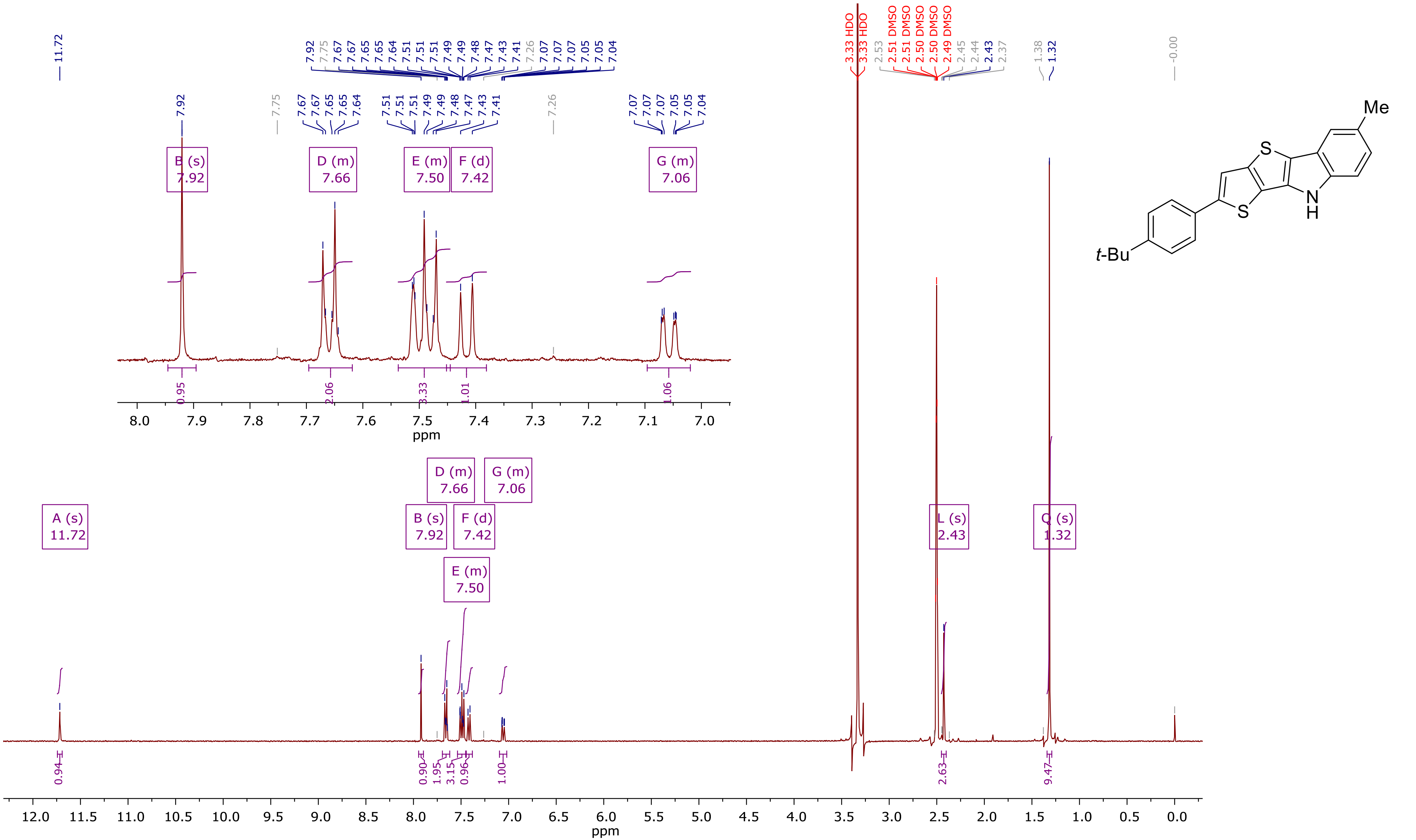

^{1}H NMR (400 MHz, DMSO-d_6) δ 11.72 (s, 1H), 7.92 (s, 1H), 7.70 – 7.62 (m, 2H), 7.54 – 7.45 (m, 3H), 7.42 (d, *J* = 8.3 Hz, 1H), 7.10 – 7.02 (m, 1H), 2.43 (s, 3H), 1.32 (s, 9H).

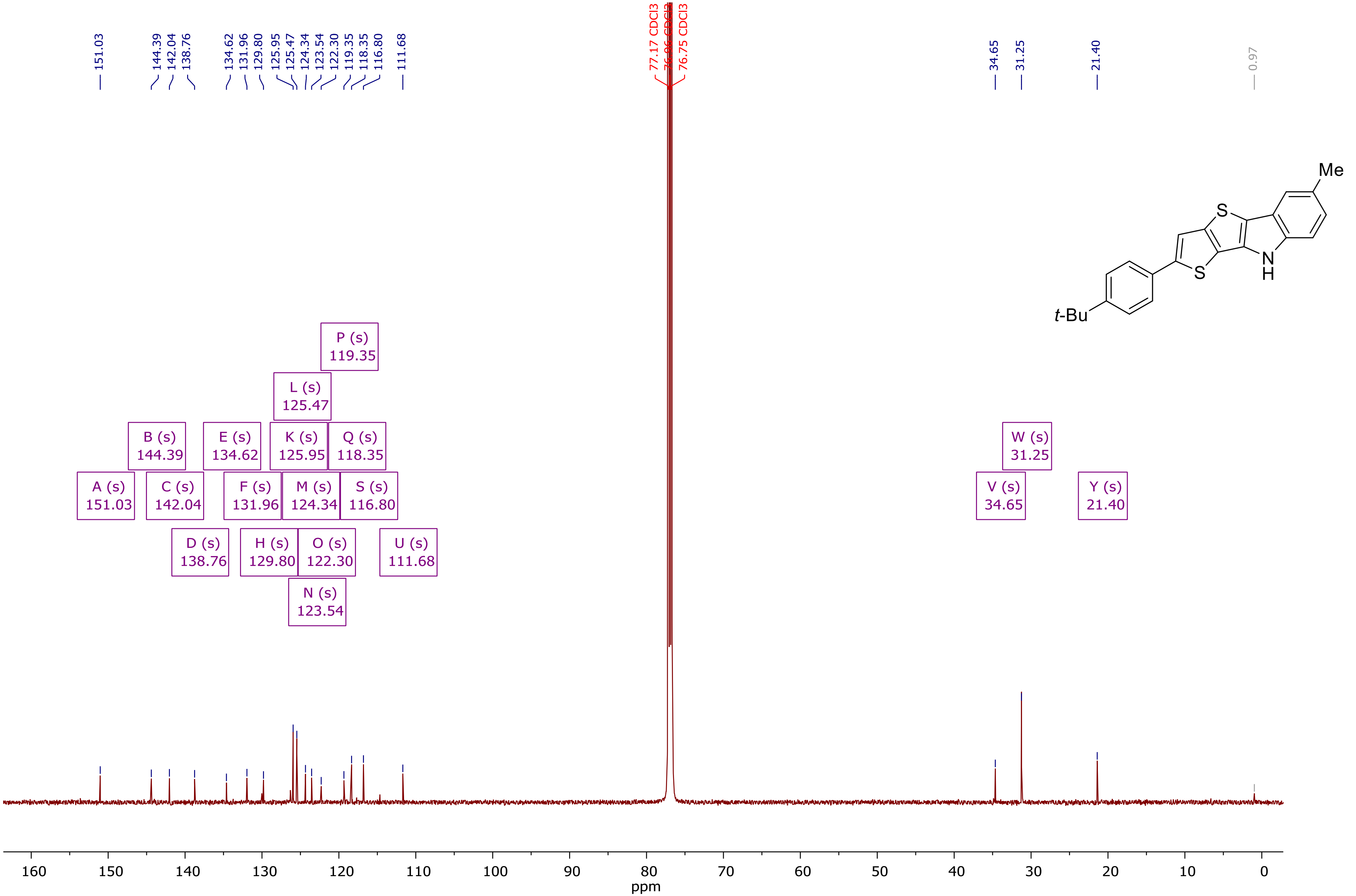

^{13}C NMR (151 MHz, chloroform-*d*) δ 151.0, 144.4, 142.0, 138.8, 134.6, 132.0, 129.8, 125.9, 125.5, 124.3, 123.5, 122.3, 119.3, 118.3, 116.8, 111.7, 34.6, 31.2, 21.4.

9-(4-(*Tert*-butyl)phenyl)-11*H*-benzo[*g*]thieno[2',3':4,5]thieno[3,2-*b*]indole (6m)

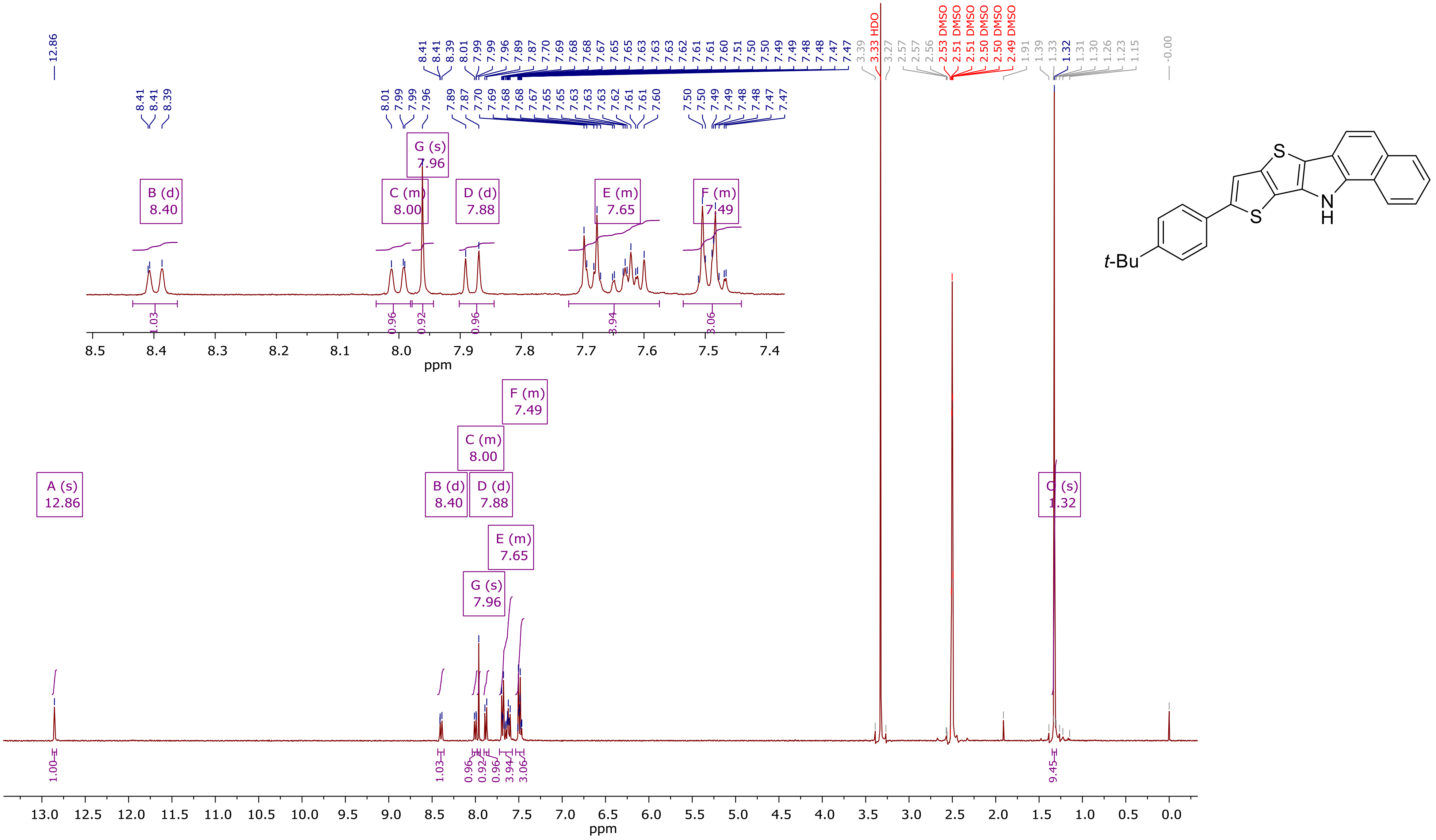

^{1}H NMR (400 MHz, DMSO-d_6) δ 12.86 (s, 1H), 8.40 (d, *J* = 8.1 Hz, 1H), 8.04 – 7.98 (m, 4H), 7.96 (s, 1H), 7.88 (d, *J* = 8.6 Hz, 2H), 7.72 – 7.57 (m, 4H), 7.54 – 7.44 (m, 3H), 1.32 (s, 9H).

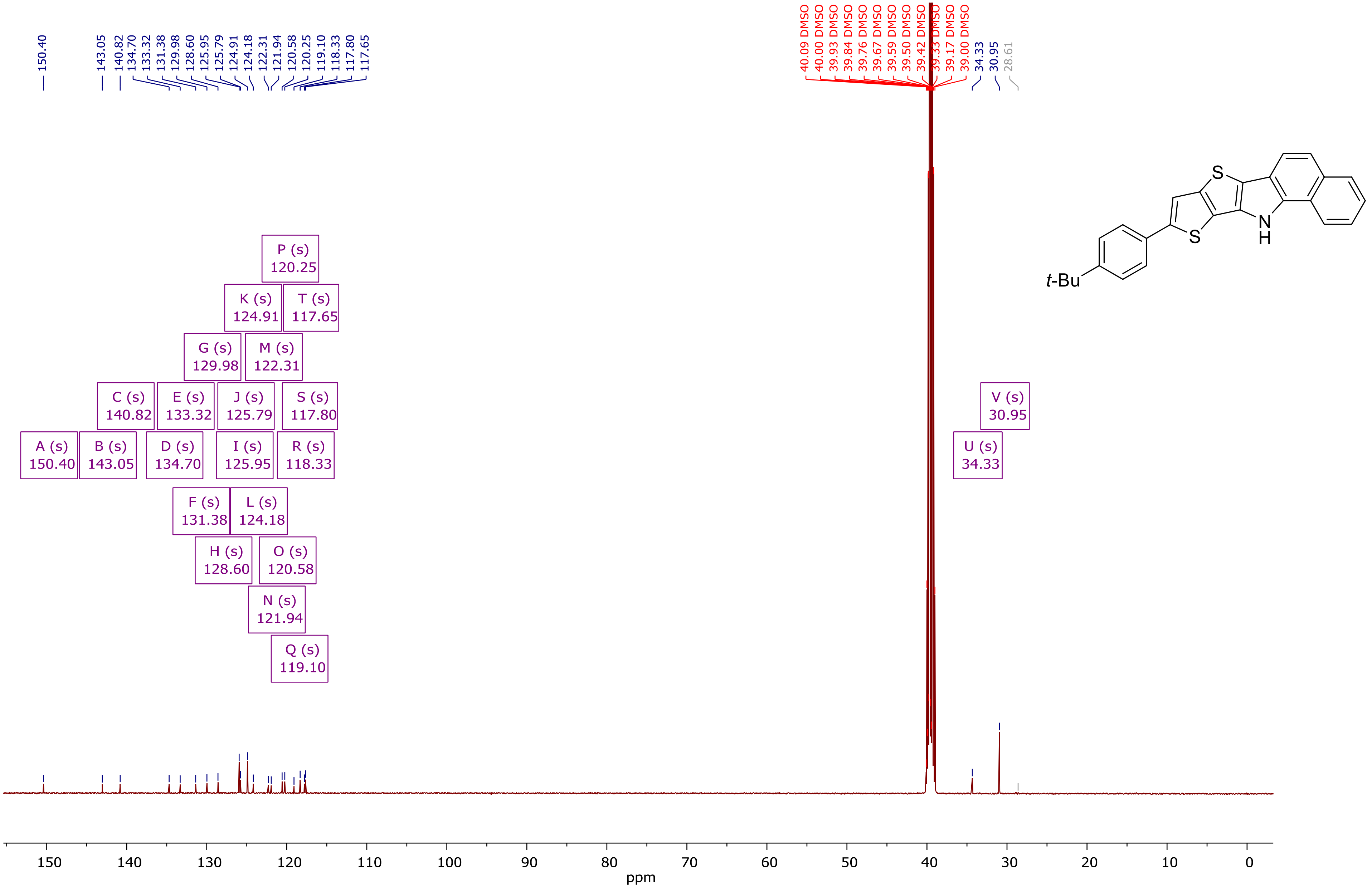

^{13}C NMR (126 MHz, DMSO-d_6) δ 150.4, 143.0, 140.8, 134.7, 133.3, 131.4, 130.0, 128.6, 125.9, 125.8, 124.9, 124.2, 122.3, 121.9, 120.6, 120.2, 119.1, 118.3, 117.8, 117.6, 34.3, 30.9.

6-(*Tert*-butyl)-2-(4-(*tert*-butyl)phenyl)-9*H*-thieno[2',3':4,5]thieno[3,2-*b*]indole (6n)

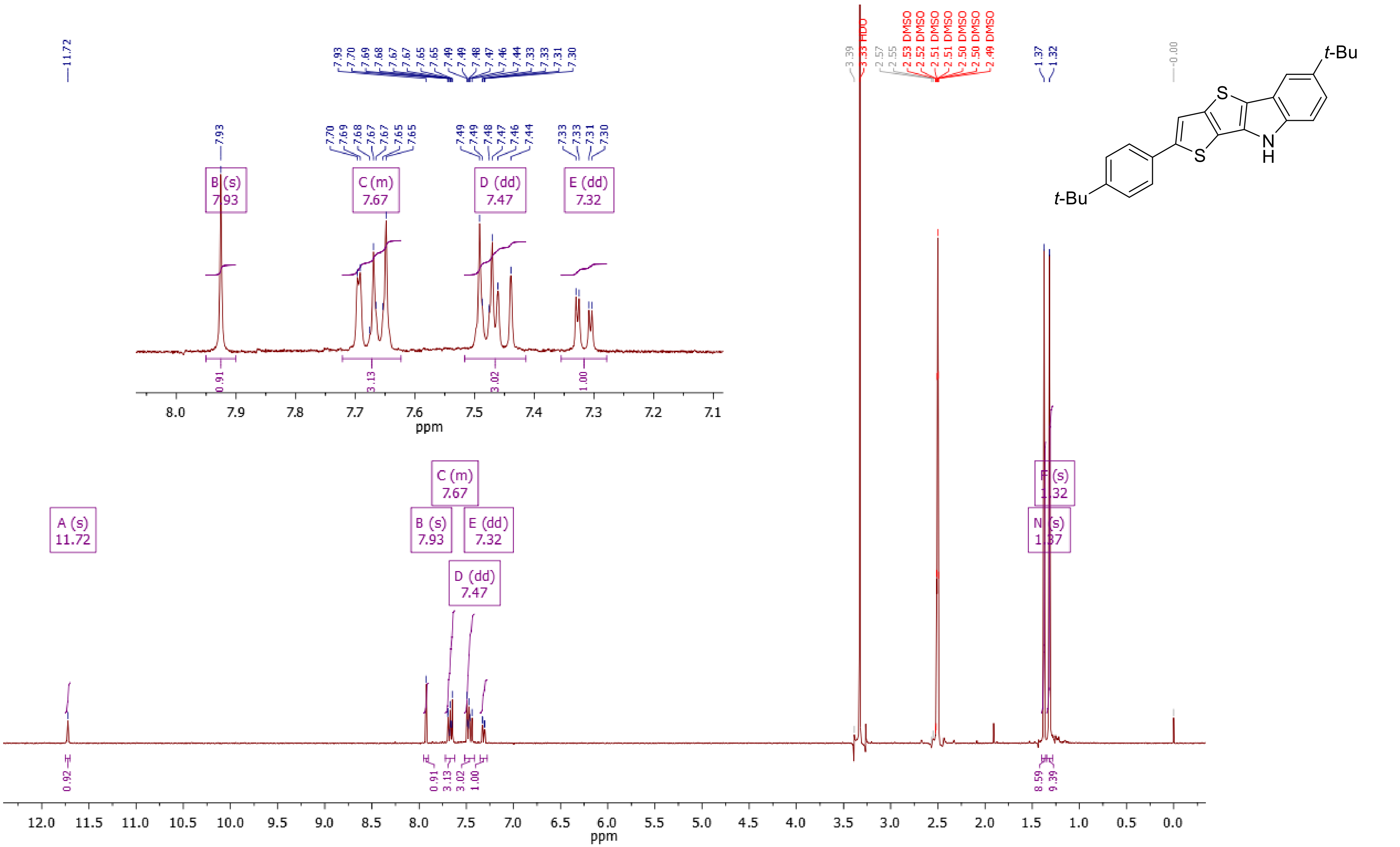

^{1}H NMR (400 MHz, DMSO-d_6) δ 11.72 (s, 1H), 7.93 (s, 1H), 7.72 – 7.62 (m, 3H), 7.47 (dd, *J* = 12.4, 8.6 Hz, 3H), 7.32 (dd, *J* = 8.6, 1.9 Hz, 1H), 1.37 (s, 9H), 1.32 (s, 9H).

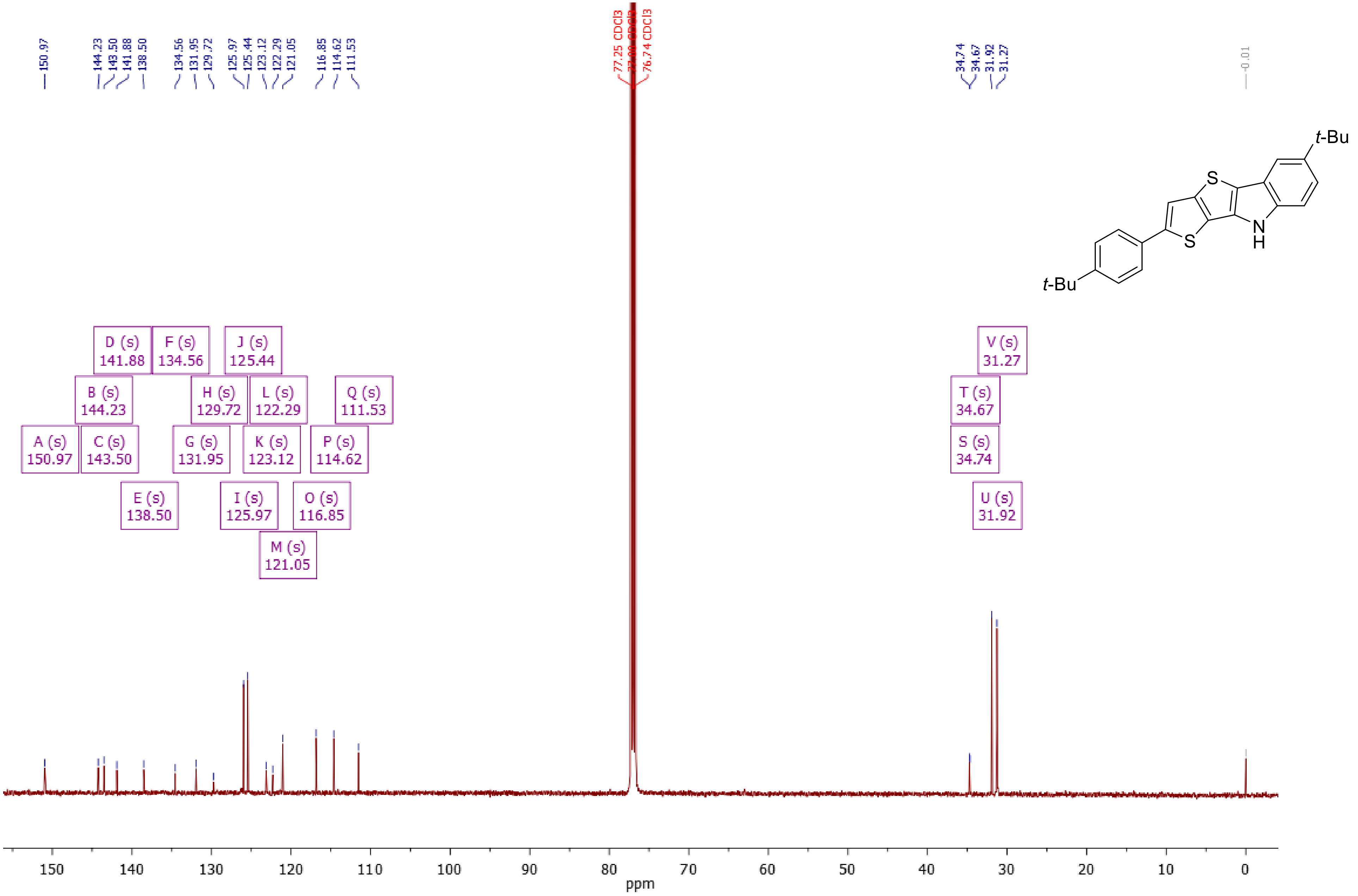

^{13}C NMR (126 MHz, chloroform-*d*) δ 151.0, 144.2, 143.5, 141.9, 138.5, 134.6, 131.9, 129.7, 126.0, 125.4, 123.1, 122.3, 121.0, 116.8, 114.6, 111.5, 34.74, 34.67, 31.9, 31.3.

2-(4-(*Tert*-butyl)phenyl)-9*H*-thieno[2',3':4,5]thieno[3,2-*b*]indole-6-carbonitrile (6o)

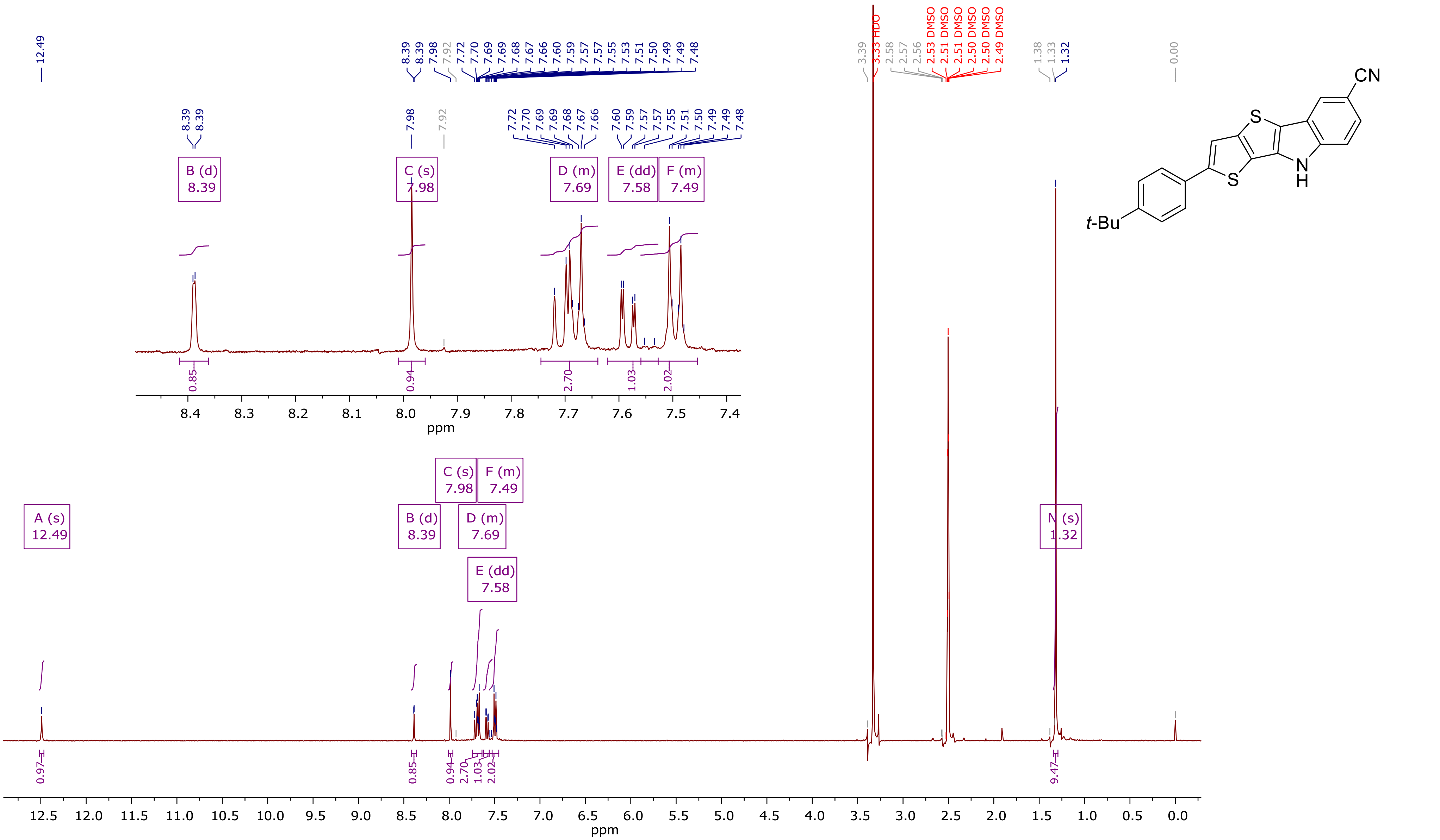

^{1}H NMR (400 MHz, DMSO-d_6) δ 12.49 (s, 1H), 8.39 (d, *J* = 1.5 Hz, 1H), 7.98 (s, 1H), 7.74 – 7.64 (m, 3H), 7.58 (dd, *J* = 8.5, 1.6 Hz, 1H), 7.56 – 7.45 (m, 2H), 1.32 (s, 9H).

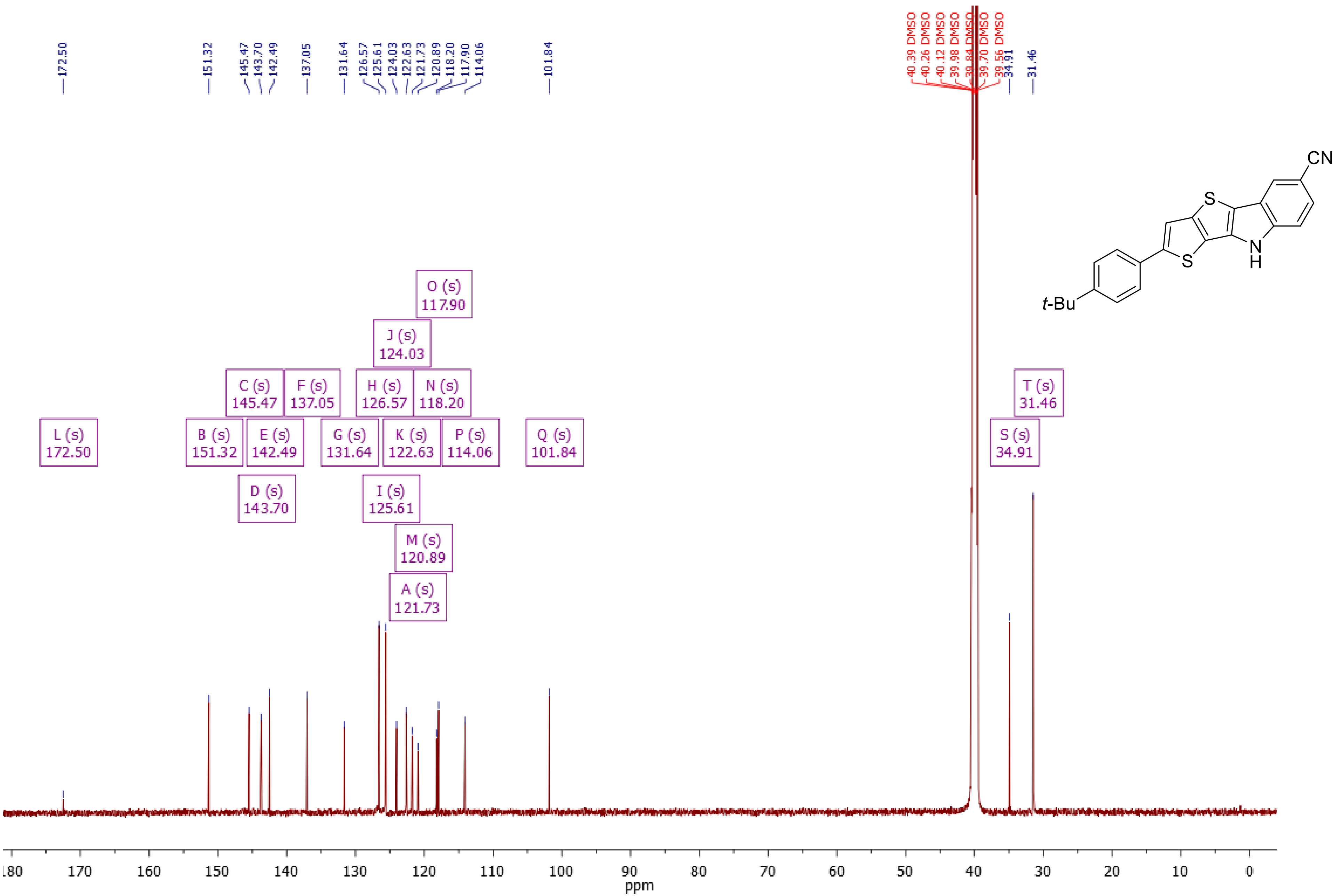

^{13}C NMR (151 MHz, DMSO-d_6) δ 172.5, 151.3, 145.5, 143.7, 142.5, 137.0, 131.6, 126.6, 125.6, 124.0, 122.6, 121.7, 120.9, 118.2, 117.9, 114.1, 101.8, 34.9, 31.5.

9-Benzyl-2-(4-(*tert*-butyl)phenyl)-9*H*-thieno[2',3':4,5]thieno[3,2-*b*]indole (7d)

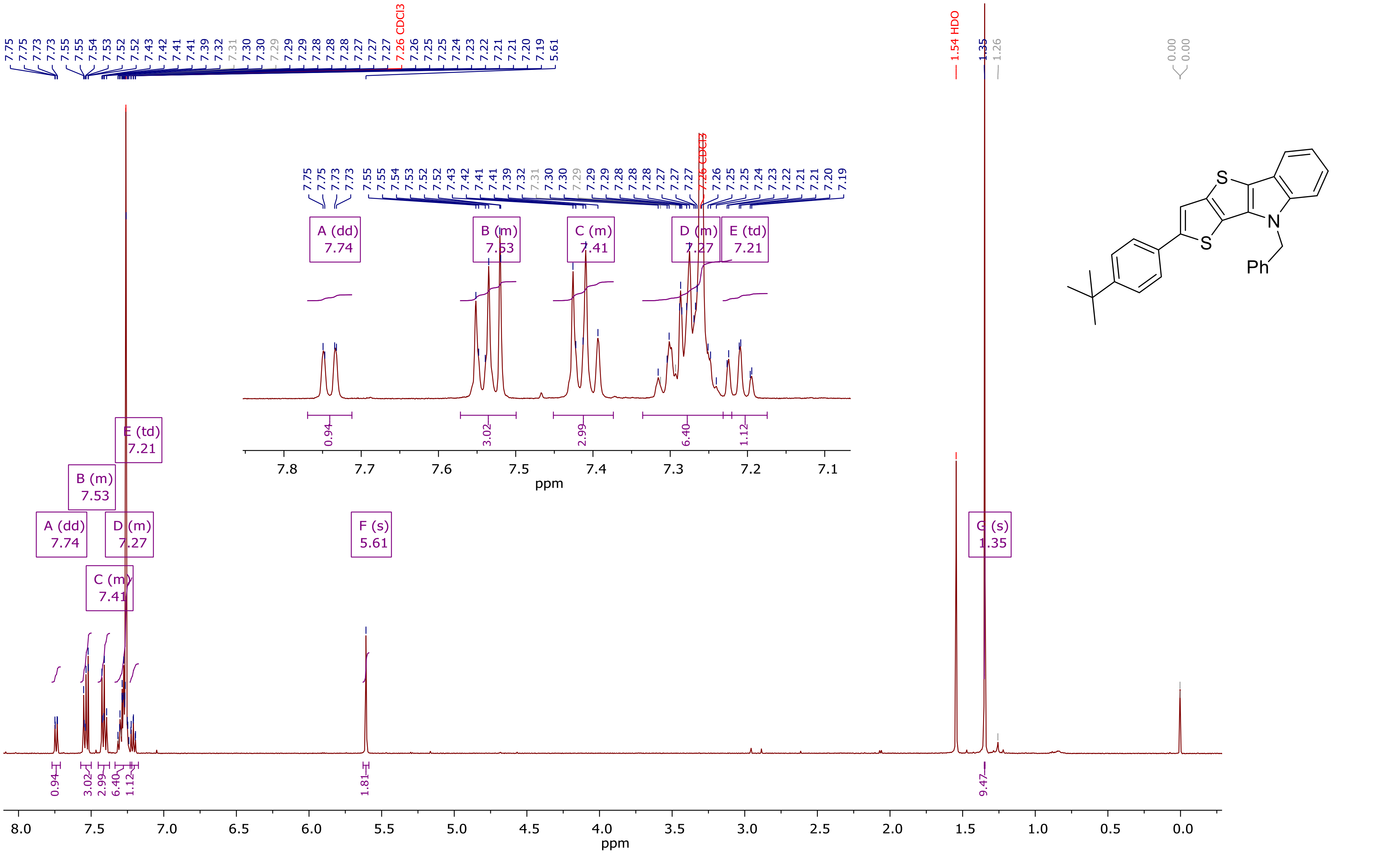

^{1}H NMR (500 MHz, chloroform-*d*) δ 7.74 (dd, *J* = 7.5, 1.2 Hz, 1H), 7.57 – 7.50 (m, 3H), 7.45 – 7.37 (m, 3H), 7.34 – 7.22 (m, 6H), 7.21 (td, *J* = 7.5, 1.0 Hz, 1H), 5.61 (s, 2H), 1.35 (s, 9H).

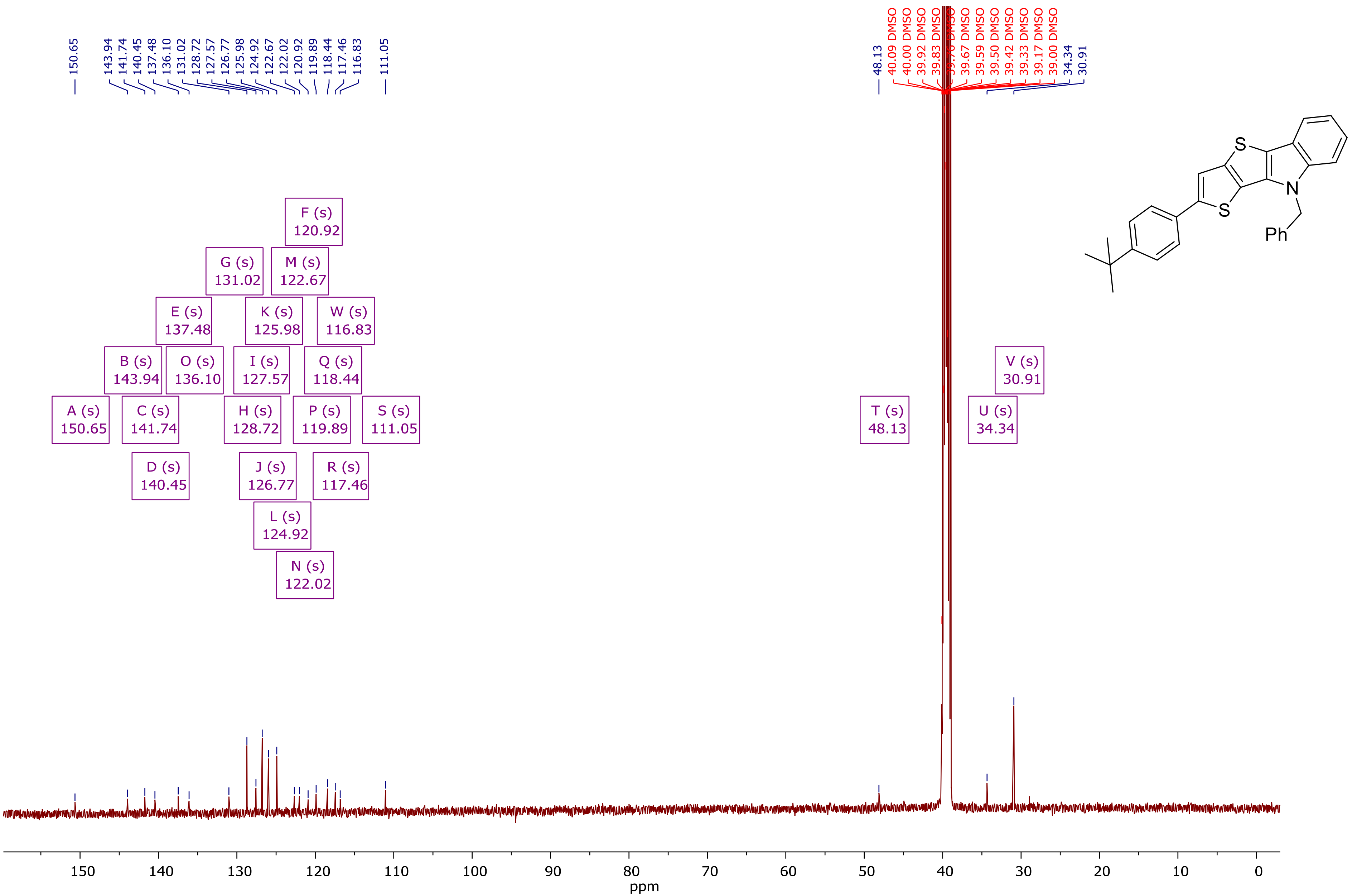

^{13}C NMR (126 MHz, DMSO-d_6) δ 150.6, 143.9, 141.7, 140.4, 137.5, 136.1, 131.0, 128.7, 127.6, 126.8, 126.0, 124.9, 122.7, 122.0, 120.9, 119.9, 118.4, 117.5, 116.8, 111.0, 48.1, 34.3, 30.91.

Crystallographic data and results of refinement for the structure 7d in the XRD experiment

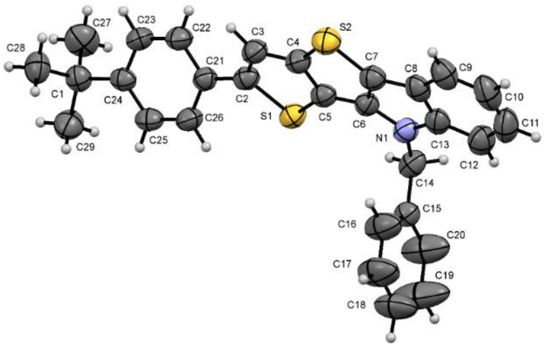

Compound **7d** in according XRD data. Thermal ellipsoids are shown at 50% probability level.

Deposition number CCDC 1944913 contains the supplementary crystallographic data for this structure. These data can be obtained free of charge from the Cambridge Crystallographic Data Centre via www.ccdc.cam.ac.uk/data_request/cif.

Compound **7d**	
Empirical formula	$C_{29}H_{25}NS_2$
Formula weight	451.62
Temperature/K	295(2)
Crystal system	triclinic
Space group	P-1
a/Å	9.5618(7)
b/Å	12.3346(9)
c/Å	20.6715(14)
α/°	93.930(6)
β/°	101.275(6)
γ/°	96.220(6)
Volume/Å^3	2366.8(3)
Z	2
ρ_{calc} mg/mm^3	1.267
m/mm^{-1}	0.242
F(000)	952
Crystal size/mm^3	0.43 × 0.36 × 0.21
2Θ range for data collection	3.57 < Θ < 30.50°
Index ranges	-12 < h < 12, -16 < k < 15, -29 < l < 29
Reflections collected	22864
Independent reflections	12841 [R(int) = 0.0466]
Data/restraints/parameters	12841 / 0 / 613
Goodness-of-fit on F^2	1.001
Final R indexes [I>2σ (I)]	R_1 = 0.0652, wR_2 = 0.1309
Final R indexes [all data]	R_1 = 0.1730, wR_2 = 0.1931
Largest diff. peak/hole / e Å^{-3}	0.233 / -0.332

References

1. Spasov, A.A.; Chepljaeva, N.I.; Vorob’ev, E.S. *Russ. J. Bioorganic Chem.* **2016**, *42* (2), 133–142. doi:10.1134/S1068162016020138.
2. Kim, Y.; Kim, J.; Kim, S.; Ki, Y.; Seo, S.H.; Tae, J.; Ko, M.K.; Jang, H.S.; Lim, E.J.; Song, C.; Cho, Y.; Koh, H.Y.; Chong, Y.; Choo, I.H.; Keum, G.; Min, S.J.; Choo, H. *Eur. J. Med. Chem.* **2014**, *85*, 629–637. doi:10.1016/j.ejmech.2014.08.027.
3. Jourdan, F.; Ladurée, D.; Robba, M. *J. Heterocycl. Chem.* **1994**, *31* (2), 305–312. doi:10.1002/jhet.5570310208.
4. Chen, L.; Bruneau, C.; Dixneuf, P.H.; Doucet, H. *Green Chem.* **2012**, *14* (4), 1111. doi:10.1039/c2gc16460d.
